# Asymmetric Isothiourea-Catalysed Formal [3+2] Cycloadditions of Ammonium Enolates with Oxaziridines

**DOI:** 10.1002/chem.201501271

**Published:** 2015-06-12

**Authors:** Siobhan R Smith, Charlene Fallan, James E Taylor, Ross McLennan, David S B Daniels, Louis C Morrill, Alexandra M Z Slawin, Andrew D Smith

**Affiliations:** [a] EaStCHEM, School of Chemistry, University of St Andrews North Haugh, St Andrews, KY16 9ST (UK) E-mail: ads10@st.andrews.ac.uk

**Keywords:** asymmetric synthesis, heterocycles, Lewis base, organocatalysis, oxaziridines

## Abstract

A highly enantioselective Lewis base-catalysed formal [3+2] cycloaddition of ammonium enolates and oxaziridines to give stereodefined oxazolidin-4-ones in high yield is described. Employing an enantioenriched oxaziridine in this process leads to a matched/mis-matched effect with the isothiourea catalyst and allowed the synthesis of either *syn-* or *anti*-stereodefined oxazolidin-4-ones in high d.r., yield and *ee*. Additionally, the oxazolidin-4-one products have been derivatised to afford functionalised enantioenriched building blocks.

## Introduction

The ubiquitous use of heterocycles in the pharmaceutical, agrochemical as well as in the dye and fine-chemical industries has led to the establishment of numerous strategies for their synthesis and functionalisation.[1] Stereodefined heterocycles are also significant components of numerous biologically active natural products.[2] As a result of the widespread prevalence of heterocyclic motifs in synthetic chemistry,[3] alongside the continued drive for efficient, selective synthetic protocols within the chemical community, there is an ongoing requirement for novel asymmetric syntheses of heterocyclic scaffolds.

**Figure 1:**
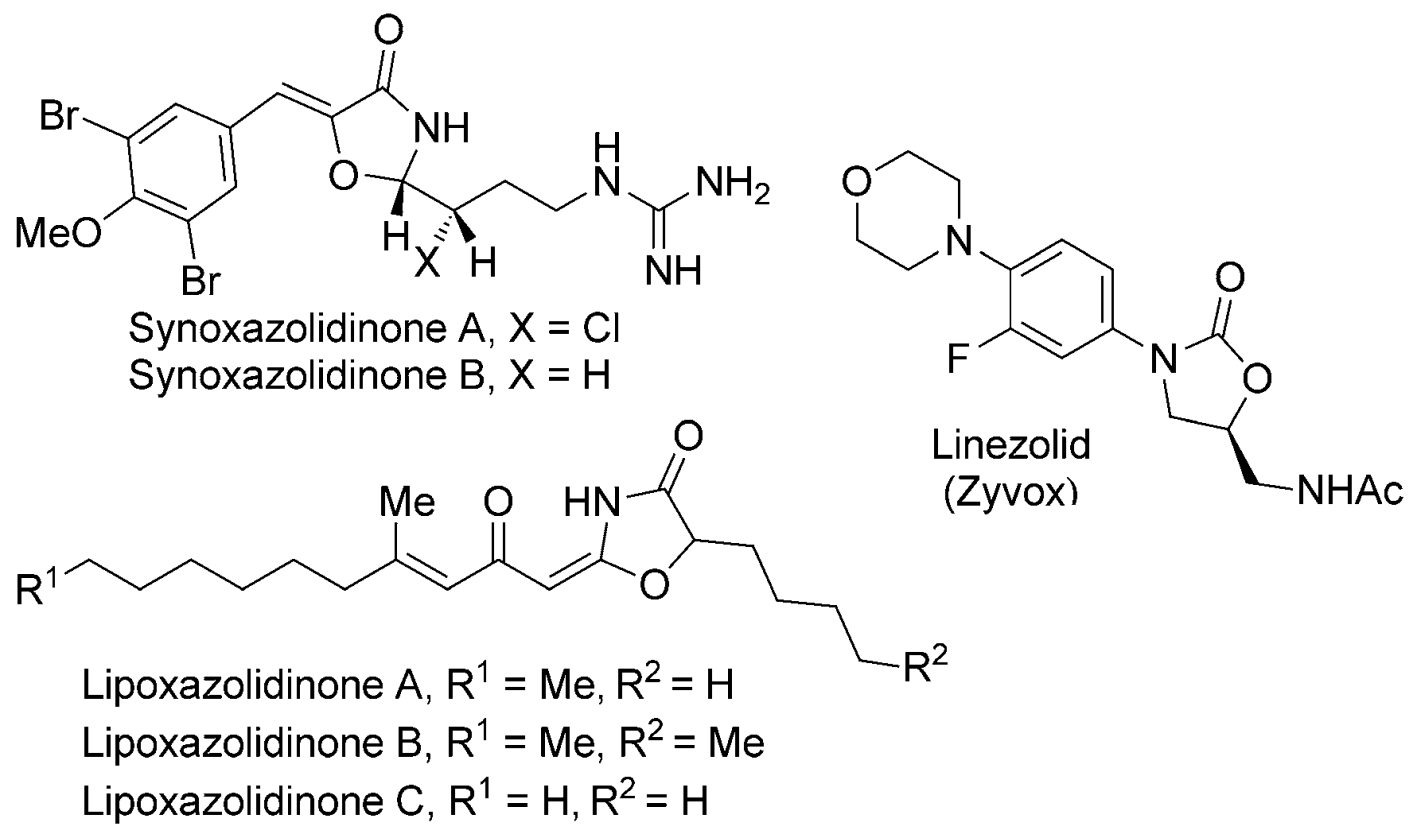
Biologically active natural products and medicinal agents based upon the oxazolidinone core.

Oxazolidin-4-ones represent a unique heterocyclic structural motif found within natural products and bioactive molecules. For example, the oxazolidin-4-one core is found in the natural products synoxazolidinone A and B which were isolated from *S. pulmonaria* and exhibit antibiotic and antifungal activity at low concentrations (Figure 1).[4] In addition, oxazolidin-4-ones are found in lipoxazolidinones A, B, and C isolated from a marine actinomycete strain.[5] These naturally occurring oxazolidin-4-ones also exhibit antibacterial activity comparable with the commercial antibacterial agent Linezolid (Zyvox) that contains a structurally related oxazolidin-2-one core.[6] Therefore, the development of a synthetic strategy for the asymmetric generation of heterocyclic scaffolds of this type is a worthwhile goal. In this manuscript, we describe an isothiourea-catalysed formal [3+2] cycloaddition using both racemic and enantioenriched oxaziridines[7] to form stereodefined oxazolidin-4-ones.

Building on Birman and Okamoto’s introduction of isothiourea catalysts for kinetic resolutions[8] we have recently established, alongside Romo,[9] isothiourea Lewis base catalysis[10] for the preparation of a range of synthetically relevant heterocyclic scaffolds. Substituted THFs,[11] dihydrobenzofurans and pyrrolidines[12] have been accessed by an asymmetric intramolecular Michael addition/lactonisation process. In addition, stereodefined *anti*-δ-lactams[13] and dihydropyranones[14] were obtained by related intermolecular Michael addition/cyclisation protocols. This methodology was extended using a strategic PhSH elimination as part of a cascade process for the synthesis of substituted pyrones[15] and functionalised pyridines.[16] Additionally, asymmetric formal [2+2] cycloadditions employing *N*-sulfonyl imines to form *anti*-β-lactams have been studied.[17] However, to date, formal [3+2] cycloaddition processes catalysed by isothioureas have not been developed.[18]

**Scheme 1:**
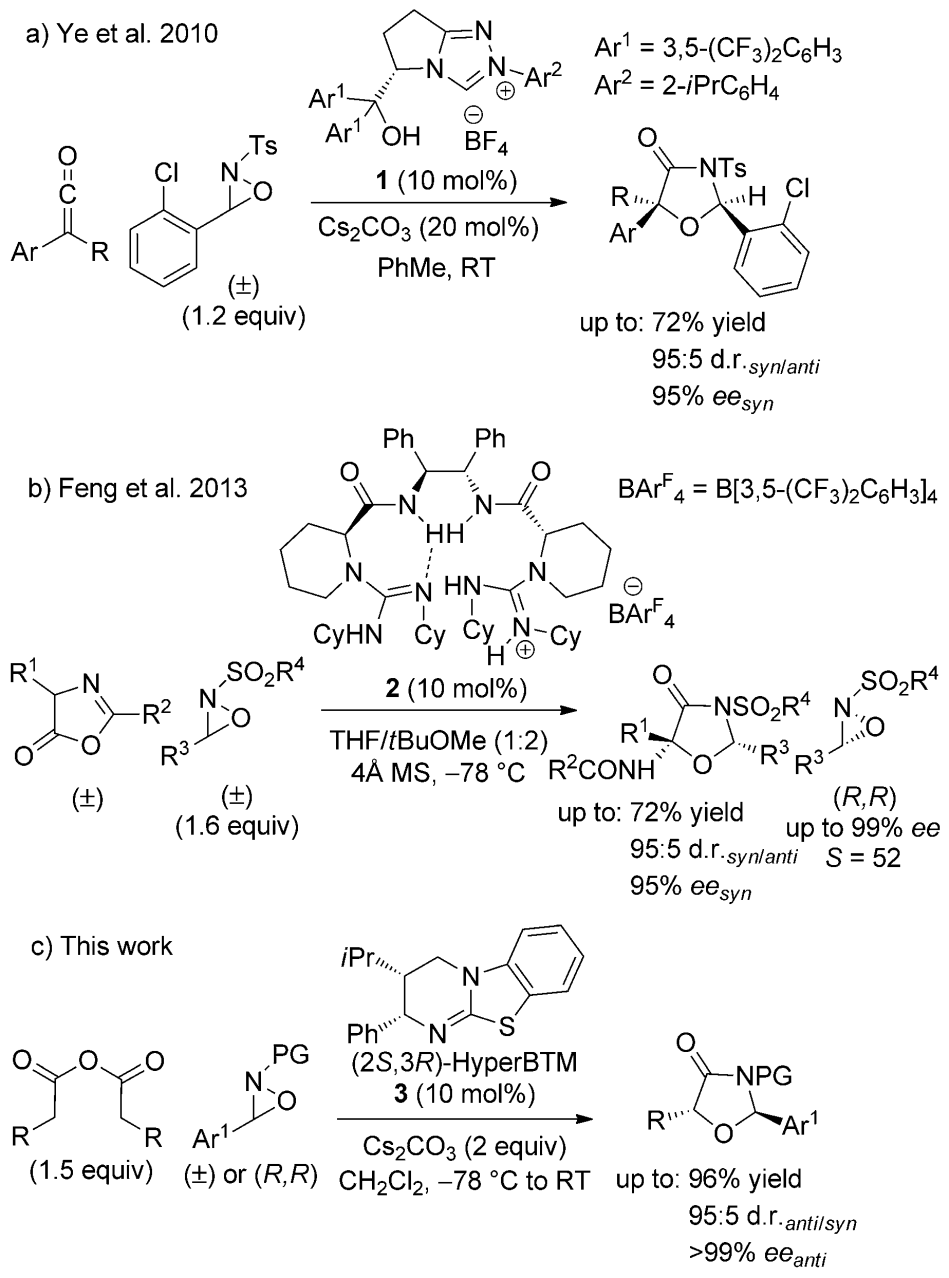
Formal [3+2] intermolecular cycloadditions for the synthesis of oxazolidin-4-ones catalysed by a) NHC precatalyst 1; b) bisguanidinium salt 2; c) HyperBTM 3.

Oxaziridines have previously been reported as electrophiles for the synthesis of oxazolidin-4-ones by Ye and co-workers using ketenes in the presence of either N-heterocyclic carbene (NHC) precatalyst **1** or cinchona alkaloids.[19] The α,α-disubstituted oxazolidin-4-ones were isolated in good yield and with high diastereo- and enantioselectivity (Scheme 1a), although this process is somewhat limited due to the use of synthetically challenging ketenes and their precursors. More recently, Feng described chiral bisguanidinium salt **2** for the asymmetric oxyamination of azlactones with concurrent kinetic resolution of the oxaziridine (Scheme 1 b).[20] Building upon these precedents, herein we report our results on the isothiourea-catalysed asymmetric formal [3+2] cycloaddition of homoanhydrides and oxaziridines to form stereodefined oxazolidin-4-ones (Scheme 1c) and their subsequent derivatisations.

## Results and Discussion

### Optimisation

**Scheme 2:**
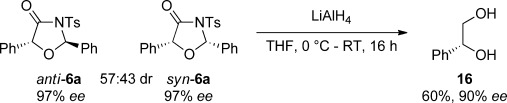
LiAlH_4_ reduction of oxazolidin-4-one 6.

Our investigation began with the Lewis base-catalysed reaction of commercially available phenylacetic acid **4** with racemic oxaziridine **5** (Table 1, conditions A). Treatment of the acid with pivaloyl chloride and *i*Pr_2_NEt to form a mixed anhydride in situ followed by addition of (2*S*,3*R*)-HyperBTM **3**[21] and oxaziridine **5** gave high conversion into the desired [3+2] oxazolidin-4-one product **6**, with a small amount of imine **14** and β-lactam **15** (derived from a previously disclosed[17] intramolecular formal [2+2] cycloaddition of an ammonium enolate and imine **14**) also observed by ^1^H NMR spectroscopy (Table 1, entry 1). However, imine **14** was difficult to remove from the desired product by column chromatography, resulting in contaminated oxazolidin-4-ones. To probe the origin of imine **14**, control experiments demonstrated that treating oxaziridine **5** in CH_2_Cl_2_ with *i*Pr_2_NEt (1 equiv) led to the formation of *i*Pr_2_(Et)*N*-oxide and imine **14**. To prevent this undesired imine formation through oxidation of the base a number of alternative bases was examined. Disappointingly, 2,6-lutidine and Cs_2_CO_3_ gave comparable amounts of imine **14** (entries 2 and 3). In the reaction with Cs_2_CO_3_ (entry 3), imine formation is presumably derived from reaction of oxaziridine **5** with chloride ions[7b] generated from the reaction of phenylacetic acid **4** with pivaloyl chloride to form the “activated” mixed anhydride. To overcome this problem and remove the need for an activation step, homoanhydride[22] **7** was used in place of phenylacetic acid and assessed under similar reaction conditions (Table 1, conditions B). Pleasingly, this alternative ammonium enolate precursor resulted in formation of oxazolidin-4-one **6** exclusively in high yield with excellent enantiocontrol, however lower levels of diastereoselectivity were obtained (entry 4). The oxazolidin-4-one diastereomeric mixture **6 a** and **6 b** was reduced using LiAlH_4_ to give diol **16** in good yield maintaining stereointegrity (Scheme 2),[23] confirming that the configuration at C(5) is equivalent in both the *syn*- and *anti*-diastereomers formed. The absolute configuration was determined by comparison of the specific rotation of diol **16** with literature values (see the Supporting Information for details). To assess the effect of the oxaziridine on the stereochemical outcome of the process, alternative oxaziridines were investigated using phenylacetic anhydride **7** as the standard ammonium enolate precursor (entries 5–7). Aromatic halogen substitution in the *ortho*- and *para*-position was examined under the optimised conditions and led to high yields of the desired [3+2] products **11** and **12**, both in approximately 55:45 d.r. but with slightly reduced levels of enantiocontrol for both diastereoisomers. Gratifyingly, the scope of the process could be extended with regards to the N-substituent. Replacing the *N*-tosyl group with an *N*-nosyl led to the formation of oxazolidin-4-one **13** in high yield, however slightly reduced *ee* values were obtained for the *syn*- and *anti*-products.

**Table 1:** Reaction optimisation and oxaziridine scope

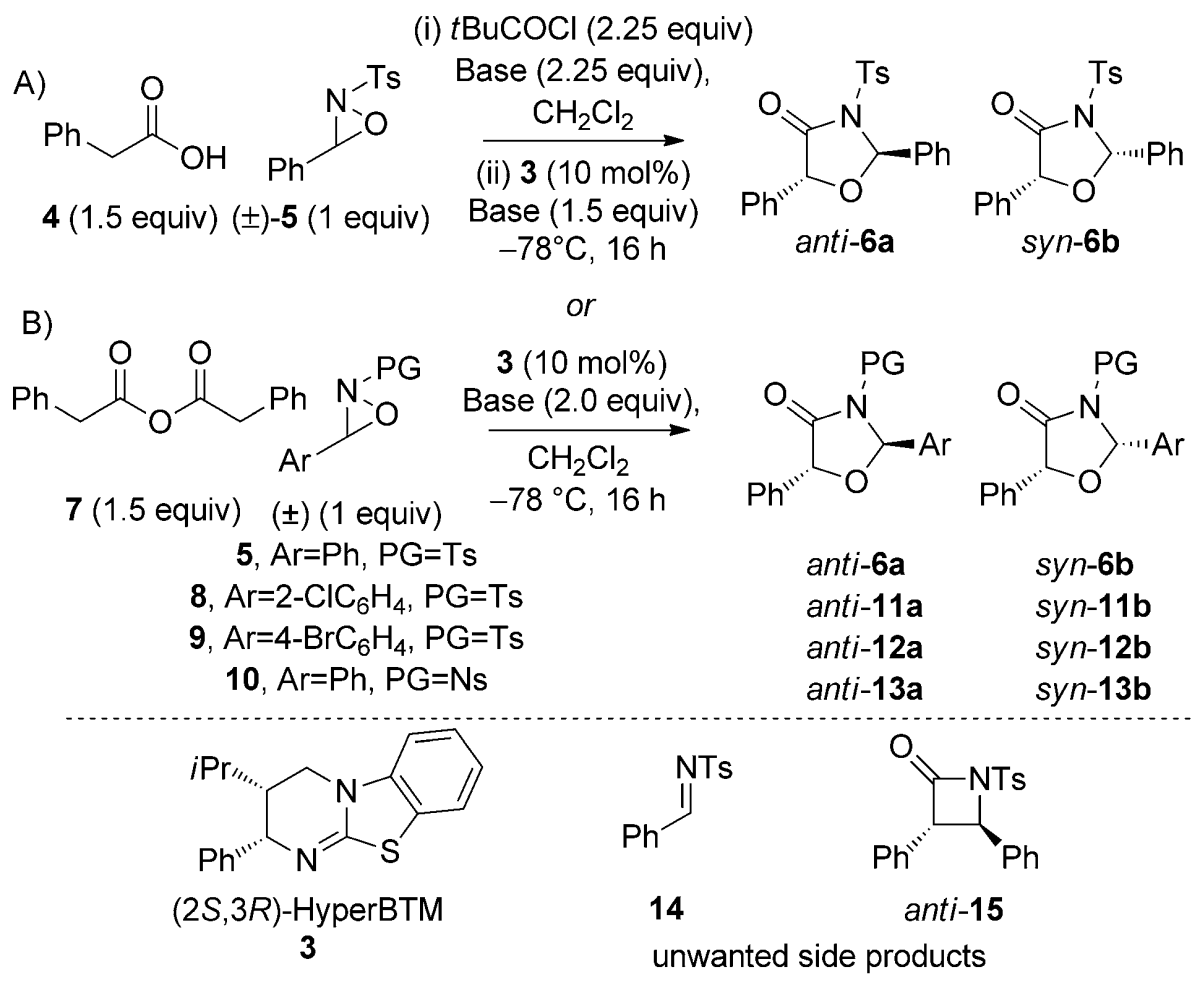							
Entry	Oxaziridine	Product	Base	Yield [%]^[a]^	d.r._*anti*/*syn*_^[b]^	*ee*_*anti*/*syn*_ [%]^[c]^	Imine14[%]
1^[d]^	**5**	**6**	*i*Pr_2_NEt	64	82:18	>99:>99	6
2^[d]^	**5**	**6**	2,6-lutidine	57	75:25	–	8
3^[d]^	**5**	**6**	Cs_2_CO_3_	72	61:39	–	9
4^[e]^	**5**	**6**	Cs_2_CO_3_	83	57:43	97:97	–
5^[e]^	**8**	**11**	Cs_2_CO_3_	78	55:45	78:78	–
6^[e]^	**9**	**12**	Cs_2_CO_3_	82	55:45	99:95	–
7^[e]^	**10**	**13**	Cs_2_CO_3_	73	59:41	85:80	–

[a] Combined isolated yield of both diastereoisomers. [b] Determined by ^1^H NMR spectroscopic analysis of the crude reaction product. [c] Determined by HPLC analysis. [d] Conditions A. [e] Conditions B.

These reaction conditions were next applied to a range of homoanhydrides to assess the scope of the reaction (Table 2). Anhydrides with both electron-withdrawing and -donating aromatic substituents were tolerated, giving a range of oxazolidin-4-ones in high yields with approximately 50:50 d.r., but with excellent levels of enantiocontrol observed for each diastereoisomer **6**, **17** and **18** (up to 99 % *ee*). Extended aromatic systems and aromatic groups bearing substituents in the *ortho*-, *meta*- and *para*-position also participated well under the previously optimised reaction conditions giving oxazolidin-4-ones **19**–**22** in good yields again with excellent levels of *ee* for both diastereoisomers. 3-Thiophenylacetic anhydride led to isolation of oxazolidin-4-one **23** in 79 % yield but lower levels of *ee* were obtained for both the *syn*- and *anti*-diastereoisomer (87 and 81 %, respectively). Pleasingly, the reaction was extended beyond aromatic substitution patterns to include alkenyl oxazolidin-4-one **24**, obtained in good yield and high *ee* (*syn*- and *anti*-diastereoisomer). Unexpectedly, *p*-trifluoromethyl substitution gave oxazolidin-4-one **25** in 49:51 d.r._*anti*/*syn*_, with both diastereoisomers formed with low levels of enantioselectivity (43 % *ee_anti_*, 36 % *ee_syn_*).

**Table 2:** Investigation of homoanhydride substrate scope

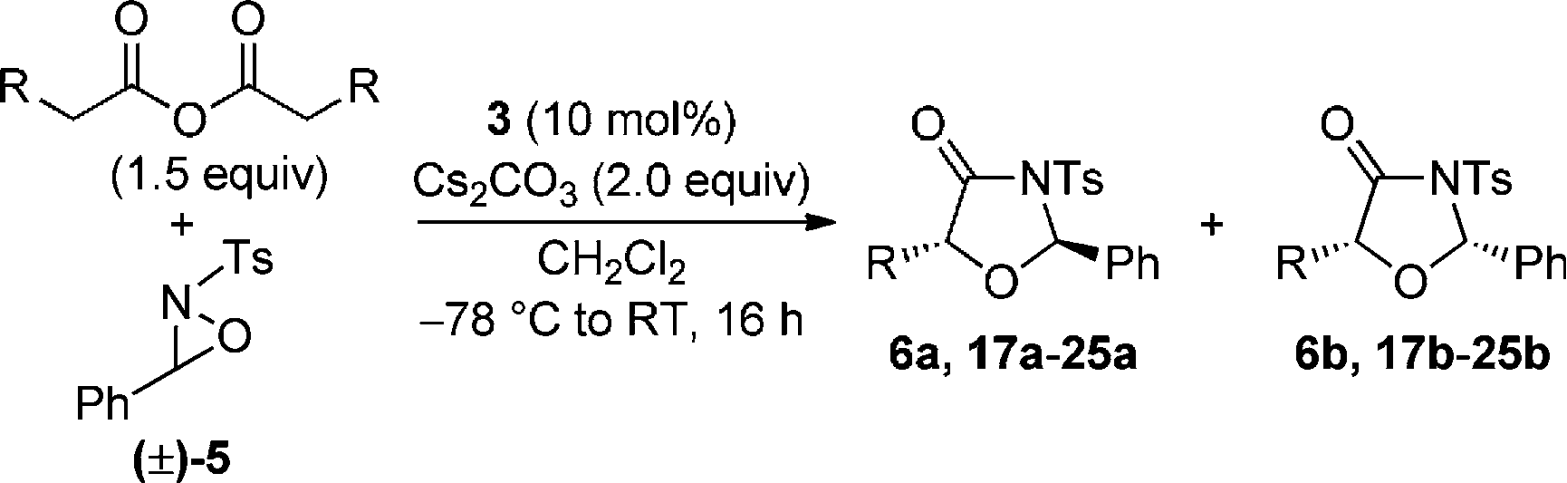			
Products	Yield [%]^[a]^ d.r._*anti*/*syn*_^[b]^ *ee_anti_*/*ee_syn_* [%]^[c]^	Products	Yield [%]^[a]^ d.r._*anti*/*syn*_^[b]^ *ee_anti_*/*ee_syn_* [%]^[c]^
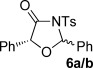	83 57:43 97:97	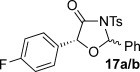	73 54:46 99:99
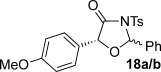	89 55:45 97:94	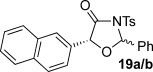	48 53:47 99:99
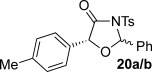	88 53:47 97:99	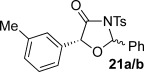	79 59:41 92:94
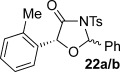	96 53:47 99:99	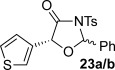	79 59:41 87:81
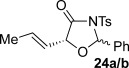	61 54:46 99:99	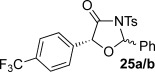	68 49:51 43:36

[a] Combined isolated yield of both diastereoisomers. [b] Determined by ^1^H NMR spectroscopic analysis of the crude reaction product. [c] Determined by HPLC analysis.

Whilst these results are synthetically relevant, their utility for the synthesis of oxazolidin-4-ones is partially limited due to the diastereomeric mixtures of heterocycles obtained. Although this methodology is applicable to the synthesis of enantioenriched diols (Scheme 2), further investigations sought to investigate the cause of low diastereocontrol in this process allowing selective access to either *syn* and *anti* diastereoisomers. The conversion of (±)-oxaziridine **5** into product **6** under the standard reaction conditions was monitored over time by ^1^H NMR spectroscopy and the *ee* of unreacted oxaziridine **5** and oxazolidin-4-one **6** was analysed by chiral HPLC analysis (Table 3). Notably, over the early part of the reaction the d.r. of **6** remains fairly constant with the initial d.r. of 78:22 *anti*/*syn* at 1 min, reducing to 71:29 after 4 h at −78 °C. The *ee* of both diastereoisomers of oxazolidin-4-one **6** remain consistently high throughout the duration of the reaction. Interestingly, the *ee* of the unreacted oxaziridine **5** gradually increased with conversion up to 41 % *ee* at 4 h, which indicates that a partial kinetic resolution was occurring under the reaction conditions. The *ee* values of **5** obtained experimentally in Table 3 correlate with the predicted values based upon the given conversion and d.r., within error. Significantly, high conversion was only achieved after an extended reaction time and upon warming to room temperature, which indicates that one enantiomer of the oxaziridine requires increased temperature to react efficiently with the ammonium enolate. This experiment also provided evidence that chirality transfer from (±)-oxaziridine **5** to product **6** was the cause of the low diastereocontrol in this process, which has implications with regard to the mechanism of this isothiourea-catalysed formal [3+2] process.

**Table 3:** Investigation of enantio- and diastereoselectivity over time

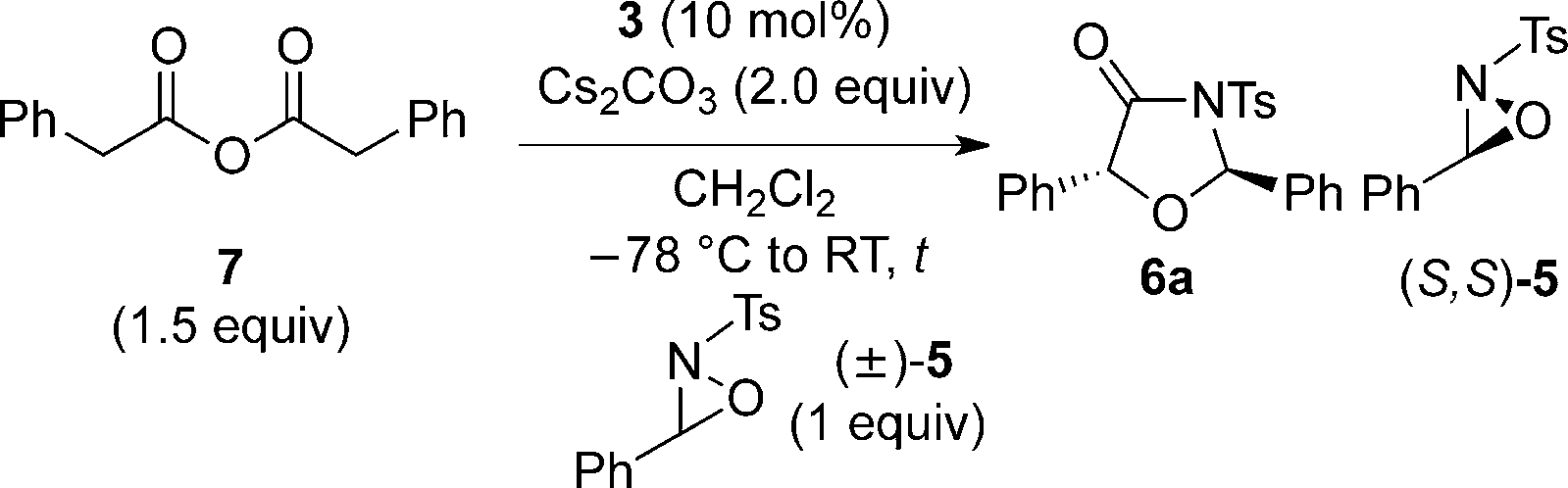				
*t*	Conv.^[a]^ [%]	d.r._*anti*/*syn*_^[b]^	6­ *ee_anti_*/*ee_syn_* [%]^[c]^	5­ *ee* [%]^[c]^
1 min	9	78:22	>99:99	7
5 min	24	75:25	>99:99	10
15 min	25	70:30	>99:99	11
30 min	26	74:26	>99:99	12
60 min	26	75:25	>99:99	13
120 min	30	75:25	>99:99	17
240 min	52	71:29	>99:99	41
18 h	91	59:41	>99:99	–

[a] Determined by ^1^H NMR spectroscopic analysis. [b] Determined by ^1^H NMR spectroscopic analysis of the crude reaction product. [c] Determined by HPLC analysis.

**Scheme 3:**
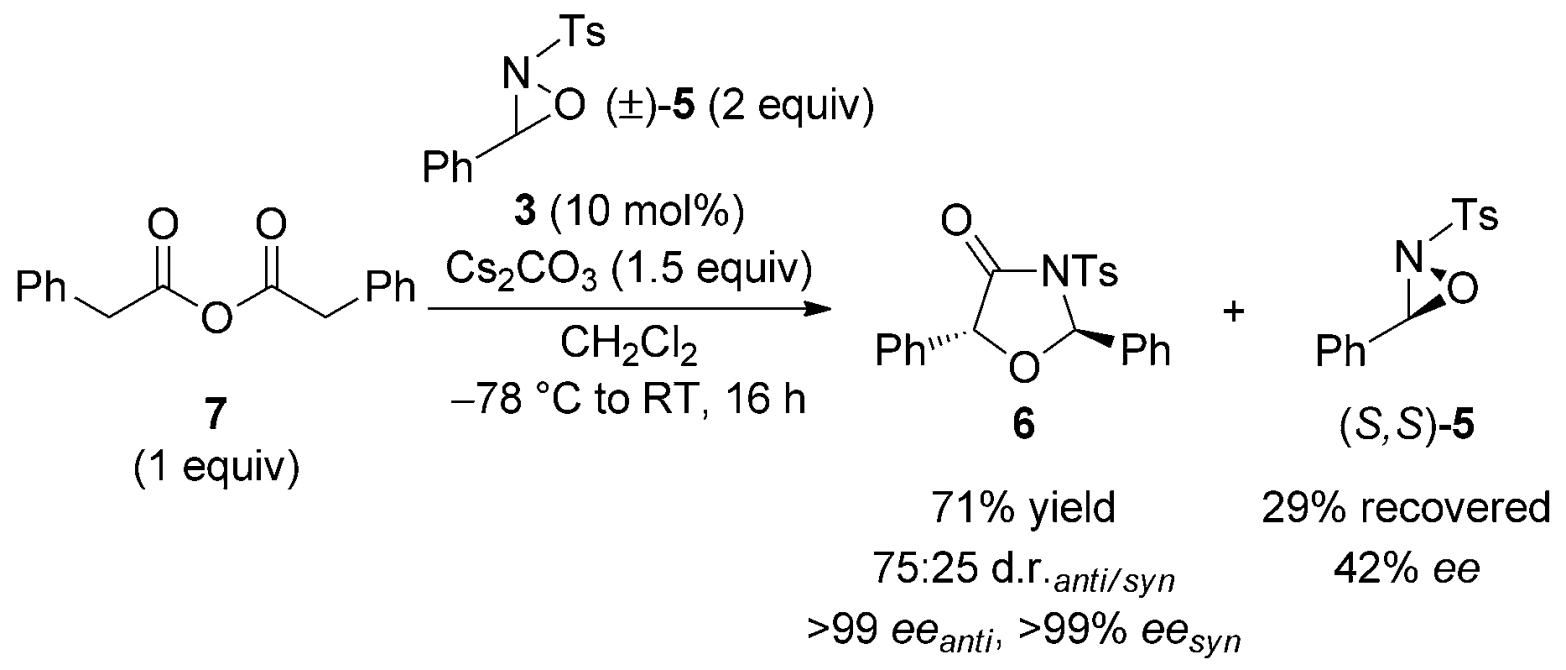
Use of excess (±)-oxaziridine 5.

To further investigate and utilise the chirality transfer in this process the use of an excess of (±)-oxaziridine **5** (2 equiv with respect to homoanhydride **7**) was trialled (Scheme 3).[24] In this case, oxazolidin-4-one **6** was isolated in 71 % yield with an improved 75:25 d.r., with both diastereoisomers again formed with excellent enantioselectivity. The remaining oxaziridine **5** was isolated in 42 % *ee*, with the (*S*,*S*)-enantiomer in excess. This formally represents a kinetic resolution of (±)-**5** with 49 % conversion with respect to the oxaziridine (as judged by crude ^1^H NMR spectroscopic analysis) equating to a selectivity factor S=4.[25]

**Scheme 4:**
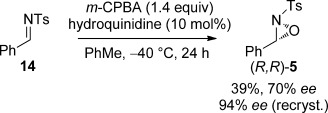
Synthesis of enantioenriched oxaziridine (*R*,*R*)-5. *m*-CPBA=*meta*-chloroperbenzoic acid.

In light of these results, it was reasoned that using an enantiomerically pure oxaziridine would lead to the formation of a single diastereoisomer of the corresponding oxazolidin-4-one product through complete chirality transfer. To assess this, enantioenriched oxaziridine (*R*,*R*)-**5** was accessed in 94 % *ee* (following a single recrystallisation) using a modified procedure developed by Jørgensen and co-workers (Scheme 4).[26]

**Scheme 5:**
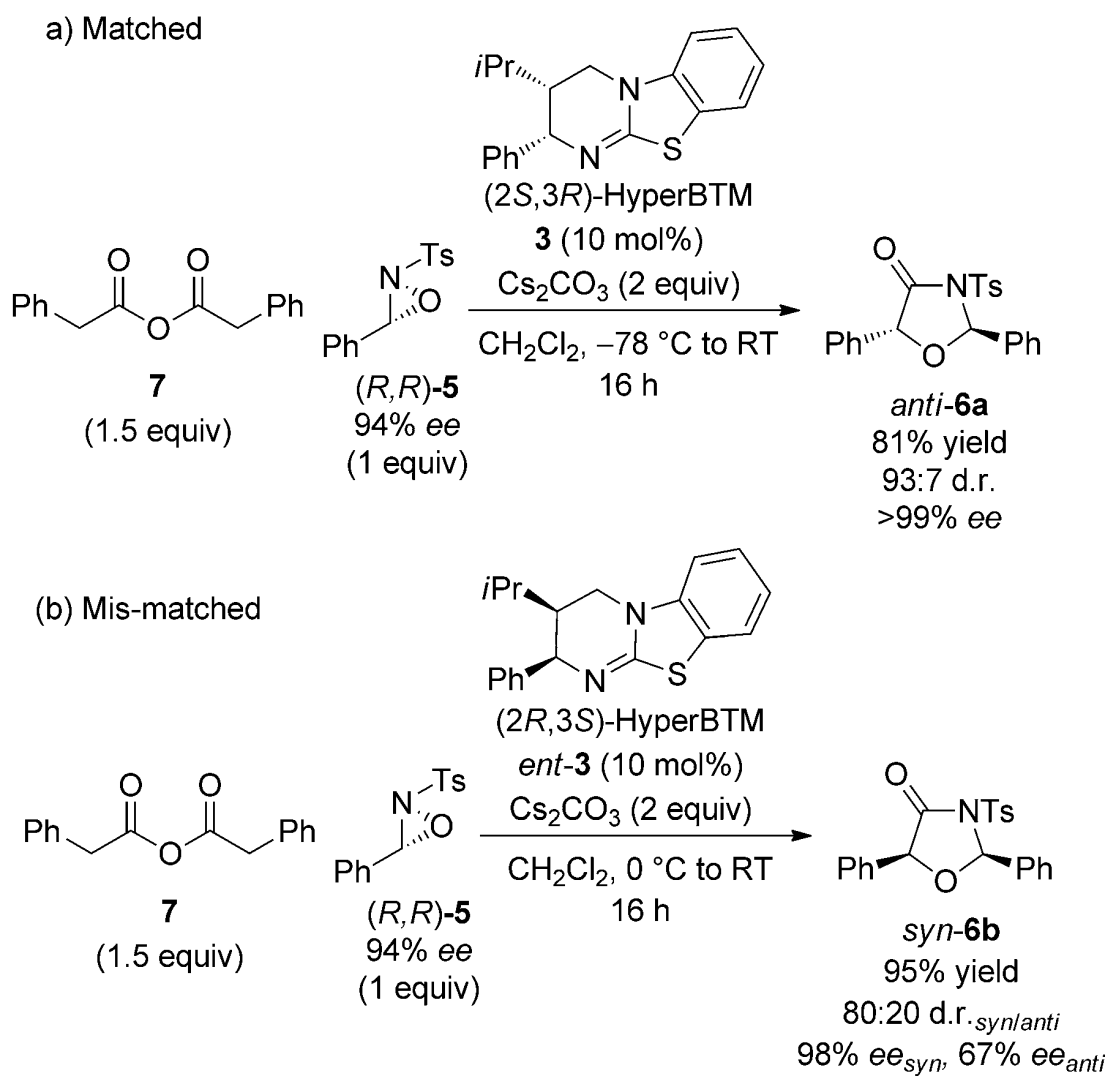
Investigation of a) matched and b) mis-matched effects between ammonium enolate and enantiomerically enriched oxaziridine.

Pleasingly, using enantioenriched oxaziridine (*R*,*R*)-**5** with phenylacetic anhydride **7** and (2*S*,3*R*)-HyperBTM **3** (Scheme 5a) gave *anti*-oxazolidin-4-one **6 a** in high yield, *ee* and excellent d.r. (93:7, *anti*/*syn*). This matched case arises from the ammonium enolate generated with homoanhydride **7** and (2*S*,3*R*)-HyperBTM **3** reacting with (*R*,*R*)-**5** with excellent stereocontrol. Using enantiomeric catalyst (2*R*,3*S*)-HyperBTM *ent*-**3**, low reactivity and reduced isolated yields were observed at −78 °C. However, performing the reaction at 0 °C allowed the desired *syn*-oxazolidin-4-one **6 b** to be isolated in 95 % yield and 80:20 d.r. (*syn*/*anti*), with the major *syn* product formed in excellent *ee* (98 %) (Scheme 5 b). This again suggests complete chirality transfer from the oxaziridine with the configuration at C(5) determined by the catalyst. In the mis-matched case the minor *anti*-oxazolidin-4-one product was isolated in reduced *ee* (67 %), presumably as a result of a competitive uncatalysed background reaction for this catalytically unfavoured process.

**Scheme 6:**
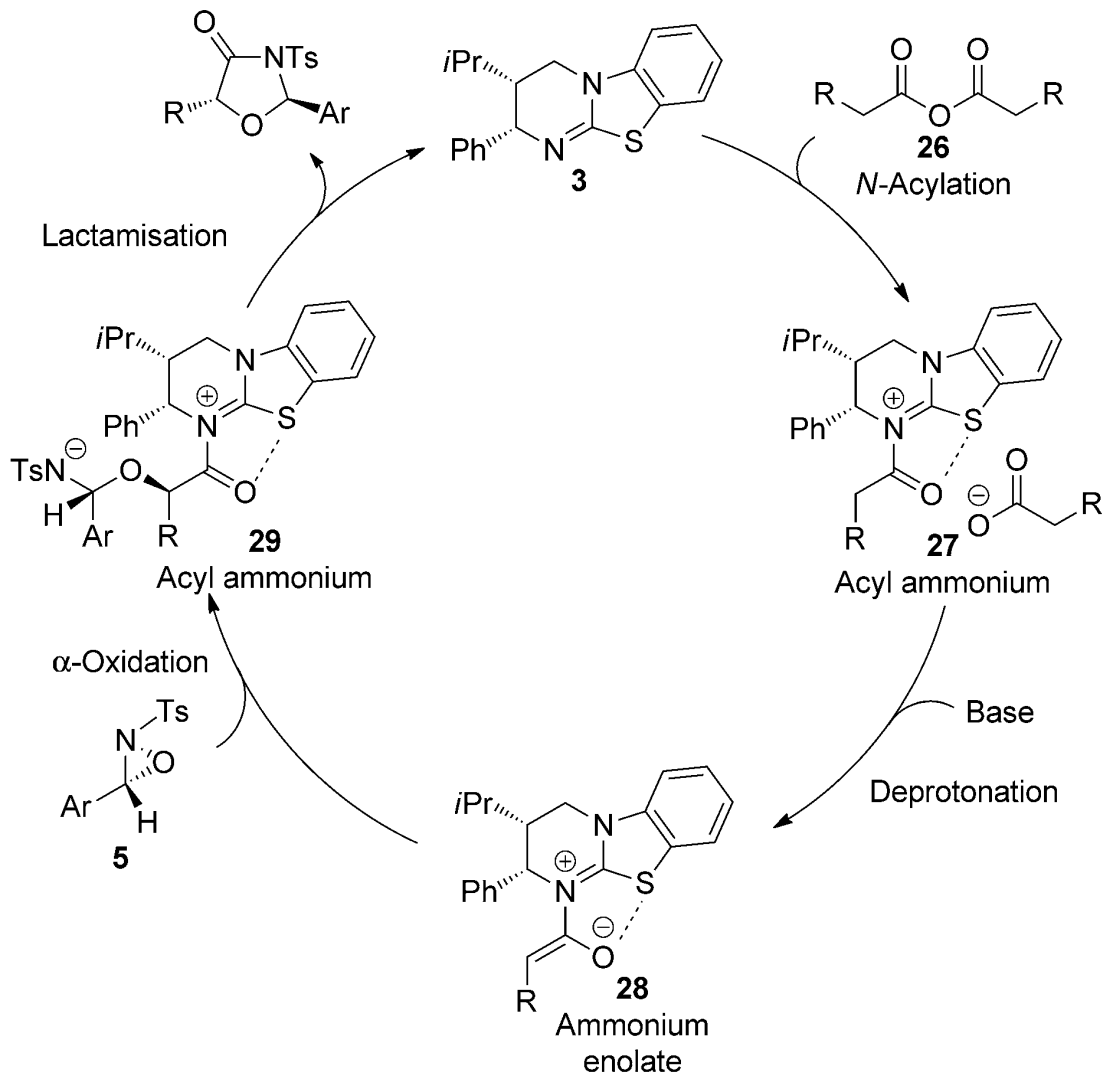
Proposed mechanism for Lewis base catalysed formal [3+2] cycloaddition of ammonium enolates with oxaziridines.

The results described in Scheme 5 lead us to propose a catalytic cycle for the synthesis of oxazolidin-4-ones, shown in Scheme 6. Firstly, homoanhydride **26** acylates HyperBTM **3** to give acyl ammonium **27**. Subsequent deprotonation of **27** to give (*Z*)-ammonium enolate **28**, stabilised by a favourable *n*_o_ to σ*_C–S_ interaction,[8h, 9d, 27] followed by intermolecular stereoselective α-oxidation[28] leads to acyl ammonium **29**. Finally, lactamisation gives the oxazolidin-4-one product and regenerates the catalyst. This mechanism provides an alternative to that proposed by Ye and co-workers who suggest that for their related NHC-catalysed formal [3+2] process the azolium enolate generated is oxidised by an oxaziridine to form a transient epoxide species and an imine, with subsequent collapse of the epoxide and nucleophilic attack onto the imine generating an acyl azolium species that can cyclise into an oxazolidin-4-one. Our observation of a matched/mis-matched effect using enantioenriched oxaziridine suggests the formation of a transient planar imine intermediate in this process is unlikely. However, the possibility of an alternative mechanistic pathway operating in the mis-matched case cannot be ruled out.

The significance of the matched/mis-matched effect was further demonstrated through reaction of a range of homoanhydrides with (*R*,*R*)-oxaziridine **5** (94 % *ee*) using HyperBTM **3** (Table 4). Under the previously optimised conditions, electron-donating and -withdrawing aromatic substituents were easily incorporated resulting in high yields, enantioselectivities and, importantly, high d.r. of oxazolidin-4-ones **17 a** and **18 a**, respectively. Substitution in either the *ortho*- or *meta*-positions of the aryl ring was also well tolerated, forming oxazolidin-4-ones **19 a**, **21 a** and **22 a** as single diastereoisomers with excellent levels of enantioselectivity. Alkenyl and heteroaryl homoanhydride substituents were also successfully incorporated to give **30 a** and **31 a** respectively, with high levels of stereocontrol. However, the introduction of a *p*-trifluoromethyl substituent gave oxazolidin-4-one **25** in a reduced 60:40 d.r._*anti/syn*_, with both diastereoisomers formed in high enantioselectivity (>99 % *ee*). This suggests that major product *anti*-**25 a** is formed with high levels of enantioselectivity but undergoes base-mediated epimerisation at C(5) into *syn*-**25 b**. This result also provides a plausible explanation for the unexpected result using the *p*-trifluoromethyl-substituted homoanhydride with (±)-oxaziridine **5**, with epimerisation at C(5) in combination with the expected mixture at C(2) leading to the observed drop in *ee* of both diastereoisomers of **25** (Table 2).

**Table 4:** Investigation of the substrate scope with enantioenriched oxaziridine 5

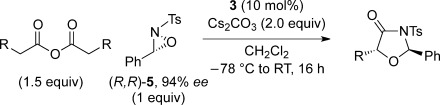			
Major Product	Yield [%]^[a]^ d.r._*anti*/*syn*_^[b]^ *ee_anti_* [%]^[c]^	Major product	Yield [%]^[a]^ d.r._*anti*/*syn*_^[b]^ *ee_anti_* [%]^[c]^
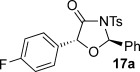	81 94:6 99	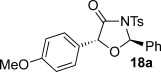	87 95:5 97
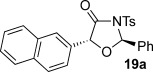	60 95:5 >99	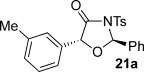	87 >95:5 >99
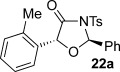	95 >95:5 >99	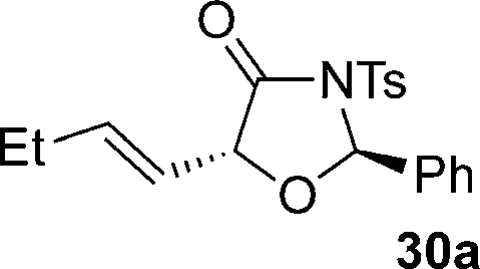	71 96:4 >99
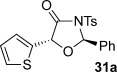	61 94:6 >99	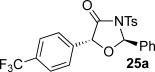	49 60:40 >99:99

[a] Isolated yield. [b] Determined by ^1^H NMR spectroscopic analysis of the crude reaction product. [c] Determined by HPLC analysis.

**Scheme 7:**
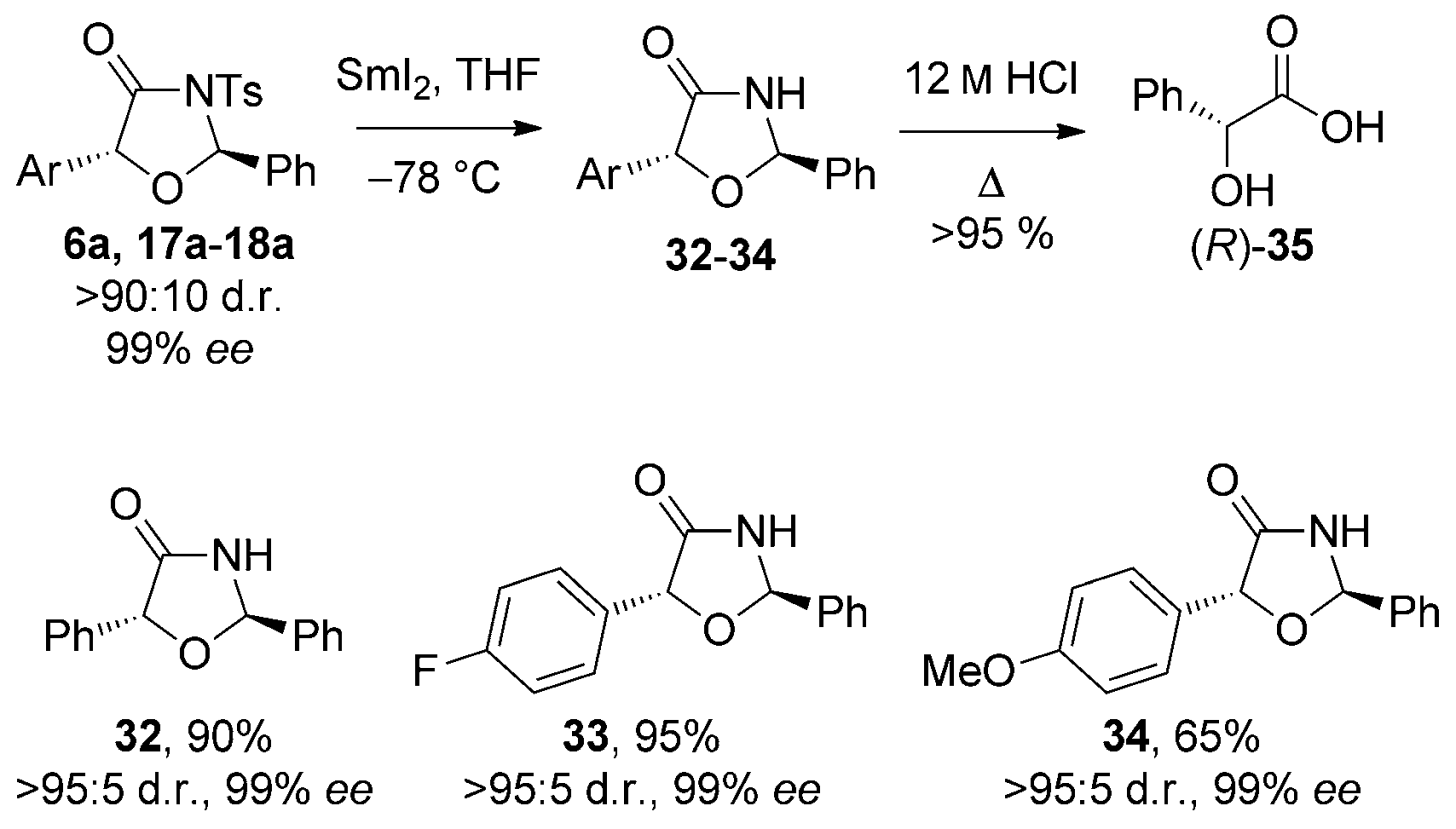
Deprotection of oxazolidin-4-one products with SmI_2_ and subsequent hydrolysis.

To demonstrate the synthetic utility of this [3+2] process, additional product derivatisations have been investigated (Scheme 7). Removal of the *N*-tosyl protecting group on oxazolidin-4-ones **6 a** and **17 a–18 a** was achieved with SmI_2_ at low temperature to give the parent heterocycles **32**–**34** in high yields, with complete retention of *ee*. Further hydrolysis of oxazolidin-4-one **32** with HCl led to formation of (*R*)-mandelic acid **35** in quantitative yield.

## Conclusion

The asymmetric formal [3+2] cycloaddition of ammonium enolates with both (±)-oxaziridines and (*R*,*R*)-oxaziridines has been developed using a range of 2-aryl and 2-alkenylacetic anhydrides with the commercially available isothiourea catalyst HyperBTM **3**. This process allows access to stereodefined oxazolidin-4-ones that can be readily deprotected or reduced to give enantioenriched building blocks in high yield. Further studies using enantioenriched oxaziridines led to the observation of a matched/mis-matched effect with isothiourea HyperBTM **3**, which has been utilised to obtain oxazolidin-4-ones in high d.r. with excellent *ee*. Ongoing studies within this laboratory are focused upon the continued development of Lewis base catalysis.

## Experimental Section

### General

For general experimental details, full characterisation data, NMR spectra, and HPLC traces, see the Supporting Information.

### General procedure for the asymmetric organocatalytic formation of oxazolidin-4-ones

The appropriate oxaziridine (1 equiv) and (2*S*,3*R*)-HyperBTM **3** (10 mol %) were added to a solution of the appropriate homoanhydride (1.5 equiv) and cesium carbonate (2 equiv) in CH_2_Cl_2_ (0.2 m) at −78 °C. The reaction mixture was stirred at −78 °C then warmed slowly to room temperature over 16 h before being quenched with HCl (1.0 m). The reaction mixture was extracted with CH_2_Cl_2_ (×2), the combined organics dried over MgSO_4_, filtered and concentrated in vacuo. The crude residue was purified by column chromatography on silica gel (eluent petrol/Et_2_O 80:20 unless otherwise stated) to afford the desired oxazolidin-4-one.

## Supporting Information

As a service to our authors and readers, this journal provides supporting information supplied by the authors. Such materials are peer reviewed and may be re-organized for online delivery, but are not copy-edited or typeset. Technical support issues arising from supporting information (other than missing files) should be addressed to the authors.

miscellaneous_information
