# Supplementary material for: Asymmetric Isothiourea-Catalysed Formal [3+2] Cycloadditions of Ammonium Enolates with Oxaziridines
Source: Chemistry. 2015 Jun 12;21(29):10530–6. doi: 10.1002/chem.201501271 (PMC4531819; doi:10.1002/chem.201501271)

CHEMISTRY
A European Journal

Supporting Information

Asymmetric Isothiourea-Catalysed Formal [3+2] Cycloadditions of Ammonium Enolates with Oxaziridines

Siobhan R. Smith, Charlene Fallan, James E. Taylor, Ross McLennan, David S. B. Daniels, Louis C. Morrill, Alexandra M. Z. Slawin, and Andrew D. Smith*[a]

chem_201501271_sm_miscellaneous_information.pdf

Asymmetric Isothiourea-Catalysed Formal [3+2] Cycloadditions of Ammonium Enolates with Oxaziridines

*Siobhan R. Smith, Charlene Fallan, James E. Taylor, Ross McLennan, David S. B. Daniels Louis C. Morrill, Alexandra M. Z. Slawin, Andrew D. Smith**

EaStCHEM, School of Chemistry, University of St Andrews, North Haugh, St Andrews, KY16 9ST, U.K.

ads10@st-andrews.ac.uk

General Experimental S2

General Procedures S4

Starting Material Synthesis S6

Homoanhydrides S6

Oxaziridines S10

Synthesis of Oxazolidin-4-ones S13

Using (±)-oxaziridines S13

Using excess (±)-oxaziridine ***5*** S25

Using (R,R)-oxaziridine ***5*** S26

Product Derivatisations S33

References S36

^{1}H and ^{13}C{^{1}H} NMR Spectra of New Compounds S37

HPLC Traces S92

General Experimental

Reactions were performed in flame-dried glassware under an Ar or N_2 atmosphere unless otherwise stated. Anhydrous CH_2Cl_2, Et_2O, THF and toluene were obtained from an MBraun SPS-800 system. Petrol is defined as petroleum ether 40–60 °C. All other solvents and commercial reagents were used as received without further purification unless otherwise stated. Room temperature (rt) refers to 20–25 °C. Temperatures of 0 °C and –78 °C were obtained using ice/water and CO_2(s)/acetone baths respectively.

Analytical thin layer chromatography was performed on pre-coated aluminium plates (Kieselgel 60 F_{254} silica). Plates were visualised under UV light (254 nm) or by staining with either phosphomolybdic acid or $KMnO_4$ followed by heating. Flash column chromatography was performed on Kieselgel 60 silica in the solvent system stated under a positive pressure of compressed air or on a Biotage® Isolera™ 4, using Biotage® Snap Ultra or Biotage® KP Sil columns under the solvent system stated.

Melting points were recorded on an Electrothermal 9100 melting point apparatus. Optical rotations were measured on a Perkin Elmer Precisely/Model-341 polarimeter operating at the sodium D line with a 100 mm path cell at 20 °C.

HPLC analyses were obtained on a Shimadzu HPLC consisting of a DGU-20A5 degasser, LC-20AT liquid chromatography SIL-20AHT autosampler, CMB-20A communications bus module, SPD-M20A diode array detector and a CTO-20A column oven that allows the temperature to be set from 25–40 °C. Separation was achieved using Chiralcel OD-H or Chiralpak AD-H columns.

Infrared spectra (ν_{max}) were recorded on a Shimadzu IRAffinity-1 Fourier transform IR spectrophotometer using either thin film or solid using Pike MIRacle ATR accessory. Analysis was carried out using Shimadzu IRsolution v1.50 and only characteristic peaks are reported.

^{1}H, ^{13}C{^{1}H}, and ^{19}F{^{1}H} NMR spectra were recorded on Bruker Avance 500 MHz, Bruker Avance 400 MHz and Bruker Avance 300 MHz NMR spectrometers. In $CDCl_3$, ^{1}H and ^{13}C{^{1}H} NMR chemical shifts are reported relative to $CHCl_3$ at 7.27 ppm and 77.0 ppm, respectively. Coupling constants (*J*) are reported in Hertz (Hz). Multiplicities are indicated by: br s (broad singlet), s (singlet), d (doublet), t (triplet), q (quartet) and m (multiplet).

Mass spectrometry (*m/z*) data were acquired by electrospray ionisation (ES) or nanospray ionisation (NSI) at the EPSRC UK National Mass Spectrometry Facility at Swansea University.

N-Sulfonyl imines **A**,[1] **B**,[1] **C**[2] and **D**[3] were prepared according to literature procedures and data matched that reported.

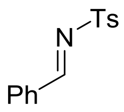

A

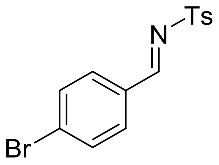

B

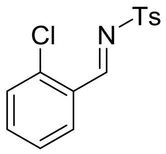

C

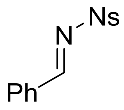

D

General Procedures

General Procedure 1: *Synthesis of Anhydrides*

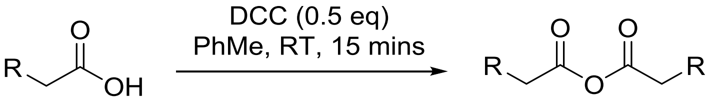

To a solution of the appropriate carboxylic acid (1 eq) in toluene (0.3 M), DCC (0.50-0.55 eq) was added and the solution stirred at room temperature for 15 min. The suspension was filtered and concentrated *in vacuo* to give the crude product, which was used without further purification.

General Procedure 2: *Synthesis of N-Sulfonyl Oxaziridine Starting Materials*

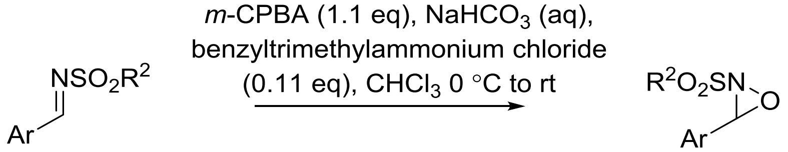

To a solution of saturated aqueous sodium bicarbonate (1 M) and benzyltrimethylammonium chloride (0.11 eq), the appropriate *N*-sulfonyl imine (1 eq) was added as a solution in $CHCl_3$ (1 M). The mixture was cooled to 0 °C and a solution of *m*-CPBA (1.1 eq) in $CHCl_3$ (0.5 M) was added dropwise at 0 °C and stirred for 1 h. The organic layer was separated, washed with water, 10% sodium sulphite solution, water and brine before drying ($MgSO_4$) and concentrating *in vacuo,* keeping the bath temperature below 40 °C. The crude oxaziridine was recrystallised from ethyl acetate/petrol without heating.

General Procedure 3: *Asymmetric Organocatalytic Formation of Oxazolidin-4-ones*

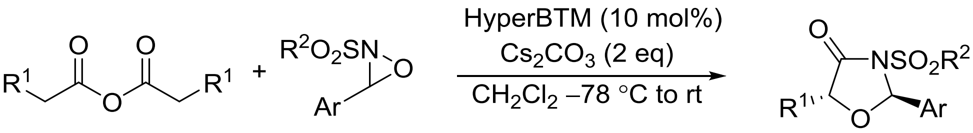

The appropriate oxaziridine (1 eq) and HyperBTM **3** (10 mol%) were added to a solution of the appropriate homoanhydride (1.5 eq) and Cs_2CO_3 (2 eq) in CH_2Cl_2 (0.2 M) at −78 °C. The reaction mixture was stirred at −78 °C allowing to warm to room temperature over 16 h before being quenched with 1 M HCl. The reaction mixture was extracted with CH_2Cl_2 (×2), the combined organics were dried ($MgSO_4$), filtered and concentrated *in vacuo*. The crude residue was purified *via* column chromatography on silica gel (petrol:Et_2O, 80:20 unless otherwise stated).

Authentic racemic samples were prepared in an analogous fashion using (*rac*)-HyperBTM.

General Procedure 4: *N-Ts Deprotection*

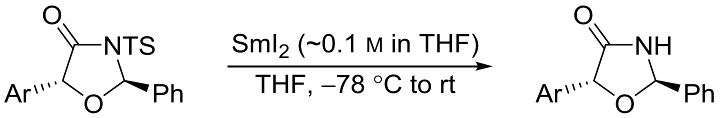

The appropriate oxazolidin-4-one (1 eq) was added to a flame-dried round-bottomed flask and placed under an Ar atmosphere. Degassed (Ar sparge, 30 min) anhydrous THF (0.1 M) was added and the solution cooled to –78 °C before SmI_2 (~0.1 M in THF, 2 eq) was added dropwise to give a deep-blue solution. The reaction was warmed to room temperature and monitored by TLC, with the solution turning yellow to indicate consumption of SmI_2. Upon completion the reaction was diluted with EtOAc and washed with $NaHCO_3$ (×3) and brine (×3) before being dried over $MgSO_4$, filtered and concentrated *in vacuo*. The crude product was purified by flash silica column chromatography using a Biotage® Isolera™ 4.

Authentic racemic samples were prepared in an analogous fashion using (*rac*)-oxazolidin-4-one.

Starting Material Synthesis

Homoanhydrides

2-Phenylacetic anhydride 7

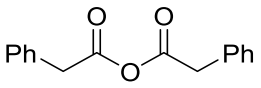

7

Following *General Procedure 1*, phenylacetic acid (0.45 g, 3.33 mmol) and DCC (0.35 g, 1.70 mmol) were stirred in toluene (10 mL) to give product **7** as a white solid (0.43 g, >99%) with data in accordance to the literature.[4] mp 68-70 °C {lit.[4] mp 72-72.5 °C}; ^{1}H NMR (400 MHz, $CDCl_3$) δ_H: 3.76 (4H, s, 2×C*H*$_2$), 7.23-7.25 (4H, m, Ar*H*), 7.32-7.38 (6H, m, Ar*H*).

2-(4-Fluorophenyl)acetic anhydride S1

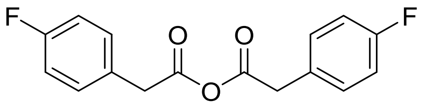

S1

Following *General Procedure 1*, 4-fluorophenylacetic acid (1.00 g, 6.49 mmol) and DCC (0.74 g, 3.57 mmol) were stirred in toluene (20 mL) to give product **S1** as a white solid (0.94 g, >99%) with data in accordance with the literature.[5] mp 46-48 °C {Lit.[5] 36-38 °C}; ^{1}H NMR (400 MHz, $CDCl_3$) δ_H: 3.70 (4H, s, 2×C*H*$_2$), 6.94-7.08 (4H, m, Ar*H*), 7.11-7.22 (4H, m, Ar*H*); ^{19}F NMR (376 MHz, $CDCl_3$) δ_F: −115.1 (Ar*F*).

2-(4-Methoxyhenyl)acetic anhydride S2

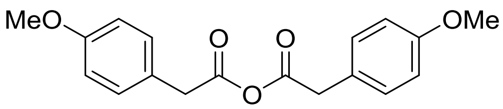

S2

Following *General Procedure 1*, 4-methoxyphenylacetic acid (1.00 g, 6.00 mmol) and DCC (0.62 g, 3.00 mmol) were stirred in toluene (25 mL) to give product **S2** as a white solid (0.94 g, >99%) with data in accordance with the literature. [5] mp 46-48 °C {Lit.[5] 60-62 °C}; ^{1}H NMR (300 MHz, $CDCl_3$) δ_H: 3.66 (4H, s, 2×C*H*$_2$), 3.80 (6H, s, 2×C*H*$_3$), 6.83-6.86 (4H, m, Ar*H*), 7.10-7.13 (4H, m, Ar*H*).

2-(Naphthalen-2-yl)acetic anhydride S3

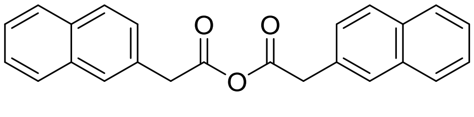

S3

Following *General Procedure 1*, 2-naphthylacetic acid (1.00 g, 5.40 mmol) and DCC (0.56 g, 2.70 mmol) were stirred in toluene (25 mL) to give product **S3** as a white solid (0.41 g, 43%) with data in accordance with the literature.[5] mp 100-104 °C {Lit.[5] 104-108 °C}; ^{1}H NMR (300 MHz, $CDCl_3$) δ_H: 3.88 (4H, s, 2×C*H*$_2$), 7.27 (2H, d, *J* 7.4, Ar*H*), 7.47-7.51 (4H, m, Ar*H*), 7.63 (2H, s, 2×Ar*H*), 7.70-7.73 (4H, m, Ar*H*), 7.80-7.83 (2H, m, Ar*H*).

2-(4-Tolyl)acetic anhydride S4

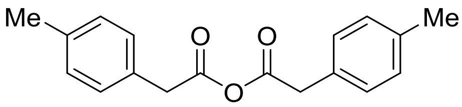

S4

Following *General Procedure 1*, *p*-tolylacetic acid (0.50 g, 3.33 mmol) and DCC (0.35 g, 1.70 mmol) were stirred in toluene (10 mL) to give product **S4** as a white solid (0.47 g, >99%) with data in accordance with the literature.[6] mp 46-48 °C {Lit.[6] 56-57 °C}; ^{1}H NMR (400 MHz, $CDCl_3$) δ_H: 2.39 (6H, s, 2×C*H*$_3$), 3.72 (4H, s, 2×C*H*$_2$), 7.12-7.19 (8H, m, Ar*H*).

2-(3-Tolyl)acetic anhydride S5

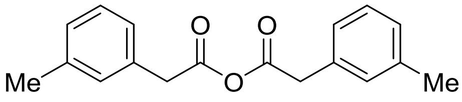

S5

Following *General Procedure 1*, *m*-tolylacetic acid (0.50 g, 3.33 mmol) and DCC (0.35 g, 1.70 mmol) were stirred in toluene (10 mL) to give product **S5** as a yellow oil (0.47 g, >99%) with data in accordance with the literature.[6] ^{1}H NMR (400 MHz, $CDCl_3$) δ_H: 2.27 (6H, s, 2×C*H*$_3$), 3.54 (4H, s, 2×C*H*$_2$), 6.99-7.02 (3H, m, Ar*H*), 7.12-7.18 (5H, m, Ar*H*).

2-(2-Tolyl)acetic anhydride S6

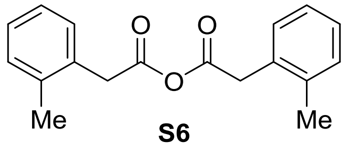

S6

Following *General Procedure 1*, *o*-tolylacetic acid (0.50 g, 3.33 mmol) and DCC (0.35 g, 1.70 mmol) were stirred in toluene (10 mL) to give product **S6** as a sticky oil (0.47 g, >99%) with data in accordance with the literature.[6] ^{1}H NMR (500 MHz, $CDCl_3$) δ_H: 2.24 (6H, s, 2×CH_3), 3.72 (4H, s, 2×CH_2), 7.07-7.11 (2H, m, Ar*H*), 7.11-7.25 (6H, m, Ar*H*); ^{13}C{^{1}H} NMR (125 MHz, $CDCl_3$) δ_C: 19.6 (*C*H_3), 40.2 (*C*H_2), 126.4 (Ar*C*), 128.1 (Ar*C*), 130.5 (Ar*C*), 130.7 (Ar*C*), 130.9 (Ar*C*(1)), 137.1 Ar*C*(2)CH_3), 167.0 (*C*=O).

2-(Thiophen-3-yl)acetic anhydride S7

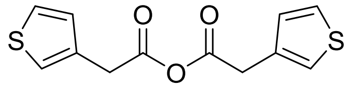

S7

Following *General Procedure 1*, 3-thiopheneacetic acid (0.47 g, 3.33 mmol) and DCC (0.35 g, 1.70 mmol) were stirred in toluene (10 mL) to give product **S7** as a yellow solid (0.44 g, >99%) with data in accordance with the literature.[5] mp 36-38 °C {Lit. [5] 40-42 °C}; ^{1}H NMR (400 MHz, $CDCl_3$) δ_H: 3.79 (4H, s, 2×CH_2), 6.99 (2H, dd, *J* 5.0, 1.2, Ar*H*), 7.15 (2H, dd, *J* 2.0, 1.0, Ar*H*), 7.31 (2H, dd, *J* 5.0, 3.0, Ar*H*).

(*E*)-Pent-3-enoic anhydride S8

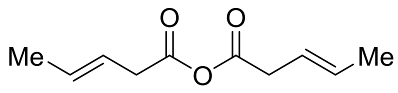

S8

Following *General Procedure 1*, 3-pentenoic acid (1.36 mL, 13.3 mmol) and DCC (1.40 g, 6.80 mmol) were stirred in toluene (40 mL) to give product **S8** as a pale yellow oil (1.20 g, >99%). ν_{max} (neat) 3000 (=CH), 2920 (C-H), 1819 (C=O), 1749 (C=O), 1699 (C=C), 1031 (C-O); ^{1}H NMR (400 MHz, $CDCl_3$) δ_H: 1.69-1.73 (6H, m, 2×CH_3), 3.11-3.20 (4H, m, 2×CH_2), 5.42-5.57 (2H, m, 2×C*H*), 5.57-5.70 (2H, m, 2×C*H*); ^{13}C{^{1}H} NMR (100 MHz, $CDCl_3$) δ_C: 18.1 (*C*H_3), 39.0 (*C*H_2), 120.9 (*C*H), 131.2 (*C*H), 167.9 (*C*=O); *m/z* (ASAP$^+$) 200 ([M+NH_4]$^+$, 75%); HRMS (ASAP$^+$) $C_{10}H_{18}O_3N$ [M+NH_4]$^+$ found 200.1277, requires 200.1281 (–2.1 ppm).

2-(4-(Trifluoromethyl)phenyl)acetic anhydride S9

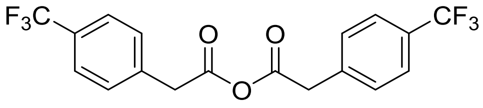

S9

Following *General Procedure 1*, *p*-trifluoromethylphenyl acid (0.68 g, 3.33 mmol) and DCC (0.35 g, 1.70 mmol) were stirred in toluene (10 mL) to give product **S9** as an off white solid (0.65 g, >99%). mp 58-60 °C; ν_{max} (neat) 2933 (C-H), 1813 (C=O), 1749 (C=O), 1062 (C-O), 766 (CF_3); ^{1}H NMR (500 MHz, $CDCl_3$) δ_H: 3.80 (4H, s, 2×C*H*$_2$), 7.33 (4H, d, *J* 8.1, Ar*H*), 7.59 (4H, d, *J* 8.1, Ar*H*); ^{13}C{^{1}H} NMR (125 MHz, $CDCl_3$) δ_C: 41.8 (*C*H$_2$), 124.1 (q, $^1J_{CF}$ 272.1, *C*F$_3$), 125.9 (q, $^3J_{CF}$ 4.1, Ar*C*(3)), 129.9 (Ar*C*(2)), 130.3 (q, $^2J_{CF}$ 32.8, Ar*C*(4)), 135.8 (Ar*C*(1)), 166.0 (*C*=O); ^{19}F NMR (376 MHz, $CDCl_3$) δ_F: −115.1 (Ar*F*); *m/z* HRMS $C_{18}H_{13}F_6O_3$ $[M+H]^+$ found 391.0759, requires 391.0763 (−1.1 ppm).

(*E*)-Hex-3-enoic anhydride S10

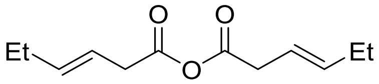

S10

Following *General Procedure 1*, (E)-hex-3-enoic acid (1.18 mL, 10.0 mmol) and DCC (1.13 g, 5.5 mmol) were stirred in toluene (50 mL) to give product **S10** as a colourless oil (2.40 g, >99%). Used crude, selected data: ν_{max} (neat) 2962 (C-H), 1816 (C=O), 1749 (C=O), 1697 (C=C), 1033 (C-O); ^{1}H NMR (400 MHz, $CDCl_3$) δ_H: 0.96 (6H, t, *J* 7.5, 2×C*H*$_3$) 2.03 (4H ddd, *J* 7.6, 6.2, 1.3, 2×C*H*$_2$CH$_3$), 3.14 (4H, dt, *J* 6.8, 1.1, 2×C*H*$_2$CH=CH), 5.31-5.55 (2H, m, 2×CH=C*H*Et), 5.53-5.82 (2H, m, 2×C*H*=CHEt),^{13}C{^{1}H} NMR (100 MHz, $CDCl_3$) δ_C: 13.1 (*C*H$_3$), 25.3 (CH$_3$*C*H$_2$), 38.6 (CH=CH*C*H$_2$), 118.5 (*C*(3)H), 137.7 (*C*(4)H), 167.6 (*C*=O).

2-(Thiophen-2-yl)acetic anhydride S11

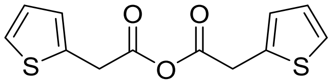

S11

Following *General Procedure 1*, 2-(thiophen-2-yl)acetic acid (1.0 g, 7.0 mmol) and DCC (0.8 g, 3.8 mmol) were stirred in toluene (20 mL) to give product **S11** as a pale yellow oil (0.93 g, >99%). Used crude, selected data: ν_{max} (neat) 3101 (=CH), 2927 (C-H), 1820 (C=O), 1749 (C=O), 1691 (C=C), 1031 (C-O);^{1}H NMR (400 MHz, $CDCl_3$) δ_H: 3.99 (4H, s, 2×C*H*$_2$), 6.98-7.02 (4H, m, 4×Ar*H*), 7.28

(2H, dd, *J* 5.1, 1.3, 2×Ar*H*); $^{13}C\{^1H\}$ NMR (100 MHz, $CDCl_3$) δ_C: 35.9 (2×CH_2), 125.5 (2×Ar*C*H), 126.9 (2×Ar*C*H), 127.6 (2×Ar*C*H), 132.4, (2×Ar*C*), 165.4 (2×*C*=O).

Oxaziridines

3-Phenyl-2-tosyl-1,2-oxaziridine (±)-5

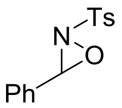

(±)-5

Following *General Procedure 2*, benzyltrimethyammonium chloride (0.28 g, 1.49 mmol), (*E*)-*N*-(benzylidene)-4-methylbenzenesulfonamide **14** (3.50 g, 13.5 mmol) and *m*-CPBA (3.34 g, 14.9 mmol, <77%) were stirred at 0 °C for 1 h. The resulting yellow solid was recrystallised to give **(±)-5** as a white solid (3.82 g, >99%) with data in accordance with the literature.[7] mp 92-94 °C {Lit.[7] 85-87 °C}; ^1H NMR (400 MHz, $CDCl_3$) δ_H: 2.49 (3H, s, CH_3), 5.45 (1H, s, C*H*), 7.31-7.55 (7H, m, Ar*H*), 7.87-7.97 (2H, m, Ar*H*).

3-(2-Chlorophenyl)-2-tosyl-1,2-oxaziridine (±)-8

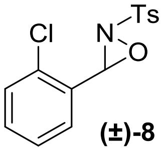

(±)-8

Following *General Procedure 2*, benzyltrimethyammonium chloride (0.23 g, 1.25 mmol), (*E*)-*N*-(2-chlorobenzylidene)-4-methylbenzenesulfonamide (3.34 g, 11.4 mmol) and *m*-CPBA (2.81 g, 12.5 mmol, <77%) were stirred at 0 °C for 1 h. The resulting yellow solid was recrystallised to give **(±)-8** as a white solid (2.97 g, 84%) with data in accordance with the literature.[8] mp 92-94 °C {Lit.[8] 105-107 °C}; ^1H NMR (400 MHz, $CDCl_3$) δ_H: 2.50 (3H, s, CH_3), 5.86 (1H, s, C*H*), 7.26-7.29 (2H, m, Ar*H*), 7.37-7.45 (4H, m, Ar*H*), 7.94 (2H, d, *J* 8.4, Ar*H*).

3-(4-Bromophenyl)-2-tosyl-1,2-oxaziridine (±)-9

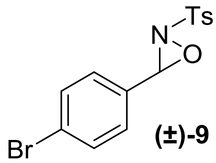

(±)-9

Following *General Procedure 2*, benzyltrimethyammonium chloride (0.22 g, 1.18 mmol), (*E*)-*N*-(4-bromobenzylidene)-4-methylbenzenesulfonamide (3.63 g, 10.7 mmol) and *m*-CPBA (2.64 g, 11.8 mmol, <77%) were stirred at 0 °C for 1 h. The resulting white solid was recrystallised to give **(±)-9** as a white solid (2.03 g, 54%) with data in accordance with the literature.[7] mp 98-100 °C {Lit.[7] 92-93 °C}; ^{1}H NMR (400 MHz, $CDCl_3$) δ_H: 2.50 (3H, s, C*H*$_3$), 5.42 (1H, s, C*H*), 7.28-7.34 (2H, m, Ar*H*), 7.43 (2H, d, *J* 8.1, Ar*H*), 7.54 (2H, d, *J* 8.5, Ar*H*), 7.92 (2H, d, *J* 8.3, Ar*H*).

2-(4-Nitrobenzenesulfonyl)-3-phenyloxaziridine (±)-10

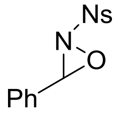

(±)-10

Following *General Procedure 2*, benzyltrimethyammonium chloride (0.08 g, 0.41 mmol), 4-nitro-*N*-[(1*E*)-phenylmethylidene]benzene-1-sulfonamide (1.08 g, 3.72 mmol) and *m*-CPBA (0.92 g, 4.09 mmol, <77%) were stirred at 0 °C for 1 h. The resulting yellow solid was recrystallised to give **(±)-10** as a white solid (0.66 g, 58%) with data in accordance with the literature.[9] mp 84-86 °C {Lit.[9] 96 °C}; ^{1}H NMR (400 MHz, $CDCl_3$) δ_H: 5.60 (1H, s, C*H*), 7.39-7.48 (4H, m, Ar*H*), 7.47-7.54 (1H, m, Ar*H*), 8.27 (2H, d, *J* 8.8, SO_2Ar*H*), 8.48 (2H, d, *J* 8.8, SO_2Ar*H*).

(*R*,*R*)-3-Phenyl-2-tosyl-1,2-oxaziridine (*R*,*R*)-5

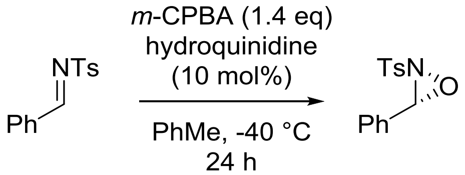

Imine **14** (1.33 g, 5.1 mmol) and hydroquinidine catalyst (167 mg, 0.51 mmol, 0.1 eq) in toluene (50 mL) was cooled to –40 °C, before addition of *m*-CPBA (77%, 1.6 g, 7.2 mmol), and the resulting solution stirred at –40 °C for 24 h. The toluene was removed under reduced pressure and the residue purified by flash chromatography (eluent: toluene) to give **(*R*,*R*)-5** as a white solid that was further purified *via* recrystallisation from EtOAc/hexanes to afford white crystals with data consistent with the literature[10] (0.53 g, 39%). mp 86-89 °C {lit.[10] 90.4-91.7 °C}; $[\alpha]_D^{22}$ +94.1 (*c* 1.0, CH_2Cl_2) {lit[10]

(*ent*).−49.8 (*c* 0.58, CH_2Cl_2)}; Chiral HPLC analysis, Chiralcel OD-H (95:5 hexane:IPA, flow rate 1.0 mL min^{-1}, 211 nm, 30 °C) t_R (*R,R*): 9.6 min, t_R (*S,S*): 13.7 min, 94% ee; ^{1}H NMR (500 MHz, $CDCl_3$) δ_H: 2.49 (3H, s, CH_3), 5.46 (1H, s, C*H*), 7.42-7.50 (7H, m, Ar*H*).

Synthesis of Oxazolidin-4-ones

Using (±)-Oxaziridines

(2*R*,5*R*)-2,5-Diphenyl-3-tosyloxazolidin-4-one (*anti*-6a) and (2*S*,5*R*)-2,5-diphenyl-3-tosyloxazolidin-4-one (*syn*-6a)

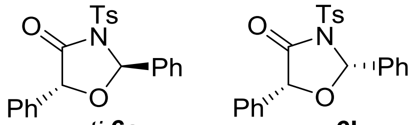

anti-**6a** *syn*-**6b**

Following *General Procedure 3*, homoanhydride **7** (76.3 mg, 0.3 mmol), cesium carbonate (131.5 mg, 0.4 mmol), oxaziridine **(±)-5** (55.1 mg, 0.2 mmol) and (2*S*,3*R*)-HyperBTM **3** (6.2 mg, 0.02 mmol) were stirred in dichloromethane (1 mL) at –78 °C to room temperature for 16 h to give the crude product (57:43 $dr_{anti:syn}$). Purification yielded the product **6** as a mixture of diastereoisomers (54:46 $dr_{anti:syn}$) as a white solid (65.2 mg, 83%). mp 154-157 °C; $[\alpha]_D^{22}$ +11.8 (*c* 0.5, $CHCl_3$); ν_{max} (neat) 1751 (C=O), 1373 (C-N), 1175 (R-SO_2N), 1088 (C-O); *m/z* (NSI^+) 395 ($[M+H]^+$, 100%); HMRS (NSI^+) $C_{22}H_{20}NSO_4$ $[M+H]^+$ found 394.1108, requires 394.1108 (+0.0 ppm).

Data for the *anti* diastereoisomer **6a**: Chiral HPLC analysis, Chiralcel AD-H (95:5 hexane:IPA, flow rate 1.5 mL min^{-1}, 211 nm, 40 °C) t_R (2*R*,5*R*): 27.1 min, t_R (2*S*,5*S*): 53.9 min, 97% ee; ^1H NMR (500 MHz, $CDCl_3$) δ_H: 2.41 (3H, s, CH_3), 5.44 (1H, d, *J* 1.2, C(5)*H*), 6.71 (1H, d, *J* 1.3, C(2)*H*), 7.17-7.19 (2H, m, SO_2ArC(3)*H*), 7.37-7.40 (10H, m, ArC*H*), 7.50-7.55 (2H, m, SO_2ArC(2)*H*); $^{13}C\{^1H\}$ NMR (125 MHz, $CDCl_3$) δ_C: 21.8 (CH_3), 77.4 (*C*(5)), 91.3 (*C*(2)), 126.5 (C(5)Ar*C*(2)), 127.5 (C(2)Ar*C*(2)), 128.4 (SO_2Ar*C*(2)), 128.8 (Ar*C*), 129.0 (Ar*C*), 129.2 (Ar*C*), 129.6 (SO_2Ar*C*(3)), 130.3 (Ar*C*), 134.4 (C(5)Ar*C*(1)), 134.8 (C(2)Ar*C*(1)), 136.6 (SO_2Ar*C*(1)), 145.8 (SO_2Ar*C*(4)), 168.8 (*C*(4)).

Selected data for the *syn* diastereoisomer **6b**: Chiral HPLC analysis, Chiralcel AD-H (95:5 hexane:IPA, flow rate 1.5 mL min^{-1}, 211 nm, 40 °C) t_R (2*S*,5*R*): 25.1 min, t_R (2*R*,5*S*): 30.4 min, 97% ee; ^1H NMR (500 MHz, $CDCl_3$) δ_H: 2.39 (3H, s, CH_3), 5.41 (1H, d, *J* 1.3, C(5)*H*), 6.59 (1H, d, *J* 1.4, C(2)*H*); $^{13}C\{^1H\}$ NMR (125 MHz, $CDCl_3$) δ_C: 21.8 (CH_3), 78.7 (*C*(5)), 90.9 (*C*(2)), 126.4 (C(5)Ar*C*(2)), 128.3 (C(2)Ar*C*(2)), 128.4 (SO_2Ar*C*(2)), 128.6 (Ar*C*), 128.7 (Ar*C*), 129.6 (SO_2Ar*C*(3)), 130.5 (Ar*C*), 134.0 (C(5)Ar*C*(1)), 135.1 (C(2)Ar*C*(1)), 136.4 (SO_2Ar*C*(1)), 145.7 (SO_2Ar*C*(4)), 168.6 (*C*(4)).

(2*R*,5*R*)-2-(2-Chlorophenyl)-5-phenyl-3-tosyloxazolidin-4-one (*anti*-11a) and (2*S*,5*R*)-2-(2-chlorophenyl)-5-phenyl-3-tosyloxazolidin-4-one (*syn*-11b)

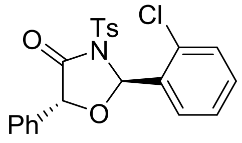

anti-**11a**

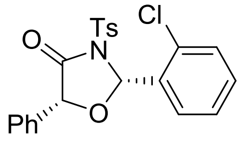

syn-**11b**

Following *General Procedure 3*, homoanhydride **7** (76.3 mg, 0.3 mmol), cesium carbonate (131.5 mg, 0.4 mmol), oxaziridine **(±)-8** (62.0 mg, 0.2 mmol) and (2*S*,3*R*)-HyperBTM **3** (6.2 mg, 0.02 mmol) were stirred at –78 °C to room temperature for 16 h to give the crude product (55:45 $dr_{anti:syn}$). Purification yielded the product **11** as a mixture of diastereoisomers (56:44 $dr_{anti:syn}$) as a white solid (66.6 mg, 78%). Careful purification allowed isolation of an analytical sample of each diastereoisomer for full characterisation.

Data for the *anti* diastereoisomer **11a**: white solid, mp 150-152 °C; $[\alpha]_D^{22}$ –16.6 (*c* 0.5, $CHCl_3$); Chiral HPLC analysis, Chiralcel AD-H (95:5 hexane:IPA, flow rate 1.5 mL min^{-1}, 211 nm, 30 °C) t_R (2*R*,5*R*): 23.0 min, t_R (2*S*,5*S*): 53.2 min, 78% ee; ^1H NMR (500 MHz, $CDCl_3$) δ_H: 2.43 (3H, s, CH_3), 5.38 (1H, d, *J* 1.3, C(5)*H*), 7.02 (1H, d, *J* 1.4, C(2)*H*), 7.14-7.25 (2H, m, SO_2ArC(3)*H*), 7.28-7.47 (9H, m, ArC*H*), 7.56-7.69 (2H, m, SO_2ArC(2)*H*).

Data for the *syn* diastereoisomer **11b**: white solid with data in accordance with the literature.[8] 156-158 °C {Lit.[8] 182-184 °C}; $[a]_D^{22}$ +40.2 (*c* 0.5, $CHCl_3$); Chiral HPLC analysis, Chiralcel AD-H (95:5 hexane:IPA, flow rate 1.5 mL min^{-1}, 211 nm, 30 °C), t_R (2*S*,5*R*): 40.4 min, t_R (2*R*,5*S*): 46.0 min, 78% ee; ^1H NMR (500 MHz, $CDCl_3$) δ_H: 2.43 (3H, s, CH_3), 5.35 (1H, d, *J* 1.3, C(5)*H*), 7.06 (1H, s, C(2)*H*), 7.23-7.31 (2H, m, SO_2ArC(3)*H*), 7.28-7.50 (9H, m), 7.75-7.64 (2H, m, SO_2ArC(3)*H*).

(2*R*,5*R*)-2-(4-Bromophenyl)-5-phenyl-3-tosyloxazolidin-4-one (*anti*-12a) and (2*S*,5*R*)-2-(4-Bromophenyl)-5-phenyl-3-tosyloxazolidin-4-one (*syn*-12b)

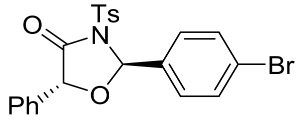

anti-**12a**

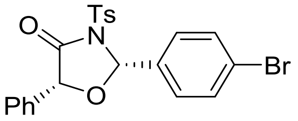

syn-**12b**

Following *General Procedure 3*, homoanhydride **7** (76.3 mg, 0.3 mmol), cesium carbonate (131.5 mg, 0.4 mmol), oxaziridine **(±)-9** (70.8 mg, 0.2 mmol) and (2*S*,3*R*)-HyperBTM **3** (6.2 mg, 0.02 mmol) were stirred at –78 °C to room temperature for 16 h to give the crude product (55:45 $dr_{anti:syn}$). Purification yielded the product **12** as a mixture of diastereoisomers (47:53 $dr_{anti:syn}$) as a white solid (77.5mg, 82%). Careful purification allowed isolation of an analytical sample of each diastereoisomer for full characterisation.

Data for the *anti* diastereoisomer **12a**: white solid, mp 96-100 °C; $[a]_D^{22}$+8.0 (*c* 0.5, $CHCl_3$); ν_{max} (neat) 1751 (C=O), 1373 (C-N), 1172 (R-SO_2N), 1068 (C-O); Chiral HPLC analysis, Chiralcel OD-H (90:10 hexane;IPA, flow rate 1.0 mL min^{-1}, 211 nm, 30 °C) t_R (2*R*,5*R*): 17.7 min, t_R (2*S*,5*S*): 19.6 min, 99% ee; ^{1}H NMR (500 MHz, $CDCl_3$) δ_H: 2.41 (3H, s, C*H*$_3$), 5.39 (1H, d, *J* 1.4, C(5)*H*), 6.54 (1H, d, *J* 1.4, C(2)*H*), 7.16-7.24 (2H, m, SO_2ArC(3)*H*), 7.23-7.30 (3H, m, Ar*H*), 7.30-7.41 (4H, m, Ar*H*), 7.46-7.56 (4H, m, Ar*H*); ^{13}C{^{1}H} NMR (125 MHz, $CDCl_3$) δ_C: 21.9 (*C*H$_3$), 79.0 (*C*(5)), 90.2 (*C*(2)), 124.8 (C(2)Ar*C*(4)), 126.3 (C(5)Ar*C*(2)), 128.3 (C(2)Ar*C*(2)), 128.8 (SO_2Ar*C*(2)), 129.1 (Ar*C*), 129.7 (C(2)Ar*C*(1)), 130.0 (SO_2Ar*C*(3)), 131.9 (Ar*C*), 133.8 (C(5)Ar*C*(1)), 134.9 (Ar*C*), 135.6 (SO_2Ar*C*(1)), 146.0 (SO_2Ar*C*(4)), 168.5 (*C*(4)); *m/z* (NSI$^+$) 472 ([M+NH$_4$]$^+$, 60%); HMRS (NSI$^+$) $C_{22}H_{19}{}^{79}BrNSO_4$ [M+H]$^+$ found 472.0203, requires 472.0213 (–2.1 ppm).

Data for the *syn* diastereoisomer **12b**: white solid, mp 162-168 °C; $[\alpha]_D^{22}$ –14.0 (*c* 0.5, $CHCl_3$); ν_{max} (neat) 1751 (C=O), 1375 (C-N), 1170 (R-SO_2N), 1090 (C-O); Chiral HPLC analysis, Chiralcel OD-H (90:10 hexane:IPA, flow rate 1.0 mL min^{-1}, 211 nm, 30 °C) t_R (2*R*,5*S*): 15.7 min, t_R (2*S*,5*R*): 23.6 min, 95% ee; ^{1}H NMR (500 MHz, $CDCl_3$) δ_H: 2.43 (3H, s, C*H*$_3$), 5.40 (1H, d, *J* 1.2, C(5)*H*), 6.64 (1H, d, *J* 1.2, C(2)*H*), 7.19-7.25 (2H, m, Ar*H*), 7.24-7.31 (4H, m, Ar*H*), 7.31-7.40 (5H, m, Ar*H*), 7.49-7.55 (2H, m, SO_2ArC(2)*H*); ^{13}C{^{1}H} NMR (125 MHz, $CDCl_3$) δ_C: 21.9 (*C*H$_3$), 78.8 (*C*(5)), 90.6 (*C*(2)), 124.6 (C(2)Ar*C*(4)), 126.5 (C(5)Ar*C*(2)), 128.3 (C(2)Ar*C*(2)), 129.0 (SO_2Ar*C*(2)), 129.2 (Ar*C*), 129.4 (Ar*C*), 129.7 (Ar*C*), 132.0 (SO_2Ar*C*(3)), 134.2 (Ar*C*), 134.8 (C(5)Ar*C*(1)), 135.7 (SO_2Ar*C*(1)), 146.0 (SO_2Ar*C*(4)), 168.7 (*C*(4)).

3-Nitro-4-((2*R*,5*R*)-4-oxo-2,5-diphenyloxazolidin-3-yl)benzenesulfonic (*anti*-13a) acid and 3-nitro-4-((2*S*,5*R*)-4-oxo-2,5-diphenyloxazolidin-3-yl)benzenesulfonic acid (*syn*-13b)

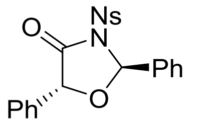

anti-**13a**

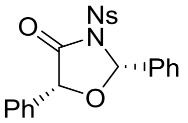

syn-**13b**

Following *General Procedure 3*, homoanhydride **7** (76.3 mg, 0.3 mmol), cesium carbonate (131.5 mg, 0.4 mmol), oxaziridine **(±)-10** (64.5 mg, 0.2 mmol) and (2*S*,3*R*)-HyperBTM **3** (6.2 mg, 0.02 mmol) were stirred at –78 °C to room temperature for 16 h to give the crude product (59:41 $dr_{anti:syn}$). Purification yielded the product **13** as a mixture of diastereoisomers (52:48 $dr_{anti:syn}$) as a white solid (62.0 mg, 73%).

Data for the *anti* diastereoisomer **13a**: white solid, mp 146-150 °C; $[\alpha]_D^{22}$+68.2 (*c* 0.5, $CHCl_3$); ν_{max} (neat) 1751 (C=O), 1531 (NO_2), 1384 (C-N), 1180 (R-SO_2N), 1086 (C-O); Chiral HPLC analysis, Chiralcel AD-H (80:20 hexane:IPA, flow rate 1.25 mL min^{-1}, 211 nm, 40 °C) t_R (2*R*,5*R*): 27.3 min, t_R (2*S*,5*S*): 29.9 min, 85% ee; ^{1}H NMR (500 MHz, $CDCl_3$) δ_H: 5.51 (1H, d, *J* 1.2, C(5)*H*), 6.72 (1H, d, *J*

1.2, C(2)*H*), 7.29-7.45 (9H, m, Ar*H*), 7.45-7.57 (1H, m, Ar*H*) 7.68-7.83 (2H, m, SO_2ArC(3)*H*), 8.12-8.26 (2H, m, SO_2ArC(2)*H*); $^{13}C\{^1H\}$ NMR (125 MHz, $CDCl_3$) δ_C: 78.9 (*C*(5)), 91.5 (*C*(2)), 124.1 (Ar*C*), 126.3 (C(5)Ar*C*(2)), 127.7 (C(2)Ar*C*(2)), 129.0 (SO_2Ar*C*(2)), 129.2 (2 Ar*C*), 129.6 (Ar*C*), 129.6 (SO_2Ar*C*(3)), 130.8 (Ar*C*), 133.9 (C_{ipso}), 136.1 (C(5)Ar*C*(1)), 143.1 (SO_2Ar*C*(1)), 151.0 (SO_2Ar*C*(4)), 168.7 (*C*(4)); *m/z* (NSI^+) 425 ($[M+H]^+$, 100%); HMRS $C_{21}H_{17}N_2O_6S$ $[M+H]^+$ found 425.0801, requires 425.0802 (–0.2 ppm).

Selected data for the *syn* diastereoisomer **13b**: white solid, mp 90-94 °C; $[a]_D^{22}$ –48.0 (*c* 0.1, $CHCl_3$); ν_{max} (neat) 1749 (C=O), 1533 (NO_2), 1386 (C-N), 1182 (R-SO_2N), 1087 (C-O); Chiral HPLC analysis, Chiralcel AD-H (80:20 hexane:IPA, flow rate 1.5 mL min^{-1}, 211 nm, 30 °C) t_R (2*S*,5*R*): 19.9 min, t_R (2*R*,5*S*): 22.6 min, 80% ee; ^1H NMR (500 MHz, $CDCl_3$) δ_H: 5.49 (1H, d, *J* 1.3, C(5)*H*), 6.62 (1H, d, *J* 1.4, C(2)*H*), 7.30-7.45 (9H, m, Ar*H*), 7.66 (2H, d, *J* 8.9, SO_2ArC(3)*H*), 8.16 (2H, d, *J* 8.9 SO_2ArC(2)*H*); $^{13}C\{^1H\}$ NMR (125 MHz, $CDCl_3$) δ_C: 79.0 (*C*(5)), 90.9 (*C*(2)), 124.1 (C(5)Ar*C*(2)), 126.4 (C(2)Ar*C*(2)), 128.7 (SO_2Ar*C*(2)), 128.8 (Ar*C*), 128.9 (Ar*C*), 129.3 (Ar*C*), 129.5 (SO_2Ar*C*(3)), 131.0 (Ar*C*), 133.4 (C(5)Ar*C*(1)), 135.6 (C(2)Ar*C*(1)), 143.5 (SO_2Ar*C*(4)), 168.5 (*C*(4)).

(2*R*,5*R*)-5-(4-Fluorophenyl)-2-phenyl-3-tosyloxazolidin-4-one (*anti*-17a) and (2*S*,5*R*)-5-(4-fluorophenyl)-2-phenyl-3-tosyloxazolidin-4-one (*syn*-17b)

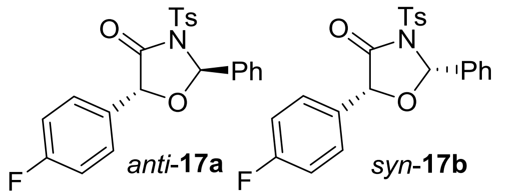

Following *General Procedure 3*, homoanhydride **S1** (87.1 mg, 0.3 mmol), cesium carbonate (131.5 mg, 0.4 mmol), oxaziridine **(±)-5** (55.1 mg, 0.2 mmol) and (2*S*,3*R*)-HyperBTM **3** (6.2 mg, 0.02 mmol) were stirred at –78 °C to room temperature for 16 h to give the crude product (54:46 $dr_{anti:syn}$). Purification yielded the product as a mixture of diastereoisomers (55:45 $dr_{anti:syn}$) as a white solid (60.4 mg, 73%). mp 84-90 °C; $[\alpha]_D^{22}$ +5.6 (*c* 0.5, $CHCl_3$); ν_{max} (neat) 1749 (C=O), 1508, 1373 (C-N), 1174 (R-SO_2N), 1068 (C-O); *m/z* (NSI^+) 412 ($[M+H]^+$, 100%); HMRS (NSI^+) $C_{22}H_{19}FNSO_4$ $[M+H]^+$ found 412.1007, requires 412.1013 (–1.5 ppm).

Data for the *anti* diastereoisomer **17a**: Chiral HPLC analysis, Chiralcel OD-H (90:10 hexane:IPA, flow rate 1.0 mL min^{-1}, 211 nm, 30 °C), t_R (2*S*,5*S*): 17.0 min, t_R (2*R*,5*R*): 18.9 min, 99% ee; ^1H NMR (500 MHz, $CDCl_3$) δ_H: 2.41 (3H, s, C*H*$_3$), 5.40 (1H, s, C(5)*H*), 6.70 (1H, d, *J* 1.3, C(2)*H*), 6.97-7.14 (2H, m, Ar*H*), 7.13-7.23 (2H, m, Ar*H*), 7.30-7.44 (5H, m, Ar*H*), 7.40-7.52 (2H, m, Ar*H*), 7.49-7.60 (2H, m, Ar*H*); $^{13}C\{^1H\}$ NMR (125 MHz, $CDCl_3$) δ_C: 21.9 (*C*H_3), 78.1 (*C*(5)), 91.3 (*C*(2)), 116.0 (d, $^2J_{CF}$ 21.8, C(5)Ar*C*(3)), 127.5 (C(2)Ar*C*(4)), 128.3 (C(2)Ar*C*(2)), 128.4 (Ar*C*), 128.6 (SO_2Ar*C*(2)), 129.9 (SO_2Ar*C*(3)), 130.3 (d, $^4J_{CF}$ 3.2, C(5)Ar*C*(1)), 130.4 (Ar*C*), 135.0 (SO_2Ar*C*(1)), 136.0 (Ar*C*),

145.9 (SO_2Ar*C*(4)), 163.3 (d, $^1J_{CF}$ 246, C(5)Ar*C*(4)), 168.6 (*C*(4)); ^{19}F{^{1}H} NMR (282 MHz, $CDCl_3$) δ_F: –112.3.

Selected data for the *syn* diastereoisomer **17b**: Chiral HPLC analysis, Chiralcel OD-H (90:10 hexane:IPA, flow rate 1.0 mL min^{-1}, 211 nm, 30 °C) t_R (2*S*,5*R*): 15.9 min, t_R (2*R*,5*S*): 27.8 min, 99% ee; ^{1}H NMR (500 MHz, $CDCl_3$) δ_H: 2.39 (3H, s, C*H*$_3$), 5.38 (1H, s, C(5)*H*), 6.57 (1H, d, *J* 1.4, C(2)*H*); ^{13}C{^{1}H} NMR (125 MHz, $CDCl_3$) δ_C: 21.9 (*C*H$_3$), 78.5 (*C*(5)), 91.0 (*C*(2)), 116.0 (d, $^2J_{CF}$ 21.9, C(5)Ar*C*(3)), 128.2 (C(2)Ar*C*(2)), 128.5 (SO_2Ar*C*(2)), 129.9 (d, $^4J_{CF}$ 3.4, C(5)Ar*C*(1)), 130.6 (SO_2Ar*C*(3)), 134.9 (Ar*C*), 136.3 (SO_2Ar*C*(1)), 145.8 (SO_2Ar*C*(4)), 163.2 (d, $^1J_{CF}$ 246, C(5)Ar*C*(4)), 168.4 (*C*(4)); ^{19}F{^{1}H} NMR (282 MHz, $CDCl_3$ δ_F: –112.8.

(2*R*,5*R*)-5-(4-Methoxyphenyl)-2-phenyl-3-tosyloxazolidin-4-one and (*anti*-18a) (2*S*,5*R*)-5-(4-methoxyphenyl)-2-phenyl-3-tosyloxazolidin-4-one (*syn*-18b)

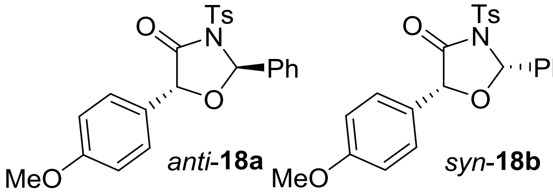

Following *General Procedure 3*, homoanhydride **S2** (94.3 mg, 0.3 mmol), cesium carbonate (131.5 mg, 0.4 mmol), oxaziridine **(±)-5** (55.1 mg, 0.2 mmol) and (2*S*,3*R*)-HyperBTM **3** (6.2 mg, 0.02 mmol) were stirred at –78 °C to room temperature for 16 h to give the crude product (55:45 $dr_{anti:syn}$). Purification yielded the product **18** as a mixture of diastereoisomers (55:45 $dr_{anti:syn}$) as a colourless oil (75.4 mg, 89%). $[\alpha]_D^{22}$ +7.8 (*c* 0.5, $CHCl_3$); ν_{max} (neat) 1749 (C=O), 1373 (C-N), 1175 (R-SO_2N), 1088 (C-O); *m/z* (NSI) 424 ([M+H]$^+$, 100%); HMRS (NSI) $C_{23}H_{22}NSO_5$ [M+H]$^+$ found 424.1214, requires 424.1213 (+0.2 ppm).

Data for the *anti* diastereoisomer **18a**: Chiral HPLC analysis, Chiralcel OD-H (90:10 hexane:IPA, flow rate 1.0 mL min^{-1}, 211 nm, 30 °C) t_R (2*S*,5*S*): 30.6 min, t_R (2*R*,5*R*): 33.4 min, 97% ee; ^{1}H NMR (500 MHz, $CDCl_3$) δ_H: 2.41 (3H, s, SO_2ArC*H*$_3$), 3.80 (3H, s, OC*H*$_3$), 5.37 (1H, s, C(5)*H*), 6.68 (1H, d, *J* 1.2, C(2)*H*), 6.87-6.92 (2H, m, C(5)ArC(3)*H*), 7.16-7.23 (2H, m, SO_2ArC(2)*H*), 7.22-7.29 (2H, m, Ar*H*), 7.36-7.40 (3H, m, Ar*H*), 7.46-7.48 (2H, m, Ar*H*), 7.51-7.58 (2H, m, Ar*H*); ^{13}C{^{1}H} NMR (125 MHz, $CDCl_3$) δ_C: 21.9 (Ar*C*H$_3$), 55.5 (O*C*H$_3$), 78.6 (*C*(5)), 91.1 (*C*(2)), 114.4 (C(5)Ar*C*(3)), 126.5 (C(5)Ar*C*(1)), 127.5 (C(2)Ar*C*(2)), 128.3 (C(5)Ar*C*(2)), 128.4 (SO_2Ar*C*(2)), 128.6 (Ar*C*), 128.8 (Ar*C*), 129.6 (SO_2Ar*C*(3)), 130.0 (Ar*C*), 134.9 (SO_2Ar*C*(1)), 136.7 (C(2)Ar*C*(1)), 145.7 (SO_2Ar*C*(4)), 160.4 (C(5)Ar*C*(4)), 169.1 (*C*(4)).

Selected data for the *syn* diastereoisomer **18b**: Chiral HPLC analysis, Chiralcel OD-H (90:10 hexane:IPA, flow rate 1.0 mL min^{-1}, 211 nm, 30 °C) t_R (2*S*,5*R*): 24.9 min, t_R (2*R*,5*S*): 27.6 min, 94% ee; ^{1}H NMR (500 MHz, $CDCl_3$) δ_H: 2.39 (3H, s, SO_2ArC*H*$_3$), 3.78 (3H, s, OC*H*$_3$), 5.35 (1H, s, C(5)*H*),

6.55 (1H, d, *J* 1.4, C(2)*H*), 6.86-6.89 (2H, m, C(5)Ar*C*(3)*H*), 7.15-7.18 (2H, m, SO_2ArC(2)*H*); $^{13}C\{^1H\}$ NMR (125 MHz, $CDCl_3$) δ_C: 21.8 (Ar*C*H_3), 55.5 (O*C*H_3), 79.0 (*C*(5)), 90.8 (*C*(2)), 114.2 (C(5)Ar*C*(3)), 126.1 (C(5)Ar*C*(1)), 127.5 (C(2)Ar*C*(2)), 128.3 (C(5)Ar*C*(2)), 128.4 (SO_2Ar*C*(2)), 128.6 (Ar*C*), 128.8 (Ar*C*), 129.6 (SO_2Ar*C*(3)), 130.0 (Ar*C*), 135.2 (SO_2Ar*C*(1)), 136.4 (C(2)Ar*C*(1)), 145.6 (SO_2Ar*C*(4)), 160.4 (C(5)Ar*C*(4)), 169.0 (*C*(4)).

(2*R*,5*R*)-5-(Naphthalen-2-yl)-2-phenyl-3-tosyloxazolidin-4-one (*anti*-19a) and (2*S*,5*R*)-5-(naphthalen-2-yl)-2-phenyl-3-tosyloxazolidin-4-one (*syn*-19b)

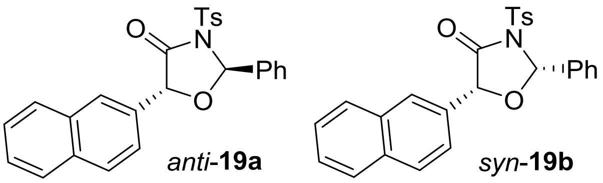

Following *General Procedure 3* homoanhydride **S3** (106.3 mg, 0.3 mmol), cesium carbonate (131.5 mg, 0.4 mmol), oxaziridine **(±)-5** (55.1 mg, 0.2 mmol) and (2*S*,3*R*)-HyperBTM **3** (6.2 mg, 0.02 mmol) were stirred at –78 °C to room temperature for 16 h to give the crude product (53:47 $dr_{anti:syn}$). Purification yielded the product as a mixture of diastereoisomers (58:42 $dr_{anti:syn}$) as a white solid (47.0 mg, 48%). mp 136-140 °C; $[\alpha]_D^{22}$ +31.8 (*c* 0.5, $CHCl_3$); ν_{max} (neat) 1749 (C=O), 1373 (C-N), 1175 (R-SO_2N), 1088 (C-O); *m/z* (NSI^+) 461 ($[M+NH_4]^+$, 60%); HMRS (NSI^+) $C_{26}H_{22}NSO_4$ $[M+H]^+$ found 444.1254, requires 444.1264 (–2.3 ppm).

Data for the *anti* diastereoisomer **19a**: Chiral HPLC analysis, Chiralcel AD-H (90:10 hexane:IPA, flow rate 1.0 mL min^{-1}, 211 nm, 40 °C) t_R (2*R*,5*R*): 35.7 min, t_R (2*S*,5*S*): 84.8 min, >99% ee; ^1H NMR (500 MHz, $CDCl_3$) δ_H: 2.39 (3H, s, ArC*H*$_3$), 5.60 (1H, s, C(5)*H*), 6.77 (1H, d, *J* 1.2, C(2)*H*), 7.16-7.20 (2H, m, SO_2ArC(2)*H*), 7.35-7.60 (9H, m, Ar*H*), 7.79-7.89 (4H, m, Ar*H*); $^{13}C\{^1H\}$ NMR (125 MHz, $CDCl_3$) δ_C: 21.8 (Ar*C*H_3), 78.9 (*C*(5)), 91.4 (*C*(2)), 123.7 (Ar*C*), 126.1 (Ar*C*), 126.7 (Ar*C*), 126.8 (Ar*C*), 127.5 (C(2)Ar*C*(2)), 127.9 (Ar*C*), 128.3 (Ar*C*), 128.4 (SO_2Ar*C*(2)), 128.7 (Ar*C*), 128.9 (Ar*C*), 129.6 (SO_2Ar*C*(3)), 130.3 (Ar*C*), 131.7 (C(5)Ar*C*(1)), 133.1 (C_{ipso}), 133.6 (C_{ipso}), 134.8 (SO_2Ar*C*(1)), 136.7 (C(2)Ar*C*(1)), 145.8 (SO_2Ar*C*(4)), 168.8 (*C*(4)).

Selected data for the *syn* diastereoisomer **19b**: Chiral HPLC analysis, Chiralcel AD-H (90:10 hexane:IPA, flow rate 1.0 mL min^{-1}, 211 nm, 40 °C) t_R (2*S*,5*R*): 30.7 min, t_R (2*R*,5*S*): 47.1 min, >99% ee; ^1H NMR (500 MHz, $CDCl_3$) δ_H: 2.37 (3H, s, ArC*H*$_3$), 5.59 (1H, s, C(5)*H*), 6.65 (1H, d, *J* 1.2, C(2)*H*), 7.11-7.16 (2H, m, SO_2ArC(2)*H*); $^{13}C\{^1H\}$ NMR (125 MHz, $CDCl_3$) δ_C: 21.8 (Ar*C*H_3), 79.2 (*C*(5)), 91.1 (*C*(2)), 123.6 (Ar*C*), 125.8 (Ar*C*), 126.5 (Ar*C*), 126.7 (Ar*C*), 127.5 (C(2)Ar*C*(2)), 127.8 (Ar*C*), 128.3 (C(5)Ar*C*(2)), 128.5 (SO_2Ar*C*(2)), 128.6 (Ar*C*), 129.0 (Ar*C*), 129.6 (SO_2Ar*C*(3)), 130.5 (Ar*C*), 131.5 (C(5)Ar*C*(1)), 133.0 (C_{ipso}), 133.5 (C_{ipso}), 135.2 (SO_2Ar*C*(1)), 136.5 (C(2)Ar*C*(1)), 145.7 (SO_2Ar*C*(4)), 168.6 (*C*(4)).

(2*R*,5*R*)-2-Phenyl-5-(*p*-tolyl)-3-tosyloxazolidin-4-one (*anti*-20a) and (2*S*,5*R*)-2-phenyl-5-(*p*-tolyl)-3-tosyloxazolidin-4-one (*syn*-20b)

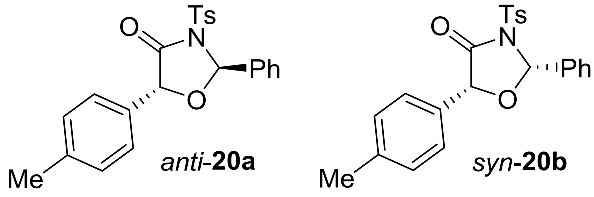

Following *General Procedure 3*, homoanhydride **S4** (84.7 mg, 0.3 mmol), cesium carbonate (131.5 mg, 0.4 mmol), oxaziridine **(±)-5** (55.1 mg, 0.2 mmol) and (2*S*,3*R*)-HyperBTM **3** (6.2 mg, 0.02 mmol) were stirred at –78 °C to room temperature for 16 h to give the crude product (53:47 $dr_{anti:syn}$). Purification yielded the product **20** as a mixture of diastereoisomers (54.46 $dr_{anti:syn}$) as a white solid (71.6 mg, 88%). mp 138-142 °C; $[\alpha]_D^{22}$ –3.0 (*c* 0.5, $CHCl_3$); ν_{max} (neat) 1749 (C=O), 1373 (C-N), 1175 (R-SO_2N); *m/z* (NSI^+) 408 ($[M+H]^+$, 85%); HMRS (NSI^+) $C_{23}H_{22}NSO_4$ $[M+H]^+$ found 408.1259, requires 408.1264 (–1.2 ppm).

Data for the *anti* diastereoisomer **20a**: Chiral HPLC analysis, Chiralcel OD-H (90:10 hexane:IPA, flow rate 1.0 mL min^{-1}, 211 nm, 30 °C) t_R (2*R*,5*R*): 17.3 min, t_R (2*S*,5*S*): 22.7 min, 97% ee; ^1H NMR (500 MHz, $CDCl_3$) δ_H: 2.35 (3H, s, C(5)ArC*H*$_3$), 2.41 (3H, s, SO_2ArC*H*$_3$), 5.39 (1H, s, C(5)*H*), 6.69 (1H, d, *J* 1.2, C(2)*H*), 7.16-7.21 (4H, m, Ar*H*), 7.24 (2H, d, *J* 8.1, Ar*H*), 7.34-7.43 (3H, m, Ar*H*), 7.40-7.50 (2H, m, Ar*H*), 7.50-7.58 (2H, m, Ar*H*); $^{13}C\{^1H\}$ NMR (125 MHz, $CDCl_3$) δ_C: 21.3 (C(5)Ar*C*H$_3$), 21.7 (SO_2Ar*C*H$_3$), 78.7 (*C*(5)), 91.1 (*C*(2)), 126.5 (C(5)Ar*C*(2)), 127.4 (2 Ar*C*), 128.2 (C(2)Ar*C*(2)), 128.7 (SO_2Ar*C*(2)), 129.5 (SO_2Ar*C*(3)), 129.6 (2 Ar*C*), 130.1 (Ar*C*), 131.4 (C(5)Ar*C*(1)), 134.8 (SO_2Ar*C*(1)), 136.6 (C(2)Ar*C*(1)), 139.2 (C(5)Ar*C*(4)), 145.6 (SO_2Ar*C*(4)), 168.9 (*C*(4)).

Selected data for the *syn* diastereoisomer **20b**: Chiral HPLC analysis, Chiralcel OD-H (90:10 hexane:IPA, flow rate 1.0 mL min^{-1}, 211 nm, 30 °C) t_R (2*S*,5*R*): 15.3 min, t_R (2*R*,5*S*): 27.1 min, 99% ee; ^1H NMR (500 MHz, $CDCl_3$) δ_H: 2.33 (3H, s, C(5)ArC*H*$_3$), 2.39 (3H, s, SO_2ArC*H*$_3$), 5.38 (1H, s, C(5)*H*); $^{13}C\{^1H\}$ NMR (125 MHz, $CDCl_3$) δ_C: 21.2 (C(5)Ar*C*H$_3$), 21.7 (SO_2Ar*C*H$_3$), 79.0 (*C*(5)), 90.7 (*C*(2)), 126.3 (C(5)Ar*C*(2)), 128.1 (C(2)Ar*C*(2)), 128.3 (2×Ar*C*), 128.5 (SO_2Ar*C*(2)), 129.3 (SO_2Ar*C*(3)), 130.3 (Ar*C*), 131.0 (C(5)Ar*C*(1)), 134.8 (SO_2Ar*C*(1)), 136.3 (C(2)Ar*C*(1)), 138.8 (C(5)Ar*C*(4)), 145.4 (SO_2Ar*C*(4)), 168.7 (*C*(4)).

(2*R*,5*R*)-2-Phenyl-5-(*m*-tolyl)-3-tosyloxazolidin-4-one (*anti*-21a) and (2*S*,5*R*)-2-phenyl-5-(*m*-tolyl)-3-tosyloxazolidin-4-one (*syn*-21b)

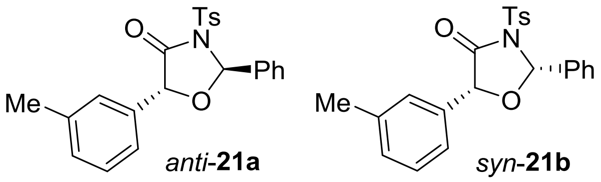

Following *General Procedure 3*, homoanhydride **S5** (84.7 mg, 0.3 mmol), cesium carbonate (131.5 mg, 0.4 mmol), oxaziridine **(±)-5** (55.1 mg, 0.2 mmol) and (2*S*,3*R*)-HyperBTM **3** (6.2 mg, 0.02 mmol) were stirred at −78 °C to room temperature for 16 h to give the crude product (51:49 $dr_{anti:syn}$). Purification yielded the product **21** as a mixture of diastereoisomers (54.46 $dr_{anti:syn}$) as a colourless oil (64.4 mg, 79%). $[\alpha]_D^{22}$ +11.6(*c* 0.5, $CHCl_3$); ν_{max} (neat) 1751 (C=O), 1373 (C-N), 1175 (R-SO_2N), 1088 (C-O); *m/z* 408 (NSI^+) ($[M+H]^+$, 100%); HMRS (NSI^+) $C_{23}H_{22}NSO_4$ $[M+H]^+$ found 408.1257, requires 408.1264 (–1.7 ppm).

Data for the *anti* diastereoisomer **21a**: Chiralpak AD-H (90:10 hexane:IPA, flow rate 1.0 mL min^{-1}, 211 nm, 40 °C) t_R (2*R*,5*R*): 22.5 min, t_R (2*S*,5*S*): 45.1 min, 92% ee; ^{1}H NMR (500 MHz, $CDCl_3$) δ_H: 2.20 (3H, s, C(5)ArC*H*$_3$), 2.27 (3H, s, SO_2ArC*H*$_3$), 5.25 (1H, s, C(5)*H*), 6.57 (1H, d, *J* 1.3, C(2)*H*), 6.96-7.08 (4H, m, Ar*H*), 7.08-7.15 (2H, m, Ar*H*), 7.21-7.29 (3H, m, Ar*H*), 7.29-7.38 (2H, m, Ar*H*), 7.37-7.27 (2H, m, Ar*H*); ^{13}C{^{1}H} NMR (125 MHz, $CDCl_3$) δ_C: 21.5 (C(5)Ar*C*H$_3$), 21.8 (SO_2Ar*C*H$_3$), 78.8 (*C*(5)), 91.3 (*C*(2)), 123.8 (Ar*C*), 127.2 (Ar*C*), 127.5 (Ar*C*), 128.4 (C(2)Ar*C*(2)), 128.6 (SO_2Ar*C*(2)), 128.8 (2 ×Ar*C*), 129.6 (SO_2Ar*C*(3)), 130.1 (Ar*C*), 130.3 (Ar*C*), 134.4 (C(5)Ar*C*(1)), 134.9 (SO_2Ar*C*(1)), 136.7 (C(2)Ar*C*(1)), 138.8 (C(5)Ar*C*(2)CH$_3$), 145.7 (SO_2Ar*C*(4)), 169.0 (*C*(4)).

Selected data for the *syn* diastereoisomer **21b**: Chiralpak AD-H (90:10 hexane:IPA, flow rate 1.0 mL min^{-1}, 211 nm, 40 °C) t_R (2*S*,5*R*): 20.5 min, t_R (2*R*,5*S*): 28.3 min, 94% ee; ^{1}H NMR (500 MHz, $CDCl_3$) δ_H: 2.18 (3H, s, C(5)ArC*H*$_3$), 2.25 (3H, s, SO_2ArC*H*$_3$), 5.23 (1H, d, *J* 1.2, C(5)*H*), 6.44 (1H, d, *J* 1.4, C(2)*H*); ^{13}C{^{1}H} NMR (125 MHz, $CDCl_3$) δ_C: 21.6 (C(5)Ar*C*H$_3$), 21.8 (SO_2Ar*C*H$_3$), 79.1 (*C*(5)), 90.9 (*C*(2)), 123.4 (Ar*C*), 127.1 (Ar*C*), 127.5 (2 × Ar*C*), 128.3 (C(2)Ar*C*(2)), 128.4 (SO_2Ar*C*(2)), 128.9 (Ar*C*), 129.6 (SO_2Ar*C*(3)), 129.8 (Ar*C*), 130.5 (Ar*C*), 133.9 (C(5)Ar*C*(1)), 135.1 (SO_2Ar*C*(1)), 136.5 (C(2)Ar*C*(1)), 138.4 (C(5)Ar*C*(2)CH$_3$), 145.6 (SO_2Ar*C*(4)), 168.7 (*C*(4)).

(2*R*,5*R*)-2-Phenyl-5-(*o*-tolyl)-3-tosyloxazolidin-4-one (*anti*-22a) and (2*S*,5*R*)-2-phenyl-5-(*o*-tolyl)-3-tosyloxazolidin-4-one (*syn*-22b)

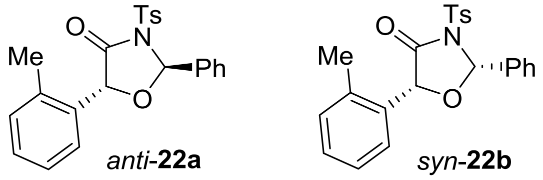

Following *General Procedure 3*, homoanhydride **S6** (84.7 mg, 0.3 mmol), cesium carbonate (131.5 mg, 0.4 mmol), oxaziridine **(±)-5** (55.1 mg, 0.2 mmol) and (2*S*,3*R*)-HyperBTM **3** (6.2 mg, 0.02 mmol) were stirred at –78 °C to room temperature for 16 h to give the crude product (53:47 $dr_{anti:syn}$). Purification yielded the product **22** as a mixture of diastereoisomers (58:42 $dr_{anti:syn}$) as a white solid (78.5 mg, 96%). Careful purification allowed isolation of an analytical sample of each diastereoisomer for full characterisation.

Data for the *anti* diastereoisomer **22a**: white solid, mp 90-96 °C; $[\alpha]_D^{22}$ +42.8 (*c* 0.5, $CHCl_3$); ν_{max} (neat) 1749 (C=O), 1373 (C-N), 1175 (R-SO_2N), 1088 (C-O); Chiral HPLC analysis, Chiralpak AD-H (90:10 hexane:IPA, flow rate 1.0 mL min^{-1}, 211 nm, 40 °C) t_R (2*S*,5*S*): 22.9 min, t_R (2*R*,5*R*): 25.5 min, >99% ee; ^{1}H NMR (500 MHz, $CDCl_3$) δ_H: 2.30 (3H, s, C(5)ArC*H*$_3$), 2.43 (3H, s, SO_2ArC*H*$_3$), 5.60 (1H, d, *J* 1.2, C(5)*H*), 6.70 (1H, d, *J* 1.2, C(2)*H*), 7.14-7.20 (3H, m, Ar*H*), 7.20-7.25 (2H, m, Ar*H*), 7.37-7.47 (5H, m, Ar*H*), 7.58-7.64 (2H, m, Ar*H*); ^{13}C{^{1}H} NMR (125 MHz, $CDCl_3$) δ_C: 19.3 (C(5)Ar*C*H_3), 21.9 (SO_2Ar*C*H_3), 77.4 (*C*(5)), 91.0 (*C*(2)), 126.4 (Ar*C*), 127.4 (C(2)Ar*C*(2)), 127.6 (Ar*C*), 128.5 (SO_2Ar*C*(2)), 128.9 (2 Ar*C*), 129.5 (Ar*C*), 129.7 (SO_2Ar*C*(3)), 130.2 (Ar*C*), 131.3 (Ar*C*), 132.8 (C(5)Ar*C*(1)), 134.8 (SO_2Ar*C*(1)), 136.9 (C(2)Ar*C*(1)), 137.3 (C(5)Ar*C*(2)CH_3), 145.8 (SO_2Ar*C*(4)), 169.1 (*C*(4)); *m/z* (NSI^+) 408 ($[M+H]^+$, 99%) HMRS (NSI^+) $C_{23}H_{22}NSO_4^+$ ($[M+H]^+$) requires 408.1264; found 408.1258 (–1.5 ppm).

Data for the *syn* diastereoisomer **22b**: white solid, mp 126-132 °C; $[a]_D^{22}$ –56.2 (*c* 0.5, $CHCl_3$); ν_{max} (neat) 1749 (C=O), 1373 (C-N), 1172 (R-SO_2N), 1088 (C-O); Chiral HPLC analysis, Chiralpak AD-H (90:10 hexane: IPA, flow rate 1.0 mL min^{-1}, 211 nm, 40 °C) t_R (2*R*,5*S*): 16.8 min, t_R (2*S*,5*R*): 18.7 min, >99% ee; ^{1}H NMR (500 MHz, $CDCl_3$) δ_H: 2.40 (3H, s, C(5)ArC*H*$_3$), 2.45 (3H, s, SO_2ArC*H*$_3$), 5.66 (1H, d, *J* 1.6, C(5)*H*), 6.57 (1H, d, *J* 1.6, C(2)*H*), 7.12-7.19 (3H, m, Ar*H*), 7.19-7.25 (2H, m, Ar*H*), 7.29-7.34 (1H, m, Ar*H*), 7.35-7.52 (7H, m, Ar*H*); ^{13}C{^{1}H} NMR (125 MHz, $CDCl_3$) δ_C: 19.7 (C(5)Ar*C*H_3), 21.8 (SO_2Ar*C*H_3), 77.4 (*C*(5)), 90.8 (*C*(2)), 126.3 (Ar*C*), 126.6 (Ar*C*), 128.2 (C(2)Ar*C*(2)), 128.6 (SO_2Ar*C*(2)), 128.6 (2 × Ar*C*), 129.2 (Ar*C*), 129.6 (SO_2Ar*C*(3)), 130.5 (Ar*C*), 131.0 (Ar*C*), 132.2 (C(5)Ar*C*(1)), 135.3 (SO_2Ar*C*(1)), 136.1 (C(2)Ar*C*(1)), 137.1 (C(5)Ar*C*(2)CH_3), 145.6 (SO_2Ar*C*(4)), 168.8 (*C*(4)).

(2*R*,5*R*)-2-Phenyl-5-(thiophen-3-yl)-3-tosyloxazolidin-4-one (*anti*-23a) and (2*S*,5*R*)-2-phenyl-5-(thiophen-3-yl)-3-tosyloxazolidin-4-one (*syn*-23b)

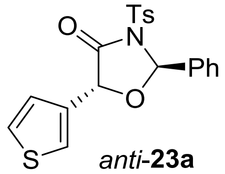
anti-**23a**

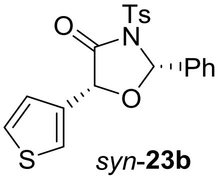
syn-**23b**

Following *General Procedure 3*, homoanhydride **S7** (79.9 mg, 0.3 mmol), cesium carbonate (131.5 mg, 0.4 mmol), oxaziridine **(±)-5** (55.1 mg, 0.2 mmol) and (2*S*,3*R*)-HyperBTM **3** (6.2 mg, 0.02 mmol) were stirred at –78 °C to room temperature for 16 h to give the crude product (59:41 $dr_{anti:syn}$). Purification yielded the product **23** as a mixture of diastereoisomers (59:41 $dr_{anti:syn}$) as a waxy white solid (62.8 mg, 79%). mp 90-96 °C; $[\alpha]_D^{22}$ +32.8 (*c* 0.5, $CHCl_3$); ν_{max} (neat) 1751 (C=O), 1373 (C-N), 1175 (R-SO_2N), 1088 (C-O); *m/z* (NSI^+) 400 ($[M+NH_4]^+$, 75%); HMRS (NSI^+) $C_{20}H_{18}NS_2O_4$ $[M+H]^+$ found 400.0667, requires 400.0672 (–1.2 ppm).

Data for the *anti* diastereoisomer **23a**: Chiral HPLC analysis, Chiralpak IA (80:20 hexane:IPA, flow rate 1.5 mL min^{-1}, 211 nm, 30 °C) t_R (2*R*,5*R*): 8.7 min, t_R (2*S*,5*S*): 13.2 min, 87% ee; ^{1}H NMR (500 MHz, $CDCl_3$) δ_H: 2.35 (3H, s, SO_2ArC*H$_3$*), 5.44 (1H, s, C(5)*H*), 6.55 (1H, d, *J* 1.2, C(2)*H*), 6.98-7.02 (1H, m, Ar*H*), 7.11-7.15 (2H, m, SO_2Ar*H*), 7.19 (1H, s, C(5)ArC(2)*H*), 7.28 (2H, dd, *J* 1.8, 1.0, Ar*H*) 7.32 (2H, app. s, Ar*H*), 7.33-7.41 (2H, m, Ar*H*), 7.46 (2H, d, *J* 8.4, SO_2Ar*H*); ^{13}C{^{1}H} NMR (125 MHz, $CDCl_3$) δ_C: 21.7 (SO_2Ar*C*H_3), 75.5 (*C*(5)), 91.1 (*C*(2)), 123.7 (C(5)Ar*C*(2)), 125.5 (Ar*C*), 127.1 (Ar*C*), 127.5 (Ar*C*), 128.2 (C(2)Ar*C*(2)), 128.7 (SO_2Ar*C*(2)), 129.5 (SO_2Ar*C*(3)), 130.9 (C(5)Ar*C*(1)), 134.7 (SO_2Ar*C*(1)), 135.0 (C(2)Ar*C*(1)), 136.3 (C(5)Ar*C*(4)), 145.7 (SO_2Ar*C*(4)), 168.3 (*C*(4)).

Data for the *syn* diastereoisomer **23b**: Chiral HPLC analysis, Chiralpak IA (80:20 hexane:IPA, flow rate 1.5 mL min^{-1}, 211 nm, 30 °C) t_R (2*S*,5*R*): 9.4 min, t_R (2*R*,5*S*): 9.8 min, 81% ee; ^{1}H NMR (500 MHz, $CDCl_3$) δ_H: 2.32 (3H, s, SO_2ArC*H$_3$*), 5.43 (1H, d, *J* 1.2 C(5)*H*), 6.49 (1H, d, *J* 1.3, C(2)*H*), 7.04-7.07 (1H, m, Ar*H*), 7.01-7.10 (2H, m, SO_2Ar*H*), 7.24 (1H, s, C(5)ArC(2)*H*), 7.33-7.41 (2H, m, SO_2Ar*H*); ^{13}C{^{1}H} NMR (125 MHz, $CDCl_3$) δ_C: 21.7 (SO_2Ar*C*H_3), 76.2 (*C*(5)), 91.0 (*C*(2)), 123.2 (C(5)Ar*C*(2)), 126.6 (Ar*C*), 128.1 (2 Ar*C*), 128.2 (C(2)Ar*C*(2)), 128.5 (SO_2Ar*C*(2)), 130.3 (2 Ar*C*), 134.6 (SO_2Ar*C*(1)), 134.9 (C(2)Ar*C*(1)), 136.3 (C(5)Ar*C*(4)), 145.6 (SO_2Ar*C*(4)), 168.0 (*C*(4)).

(2*R*,5*R*)-2-Phenyl-5-((*E*)-prop-1-en-1-yl)-3-tosyloxazolidin-4-one (*anti*-24a) and (2*S*,5*R*)-2-phenyl-5-((*E*)-prop-1-en-1-yl)-3-tosyloxazolidin-4-one (*syn*-24b)

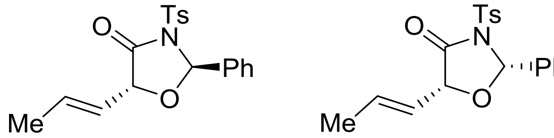

anti-**24a** *syn*-**24b**

Following *General Procedure K*, homoanhydride **S8** (1.2 g, 6.5 mmol), cesium carbonate (2.8 g, 8.6 mmol), oxaziridine **(±)-5** (1.2 g, 4.3 mmol) and (2*S*,3*R*)-HyperBTM **3** (0.13 g, 0.43 mmol) were stirred at –78 °C for 24 h to give the crude product **24** (49:51 $dr_{anti:syn}$). Purification yielded the product as a mixture of diastereoisomers (54:46 $dr_{anti:syn}$) as a colourless oil (1.41 g, 61%).

Data for the *anti* diastereoisomer **24a**: colourless oil, $[\alpha]_D^{22}$ +10.0 (*c* 0.2, $CHCl_3$); ν_{max} (neat) 1749 (C=O), 1373 (C-N), 1175 (R-SO_2N), 1090 (C-O); Chiral HPLC analysis, Chiralcel OD-H (95:5 hexane:IPA, flow rate 1.0 mL min^{-1}, 211 nm, 30 °C) t_R (2*S*,5*S*): 19.0 min, t_R (2*R*,5*R*): 22.3 min, >99% ee; ^{1}H NMR (500 MHz, $CDCl_3$) δ_H: 1.66-1.71 (3H, m, CHC*H*$_3$), 2.34 (3H, s, ArC*H*$_3$), 4.76 (1H, dq, *J* 6.7, 1.1, C(5)*H*), 5.44 (1H, td, *J* 6.8, 1.7, C*H*), 5.83-5.91 (1H, m, C*H*), 6.47 (1H, d, *J* 1.2, C(2)*H*), 7.05-7.15 (2H, m, SO_2ArC(3)*H*), 7.20-7.31 (3H, m, ArC*H*), 7.31-7.40 (2H, m, ArC*H*), 7.42-7.54 (2H, m, SO_2ArC(2)*H*); ^{13}C{^{1}H} NMR (125 MHz, $CDCl_3$) δ_C: 17.9 (CHC*H*$_3$), 21.7 (Ar*C*H$_3$), 78.0 (*C*(5)), 90.8 (*C*(2)), 123.3 (*C*HCHCH$_3$), 127.3 (C(5)Ar*C*(2)), 128.0 (Ar*C*), 128.2 (Ar*C*), 128.3 (SO_2Ar*C*(2)), 128.4 (Ar*C*), 128.6 (Ar*C*), 129.4 (SO_2Ar*C*(3)), 130.0 (Ar*C*), 133.5 (*C*HCH$_3$), 134.8 (C(2)Ar*C*(1)), 136.6 (SO_2Ar*C*(1)), 145.6 (SO_2Ar*C*(4)), 169.0 (*C*(4)); *m/z* (NSI^+) 358 ($[M+H]^+$, 100%) HMRS (NSI^+) $C_{19}H_{20}NSO_4$ $[M+H]^+$ found 358.1105, requires 358.1108 (–0.7 ppm).

Selected data for the *syn* diastereoisomer **24b**: colourless oil, $[\alpha]_D^{22}$+2.6 (*c* 0.1, $CHCl_3$); Chiral HPLC analysis, Chiralcel OD-H (95:5 hexane:IPA, flow rate 1.0 mL min^{-1}, 211 nm, 30 °C), t_R (2*S*,5*R*): 21.0 min, t_R (2*R*,5*S*): 29.9 min, >99% ee; ^{1}H NMR (500 MHz, $CDCl_3$) δ_H: 2.42 (3H, s, C*H*$_3$), 4.80-4.87 (1H, m, C(5)*H*), 5.47-5.57 (1H, m, C*H*), 5.87-5.99 (1H, m, C*H*), 6.45 (1H, d, *J* 1.2, C(2)*H*); ^{13}C{^{1}H} NMR (125 MHz, $CDCl_3$)^{13}C{^{1}H} NMR (125 MHz, $CDCl_3$) δ_C: 78.8 (*C*(5)), 90.7 (*C*(2)), 123.7 (*C*HCHCH$_3$), (SO_2Ar*C*(3)), 130.2 (Ar*C*), 134.0 (*C*HCH$_3$), 135.1 (C(2)Ar*C*(1)), 136.5 (SO_2Ar*C*(1)), 145.4 (SO_2Ar*C*(4)), 168.9 (*C*(4)).

(2*R*,5*R*)-2-Phenyl-3-tosyl-5-(4-(trifluoromethyl)phenyl)oxazolidin-4-one (*anti*-25a) and (2*S*,5*R*)-2-phenyl-3-tosyl-5-(4-(trifluoromethyl)phenyl)oxazolidin-4-one (*syn*-25b)

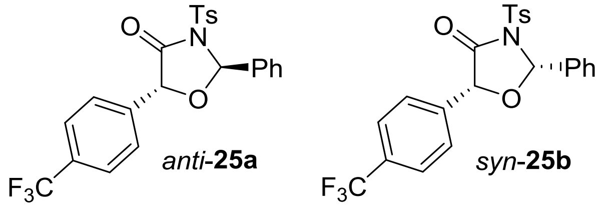

Following *General Procedure 3*, homoanhydride **S9** (117.1 mg, 0.3 mmol), cesium carbonate (131.5 mg, 0.4 mmol), oxaziridine **(±)-5** (55.1 mg, 0.2 mmol) and (2*S*,3*R*)-HyperBTM **3** (6.2 mg, 0.02 mmol) were stirred at –78 °C to room temperature for 16 h to give the crude product (49:51 $dr_{anti:syn}$). Purification yielded the product **25** as a mixture of diastereoisomers (48:52 $dr_{anti:syn}$) as a white solid (62.9 mg, 68%).

Data for the *anti* diastereoisomer **25a**: colourless oil, $[\alpha]_D^{22}$ +69.0 (*c* 0.1, $CHCl_3$); ν_{max} (neat) 1749 (C=O), 1489 (C-F); Chiral HPLC analysis, Chiralcel OD-H (90:10 hexane:IPA, flow rate 1.0 mL min^{-1}, 211 nm, 30 °C) t_R (2*S*,5*S*): 19.5 min, t_R (2*R*,5*R*): 22.1 min, 43% ee; ^1H NMR (500 MHz, $CDCl_3$) δ_H: 2.41 (3H, s, C*H*3), 5.48 (1H, s, C(5)*H*), 6.72 (1H, d, *J* 1.3, C(2)*H*), 7.12-7.23 (2H, m, SO_2ArC(3)*H*), 7.32-7.45 (4H, m, ArC*H*), 7.49-7.53 (2H, m, SO_2ArC(2)*H*), 7.53-7.57 (3H, m, ArC*H*), 7.64-7.66 (2H, m, ArC*H*); $^{13}C\{^1H\}$ NMR (125 MHz, $CDCl_3$) δ_C: 21.9 (*C*H3), 77.9 (*C*(5)), 91.5 (*C*(2)), 125.9 (q, $^3J_{CF}$ 4.0, C(5)Ar*C*(3)), 126.6 (C(2)Ar*C*(2)), 126.9 (q, $^1J_{CF}$ 272.4, *C*F3), 127.5, 128.4 (C(5)Ar*C*(2)), 129.0 (SO_2Ar*C*(2)), 129.6 (Ar*C*), 130.5 (Ar*C*), 131.4 (q, $^2J_{CF}$ 32.5, C(5)Ar*C*(4)), 134.6 (C(2)Ar*C*(1)), 136.3 (SO_2Ar*C*(1)), 138.2 (C_{ipso}), 146.0 (SO_2Ar*C*(4)), 168.8 (*C*(4)); ^{19}F NMR (376 MHz, $CDCl_3$) δ_F: –62.7 (Ar*F*); *m/z* (NSI^+) 461 ($[M+H]^+$, 40%); HMRS (NSI^+) $C_{23}H_{19}F_3NSO_4$ $[M+H]^+$ found 462.0981, requires 462.0976 (–1.2 ppm).

Selected Data for the *syn* diastereoisomer **25b**: colourless oil, Chiral HPLC analysis, Chiralcel OD-H (90:10 hexane:IPA, flow rate 1.0 mL min^{-1}, 211 nm, 30 °C) t_R (2*S*,5*R*): 15.5 min, t_R (2*R*,5*S*): 35.3 min, 36% ee; ^1H NMR (500 MHz, $CDCl_3$) δ_H: 2.38 (3H, s, C*H*3), 5.48 (1H, s, C(5)*H*), 6.62 (1H, d, *J* 1.3, C(2)*H*), 7.15-7.17 (2H, m, SO_2ArC(3)*H*), 7.31-7.35 (2H, m, ArC*H*), 7.35-7.40 (2H, m, Ar*H*), 7.40-7.44 (2H, m, ArC*H*), 7.52-7.58 (2H, m, ArC*H*), 7.58-7.63 (2H, m, ArC*H*); $^{13}C\{^1H\}$ NMR (125 MHz, $CDCl_3$) δ_C: 21.9 (*C*H3), 78.1 (*C*(5)), 91.2 (*C*(2)), 125.6 (q, $^3J_{CF}$ 4.0, C(5)Ar*C*(3)), 126.4 (C(2)Ar*C*(2)), 128.3 (C(5)Ar*C*(2)), 128.8 (SO_2Ar*C*(2)), 129.7 (Ar*C*), 130.7 (Ar*C*); ^{19}F NMR (376 MHz, $CDCl_3$) δ_F: –62.7 (Ar*F*).

Using Excess (±)-Oxaziridine 5

Oxaziridine (±)-**5** (110 mg, 0.400 mmol) and HyperBTM **3** (6.2 mg, 0.020 mmol) were added to a solution of homoanhydride **7** (50.9 mg, 0.200 mmol) and Cs_2CO_3 (98.0 mg, 0.300 mmol) in CH_2Cl_2 (1 mL) at −78 °C. The reaction was allowed to warm to room temperature over 16 h before being quenched with 1 M HCl (5 mL). The solution was extracted with CH_2Cl_2 (2×5 mL), the combined organics were dried ($MgSO_4$), filtered and concentrated *in vacuo*. The crude product was purified by Biotage® Isolera™ 4 [SNAP KP-Sil 10 g, 36 mL min^{-1}, hexane : Et_2O (95 : 5 1 CV, 95 : 5 to 70 : 30 10 CV, 70 : 30 9 CV)] to give oxazolidin-4-one **6** as a white solid (75:25 $dr_{anti:syn}$, 56 mg, 71%) and oxaziridine **5** as a white solid (32 mg, 29%), with data in accordance with that reported above.

Chiral HPLC analysis *anti*-**6a**, Chiralcel AD-H (95:5 hexane:IPA, flow rate 1.5 mL min^{-1}, 211 nm, 40 °C)) t_R (2*R*,5*R*): 27.1 min, t_R (2*S*,5*S*): 53.9 min, >99% ee.

Chiral HPLC analysis *syn*-**6b**, Chiralcel AD-H (95:5 hexane:IPA, flow rate 1.5 mL min^{-1}, 211 nm, 40 °C) t_R (2*S*,5*R*): 25.1 min, t_R (2*R*,5*S*): 30.4 min >99% ee.

Chiral HPLC analysis **5**, Chiralcel OD-H (95:5 hexane:IPA, flow rate 1.0 mL min^{-1}, 211 nm, 30 °C) t_R (*R*,*R*): 10.2 min, t_R (*S*,*S*): 14.7 min, 42% ee.

Using (R,R)-Oxaziridine 5

(2*R*,5*R*)-2,5-Diphenyl-3-tosyloxazolidin-4-one (*anti*-6a)

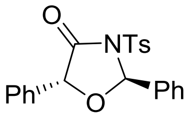

6a

Following *General Procedure 3*, homoanhydride **7** (152 mg, 0.6 mmol), Cs_2CO_3 (263 mg, 0.8 mmol), oxaziridine (*R*,*R*)-**5** (110 mg, 0.4 mmol) and (2*S*,3*R*)-HyperBTM **3** (12 mg, 0.04 mmol) were stirred at −78 °C and warmed to room temperature over 16 h to give the crude product (93:7 $dr_{anti:syn}$). Purification yielded oxazolidin-4-one **6a** (95:5 $dr_{anti:syn}$) as a white solid (128 mg, 81%). mp 143-144 °C, $[\alpha]_D^{22}$ +56.8 (*c* 0.85, $CHCl_3$), ν_{max} (neat)/cm^{-1} 1745 (C=O), 1363 (C-N), 1172 (R-SO_2N), 1087 (C-O); Chiral HPLC analysis, AD-H (95:5 hexane:IPA, flow rate 1.5 mL min^{-1}, 211 nm, 40 °C), t_R (2*R*,5*R*): 27.1 min, t_R (2*S*,5*S*): 53.9 min, >99% ee; ^{1}H NMR (400 MHz, $CDCl_3$) δ_H: 2.42 (3H, s, ArC*H*$_3$), 5.44 (1H, d, *J* 1.2, C(5)*H*), 6.72 (1H, d, *J* 1.3, C(2)*H*), 7.19 (2H, dt, *J* 8.1, 0.8, 2×SO_2ArC(3)*H*), 7.35-7.43 (9H, m, Ar*H*), 7.44-7.49 (1H, m, ArC(4)*H*), 7.53 (2H, d, *J* 8.4, 2×SO_2ArC(2)*H*); ^{13}C{^{1}H} NMR (125 MHz, $CDCl_3$) δ_C: 21.7 (Ar*C*H$_3$), 78.6 (*C*(2)H), 91.2 (*C*(5)H), 126.4 (C(2)Ar*C*(2)H), 127.4 (SO_2Ar*C*(2)H), 128.2 (C(2)Ar*C*(3)H), 128.7 (C(5)Ar*C*(3)H), 128.8 (SO_2Ar*C*(3)H), 129.1 (Ar*C*(4)H), 129.4 (C(5)Ar*C*(2)H), 130.1 (Ar*C*(4)H), 134.3 (SO_2Ar*C*(1)), 134.7 (C(5)Ar*C*(1)), 136.5 (SO_2Ar*C*(4)), 145.6 (C(2)Ar*C*(1)), 168.7 (*C*(4)).

X-Ray Structure (**6a**)

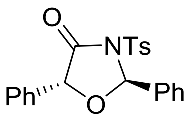

6a

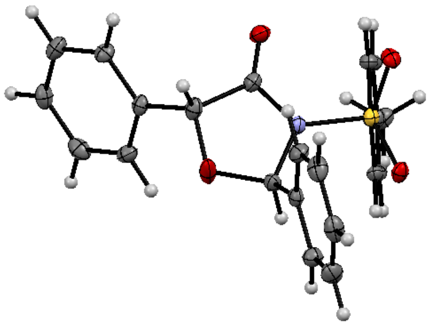

CCDC 1046834

Formula:$C_{22}H_{19}NO_4S$
Unit Cell Parameters: a 9.474(9) b 5.904(5) c 17.219(13) P21

(2*R*,5*S*)-2,5-Diphenyl-3-tosyloxazolidin-4-one (*syn*-6b)

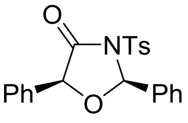

6b

Following a slightly modified version of *General Procedure 3*, homoanhydride **7** (76 mg, 0.3 mmol), Cs_2CO_3 (131 mg, 0.4 mmol), oxaziridine (*R*,*R*)-**5** (55 mg, 0.2 mmol) and (2*R*,3*S*)-HyperBTM **3** (6 mg, 0.02 mmol) were stirred at 0 °C to room temperature for 16 h to give the crude product (80:20 $dr_{syn:anti}$). Purification yielded the oxazolidin-4-one **6b** (80:20 $dr_{syn:anti}$) as a white solid (75 mg, 95%). mp 177-179 °C, $[\alpha]_D^{22}$ +38.5 (*c* 0.55, $CHCl_3$), ν_{max} (neat)/cm^{-1} 1757 (C=O), 1377 (C-N), 1163, (R-SO_2N), 1085 (C-O); Chiral HPLC analysis, AD-H (95:5 hexane:IPA, flow rate 1.5 mL min^{-1}, 211 nm, 40 °C), t_R (2*S*,5*R*): 25.1 min, t_R (2R,5*S*): 30.4 min, >98% ee; ^{1}H NMR (400 MHz, $CDCl_3$) δ_H: 2.40 (3H, s, ArC*H*$_3$), 5.41-5.42 (1H, m, C(5)*H*), 6.60 (1H, d, *J* 1.4, C(2)*H*), 7.14-7.20 (2H, m, Ar*H*), 7.35-7.42 (10H, m Ar*H*), 7.47 (2H, *J* 8.4, Ar*H*); ^{13}C{^{1}H} NMR (101 MHz, $CDCl_3$) δ_C: 21.7 (Ar*C*H$_3$), 78.9 (*C*(2)H), 90.8 (*C*(5)H), 126.2 (2×Ar*C*H), 128.1 (2×Ar*C*H), 128.3 (2×Ar*C*H), 128.5 (2×Ar*C*H), 128.5 (2×Ar*C*H), 128.8 (Ar*C*H), 129.4 (2×Ar*C*H), 130.3(Ar*C*H), 133.9 (*N*SO$_2$Ar*C*(1)), 135.0 (C(3)Ar*C*(1)), 136.3 (Ar*C*(4)CH$_3$), 145.5 (C(2)Ar*C*(1)), 168.5 (*C*(4)).

(2*R*,5*R*)-5-(4-Fluorophenyl)-2-phenyl-3-tosyloxazolidin-4-one (*anti*-17a)

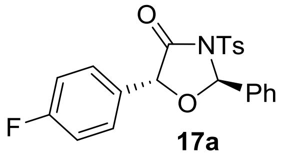

17a

Following *General Procedure 3*, homoanhydride **S1** (174 mg, 0.6 mmol), Cs_2CO_3 (263 mg, 0.8 mmol), oxaziridine (*R*,*R*)-**5** (110 mg, 0.4 mmol) and (2*S*,3*R*)-HyperBTM **3** (12 mg, 0.04 mmol) were stirred at −78 °C to room temperature for 16 h to give the crude product (94:6 $dr_{anti:syn}$). Purification yielded the oxazolidin-4-one **17a** (95:5 $dr_{anti:syn}$) as a white solid (128 mg, 81%). mp 131-132 °C, $[\alpha]_D^{22}$ +50.7 (*c* 1.75, $CHCl_3$), ν_{max} (neat)/cm^{-1} 1743 (C=O), 1508, 1367 (C-N), 1174 (R-SO_2N), 1087 (C-O); Chiral HPLC analysis, OD-H (90:10 hexane:IPA, flow rate 1.0 mL min^{-1}, 211 nm, 30 °C), t_R (2*R*,5*R*): 13.5 min, t_R (2*S*,5*S*): 14.9 min, >99% ee; ^{1}H NMR (400 MHz, $CDCl_3$) δ_H: 2.42 (3H, s, ArC*H*$_3$), 5.41 (1H, d, *J* 1.0, C(5)*H*), 6.71 (1H, d, *J* 1.3, C(2)*H*), 7.02-7.13 (2H, m, Ar*H*), 7.17-7.23 (2H, m, Ar*H*), 7.34-7.41 (6H, m, Ar*H*), 7.44-7.51 (1H, m, Ar*H*), 7.54 (2H, d, *J* 8.4, Ar*H*); ^{13}C{^{1}H} NMR (100 MHz, $CDCl_3$) δ_C: 21.7 (ArCH$_3$), 77.9 (*C*(2)H), 91.1 (*C*(5)H), 115.8 (d, $^2J_{CF}$ 21.9, C(5)Ar*C*(3)H), 127.3 (C(2)Ar*C*(4)H), 128.2 (C(2)Ar*C*(2)H), 128.3 (Ar*C*(4)H), 128.7 (SO$_2$Ar*C*(2)H), 129.4 (SO$_2$Ar*C*(3)H), 130.1 (d, $^4J_{CF}$ 3.1, C(5)Ar*C*(1)), 130.2 (ArC(4), 134.6 (SO$_2$Ar*C*(1)), 136.3

(SO_2Ar*C*(4)CH_3), 145.7 (C(3)Ar*C*(1)), 163.1 (d, $^1J_{CF}$ 248.2, C(5)Ar*C*F), 168.5 (*C*(4)); ^{19}F NMR (282 MHz, $CDCl_3$) δ_F −112.3.

(2*R*,5*R*)-5-(4-Methoxyphenyl)-2-phenyl-3-tosyloxazolidin-4-one (*anti*-18a)

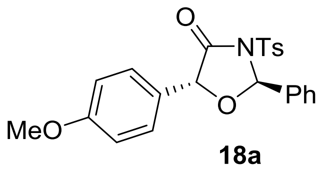

18a

Following *General Procedure 3*, homoanhydride **S2** (188 mg, 0.6 mmol), Cs_2CO_3 (263 mg, 0.8 mmol), oxaziridine (*R*,*R*)-**5** (110 mg, 0.4 mmol) and (2*S*,3*R*)-HyperBTM **3** (12 mg, 0.04 mmol) were stirred at −78 °C to room temperature for 16 h to give the crude product (94:6 $dr_{anti:syn}$). Purification yielded the oxazolidin-4-one **18a** (95:5 $dr_{anti:syn}$) as a white solid (147 mg, 87%). mp 126-127 °C, $[\alpha]_D^{22}$ +65.4 (*c* 0.95, $CHCl_3$), ν_{max} (neat)/cm^{-1} 1741 (C=O), 1361 (C-N), 1172 (R-SO_2N), 1088 (C-O); Chiral HPLC analysis, OD-H (90:10 hexane:IPA, flow rate 1.0 mL min^{-1}, 211 nm, 30 °C), t_R (2*R*,5*R*): 24.2 min, t_R (2*S*,5*S*): 26.0 min, >99% ee; ^{1}H NMR (400 MHz, $CDCl_3$) δ_H: 2.43 (3H, s, ArC*H*$_3$), 3.82 (3H, s, ArOC*H*$_3$), 5.39-5.40 (1H, m, C(5)*H*), 6.71 (1H, d, *J* 1.2, C(2)*H*), 6.91 (2H, d, *J* 8.8, Ar*H*), 7.22 (2H, d, *J* 8.1, Ar*H*), 7.26-7.30 (2H, m, Ar*H*), 7.40-7.42 (4H, m, Ar*H*), 7.44-7.50 (1H, m , Ar*H*), 7.57 (2H, d, *J* 8.4, Ar*H*); ^{13}C{^{1}H} NMR (125 MHz, $CDCl_3$) δ_C: 21.7 (Ar*C*H$_3$), 55.3 (ArO*C*H$_3$), 78.5 (*C*(5)H), 90.9 (*C*(2)H), 114.3 (C(5)Ar*C*(2)H), 126.4 (C(5)Ar*C*(1)H), 127.3 (C(2)Ar*C*(2)H), 128.1 (SO_2Ar*C*(2)H), 128.2 (Ar*C*H), 128.7 (Ar*C*H), 129.4 (SO_2Ar*C*(3)H), 130.1 (Ar*C*H), 134.7 (SO_2Ar*C*(1)), 136.6 (C(2)Ar*C*(1)), 145.6 (SO_2Ar*C*(4)), 160.3 (C(5)Ar*C*(4)), 169.0 (*C*(4)).

(2*R*,5*R*)-5-(Naphthalen-2-yl)-2-phenyl-3-tosyloxazolidin-4-one (*anti*-19a)

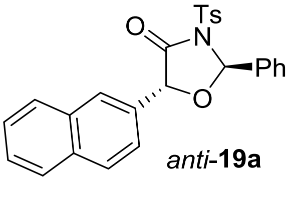

anti-**19a**

Following *General Procedure 3* homoanhydride **S3** (106 mg, 0.300 mmol), cesium carbonate (130 mg, 0.400 mmol), oxaziridine (*R*,*R*)-**5** (55.1 mg, 0.200 mmol) and (2*S*,3*R*)-HyperBTM **3** (6.2 mg, 0.020 mmol) were stirred at −78 °C to room temperature for 16 h to give the crude product (>95:5$dr_{anti:syn}$). Purification by Biotage® Isolera™ 4 [SNAP KP-Sil 10 g, 36 mL min^{-1}, hexane : Et_2O (95 : 5 1 CV, 95 : 5 to 70 : 30 10 CV, 70 : 30 9 CV)] yielded oxazolidin-4-one **19a** as a white solid (53.0 mg, 60%). mp 163-165 °C (*dec.*); $[\alpha]_D^{20}$+74.4 (*c* 1.0, $CHCl_3$); Chiral HPLC analysis, Chiralcel AD-H (90:10 hexane:IPA, flow rate 1.0 mL min^{-1}, 211 nm, 40 °C) t_R (2*R*,5*R*): 33.2 min, t_R (2*S*,5*S*):

84.8 min, >99% ee; ν_{max} (film) 1749, 1597, 1371, 1231, 1173, 1088; ^{1}H NMR (400 MHz, $CDCl_3$) δ_H: 2.39 (3H, s, ArC*H*$_3$), 5.60 (1H, s, C(5)*H*), 6.77 (1H, d, *J* 1.2, C(2)*H*), 7.18 (2H, d, *J* 8.5, SO_2ArC(2)*H*), 7.39-7.47 (6H, m, Ar*H*), 7.49-7.53 (2H, m, Ar*H*), 7.56 (2H, d, *J* 8.3, SO_2ArC(3)*H*), 7.79-7.86 (4H, m, Ar*H*); ^{13}C{^{1}H} NMR (100 MHz, $CDCl_3$) δ_C: 21.8 (Ar*C*H$_3$), 78.9 (*C*(5)), 91.4 (*C*(2)), 123.7 (Ar*C*), 126.1 (Ar*C*), 126.6 (Ar*C*), 126.8 (Ar*C*), 127.5 (C(2)Ar*C*(2)), 127.9 (Ar*C*), 128.3 (Ar*C*), 128.4 (SO_2Ar*C*(2)), 128.9 (Ar*C*), 129.0 (Ar*C*), 129.6 (SO_2Ar*C*(3)), 130.3 (Ar*C*), 131.7 (C(5)Ar*C*(1)), 133.1 (C_{ipso}), 133.6 (C_{ipso}), 134.8 (SO_2Ar*C*(1)), 136.7 (C(2)Ar*C*(1)), 145.8 (SO_2Ar*C*(4)), 168.8 (*C*(4)).

(2*R*,5*R*)-2-Phenyl-5-(*m*-tolyl)-3-tosyloxazolidin-4-one (*anti*-21a)

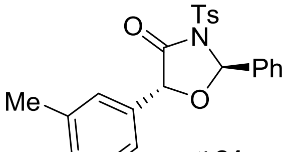

anti-**21a**

Following *General Procedure 3*, homoanhydride **S5** (141 mg, 0.500 mmol), cesium carbonate (217 mg, 0.666 mmol), oxaziridine (*R*,*R*)-**5** (92.0 mg, 0.333 mmol) and (2*S*,3*R*)-HyperBTM **3** (10.3 mg, 0.0333 mmol) were stirred at –78 °C to room temperature for 16 h to give the crude product (>95:5 $dr_{anti:syn}$). Purification by Biotage® Isolera™ 4 [SNAP KP-Sil 10 g, 36 mL min^{-1}, hexane : Et_2O (95 : 5 1 CV, 95 : 5 to 60 : 40 10 CV, 60 : 40 5 CV)] yielded oxazolidin-4-one **21a** as a colourless oil (117 mg, 87%). $[\alpha]_D^{20}$ +33.6 (*c* 2.0, $CHCl_3$); Chiral HPLC analysis, Chiralpak AD-H (90:10 hexane:IPA, flow rate 1.0 mL min^{-1}, 211 nm, 40 °C) t_R (2*R*,5*R*): 18.9 min, t_R (2*S*,5*S*): 42.0 min, >99% ee; ν_{max} (film) 1751 (C=O), 1597, 1371 (C-N), 1227, 1173 (R-SO_2N), 1088 (C-O); ^{1}H NMR (400 MHz, $CDCl_3$) δ_H: 2.25 (3H, s, C(5)ArC*H*$_3$), 2.32 (3H, s, SO_2ArC*H*$_3$), 5.30 (1H, s, C(5)*H*), 6.62 (1H, d, *J* 1.2, C(2)*H*), 7.03-7.15 (5H, m, Ar*H*), 7.15-7.19 (1H, m, Ar*H*), 7.27-7.39 (5H, m, Ar*H*), 7.46 (2H, d, *J* 8.3, SO_2ArC(3)*H*); ^{13}C{^{1}H} NMR (100 MHz, $CDCl_3$) δ_C: 21.5 (C(5)Ar*C*H$_3$), 21.8 (SO_2Ar*C*H$_3$), 78.8 (*C*(5)), 91.3 (*C*(2)), 123.8 (Ar*C*), 127.1 (Ar*C*), 127.5 (Ar*C*), 128.3 (C(2)Ar*C*(2)), 128.8 (SO_2Ar*C*(2)), 128.8 (Ar*C*), 129.6 (SO_2Ar*C*(3)), 130.1 (Ar*C*), 130.2 (Ar*C*), 134.4 (C(5)Ar*C*(1)), 134.9 (SO_2Ar*C*(1)), 136.7 (C(2)Ar*C*(1)), 138.7 (C(5)Ar*C*(2)CH_3), 145.7 (SO_2Ar*C*(4)), 169.0 (*C*(4)).

(2*R*,5*R*)-2-Phenyl-5-(*o*-tolyl)-3-tosyloxazolidin-4-one (*anti*-22a)

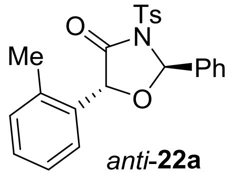

anti-**22a**

Following *General Procedure 3*, homoanhydride **S6** (84.7 mg, 0.300 mmol), cesium carbonate (130 mg, 0.400 mmol), oxaziridine (*R*,*R*)-**5** (55.1 mg, 0.200 mmol) and (2*S*,3*R*)-HyperBTM **3** (6.2 mg, 0.020 mmol) were stirred at –78 °C to room temperature for 16 h to give the crude product (>95:5 $dr_{anti:syn}$). Purification by Biotage® Isolera™ 4 [SNAP KP-Sil 10 g, 36 mL min^{-1}, hexane : Et_2O (95 : 5 1 CV, 95 : 5 to 70 : 30 10 CV, 70 : 30 9 CV)] yielded oxazolidin-4-one **22a** as a white solid (77 mg, 95%). mp 104-106 °C; $[\alpha]_D^{20}$+42.7 (*c* 1.1, $CHCl_3$); Chiral HPLC analysis, Chiralpak AD-H (90:10 hexane:IPA, flow rate 1.0 mL min^{-1}, 211 nm, 40 °C) t_R (2*R*,5*R*): 21.1 min, t_R (2*S*,5*S*): 24.4 min, >99% ee; ν_{max} (film) 1751 (C=O), 1597, 1371 (C-N), 1230, 1175 (R-SO_2N), 1088 (C-O); ^{1}H NMR (400 MHz, $CDCl_3$) δ_H: 2.33 (3H, s, C(5)ArC*H*$_3$), 2.45 (3H, s, SO_2ArC*H*$_3$), 5.63 (1H, d, *J* 1.2, C(5)*H*), 6.73 (1H, d, *J* 1.2, C(2)*H*), 7.18-7.30 (6H, m, Ar*H*), 7.41-7.51 (5H, m, Ar*H*), 7.64 (2H, d, *J* 8.3, SO_2ArC(3)*H*); ^{13}C{^{1}H} NMR (100 MHz, $CDCl_3$) δ_C: 19.3 (C(5)Ar*C*H$_3$), 21.9 (SO_2Ar*C*H$_3$), 77.4 (*C*(5)), 91.0 (*C*(2)), 126.4 (Ar*C*), 127.3 (C(2)Ar*C*(2)), 127.6 (Ar*C*), 128.5 (SO_2Ar*C*(2)), 128.9 (2 Ar*C*), 129.5 (Ar*C*), 129.6 (SO_2Ar*C*(3)), 130.2 (Ar*C*), 131.2 (Ar*C*), 132.8 (C(5)Ar*C*(1)), 134.8 (SO_2Ar*C*(1)), 136.9 (C(2)Ar*C*(1)), 137.3 (C(5)Ar*C*(2)CH$_3$), 145.8 (SO_2Ar*C*(4)), 169.1 (*C*(4)); HMRS (NSI^+) $C_{23}H_{22}NSO_4^+$ $[M+H]^+$ found 408.1258, requires 408.1264; (–1.5 ppm).

(2*R*,5*R*)-5-((*E*)-But-1-en-1-yl)-2-phenyl-3-tosyloxazolidin-4-one (*anti*-30a)

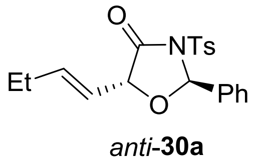

anti-**30a**

Following *General Procedure 3*, homoanhydride **S10** (126 mg, 0.6 mmol), Cs_2CO_3 (263 mg, 0.8 mmol), oxaziridine **(*R*,*R*)-5** (110 mg, 0.4 mmol) and (2*S*,3*R*)-HyperBTM **3** (12 mg, 0.04 mmol) were stirred at –78 °C to room temperature for 16 h to give the crude product (94:6 $dr_{anti:syn}$). Purification yielded the oxazolidin-4-one **30a** (95:5 $dr_{anti:syn}$) as a colourless oil (106 mg, 71%). $[\alpha]_D^{22}$ +43.3 (*c* 0.75, $CHCl_3$), ν_{max} (neat)/cm^{-1} 1743 (C=O), 1514, 1361 (C-N), 1174 (R-SO_2N), 1087 (C-O); Chiral HPLC analysis, AD-H (90:10 hexane:IPA, flow rate 1.0 mL min^{-1}, 211 nm, 30 °C), t_R (2*R*,5*R*): 12.1 min, t_R (2*S*,5*S*): 27.7 min, >99% ee; ^{1}H NMR (300 MHz, $CDCl_3$) δ_H: 0.92 (3H, t, *J* 7.4, CH$_2$C*H*$_3$), 1.98-2.07 (2H, m, C*H*$_2$CH$_3$), 2.33 (3H, s, ArC*H*$_3$), 4.78 (1H, dt, *J* 6.5, 1.1, C(5)*H*), 5.38 (1H, dd, *J* 15.5, 6.5, EtC*H*=CHCH), 5.87 (1H, dtd, *J* 15.5, 6.2, 1.3, EtCH=C*H*CH), 6.47 (1H, d, *J* 1.2, C(2)*H*),

7.09-7.10 (2H, m, Ar*H*), 7.21-7.37 (5H, m, Ar*H*), 7.45 (2H, d, *J* 8.4, Ar*H*); $^{13}C\{^1H\}$ NMR (101 MHz, $CDCl_3$) δ_C: 12.7 (CH_2CH_3), 21.7 (ArCH_3), 26.2 (CH_2CH_3), 78.0 (*C*(2)H), 90.7 (*C*(5)H), 121.0 (Et*C*H=CH), 127.2(2×Ar*C*H), 128.2 (2×Ar*C*H), 128.6 (2×Ar*C*H), 129.4 (2×Ar*C*H), 130.0 (Ar*C*H), 134.8 (Ar*C*), 136.5 (ArC), 139.9 (EtCH=*C*H), 145.5 (C(2)Ar*C*(1)), 169.0 (*C*(4)=O); *m/z* (NSI^+) 371 ($[M+H]^+$, 100%) HMRS (NSI^+) $C_{20}H_{22}NO_4S$ $[M+H]^+$ found 372.1264, requires 372.1264 (+0.0 ppm).

(2*R*,5*S*)-2-Phenyl-5-(thiophen-2-yl)-3-tosyloxazolidin-4-one (*anti*-31a)

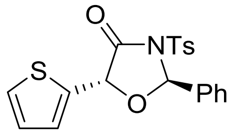

31a

Following *General Procedure 3*, homoanhydride **S11** (190 mg, 0.6 mmol), Cs_2CO_3 (263 mg, 0.8 mmol), oxaziridine **(*R*,*R*)-5** (110 mg, 0.4 mmol) and (2*S*,3*R*)-HyperBTM **3** (12 mg, 0.04 mmol) were stirred at −78 °C to room temperature for 16 h to give the crude product (94:6 $dr_{anti:syn}$). Purification yielded the oxazolidin-4-one **31a** (95:5 $dr_{anti:syn}$) as a yellow oil (97 mg, 61%). $[\alpha]_D^{22}$ +76.3 (*c* 1.35, $CHCl_3$), ν_{max} (neat)/cm^{-1} 1751 (C=O), 1516, 1377 (C-N), 1174 (R-SO_2N), 1087 (C-O); Chiral HPLC analysis, AD-H (80:20 hexane:IPA, flow rate 1.0 mL min^{-1}, 211 nm, 30 °C), t_R (2*R*,5*R*): 17.1 min, t_R (2*S*,5*S*): 33.5 min, >99% ee; ^1H NMR (500 MHz, $CDCl_3$) δ_H: 2.43 (3H, s, ArCH_3), 5.67 (1H, s, C(5)*H*), 6.67 (1H, *J* 1.1, C(2)*H*), 7.02 (1H, dd, *J* 5.1, 3.5, HetArC*H*), 7.11 (1H, d, *J* 3.6, HetArC*H*), 7.22 (2H, d, *J* 8.1, SO_2ArC(3)*H*), 7.37 (1H, dd, *J* 5.0, 1.2, HetArC*H*), 7.39-7.41 (4H, m, ArC*H*), 7.44-7.48 (1H, m, Ar*H*), 7.57 (2H, d, *J* 8.3, SO_2ArC(2)*H*); ^{13}C NMR (100 MHz, $CDCl_3$) δ 21.7 (ArCH_3), 75.2 (*C*(2)H), 90.9 (*C*(5)H), 126.8 (Ar*C*H), 127.1 (Ar*C*H), 127.2 (Ar*C*H), 127.4 (2×Ar*C*H), 128.2 (2×Ar*C*H), 128.7 (2×Ar*C*H), 129.5 (2×Ar*C*H), 130.2 (Ar*C*H), 134.6 (SO_2ArC(1)), 136.1 (Ar*C*(CH_3), 136.3 (C(2)Ar*C*(1)H), 145.7 (C(5)Ar*C*(2)), 167.5 (*C*(4)=O); *m/z* (NSI^+) 399 ($[M+NH_4]^+$, 100%) HMRS (NSI^+) $C_{20}H_{21}N_2O_4S_2$ $[M+NH_4]^+$ found 417.0935, requires 417.0937 (–0.5 ppm).

(2*R*,5*R*)-2-Phenyl-3-tosyl-5-(4-(trifluoromethyl)phenyl)oxazolidin-4-one (*anti*-25a) and (2*S*,5*R*)-2-phenyl-3-tosyl-5-(4-(trifluoromethyl)phenyl)oxazolidin-4-one (*syn*-25b)

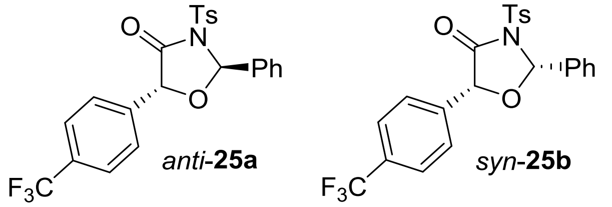

Following *General Procedure 3*, homoanhydride **S9** (117 mg, 0.300 mmol), cesium carbonate (130 mg, 0.400 mmol), oxaziridine (*R*,*R*)-**5** (55.1 mg, 0.200 mmol) and (2*S*,3*R*)-HyperBTM **3** (6.2 mg, 0.020 mmol) were stirred at –78 °C to room temperature for 16 h to give the crude product (60:40 $dr_{anti:syn}$). Purification by Biotage® Isolera™ 4 [SNAP KP-Sil 10 g, 36 mL min^{-1}, hexane : Et_2O (95 : 5 1 CV, 95 : 5 to 70 : 30 10 CV, 70 : 30 9 CV)] yielded the product **25** as a mixture of diastereoisomers (60:40 $dr_{anti:syn}$) as a white solid (45 mg, 49%). mp 124-126 °C; $[\alpha]_D^{20}$ +48.1 (*c* 1.1, $CHCl_3$); ν_{max} (film) 1749 (C=O), 1489 (C-F); HMRS (NSI^+) $C_{23}H_{19}F_3NSO_4$ $[M+H]^+$ found 462.0981, requires 462.0976 (–1.2 ppm).

Data for the *anti* diastereoisomer **25a**: Chiral HPLC analysis, Chiralcel OD-H (90:10 hexane:IPA, flow rate 1.0 mL min^{-1}, 211 nm, 30 °C) t_R (2*S*,5*S*): 19.5 min, t_R (2*R*,5*R*): 25.2 min, >99% ee; ^{1}H NMR (400 MHz, $CDCl_3$) δ_H: 2.41 (3H, s, CH_3), 5.48 (1H, s, C(5)*H*), 6.72 (1H, d, *J* 1.3, C(2)*H*), 7.12-7.23 (2H, m, SO_2ArC(3)*H*), 7.32-7.45 (4H, m, ArC*H*), 7.49-7.53 (2H, m, SO_2ArC(2)*H*), 7.53-7.57 (3H, m, ArC*H*), 7.64-7.66 (2H, m, ArC*H*); $^{13}C\{^1H\}$ NMR (125 MHz, $CDCl_3$) δ_C: 21.9 (*C*H_3), 77.9 (*C*(5)), 91.5 (*C*(2)), 125.9 (q, $^3J_{CF}$ 4.0, C(5)Ar*C*(3)), 126.6 (C(2)Ar*C*(2)), 126.9 (q, $^1J_{CF}$ 272.4, *C*F_3), 127.5, 128.4 (C(5)Ar*C*(2)), 129.0 (SO_2Ar*C*(2)), 129.6 (Ar*C*), 130.5 (Ar*C*), 131.4 (q, $^2J_{CF}$ 32.5, C(5)Ar*C*(4)), 134.6 (C(2)Ar*C*(1)), 136.3 (SO_2Ar*C*(1)), 138.2 (C_{ipso}), 146.0 (SO_2Ar*C*(4)), 168.8 (*C*(4)); ^{19}F NMR (376 MHz, $CDCl_3$) δ_F: −62.7 (Ar*F*).

Selected Data for the *syn* diastereoisomer **25b**: Chiral HPLC analysis, Chiralcel OD-H (90:10 hexane:IPA, flow rate 1.0 mL min^{-1}, 211 nm, 30 °C) t_R (2*S*,5*R*): 15.8 min, t_R (2*R*,5*S*): 36.0 min, >99% ee; ^{1}H NMR (400 MHz, $CDCl_3$) δ_H: 2.38 (3H, s, CH_3), 5.48 (1H, s, C(5)*H*), 6.62 (1H, d, *J* 1.3, C(2)*H*), 7.15-7.17 (2H, m, SO_2ArC(3)*H*), 7.31-7.35 (2H, m, ArC*H*), 7.35-7.40 (2H, m, Ar*H*), 7.40-7.44 (2H, m, ArC*H*), 7.52-7.58 (2H, m, ArC*H*), 7.58-7.63 (2H, m, ArC*H*); $^{13}C\{^1H\}$ NMR (125 MHz, $CDCl_3$) δ_C: 21.9 (*C*H_3), 78.1 (*C*(5)), 91.2 (*C*(2)), 125.6 (q, $^3J_{CF}$ 4.0, C(5)Ar*C*(3)), 126.4 (C(2)Ar*C*(2)), 128.3 (C(5)Ar*C*(2)), 128.8 (SO_2Ar*C*(2)), 129.7 (Ar*C*), 130.7 (Ar*C*); ^{19}F NMR (376 MHz, $CDCl_3$) δ_F: −62.7 (Ar*F*).

Product Derivatisations

(*R*)-1-Phenylethane-1,2-diol 16

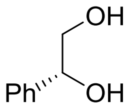

16

Following a procedure developed by Ye *et al.*[11] A solution of oxazolidin-4-one **6** (200 mg, 0.5 mmol, 1 eq) in dry THF (6 mL) at 0 °C, 1 M $LiAlH_4$ in THF (0.5 mL, 1.0 mmol, 2 eq) was added and the reaction stirred at 0 °C allowing to warm to room temperature over 16 h. The reaction was carefully quenched with HCl/MeOH (2.5 M, 5 mL). The resulting mixture was concentrated *in vacuo*. Purification by column chromatography using silica gel (Petrol:EtOAc 70:30) gave **16** as a white solid (40 mg, 60%) with data in accordance with the literature. mp 48-50 °C {lit.[11] 66-68 °C}; $[\alpha]_D^{22}$ –50 (*c* 1.0, $CHCl_3$) {lit.[12] $[\alpha]_D^{22}$ −54.1 (*c* 0.9 $CHCl_3$)}; Chiral HPLC analysis, Chiralcel OD-H (95:5 hexane:IPA, flow rate 1.0 mL min^{-1}, 211 nm, 30 °C) t_R (*R*): 23.3 min, t_R (*S*): 27.2 min, 90% ee; ^1H NMR (500 MHz, $CDCl_3$) δ_H: 2.07 (1H, br. s, O*H*), 2.52 (1H, br. s, O*H*), 3.67 (1H, dd, *J* 11.3, 8.1, C$H_A$$H_B$), 3.76 (1H, t, *J* 2.1, C$H_A$$H_B$), 4.84 (1H, dd, *J* 8.1, 3.6, C*H*), 7.28-7.41 (5H, m, Ar*H*).

Preparation of SmI_2 (~0.1 M in THF)

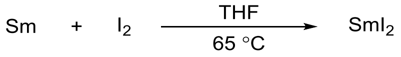

Following a literature procedure,[13] samarium metal[14] (40 mesh, 1.00 g, 6.65 mmol) and iodine (1.25 g, 4.93 mmol) were added to a flame-dried two-necked round-bottomed flask fitted with a reflux condenser under an Ar atmosphere. Degassed (Ar sparge, 30 min) anhydrous THF (50 mL) was added and the resulting solution heated at 65 °C. After *ca*. 12 h the solution had turned deep blue. The solution was allowed to cool to room temperature and was left gently stirring under an Ar atmosphere.

(2*R*,5*R*)-2,5-Diphenyloxazolidin-4-one 32

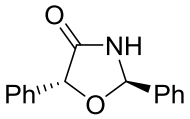

32

Following *General Procedure 4*, oxazolidin-4-one **6a** (50 mg, 0.13 mmol) in THF (1.3 mL) at –78 °C. and SmI_2 (*ca*. 0.1 M in THF, 2.6 mL) added dropwise. The crude product was purified by Biotage® Isolera™ 4 [36 $mLmin^{-1}$, hexane:EtOAc (90:10 to 50:50 8CV)] to give the title compound **32** (28 mg, 90%) as a white solid. mp 128-130 °C; $[\alpha]_D^{20}$ –66.5 (*c* 0.79 in $CHCl_3$); ν_{max} (neat) 3207 (NH), 1725

(C=O), 1373 (C-N); Chiral HPLC analysis, Chiralpak AD-H (90:10 hexane : IPA, flow rate 1.0 mLmin^{-1}, 211 nm, 30 °C) t_R (2*R*,5*R*): 10.6 min, t_R (2*S*,5*S*): 14.3 min, 99% ee; ^{1}H NMR (500 MHz, $CDCl_3$) δ_H: 5.42 (1H, d, *J* 2.2, C(5)*H*), 6.32 (1H, d, *J* 2.2, C(2)*H*), 7.31-7.47 (5H, m, ArC*H*), 7.47-7.60 (5H, m, ArC*H*), 7.77 (1H, s, N*H*); ^{13}C{^{1}H} NMR (125 MHz, $CDCl_3$) δ_C: 78.6 (*C*(5)), 88.0 (*C*(2)), 126.4 (C(5)Ar*C*(2)), 126.6 (C(2)Ar*C*(2)), 128.8 (SO_2Ar*C*(2)), 128.9 (Ar*C*), 129.1 (Ar*C*), 130.1 (Ar*C*), 136.2 (C(5)Ar*C*(1)), 138.4 (C(2)Ar*C*(1)), 173.7 (*C*(4)); *m/z* (NSI$^+$) 257 ([M+Na]$^+$, 100%); HMRS (NSI$^+$) $C_{15}H_{13}NO_2Na$ [M+Na]$^+$ found 262.0838, requires 262.0841 (+1.0 ppm).

(2*R*,5*R*)-5-(4-Fluorophenyl)-2-phenyloxazolidin-4-one 33

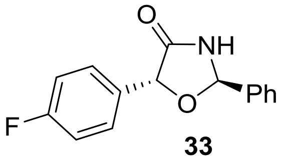

33

Following *General Procedure 4*, oxazolidin-4-one **17a** (53 mg, 0.13 mmol, 1 eq.) in THF (1.3 mL) at –78 °C and SmI_2 (0.1 M in THF, 3 mL, 0.3 mmol, 2.3 eq.) added dropwise. The crude product was purified by Biotage® Isolera™ 4 [36 mLmin^{-1}, hexane:Et_2O (80:20 to 40:60 8CV)] to give the title compound **33** as a white solid (35 mg, >95%, $dr_{anti:syn}$ >95:5 dr). mp 164-165 °C; $[\alpha]_D^{20}$ 60.2 (*c* 0.4, $CHCl_3$); ν_{max} (neat) 3208 (NH), 1714 (C=O), 1508, 1277; Chiral HPLC analysis, Chiralcel AD-H (90:10 hexane:IPA, flow rate 1.0 mL min^{-1}, 211 nm, 30 °C) t_R (2*R*,5*R*): 10.6 min, t_R (2*S*,3*S*): 14.4 min, >99% ee; ^{1}H NMR (300 MHz, $CDCl_3$) δ_H: 5.41 (1H, d, *J* 2.1, C(5)*H*), 6.32 (1H, d, *J* 2.2, C(2)*H*), 7.11 (2H, app t, *J* 8.7, 2×ArH) 7.45-7.53 (7H, m, 7×ArC*H*), 7.66 (1H, br s, N*H*); ^{13}C{^{1}H} NMR (125 MHz, $CDCl_3$) δ_C: 77.9 (*C*(2)), 87.8 (*C*(5)), 115.7 ($^2J_{C-F}$ 21.7, 2×C(5)Ar*C*(3)H), 126.3 (C(2)Ar*C*(2)H), 128.1 ($^3J_{C-F}$ 8.3, 2×C(5)Ar*C*(2)H), 129.0 (2×C(2)Ar*C*(3)H), 129.9 (C(2)Ar*C*(4)H), 131.8 ($^4J_{C-F}$ 3.0 C(5)Ar*C*(1)), 162.9 ($^1J_{C-F}$ 247.2 C(5)Ar*C*(4)F), 173.5 (*C*(4)); ^{19}F{^{1}H} NMR (282 MHz, $CDCl_3$) δ_F: –113.8; *m/z* (NSI$^+$) 257 ([M+H]$^+$, 100%); HMRS (NSI$^+$) $C_{15}H_{13}NO_2F$ [M+H]$^+$ found 258.0925, requires 258.0925 (+0.1 ppm).

(2*R*,5*R*)-5-(4-Methoxyphenyl)-2-phenyloxazolidin-4-one 34

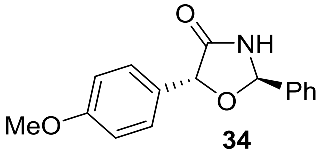

34

Following *General Procedure 4*, oxazolidin-4-one **18a** (50 mg, 0.12 mmol) in THF (1.2 mL) at –78 °C and SmI_2 (*ca*. 0.07 M in THF, 3.5 mL) added dropwise. The crude product was purified by

Biotage® Isolera™ 4 [36 mLmin^{-1}, hexane:EtOAc (90:10 to 50:50 10CV)] to give the title compound (21 mg, 65%) as a white solid. mp 138–139 °C; $[\alpha]_D^{20}$ –72.9 (*c* 0.94 in $CHCl_3$); Chiral HPLC analysis, Chiralpak AD-H (90:10 hexane : IPA, flow rate 1.0 mLmin^{-1}, 211 nm, 30 °C) t_R (2*R*,5*R*): 17.8 min, t_R (2*S*,5*S*): 24.0 min, 99% ee; ν_{max} (film) 3200, 1707, 1611, 1512, 1460, 1244, 1175; ^{1}H NMR (300 MHz, $CDCl_3$) δ_H: 3.82 (3H, s, OC*H*$_3$), 5.35 (1H, d, *J* 2.2, OC(5)*H*), 6.29 (1H, d, *J* 2.2, OC(2)*H*N), 6.94 (2H, d, *J* 8.8, C(5)ArC(3',5')*H*), 7.37–7.52 (7H, m, C(2)Ar*H* and C(5)ArC(2',6')*H*), 8.40 (1H, br. s, N*H*); ^{13}C{^{1}H} NMR (75 MHz, $CDCl_3$) δ_C: 55.5, 78.6, 87.9, 114.4, 126.5, 128.1, 128.3, 129.0, 129.9, 138.6, 160.1, 174.3; *m/z* (NSI$^+$) 269 ([M+H]$^+$, 100%); HMRS (NSI$^+$) $C_{16}H_{16}NO_3$ [M+H]$^+$ found 270.1127, requires 270.1125 (+0.7 ppm).

(*R*)-2-Hydroxy-2-phenylacetic acid ((*R*)-(–)-Mandelic acid (35))

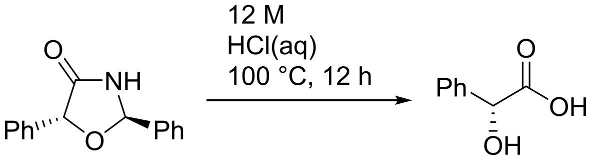

According to a literature procedure,[15] oxazolidin-4-one **32** (87 mg, 0.36 mmol) was suspended in 12 M HCl (3.6 mL) and the solution heated to 100 °C for 16 h. The reaction was cooled to rt, diluted with H_2O (10 mL) and extracted with EtOAc (3 × 10 mL). The combined organics were dried over $MgSO_4$, filtered and concentrated under reduced pressure. The crude product was purified by recrystallisation from hot $CHCl_3$ to give the title compound **35** (55 mg, 100%) as off-white crystals with data in accordance with the literature.[16] mp 126–128 °C ($CHCl_3$) {Lit.[17] 131–133 °C }; $[\alpha]_D^{20}$ –150.0 (*c* 0.22 in H_2O) {(*R*)-**35**[18] $[\alpha]_D^{20}$ –150.8 (*c* 0.24 in H_2O)}; ^{1}H NMR (300 MHz, $CDCl_3$) δ_H: 5.26 (1H, s, C*H*OH), 7.33–7.41 (3H, m, ArH), 7.43–7.48 (2H, m, ArH).

References

1. N. Duguet, C. D. Campbell, A. M. Z. Slawin, A. D. Smith, *Org. Biomol. Chem*. **2008**, *6*, 1108–1113.
2. K. Yohida, N. Akashi, A. Yanagisawa, *Tetrahedron*: *Asymmetry,* **2011**, *22*, 1225–1230.
3. Z. Xu, X. Lu, *J. Org. Chem.*, **1998**, *63*, 5031–5041.
4. J. A. Jenkins, T. Cohen, *J. Org. Chem.*, **1975**, *40*, 3566–3571
5. L.C. Morrill, L. A. Ledingham, J.-P. Couturier, J.Bickel, A. D. Harper, C. Fallan, A. D. Smith, *Org. Biomol. Chem.*, **2014**, *12*, 624–636.
6. A. A. M. Roof, H. F. van Woerden, H. Cerfontain, *Tetrahedron* **1976**, *32*, 2967–2971.
7. D. Uraguchi, R. Tsutsumi, T.i Ooi, *Tetrahedron* **2014**, 70, 1691–1701.
8. P.-L. Shao, X. Y. Chen, S. Ye, *Angew. Chem. Int. Ed.* **2010**, *49*, 8412–8416.
9. F. A. Davis, U. K. Nadir, E. W. Kluger, *J. Chem. Soc., Chem. Commun.*, **1977**, 25–26.
10. L. Lykke, C. Rodríguez-Escrich, K. A. Jørgensen, *J. Am. Chem. Soc.* **2011**, *133*, 14932–14935.
11. P.-L. Shao, L. Shen, S. Ye, *Chin. J. Chem.* **2012**, *30*, 2688–2692.
12. R. Kadyrov, R. M. Koenigs, C. Brinkmann, D. Voigtlaender, M. Rueping, *Angew. Chem. Int. Ed.* **2009**, *48*, 7556–7559.
13. M. Szostak, M. Spain, D. J. Procter, *J. Org. Chem.* **2012**, *77*, 3049-3059.
14. Samarium metal (40 mesh) purchased from Acros Organics (product code: 10450721) and stored in an inert atmopshere glove box.
15. G. Cardillo, M. A. Hashem, C. Tomasini, *J. Chem. Soc., Perkin Trans. 1* **1990**, 1487-1488.
16. A. Baeza, J. Casas, C. Nájera, J. M. Sansano, J. M. Saá, *Eur. J. Org. Chem.* **2006**, 1949-1958.
17. P. L. Polavarapu, L. P. Fontana, H. E. Smith, *J. Am. Chem. Soc.* **1986**, *108*, 94-99.
18. Authentic sample of (*R*)-(–)-mandelic acid **35** purchased from Sigma-Aldrich (98%, product code M2209).

^{1}H and ^{13}C{^{1}H} NMR Spectra of New Compounds

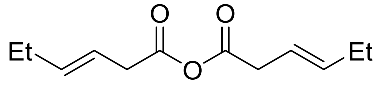

S10

^{1}H NMR (400 MHz, $CDCl_3$)

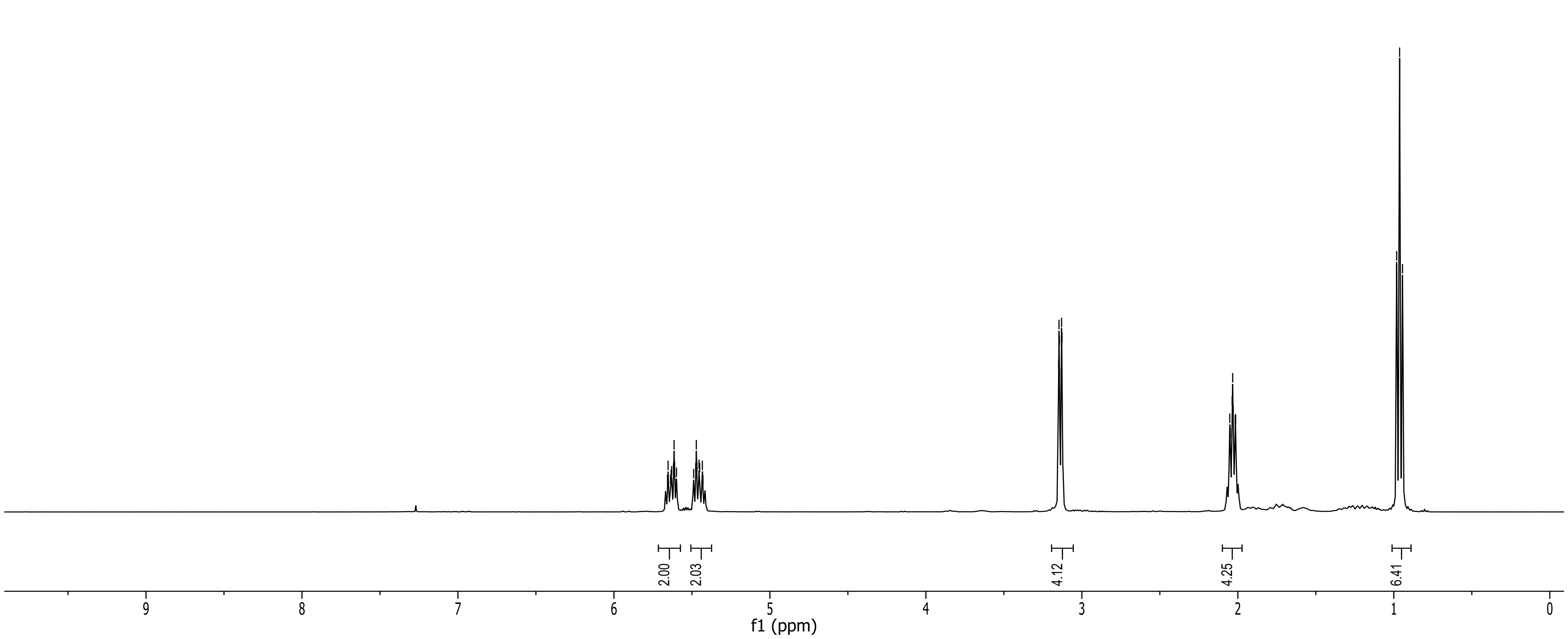

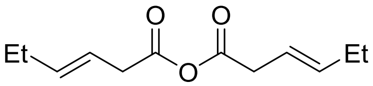

S10

$^{13}C\{^{1}H\}$ NMR (100 MHz, $CDCl_3$)

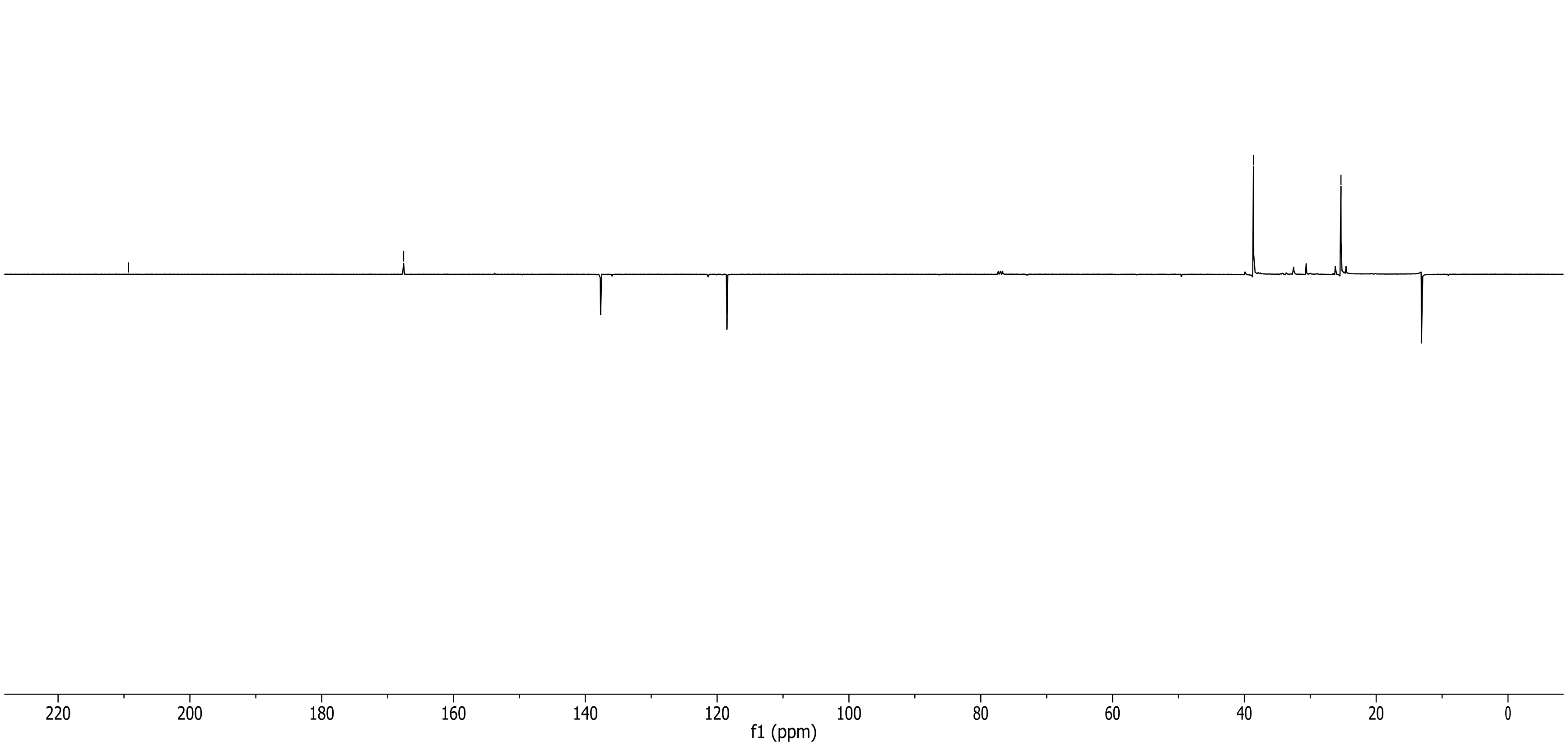

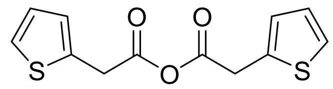

S11

^{1}H NMR (400 MHz, $CDCl_3$)

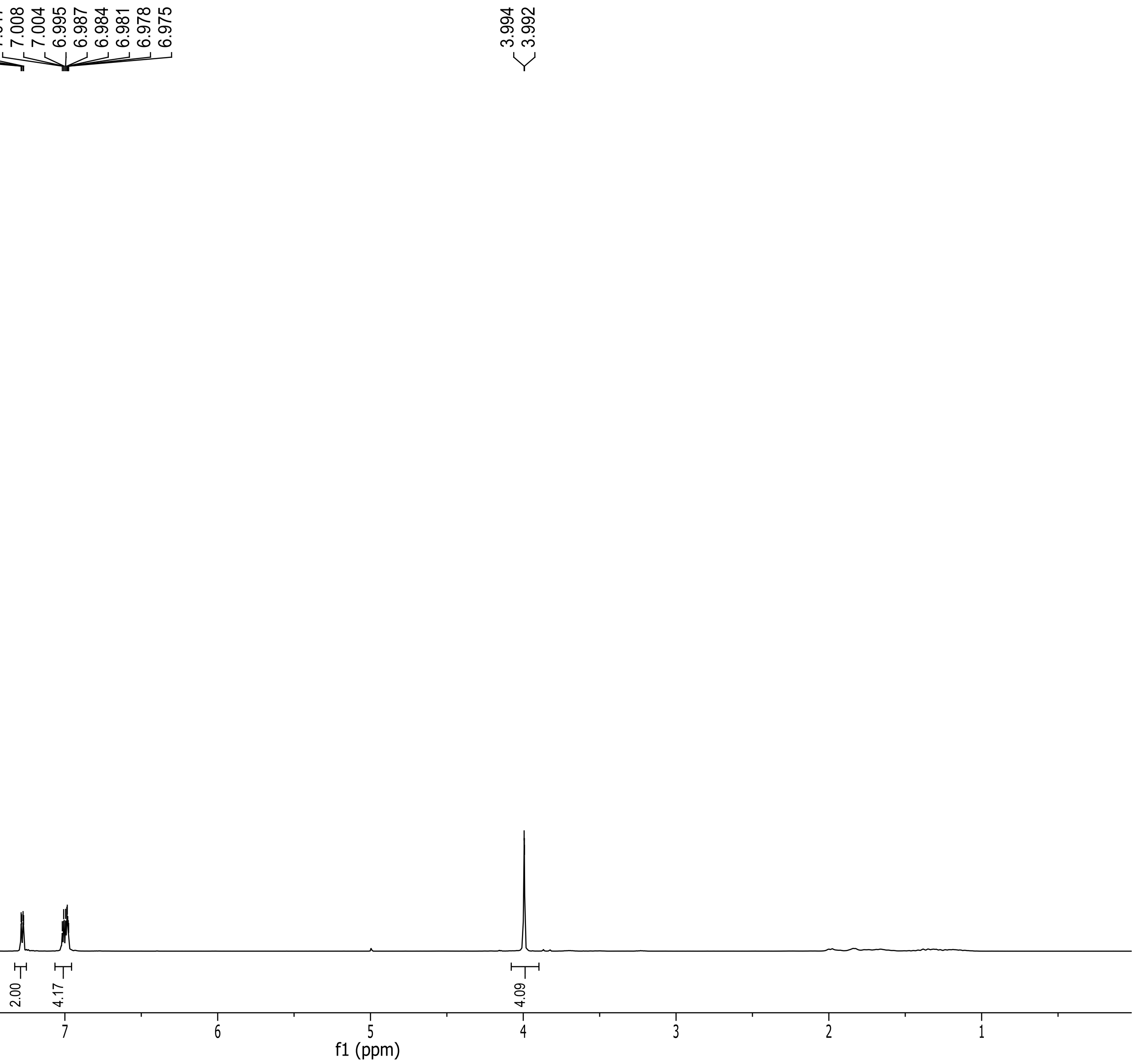

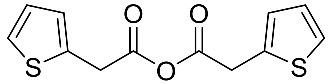

S11

$^{13}C\{^1H\}$ NMR (100 MHz, $CDCl_3$)

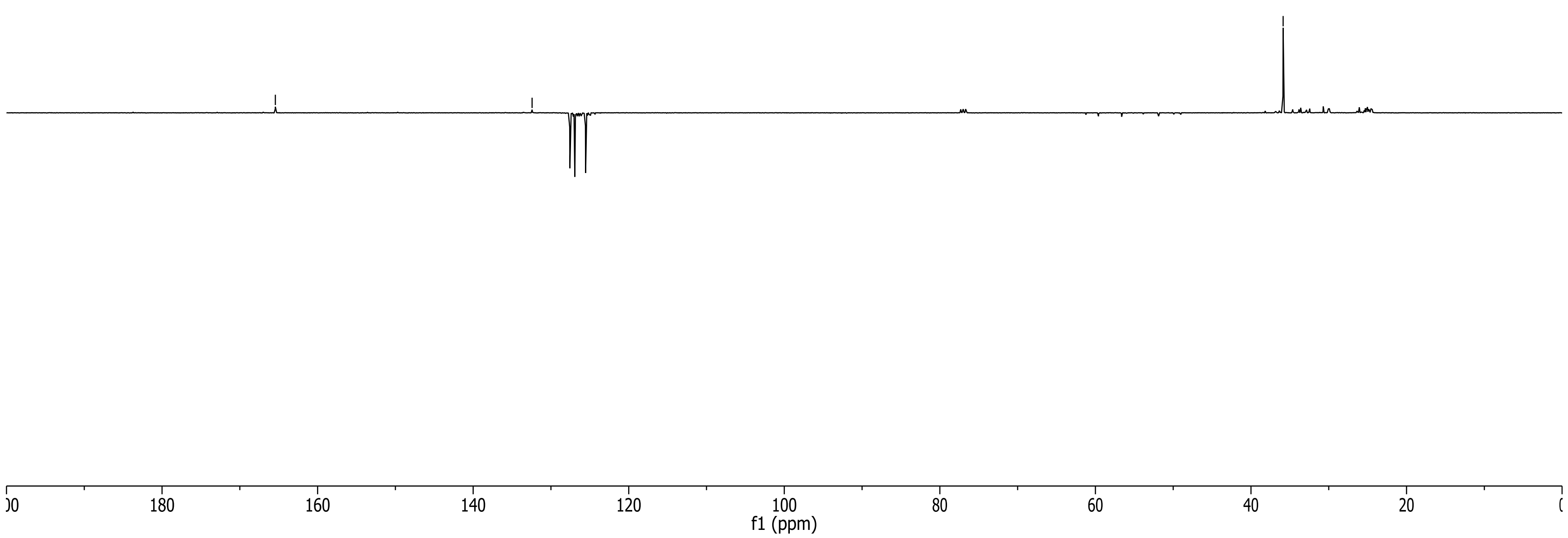

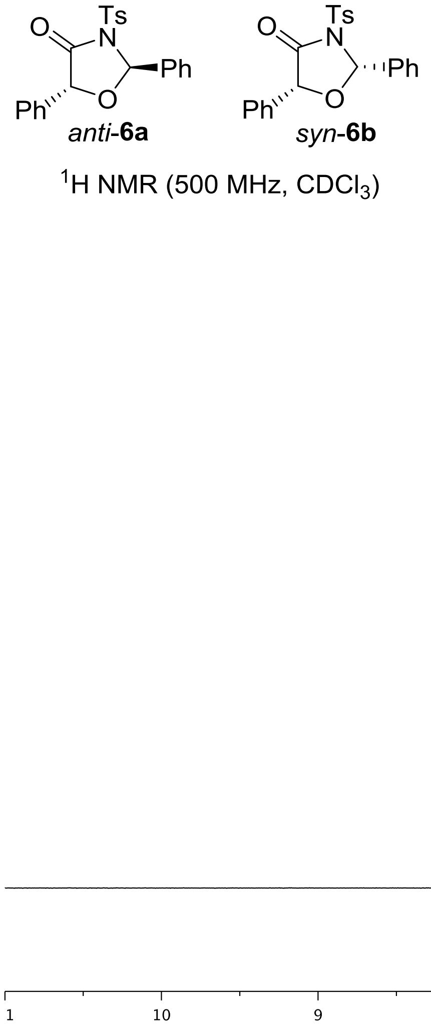

anti-**6a** syn-**6b**

^{1}H NMR (500 MHz, $CDCl_3$)

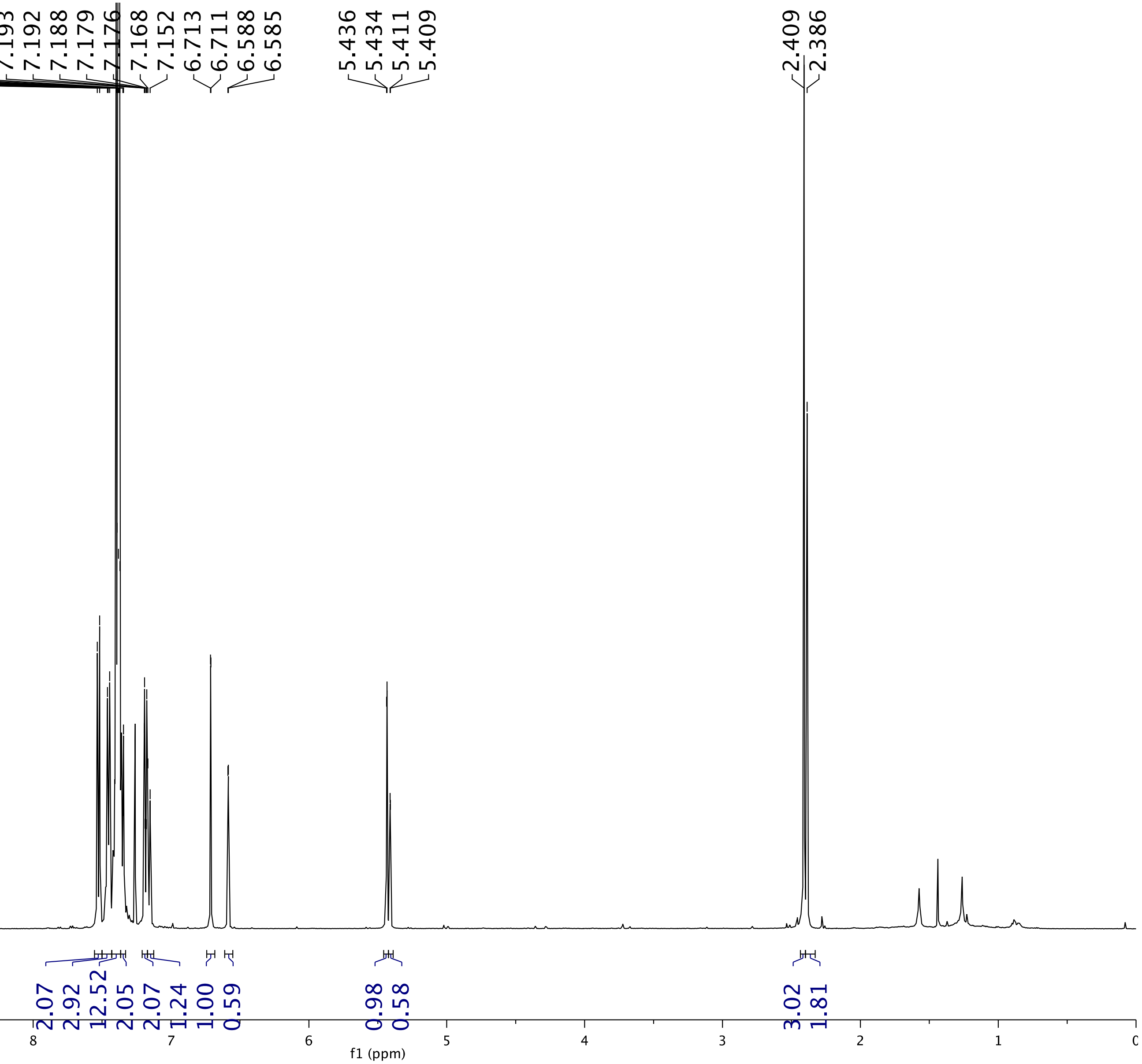

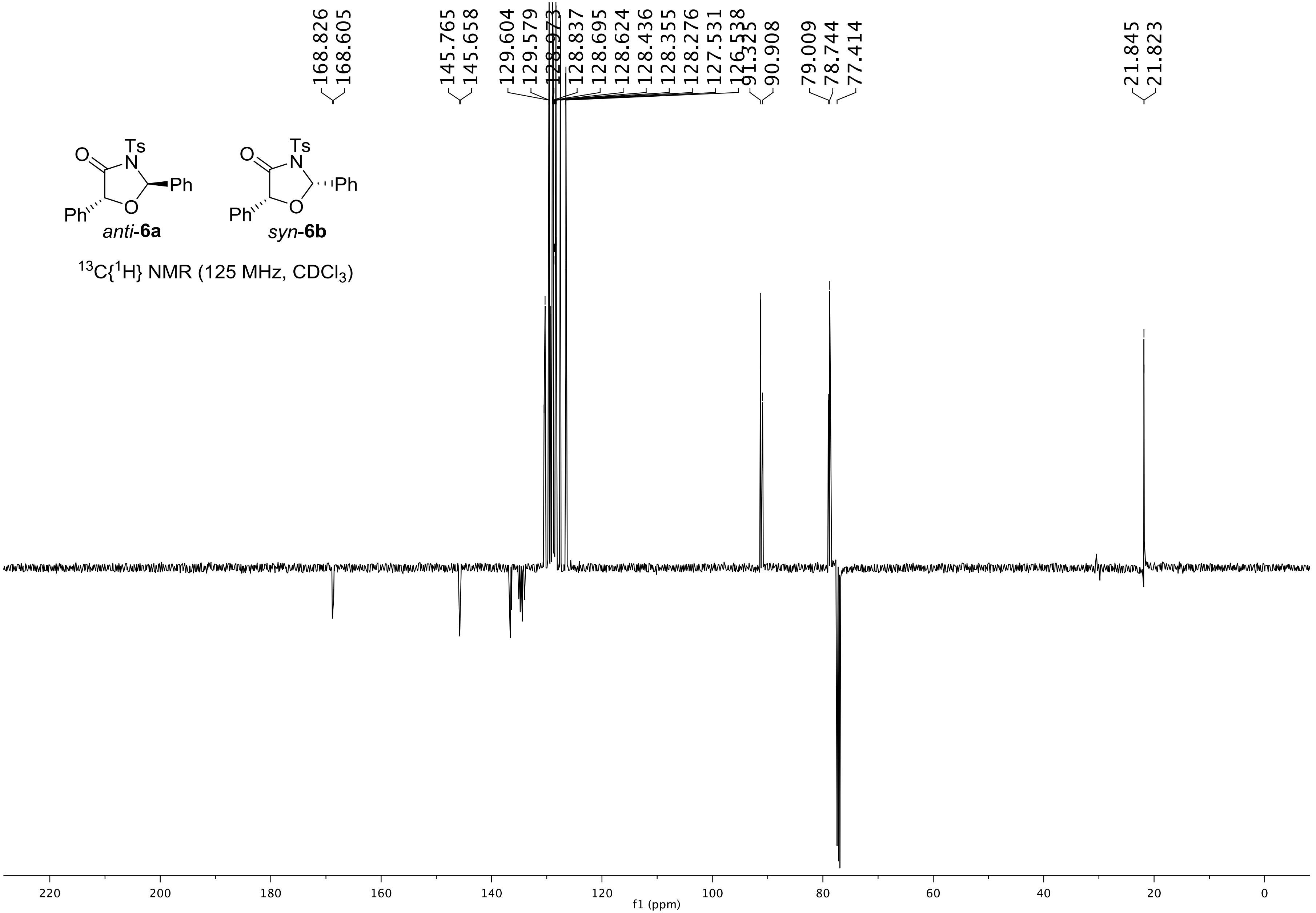
168.826
168.605
145.765
145.658
129.604
129.579
128.973
128.837
128.695
128.624
128.436
128.355
128.276
127.531
126.538
91.325
90.908
79.009
78.744
77.414
21.845
21.823
Ts
O
N
Ph
Ph
O
anti-6a
syn-6b
$^{13}C\{^1H\}$ NMR (125 MHz, $CDCl_3$)
220
200
180
160
140
120
100
80
60
40
20
0
f1 (ppm)

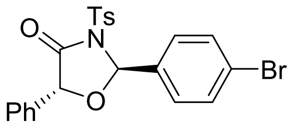

anti-**12a**

^{1}H NMR (500 MHz, $CDCl_3$)

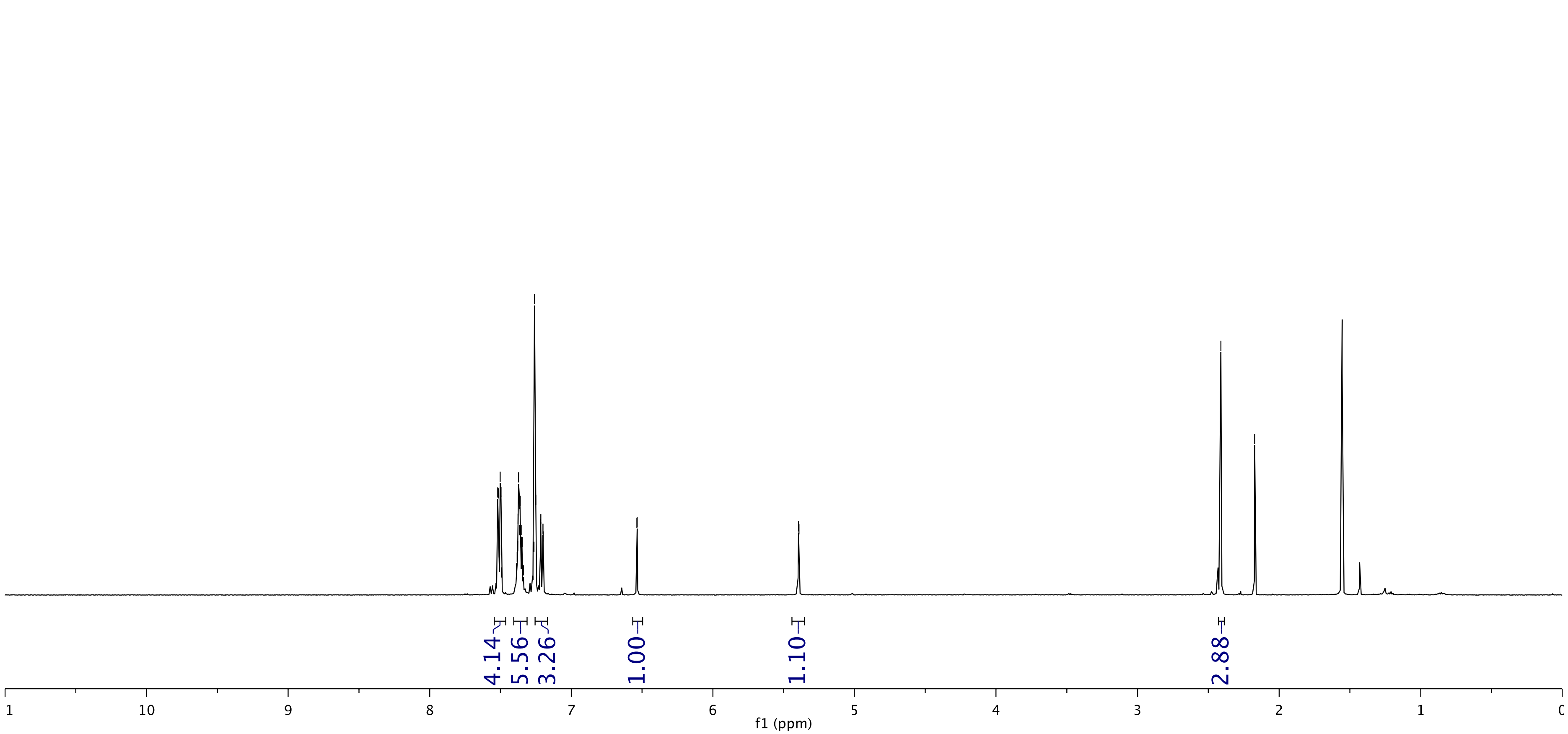

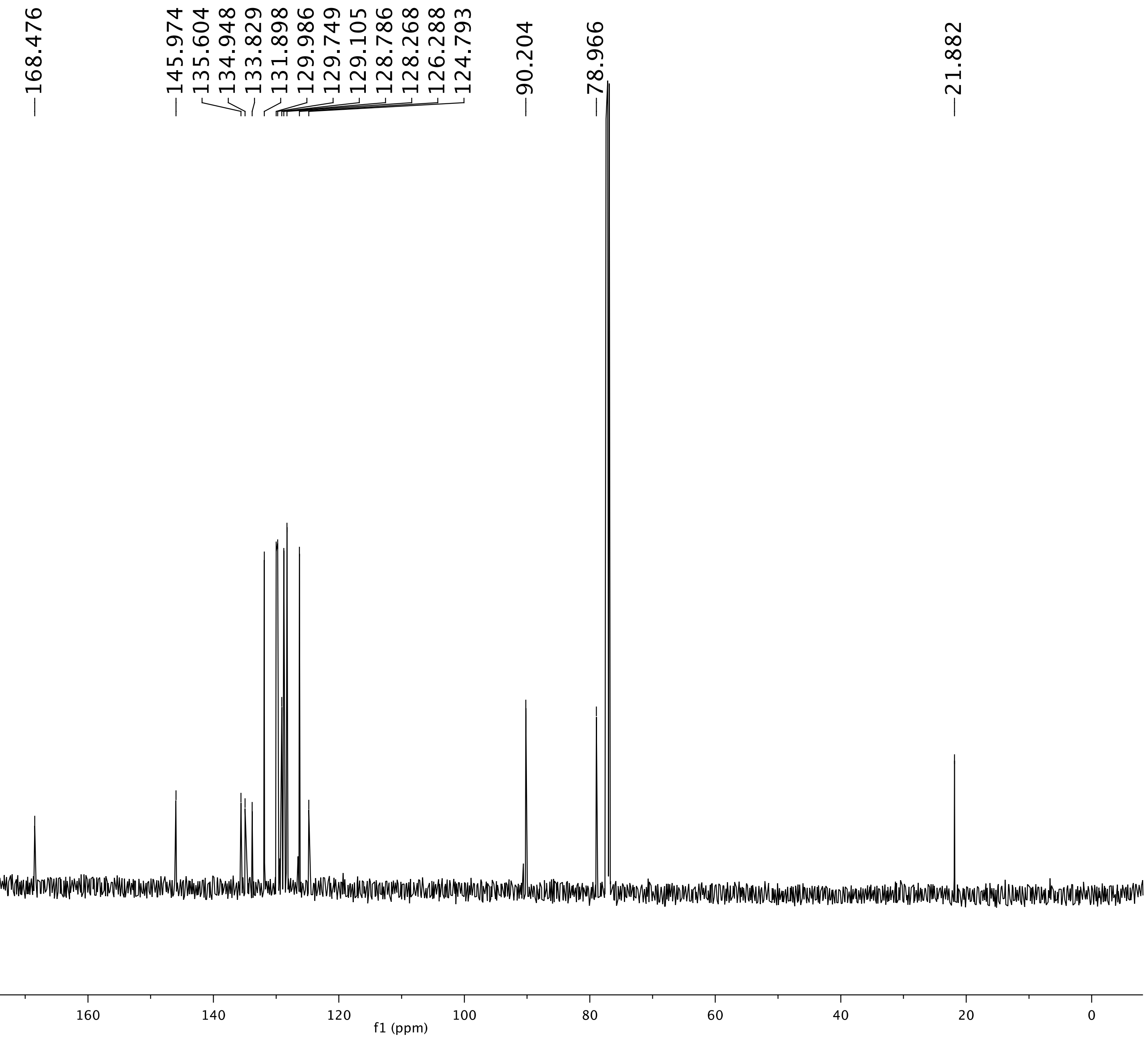

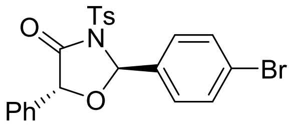

anti-**12a**

$^{13}C\{^{1}H\}$ NMR (125 MHz, $CDCl_3$)

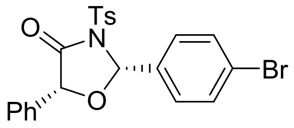

syn-**12b**

^{1}H NMR (500 MHz, $CDCl_3$)

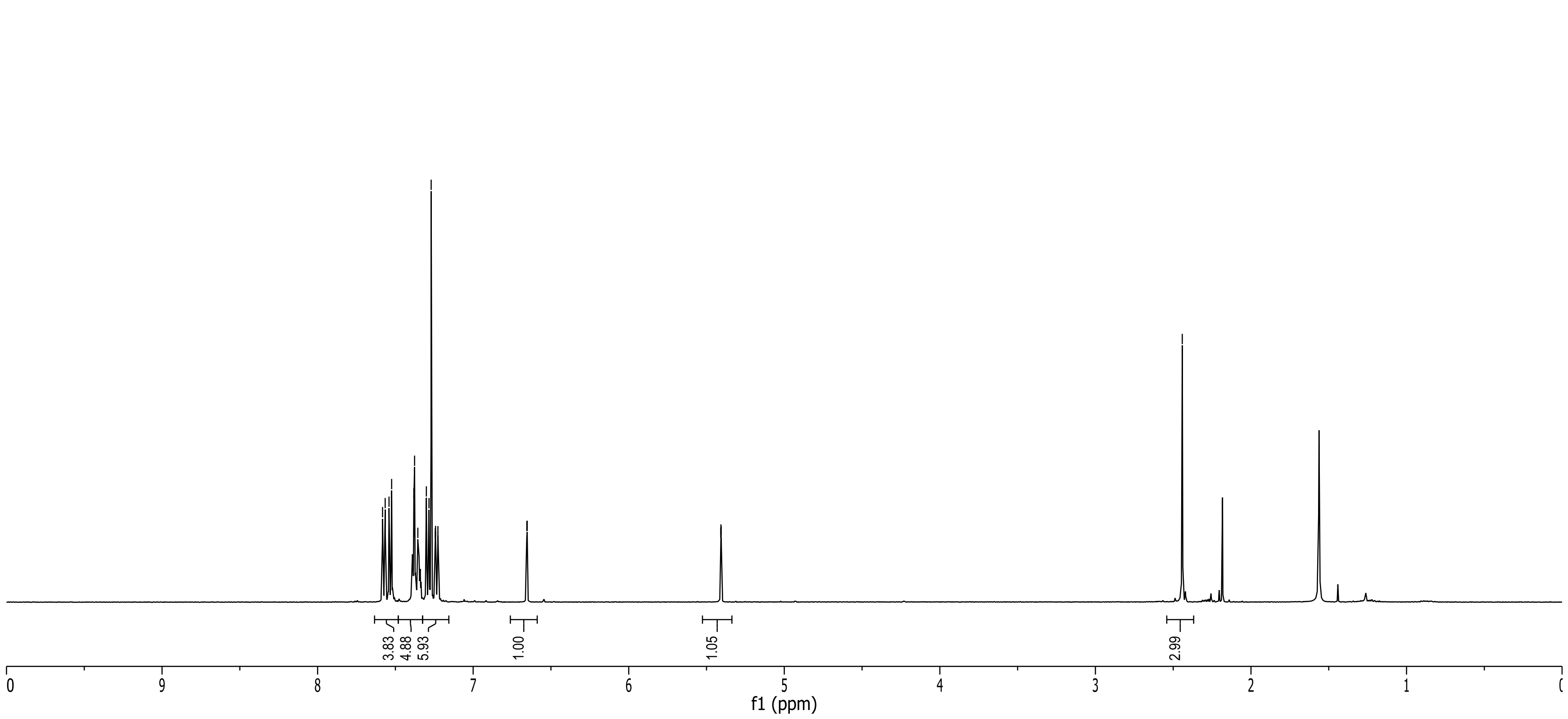

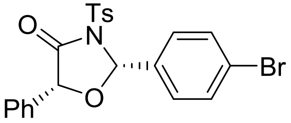

syn-**12b**

$^{13}C\{^{1}H\}$ NMR (125 MHz, $CDCl_3$)

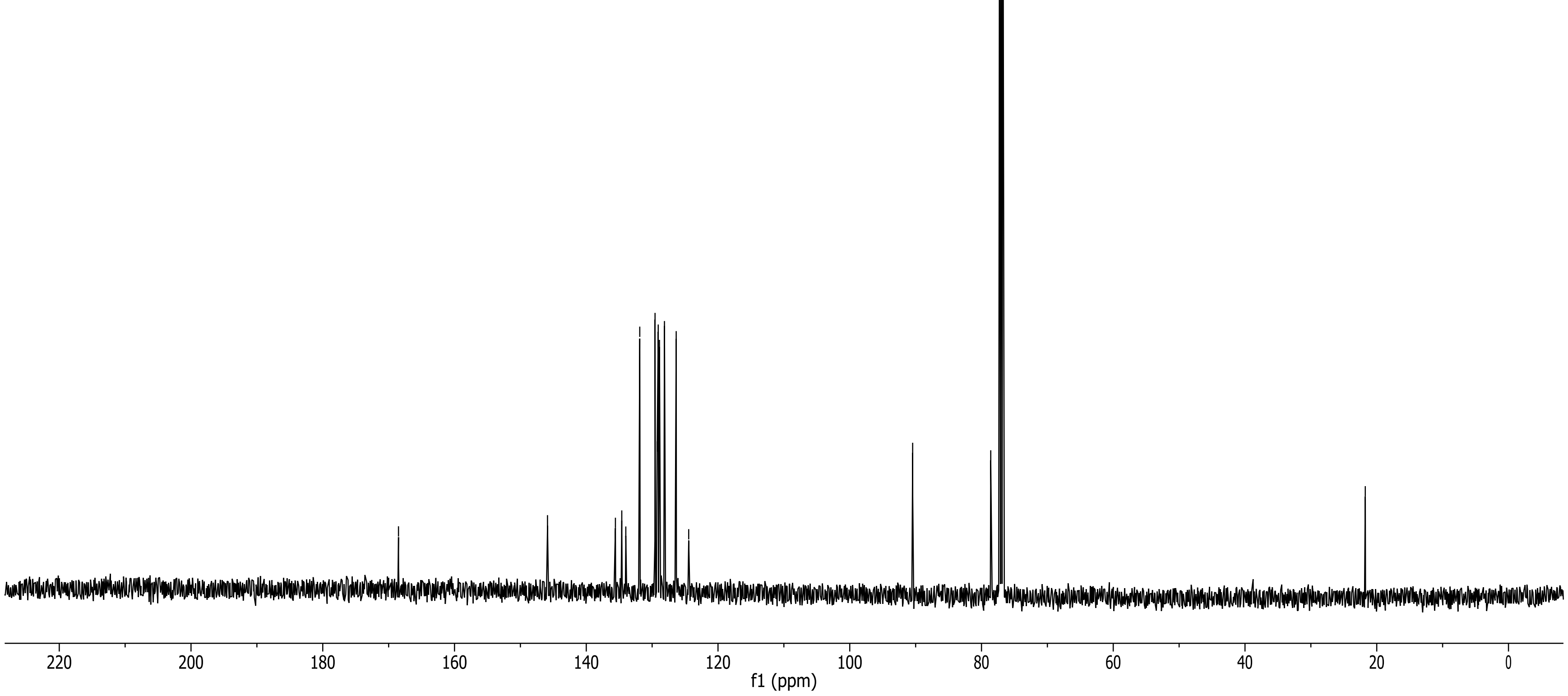

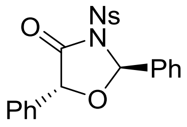

anti-**13a**

^{1}H NMR (500 MHz, $CDCl_3$)

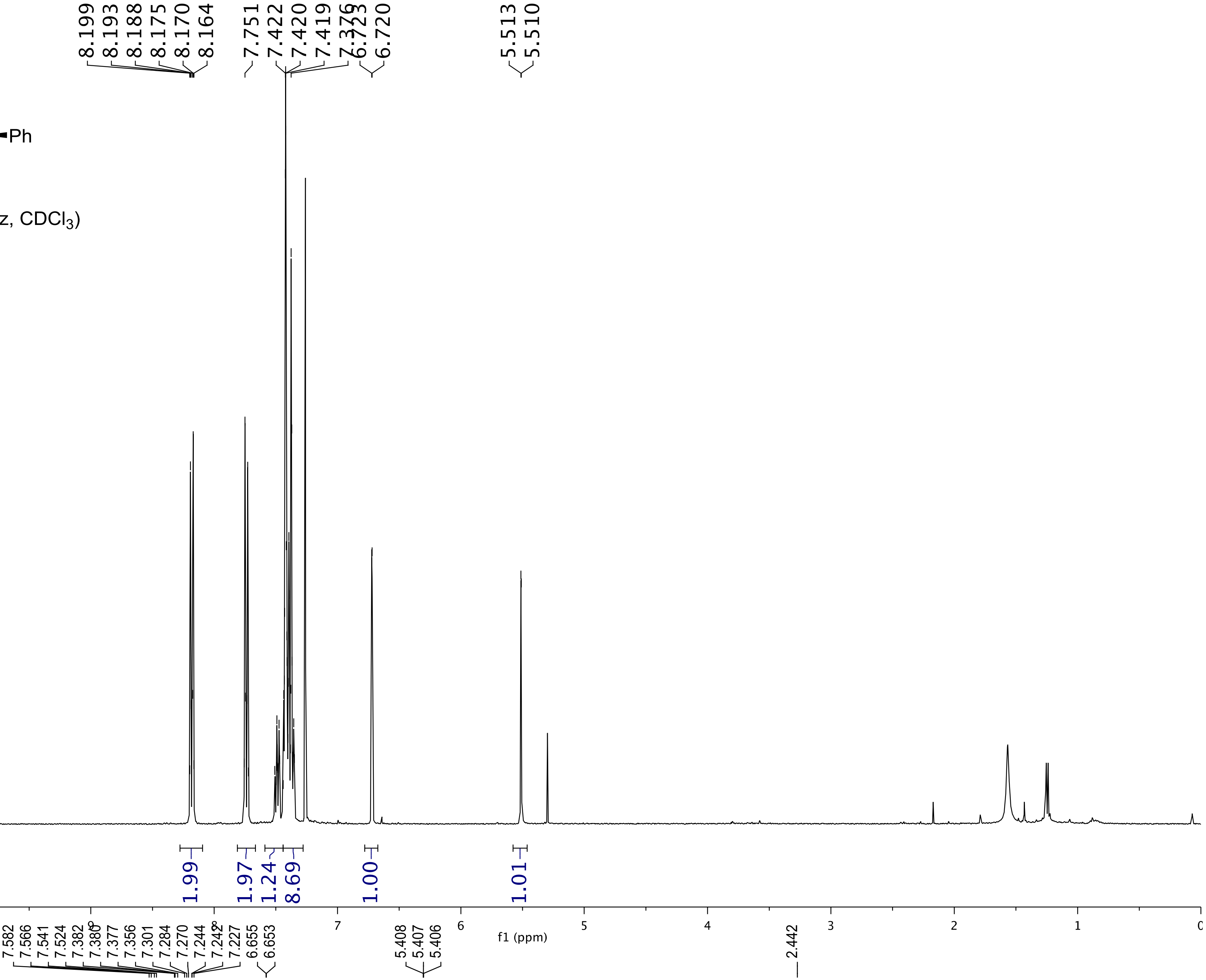

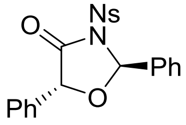

anti-**13a**

$^{13}C\{^{1}H\}$ NMR (125 MHz, $CDCl_3$)

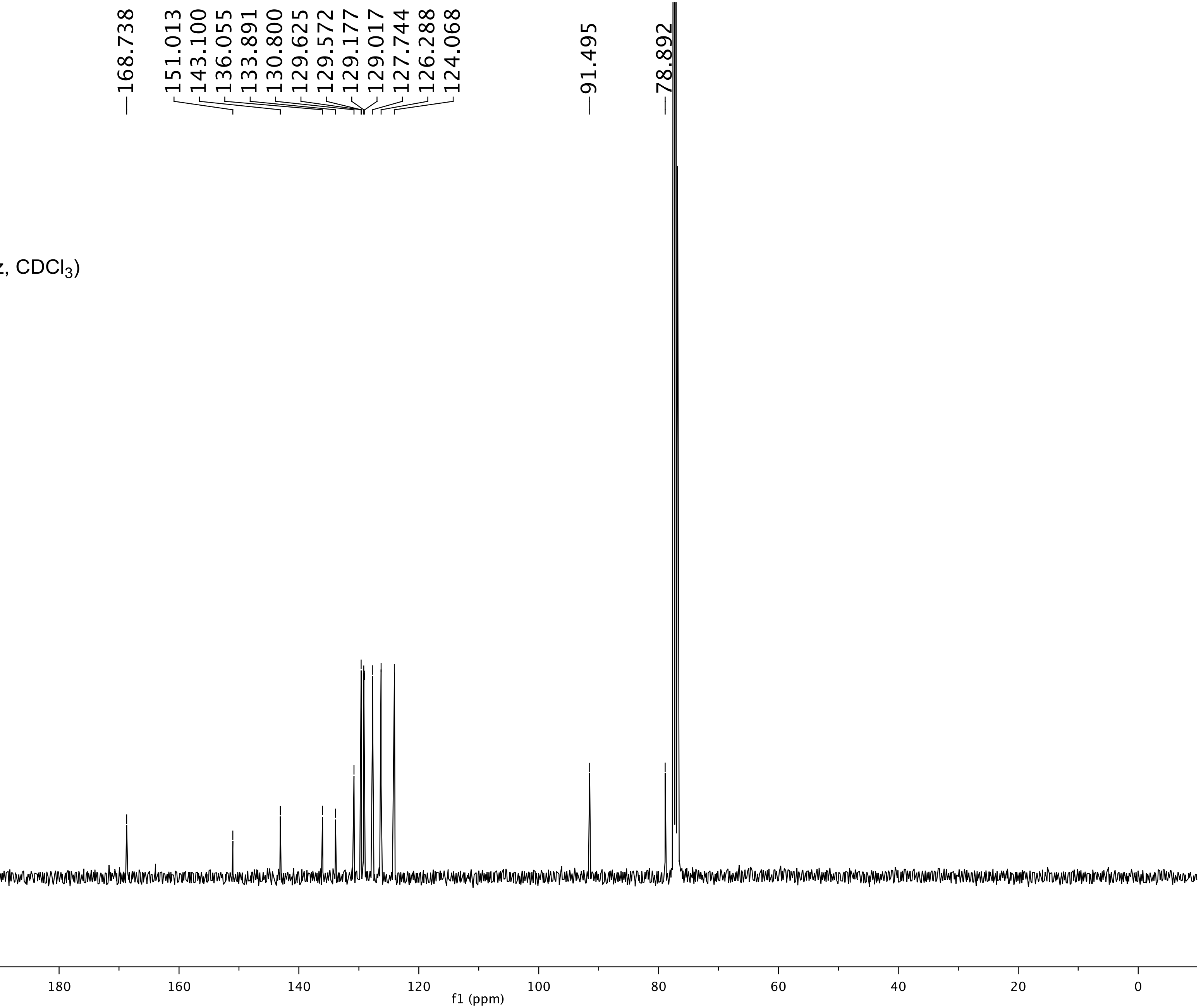

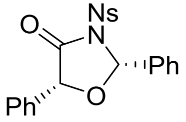

syn-**13b**

^{1}H NMR (500 MHz, $CDCl_3$)

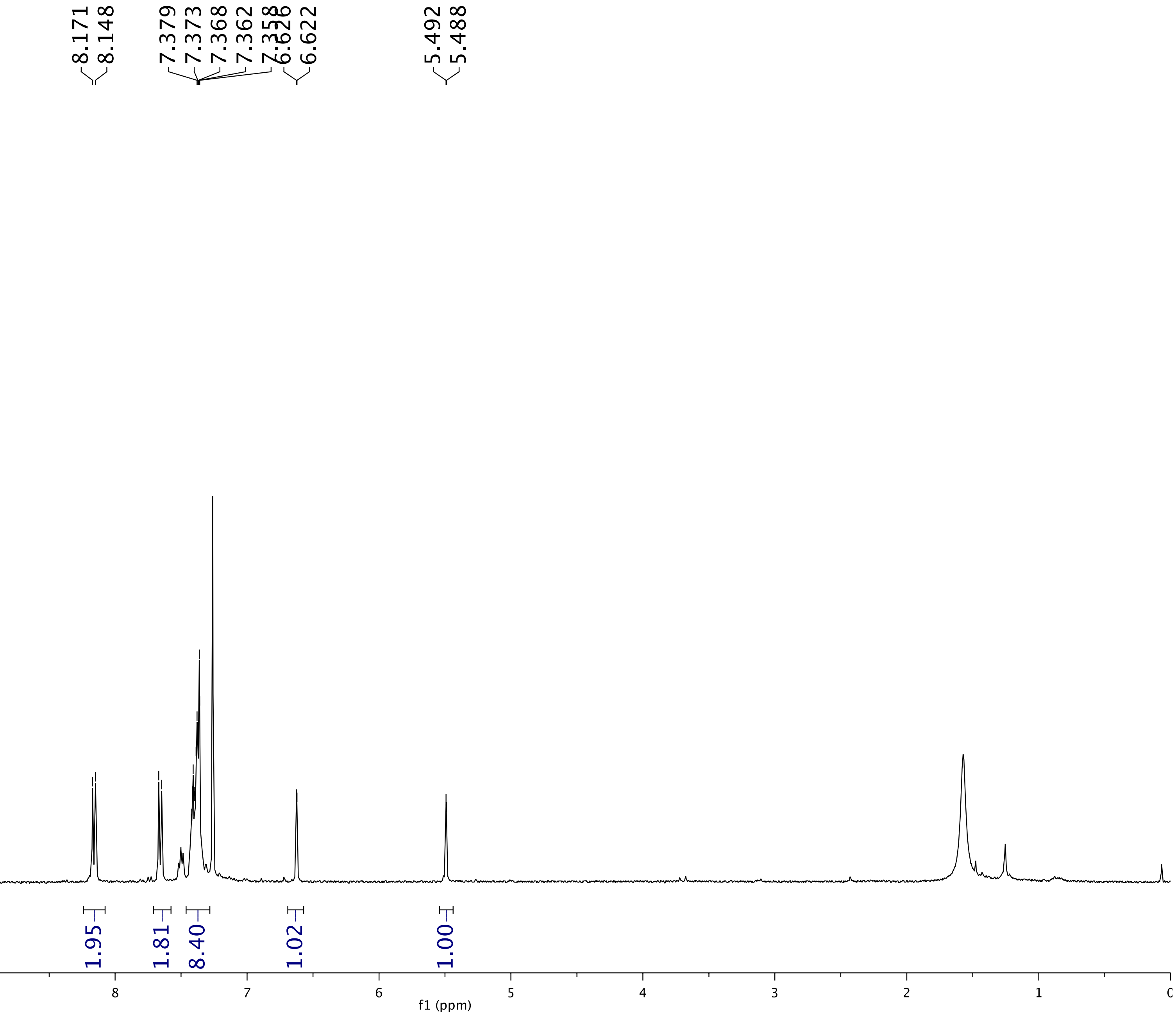

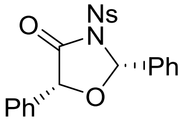

syn-**13b**

$^{13}C\{^{1}H\}$ NMR (125 MHz, $CDCl_3$)

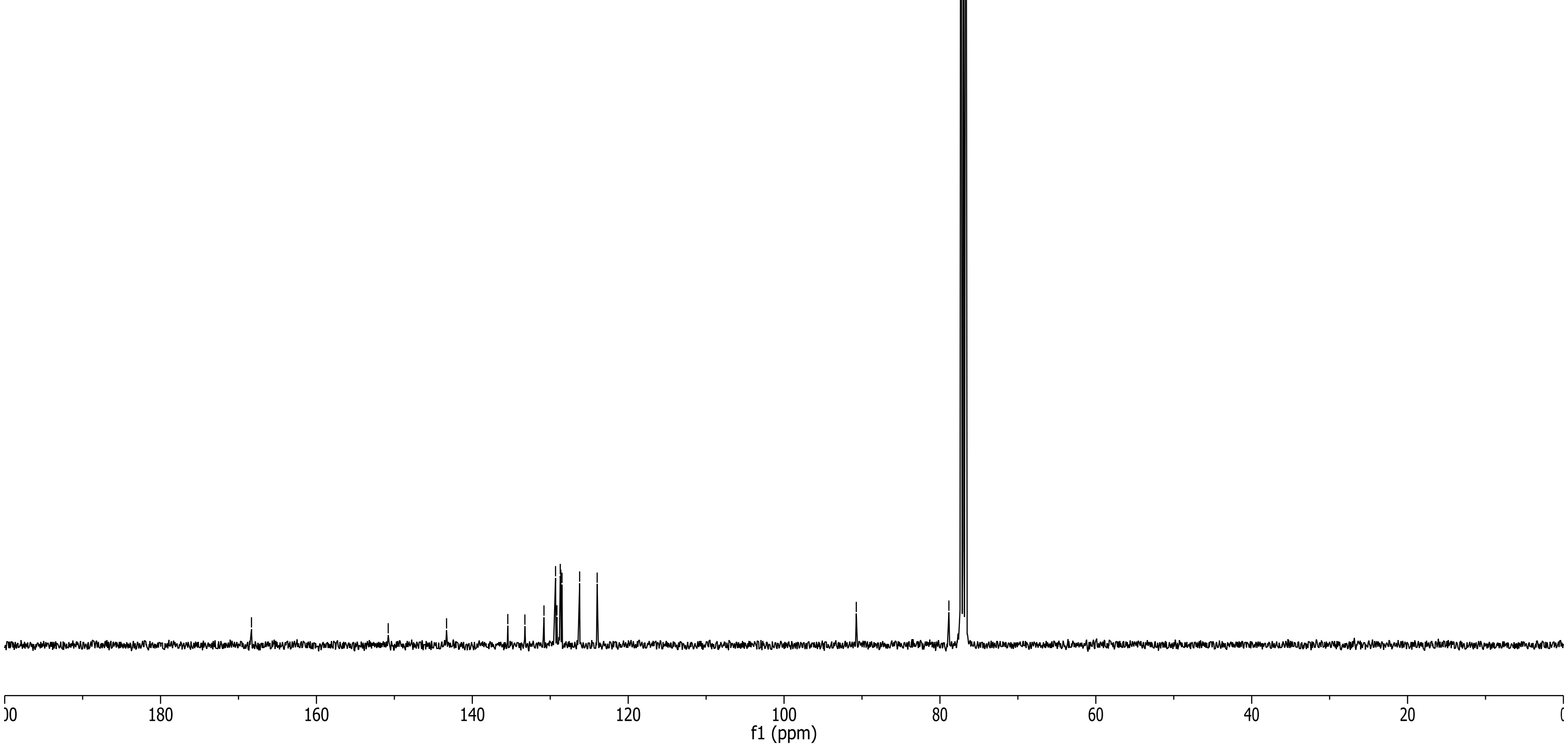

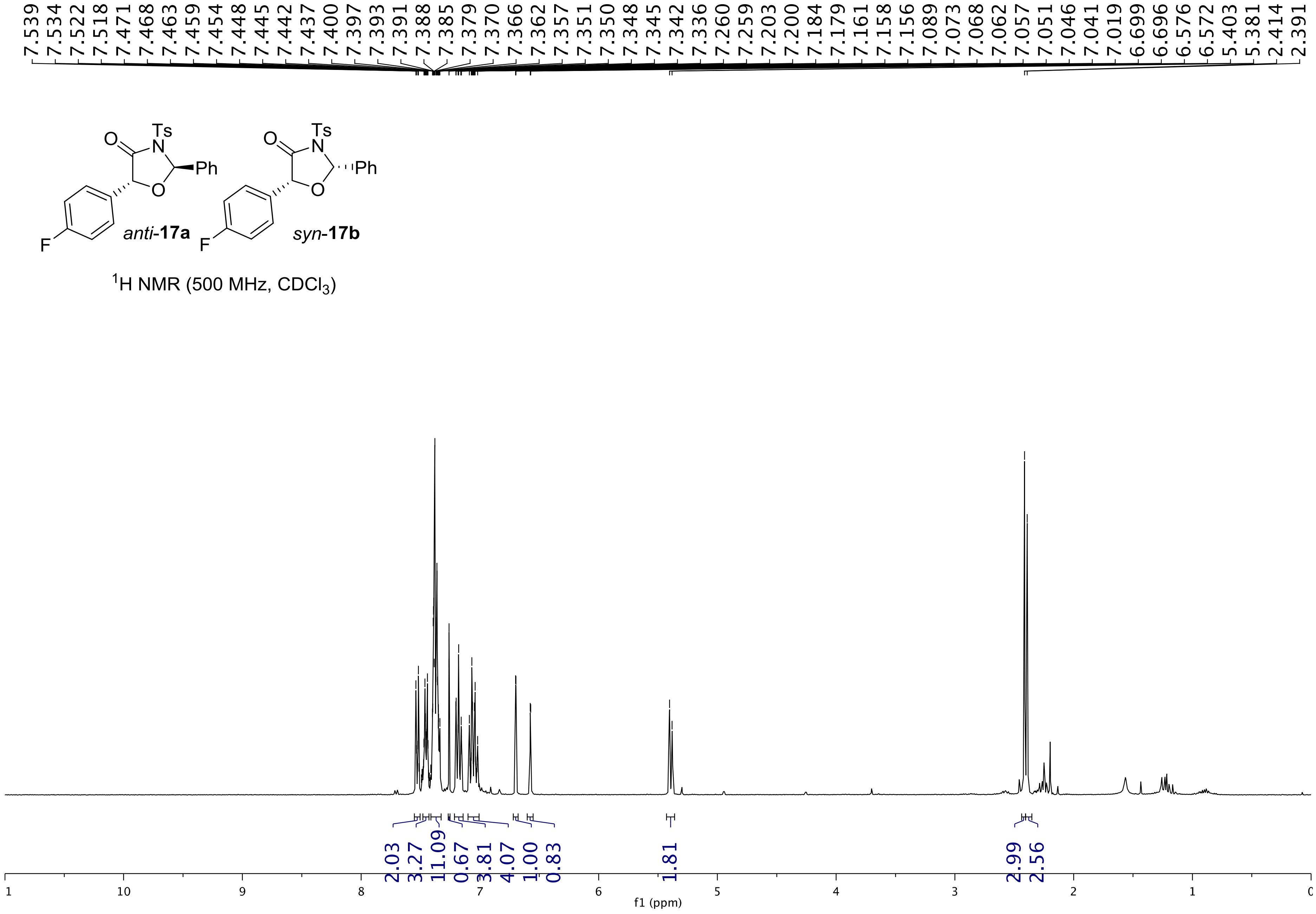

^{1}H NMR (500 MHz, $CDCl_3$)

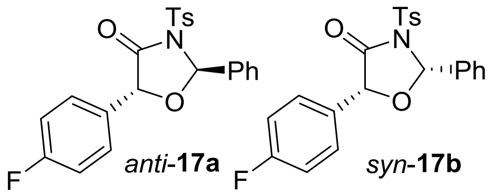

anti-**17a** *syn*-**17b**

$^{13}C\{^1H\}$ NMR (125 MHz, $CDCl_3$)

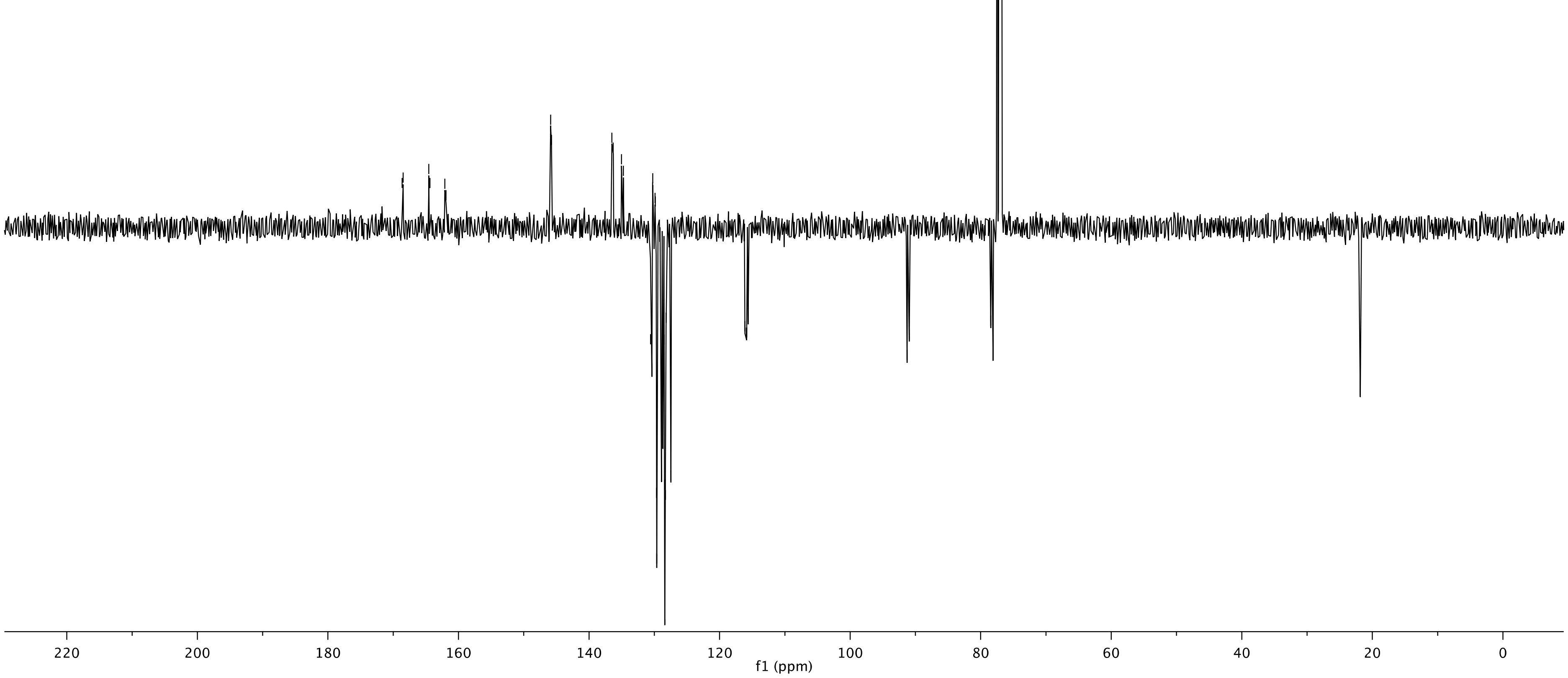

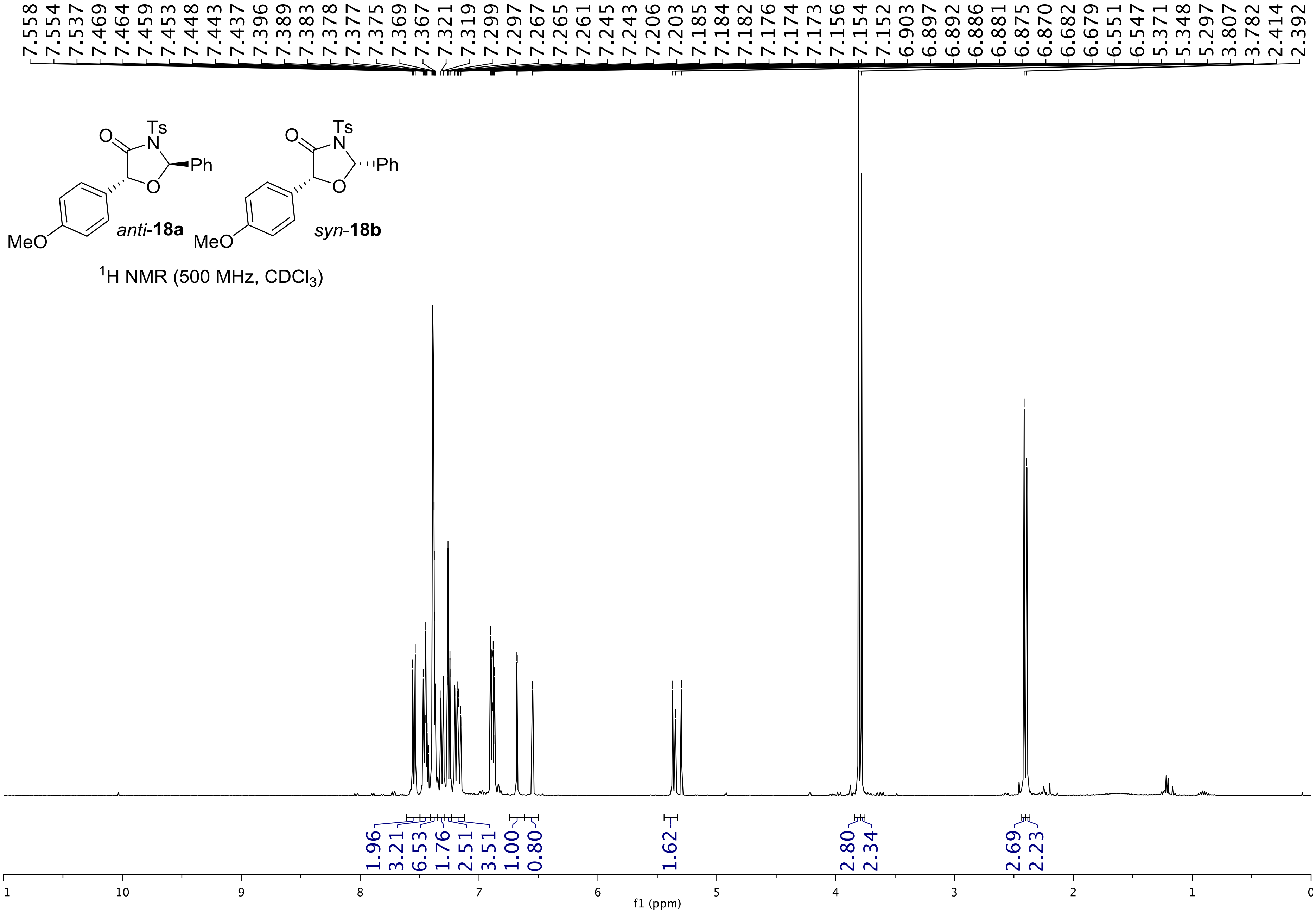
7.558 7.554 7.537 7.469 7.464 7.459 7.453 7.448 7.443 7.437 7.396 7.389 7.383 7.378 7.377 7.375 7.369 7.367 7.321 7.319 7.299 7.297 7.267 7.265 7.261 7.245 7.243 7.206 7.203 7.185 7.184 7.182 7.176 7.174 7.173 7.156 7.154 7.152 6.903 6.897 6.892 6.886 6.881 6.875 6.870 6.682 6.679 6.551 6.547 5.371 5.348 5.297 3.807 3.782 2.414 2.392
O
Ts
N
Ph
O
MeO
anti-**18a**
syn-**18b**
^{1}H NMR (500 MHz, CDCl$_3$)
1.96 3.21 6.53 1.76 2.51 3.51 1.00 0.80 1.62 2.80 2.34 2.69 2.23
10 9 8 7 6 5 4 3 2 1
f1 (ppm)

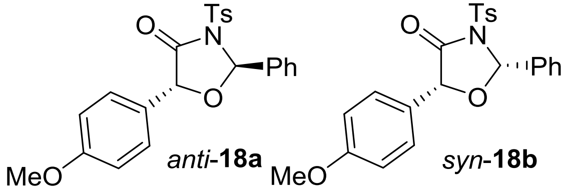

$^{13}C\{^{1}H\}$ NMR (125 MHz, $CDCl_3$)

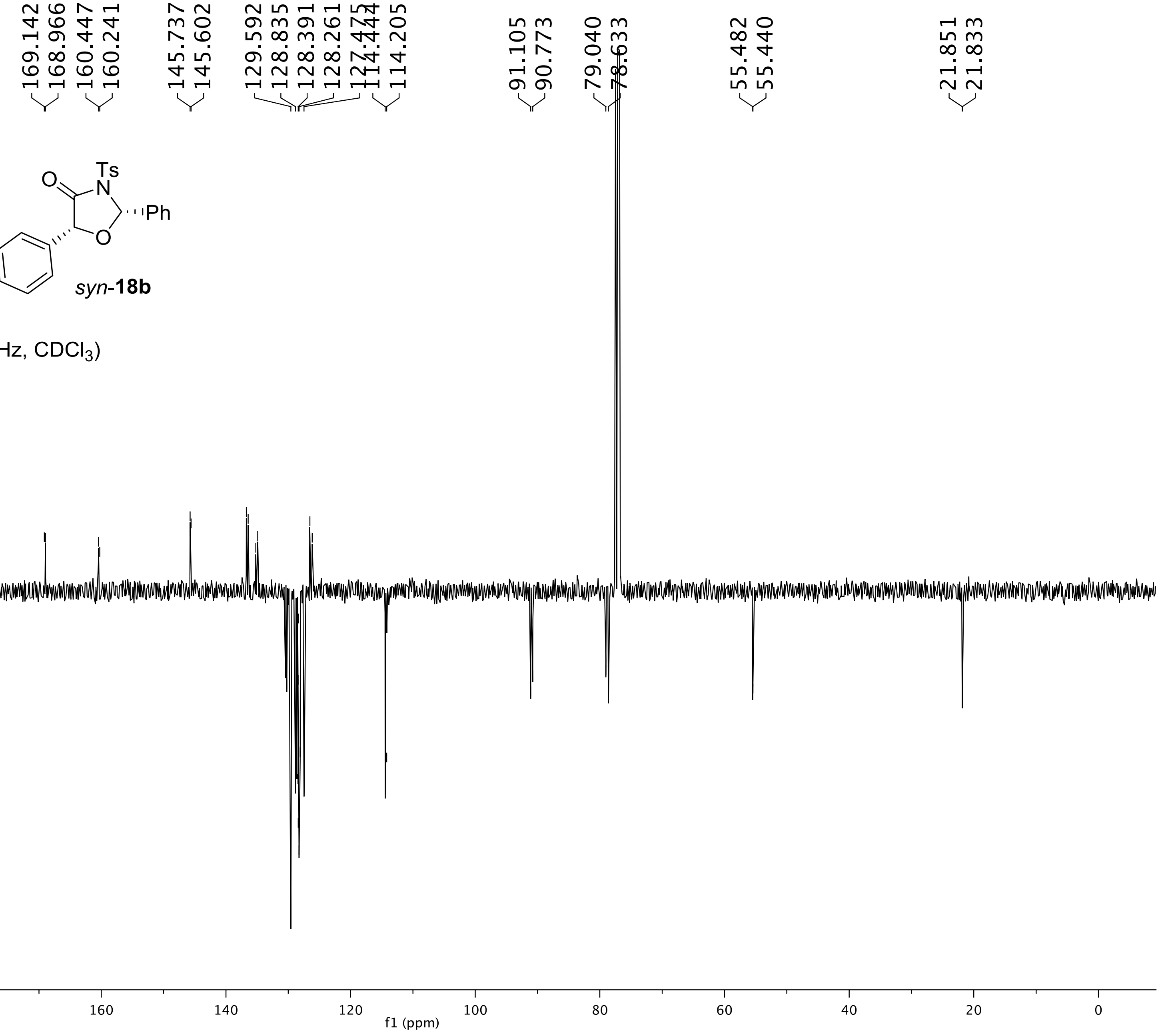

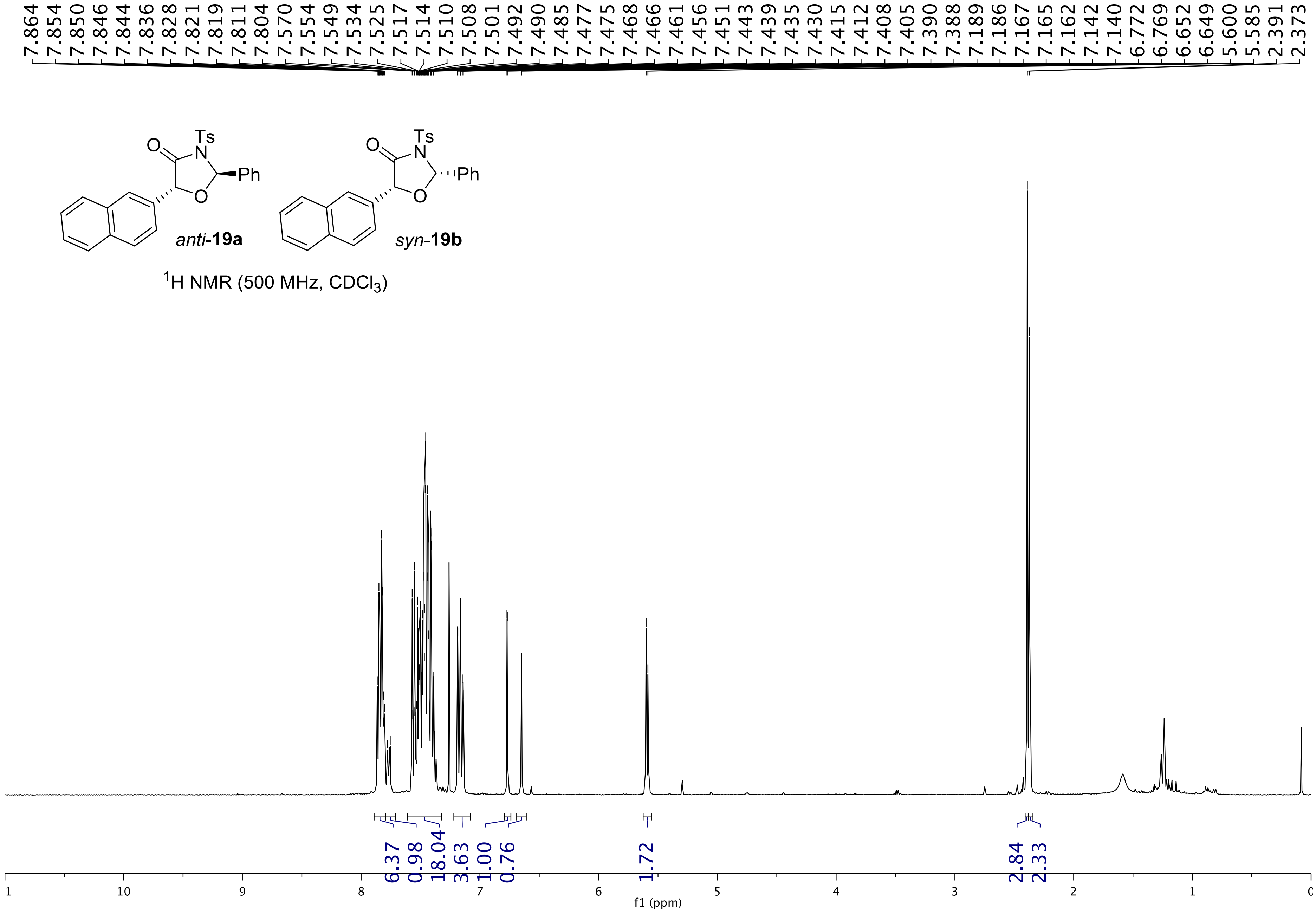

^{1}H NMR (500 MHz, $CDCl_3$)

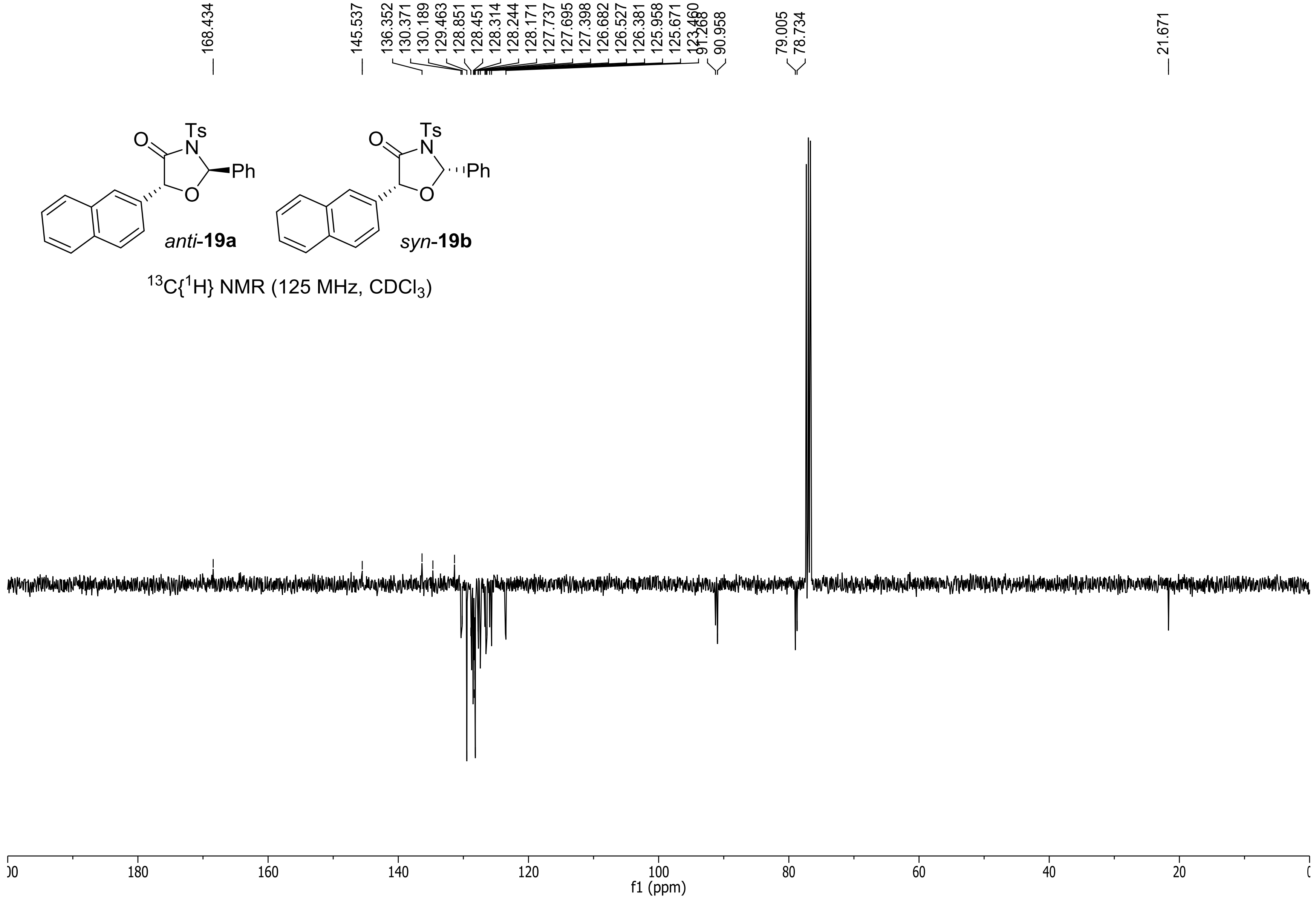
168.434
145.537
136.352
130.371
130.189
129.463
128.851
128.451
128.314
128.244
128.171
127.737
127.695
127.398
126.682
126.527
126.381
125.958
125.671
123.460
91.268
90.958
79.005
78.734
21.671
O
N
Ts
Ph
O
anti-19a
syn-19b
13C{1H} NMR (125 MHz, CDCl3)
180
160
140
120
100
80
60
40
20
f1 (ppm)

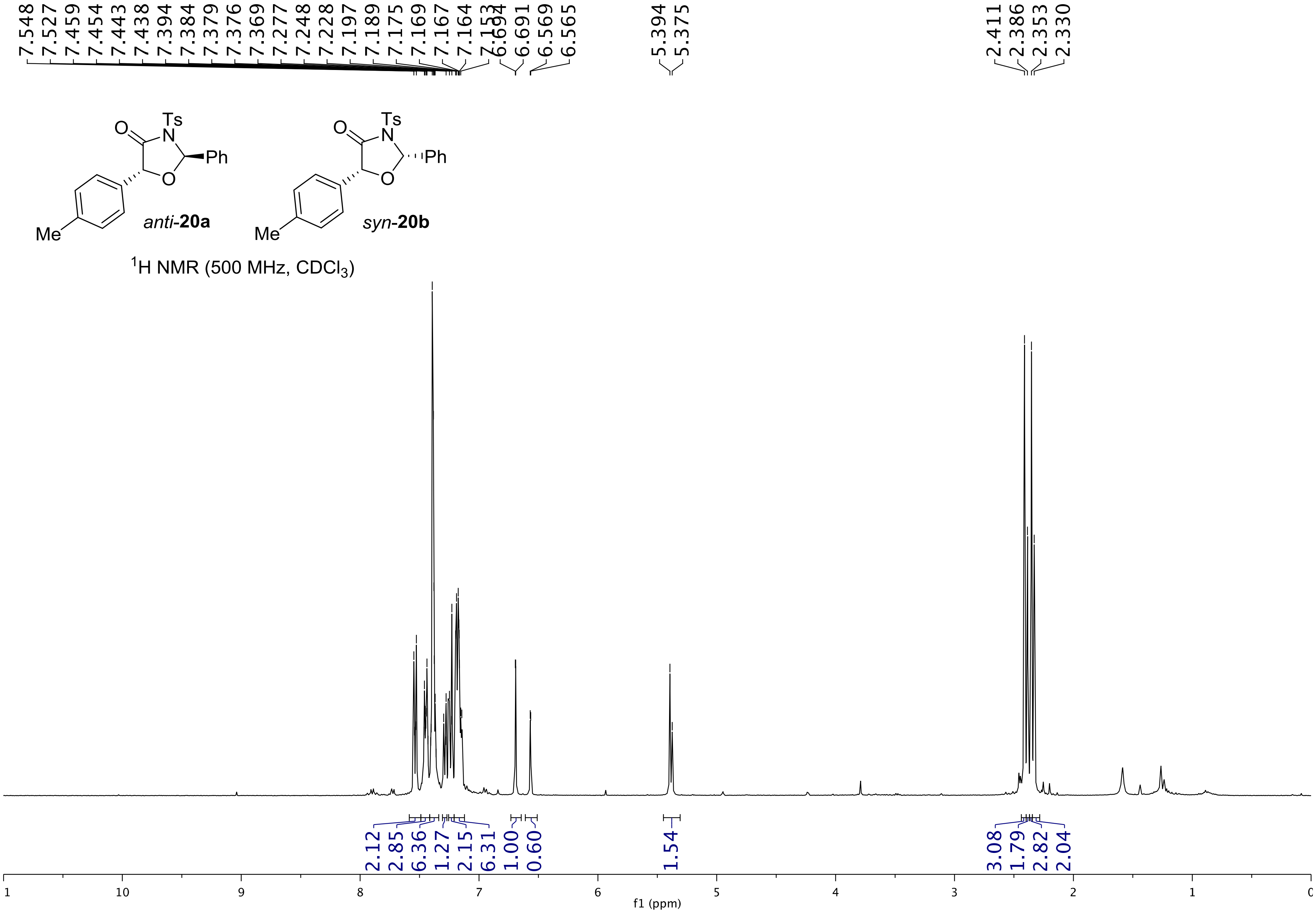
7.548
7.527
7.459
7.454
7.443
7.438
7.394
7.384
7.379
7.376
7.369
7.277
7.248
7.228
7.197
7.189
7.175
7.169
7.167
7.164
7.153
6.694
6.691
6.569
6.565
5.394
5.375
2.411
2.386
2.353
2.330
Ts
N
O
O
Ph
Me
anti-20a
syn-20b
^{1}H NMR (500 MHz, $CDCl_3$)
2.12
2.85
6.36
1.27
2.15
6.31
1.00
0.60
1.54
3.08
1.79
2.82
2.04
f1 (ppm)

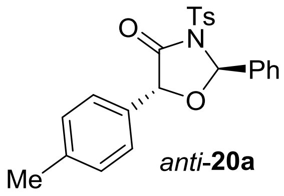

anti-**20a**

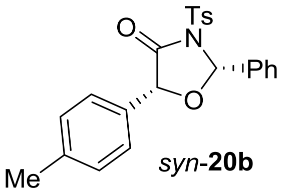

syn-**20b**

$^{13}C\{^1H\}$ NMR (125 MHz, $CDCl_3$)

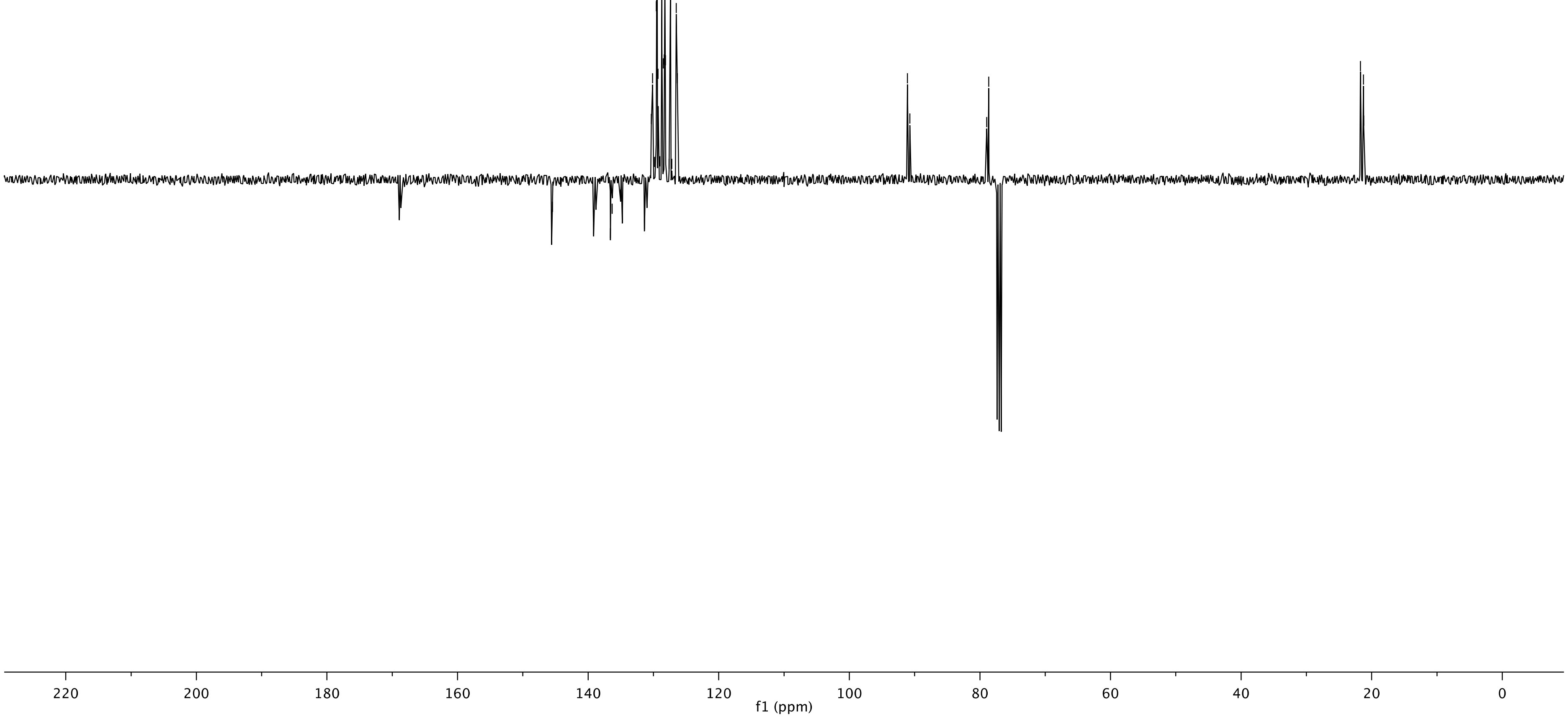

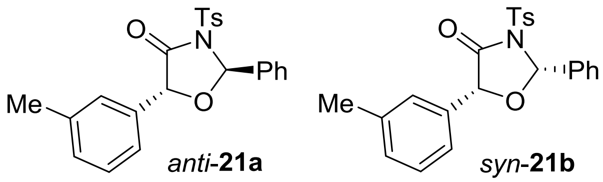

^1H NMR (500 MHz, $CDCl_3$)

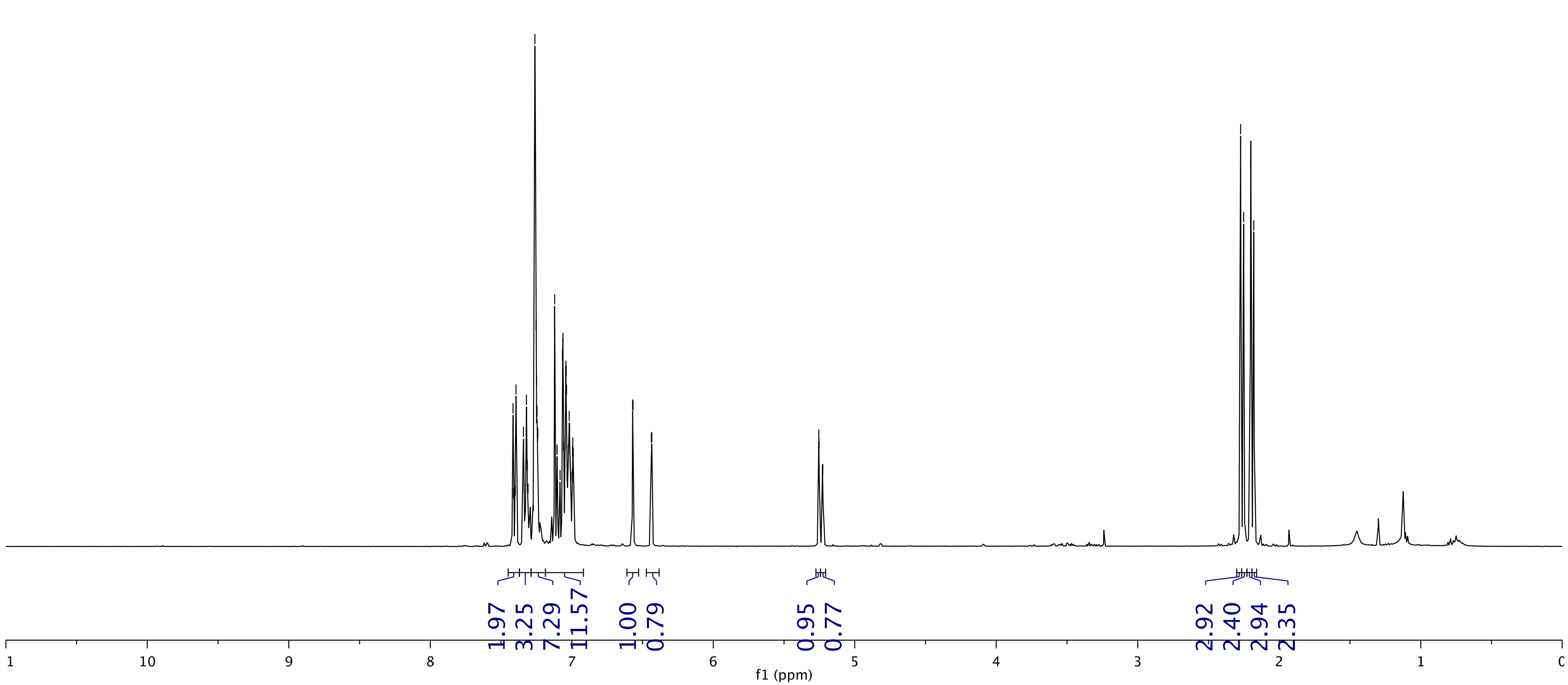

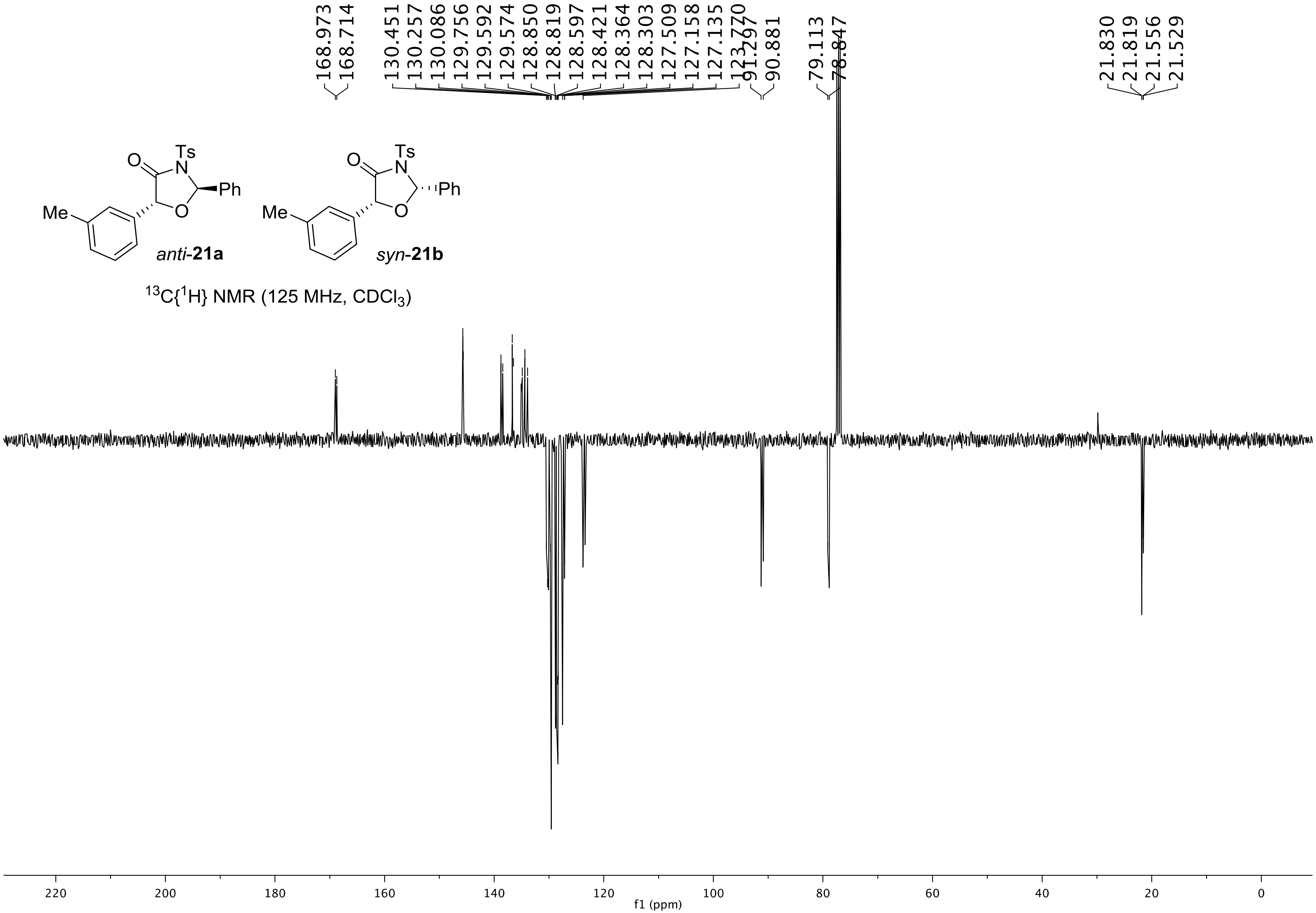
168.973
168.714
130.451
130.257
130.086
129.756
129.592
129.574
128.850
128.819
128.597
128.421
128.364
128.303
127.509
127.158
127.135
123.770
91.297
90.881
79.113
78.847
21.830
21.819
21.556
21.529
Ts
O
N
Ph
Me
anti-21a
syn-21b
$^{13}C\{^1H\}$ NMR (125 MHz, $CDCl_3$)
220
200
180
160
140
120
100
80
60
40
20
0
f1 (ppm)

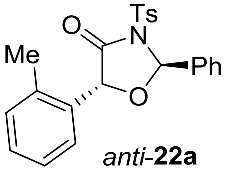

anti-**22a**

^{1}H NMR (500 MHz, $CDCl_3$)

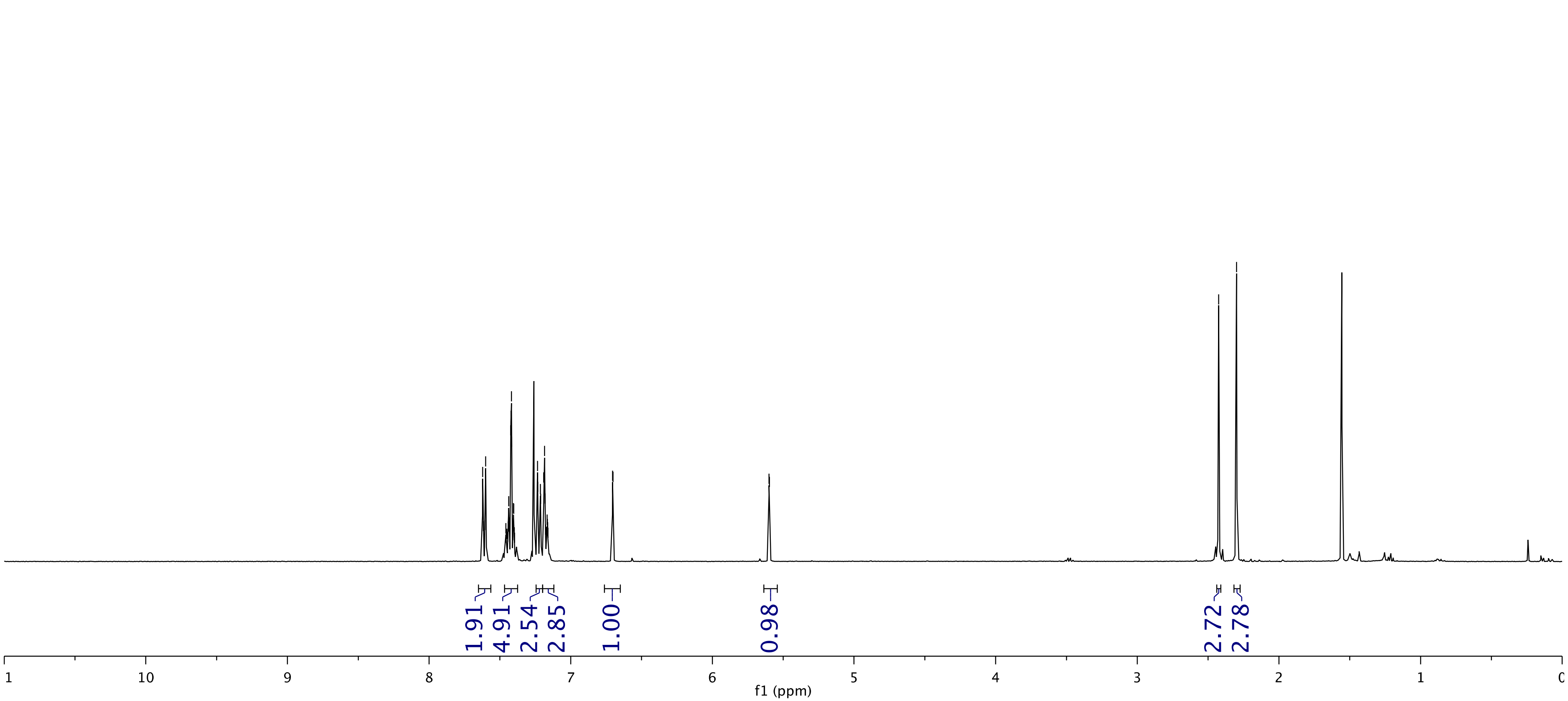

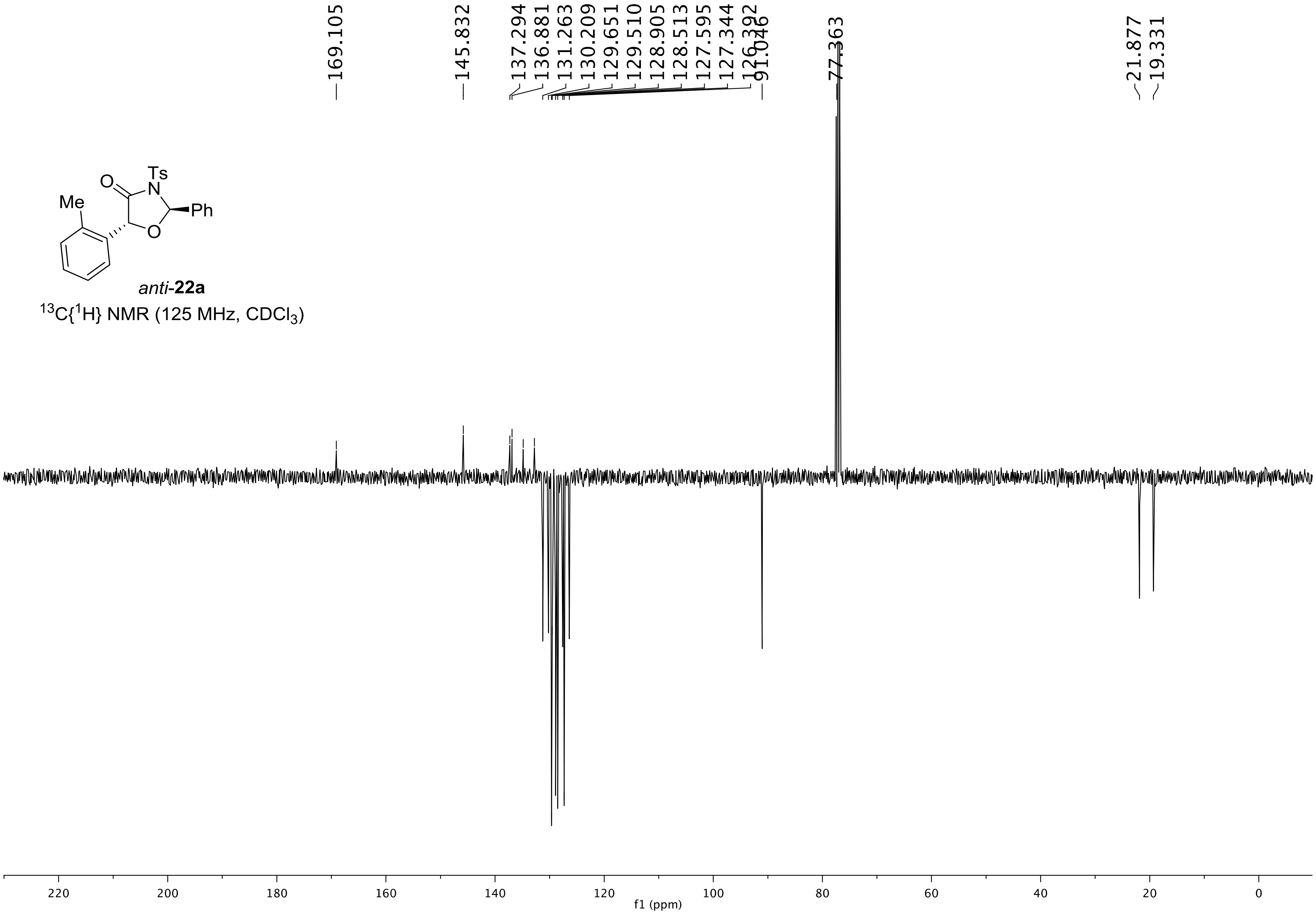
169.105
145.832
137.294
136.881
131.263
130.209
129.651
129.510
128.905
128.513
127.595
127.344
126.392
91.046
77.363
21.877
19.331
Ts
O
N
Me
Ph
O
anti-22a
$^{13}C\{^1H\}$ NMR (125 MHz, $CDCl_3$)
f1 (ppm)

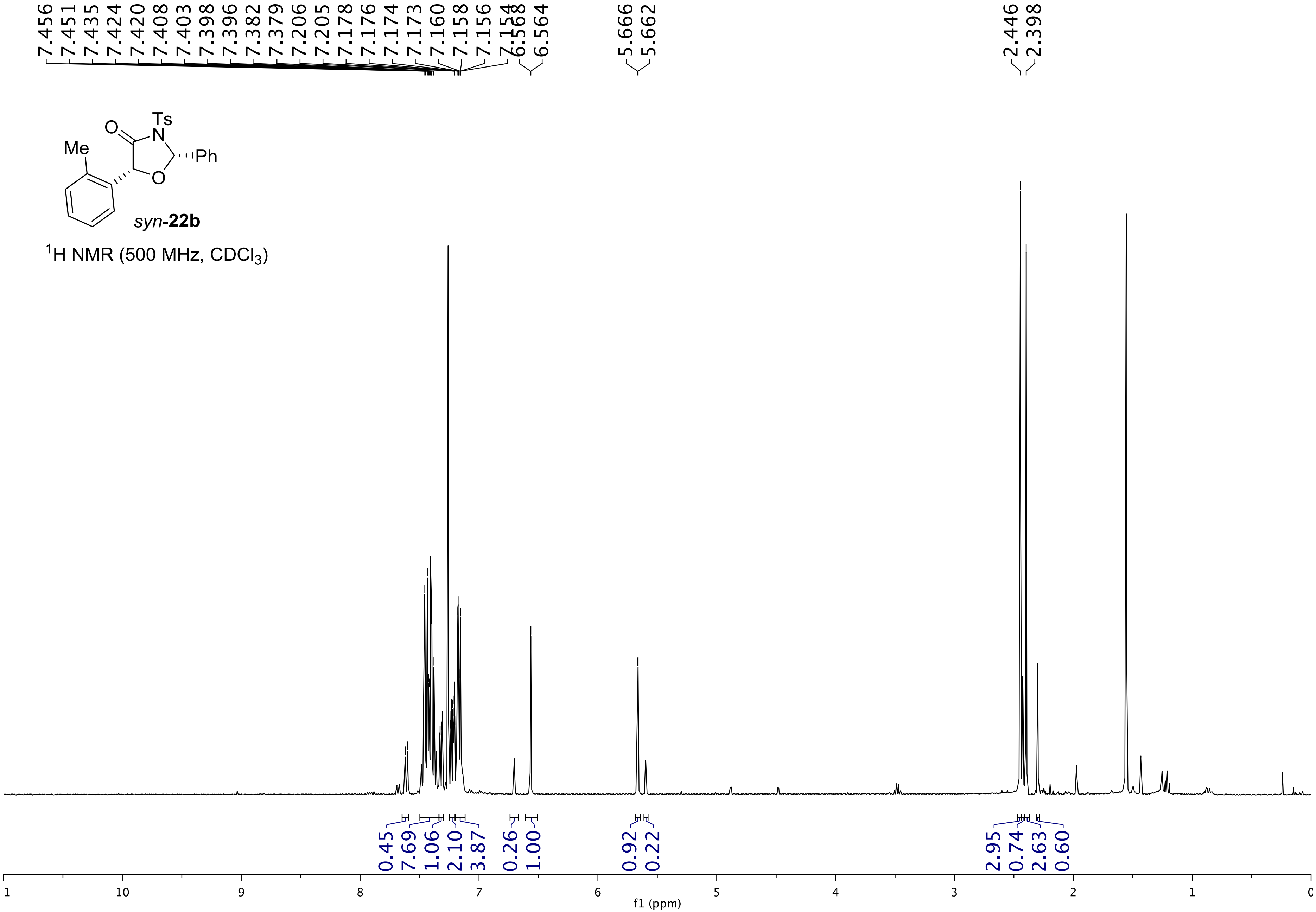
7.456
7.451
7.435
7.424
7.420
7.408
7.403
7.398
7.396
7.382
7.379
7.206
7.205
7.178
7.176
7.174
7.173
7.160
7.158
7.156
7.154
6.568
6.564
5.666
5.662
2.446
2.398
O
Ts
N
Me
Ph
O
syn-22b
^{1}H NMR (500 MHz, $CDCl_3$)
0.45
7.69
1.06
2.10
3.87
0.26
1.00
0.92
0.22
2.95
0.74
2.63
0.60
10
9
8
7
6
5
4
3
2
1
f1 (ppm)

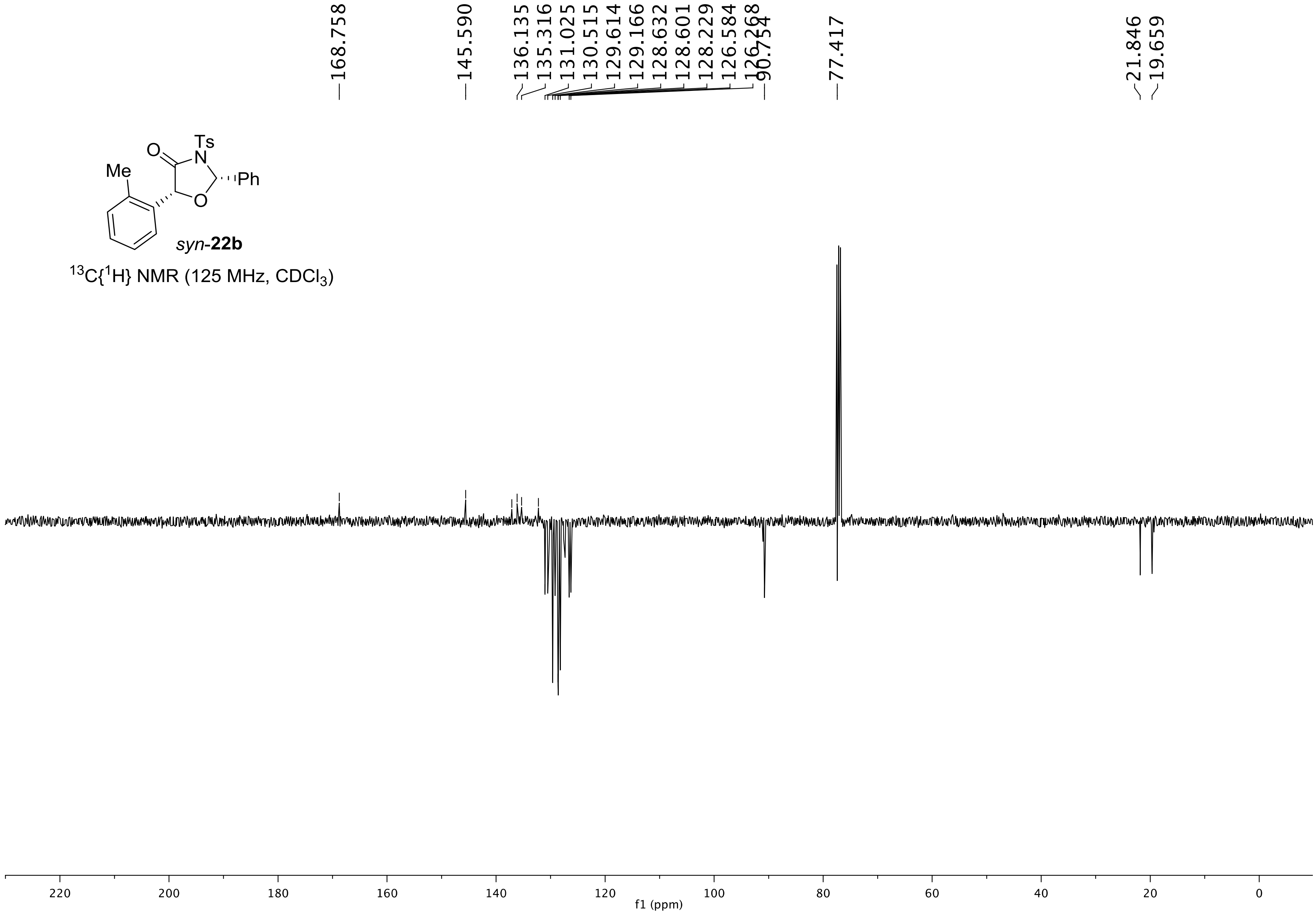
168.758
145.590
136.135
135.316
131.025
130.515
129.614
129.166
128.632
128.601
128.229
126.584
126.268
90.754
77.417
21.846
19.659
O
Ts
N
Me
Ph
O
syn-22b
$^{13}C\{^1H\}$ NMR (125 MHz, $CDCl_3$)
220
200
180
160
140
120
100
80
60
40
20
0
f1 (ppm)

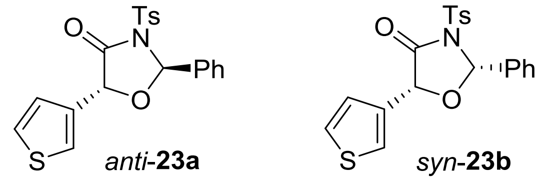

anti-**23a** *syn*-**23b**

^{1}H NMR (500 MHz, CDCl3)

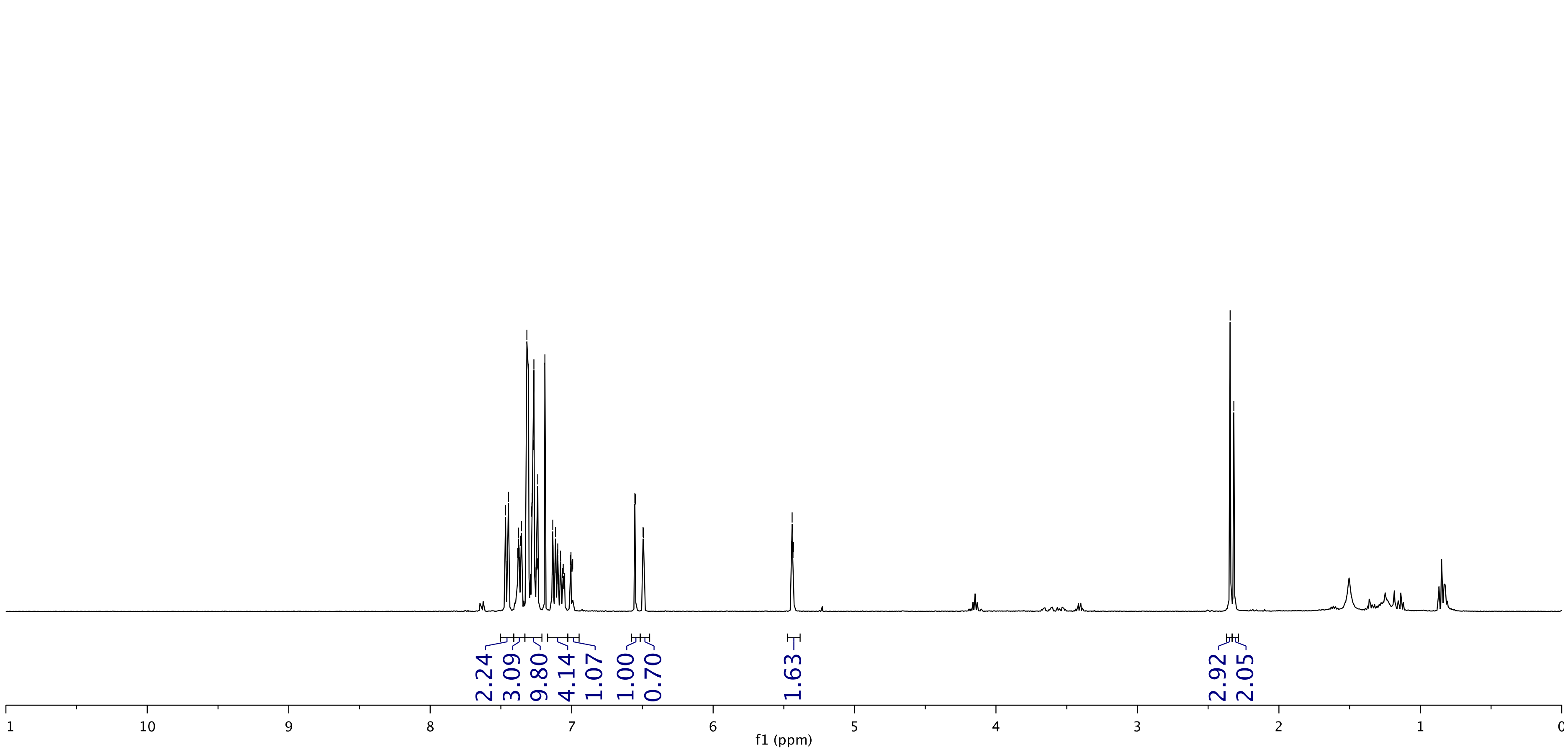

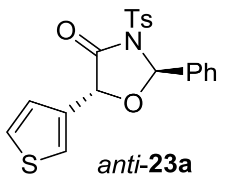

anti-**23a**

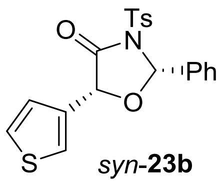

syn-**23b**

$^{13}C\{^1H\}$ NMR (125 MHz, $CDCl_3$)

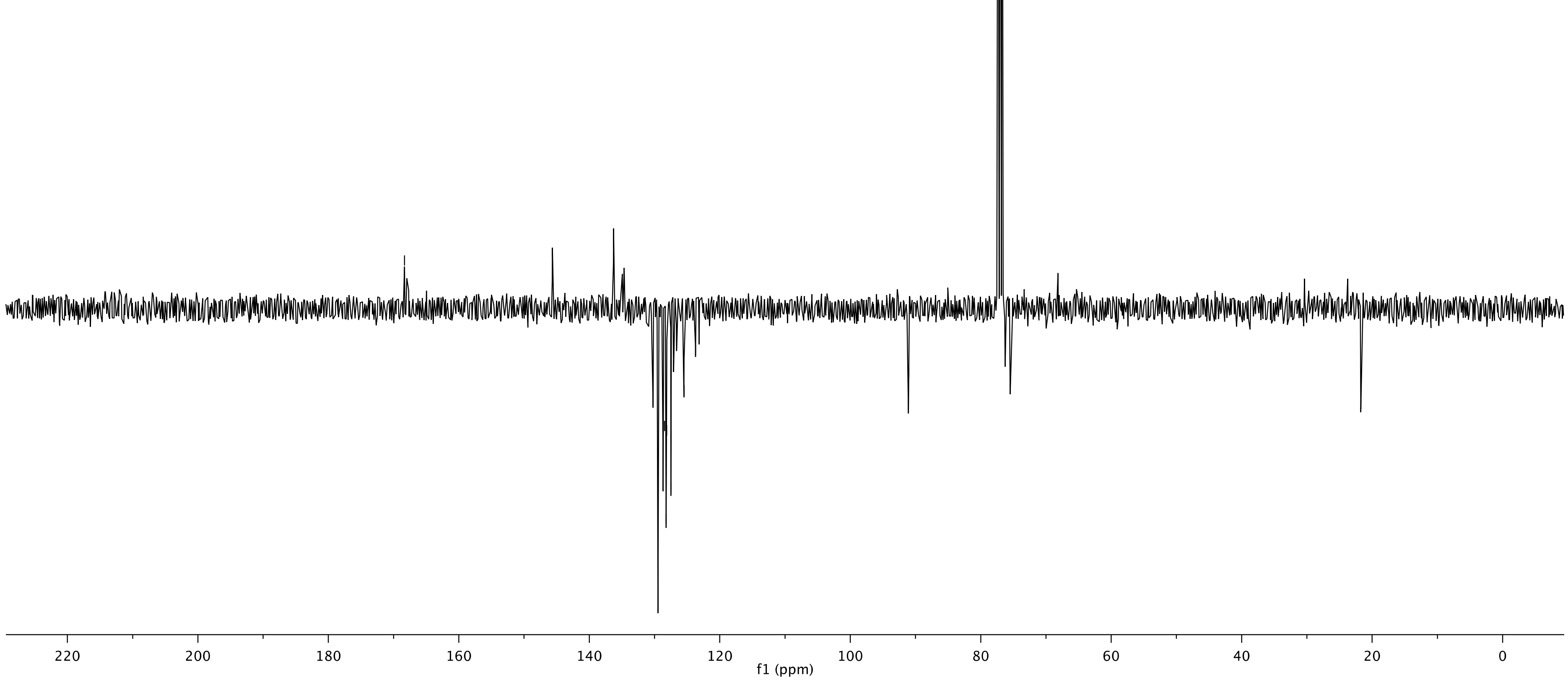

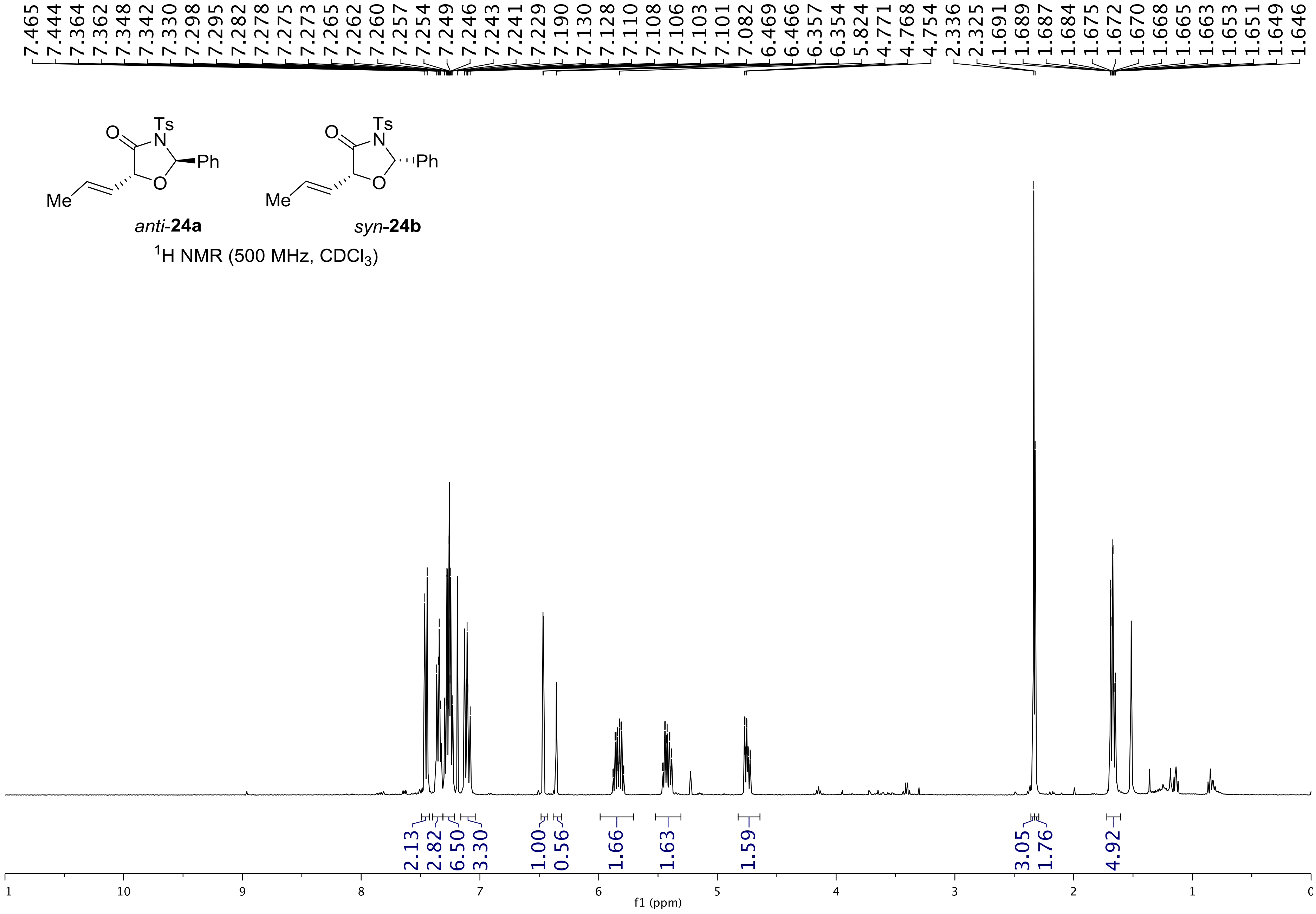
O
Ts
N
Ph
Me
O
anti-24a
syn-24b
¹H NMR (500 MHz, CDCl₃)
f1 (ppm)

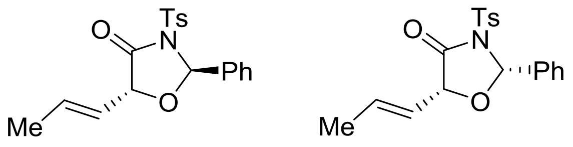

anti-**24a** *syn*-**24b**

$^{13}C\{^1H\}$ NMR (125 MHz, $CDCl_3$)

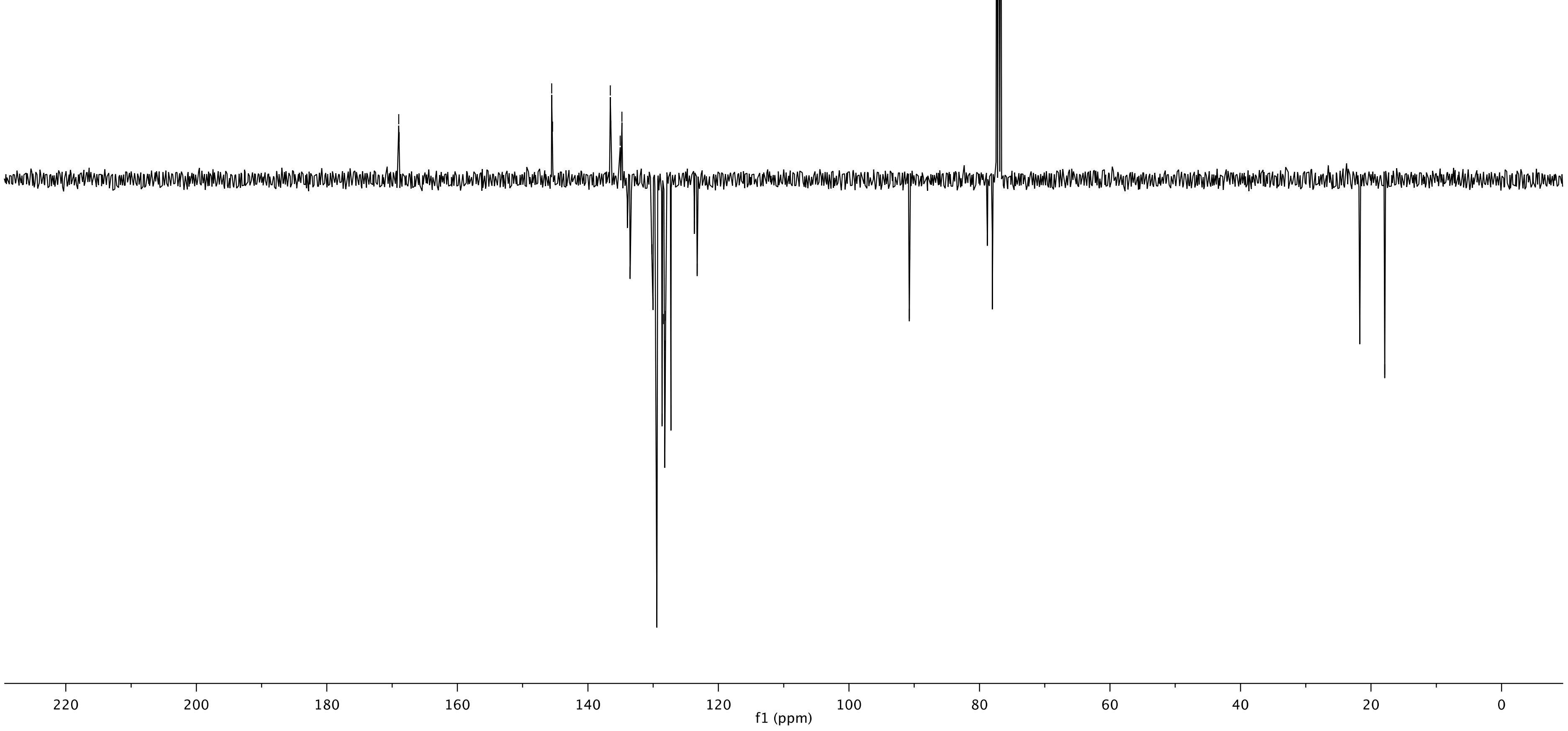

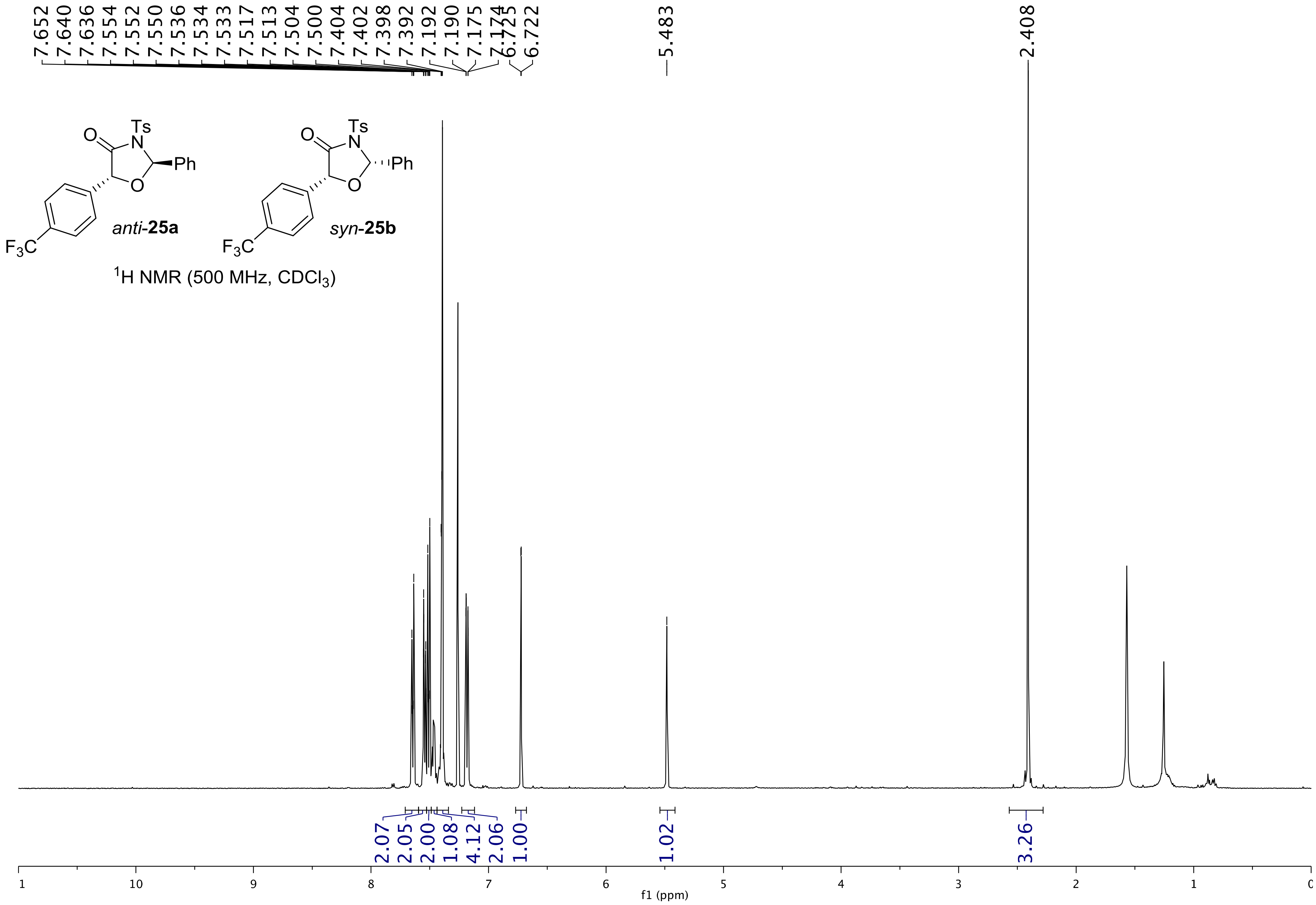
7.652
7.640
7.636
7.554
7.552
7.550
7.536
7.534
7.533
7.517
7.513
7.504
7.500
7.404
7.402
7.398
7.392
7.192
7.190
7.175
7.174
6.725
6.722
5.483
2.408
O
Ts
N
Ph
O
F3C
anti-25a
syn-25b
1H NMR (500 MHz, CDCl3)
2.07
2.05
2.00
1.08
4.12
2.06
1.00
1.02
3.26
f1 (ppm)

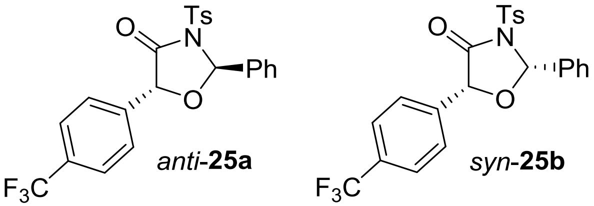

anti-**25a** *syn*-**25b**

$^{13}C\ \{^{1}H\}$ NMR (125 MHz, $CDCl_3$)

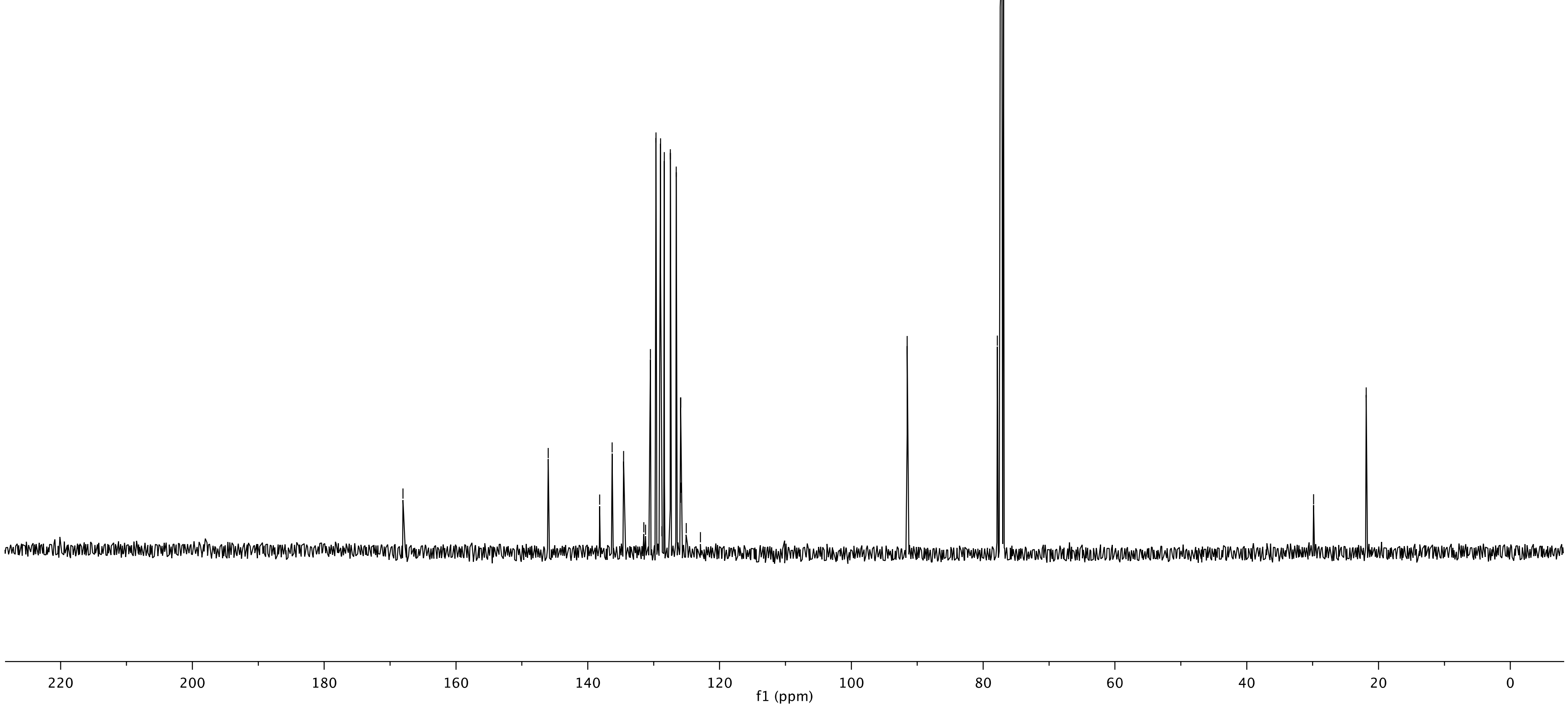

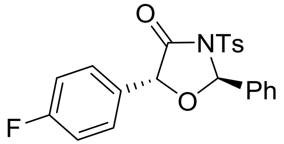

anti-**17a**

^{1}H NMR (400 MHz, $CDCl_3$)

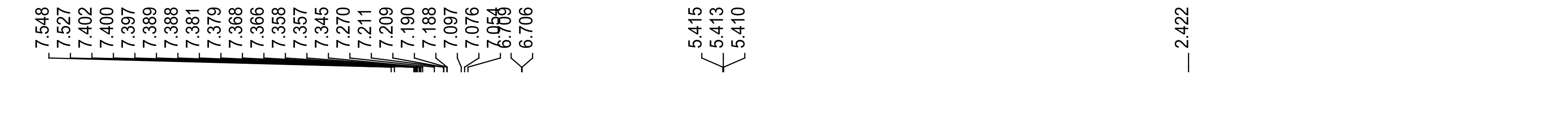

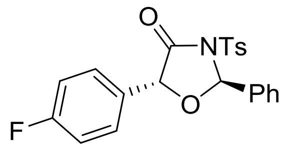

anti-**17a**

$^{13}C\{^1H\}$ NMR (100 MHz, $CDCl_3$)

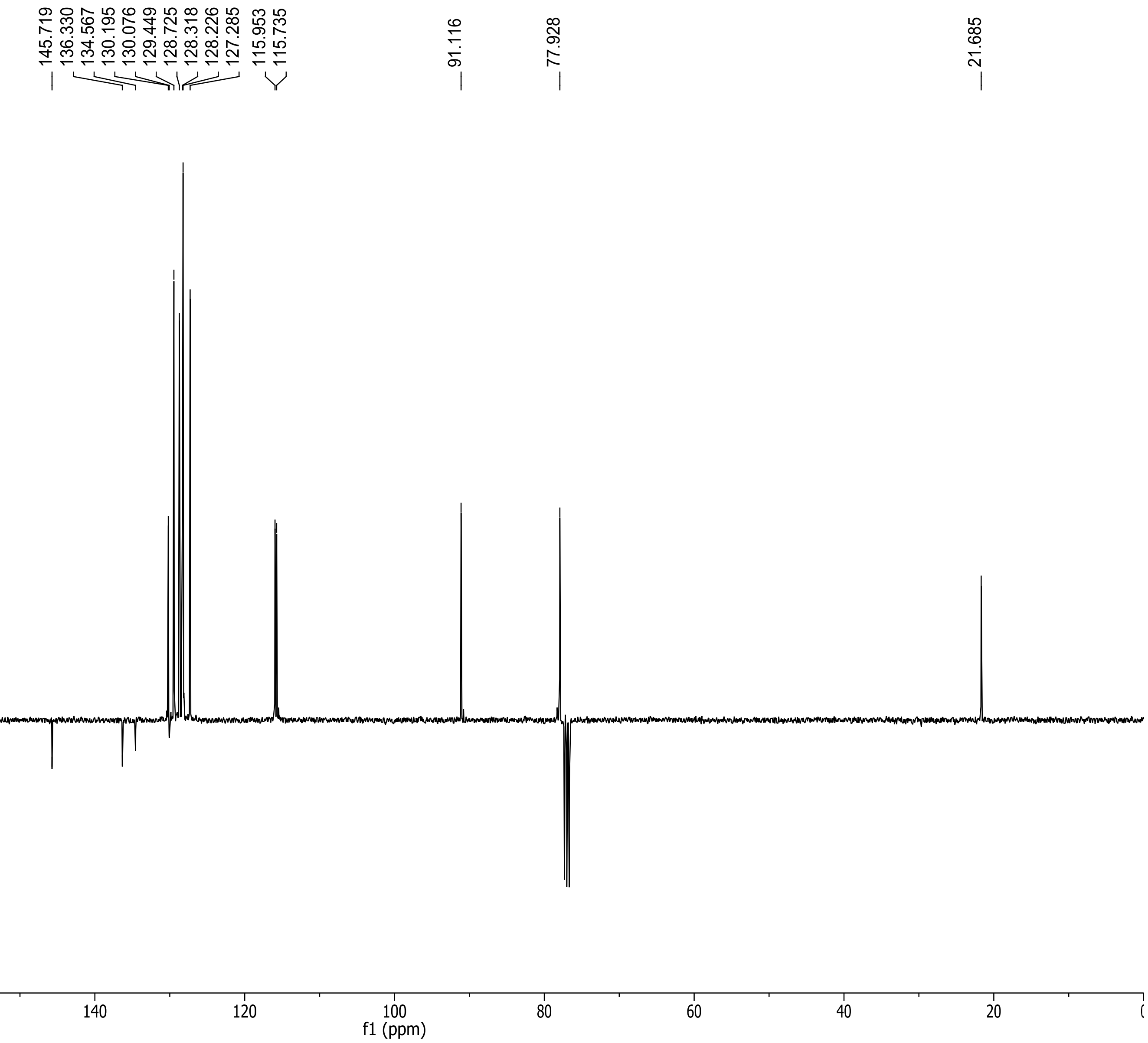

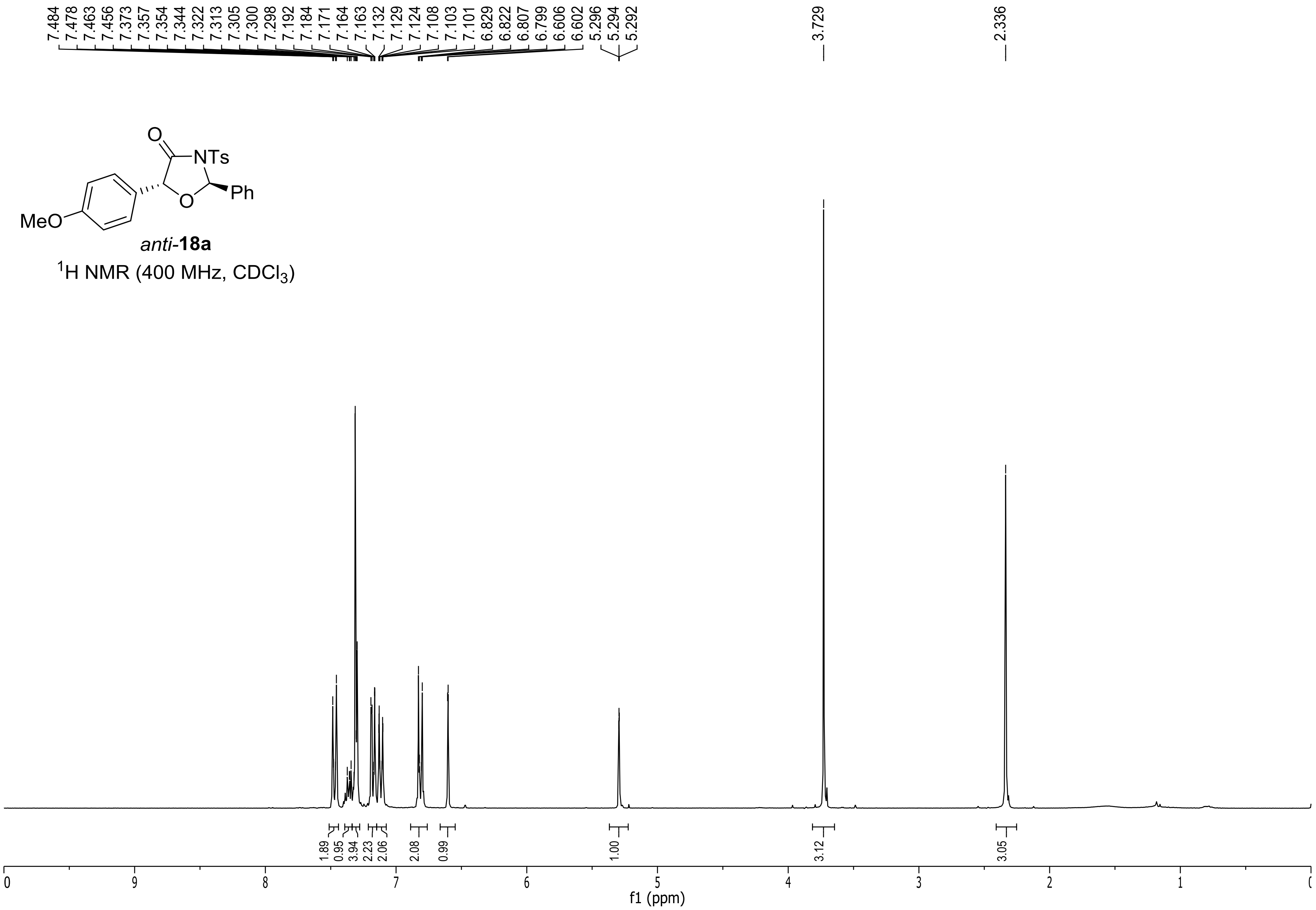

anti-**18a**

^{1}H NMR (400 MHz, $CDCl_3$)

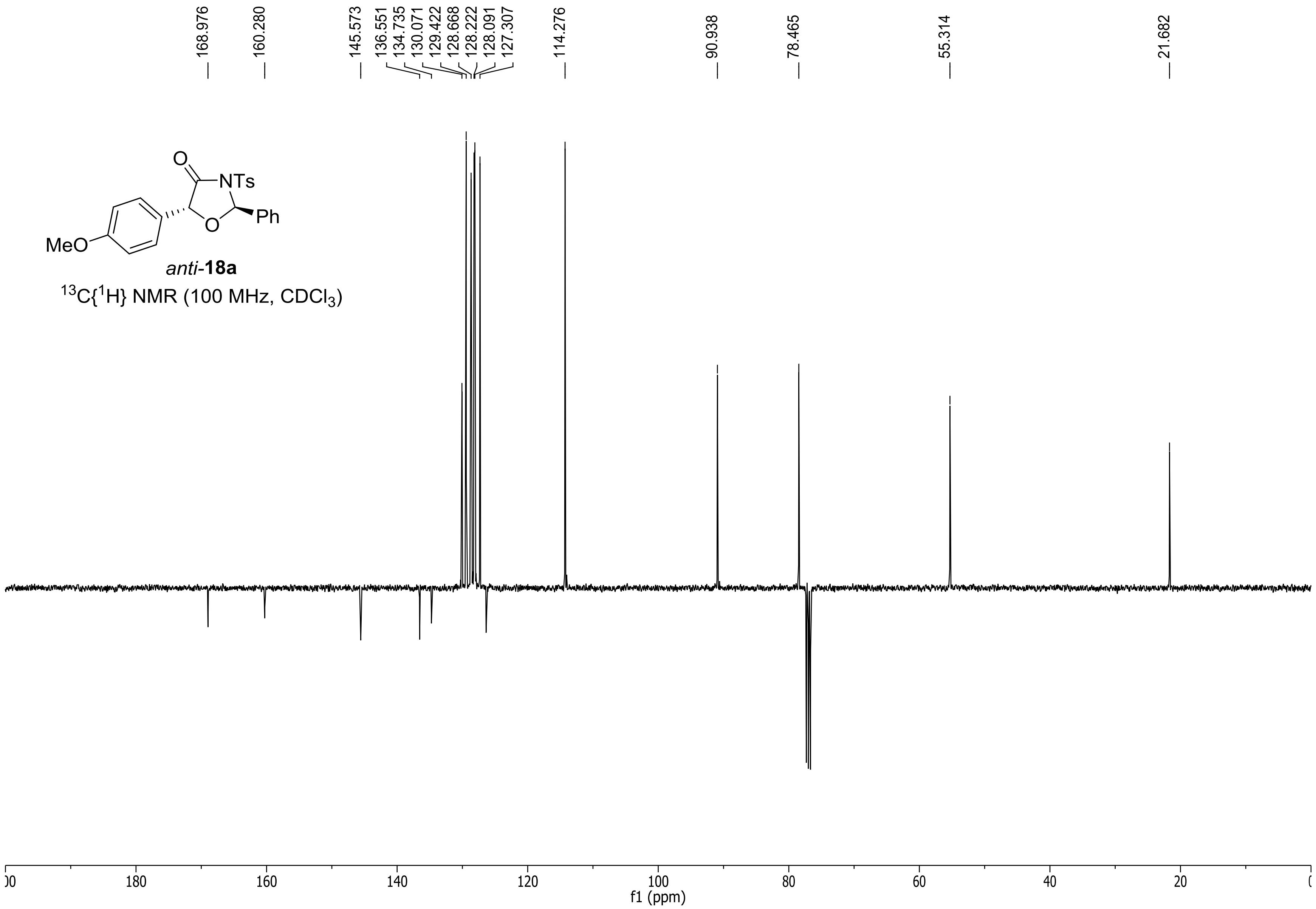
168.976
160.280
145.573
136.551
134.735
130.071
129.422
128.668
128.222
128.091
127.307
114.276
90.938
78.465
55.314
21.682
O
NTs
Ph
O
MeO
anti-18a
13C{1H} NMR (100 MHz, CDCl3)
180
160
140
120
100
80
60
40
20
f1 (ppm)

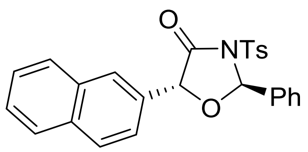

anti-**19a**

^{1}H NMR (400 MHz, $CDCl_3$)

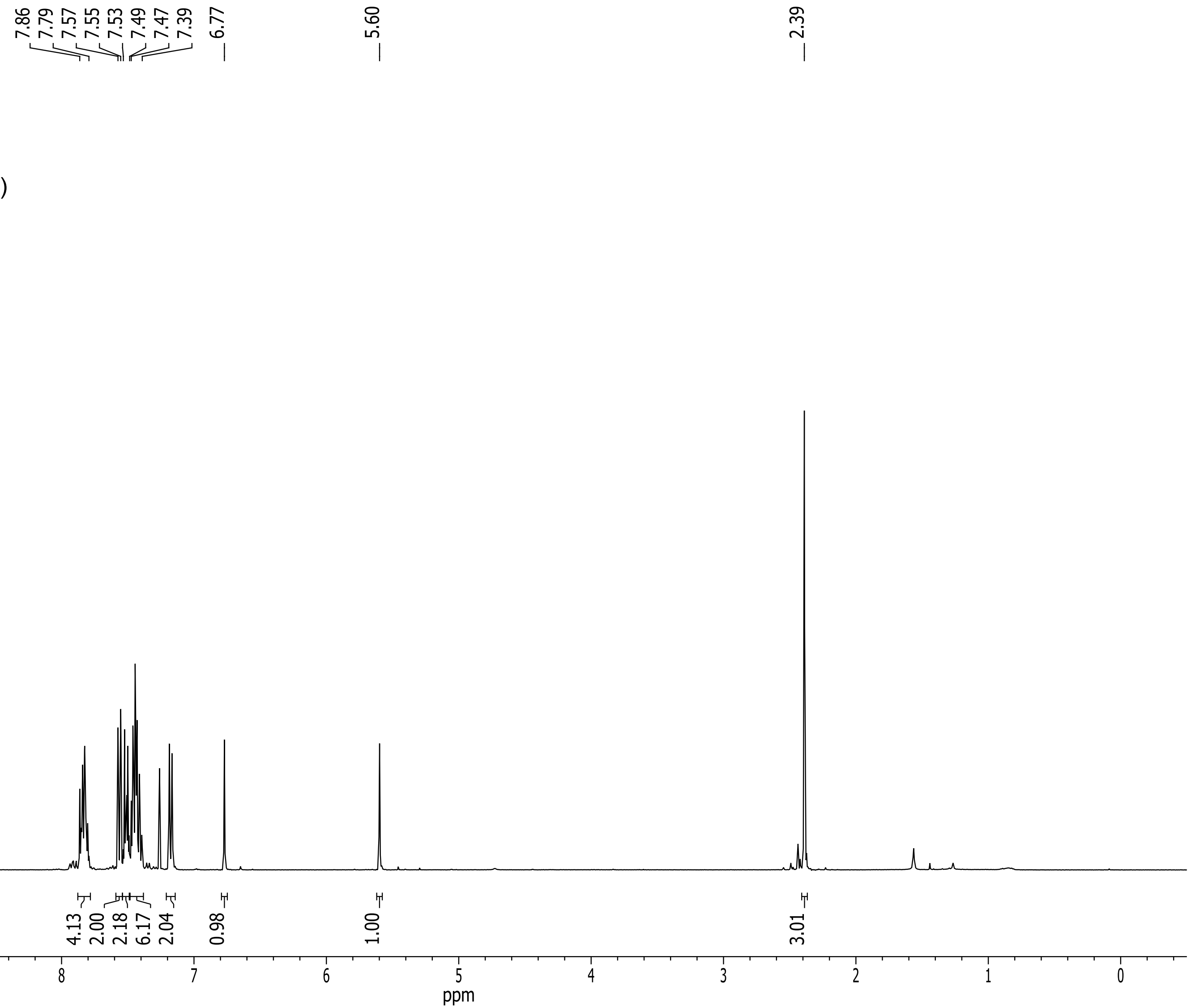

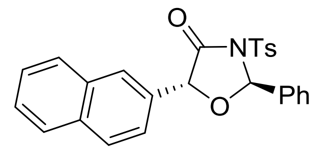

anti-**19a**

$^{13}C\{^{1}H\}$ NMR (100 MHz, $CDCl_3$)

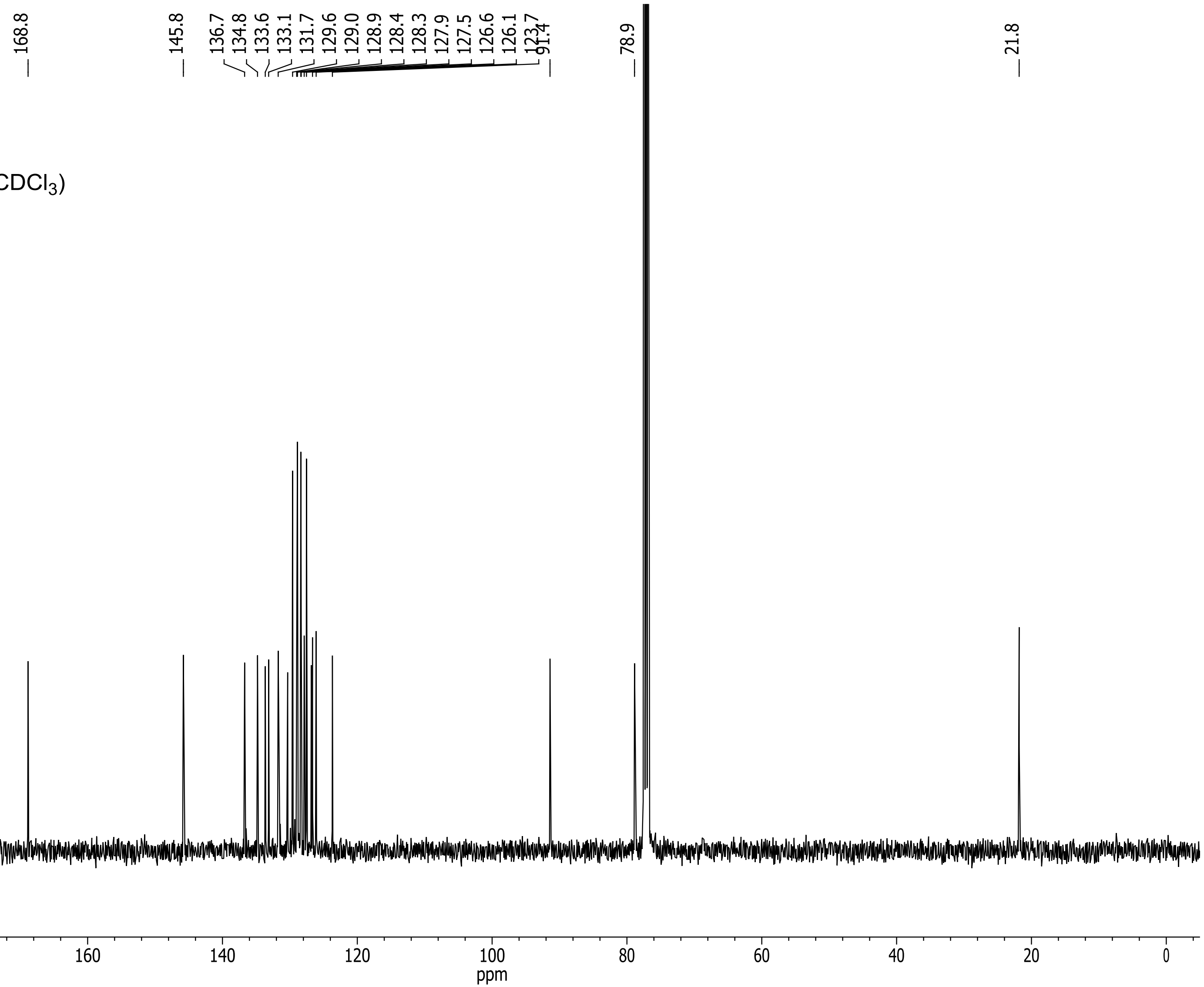

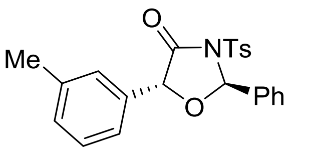

anti-**21a**

^{1}H NMR (400 MHz, $CDCl_3$)

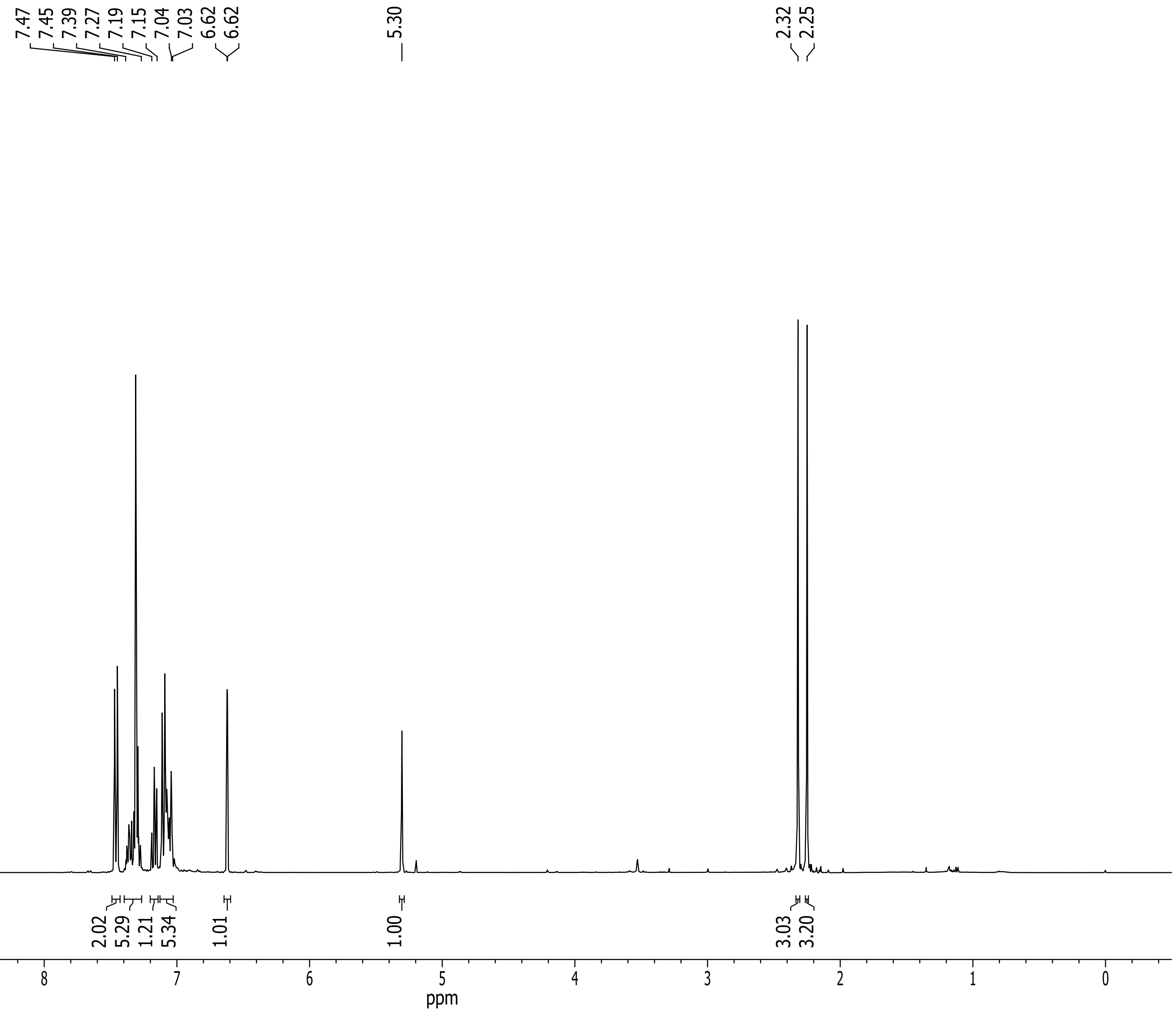

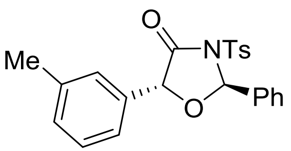

anti-**21a**

$^{13}C\{^{1}H\}$ NMR (100 MHz, $CDCl_3$)

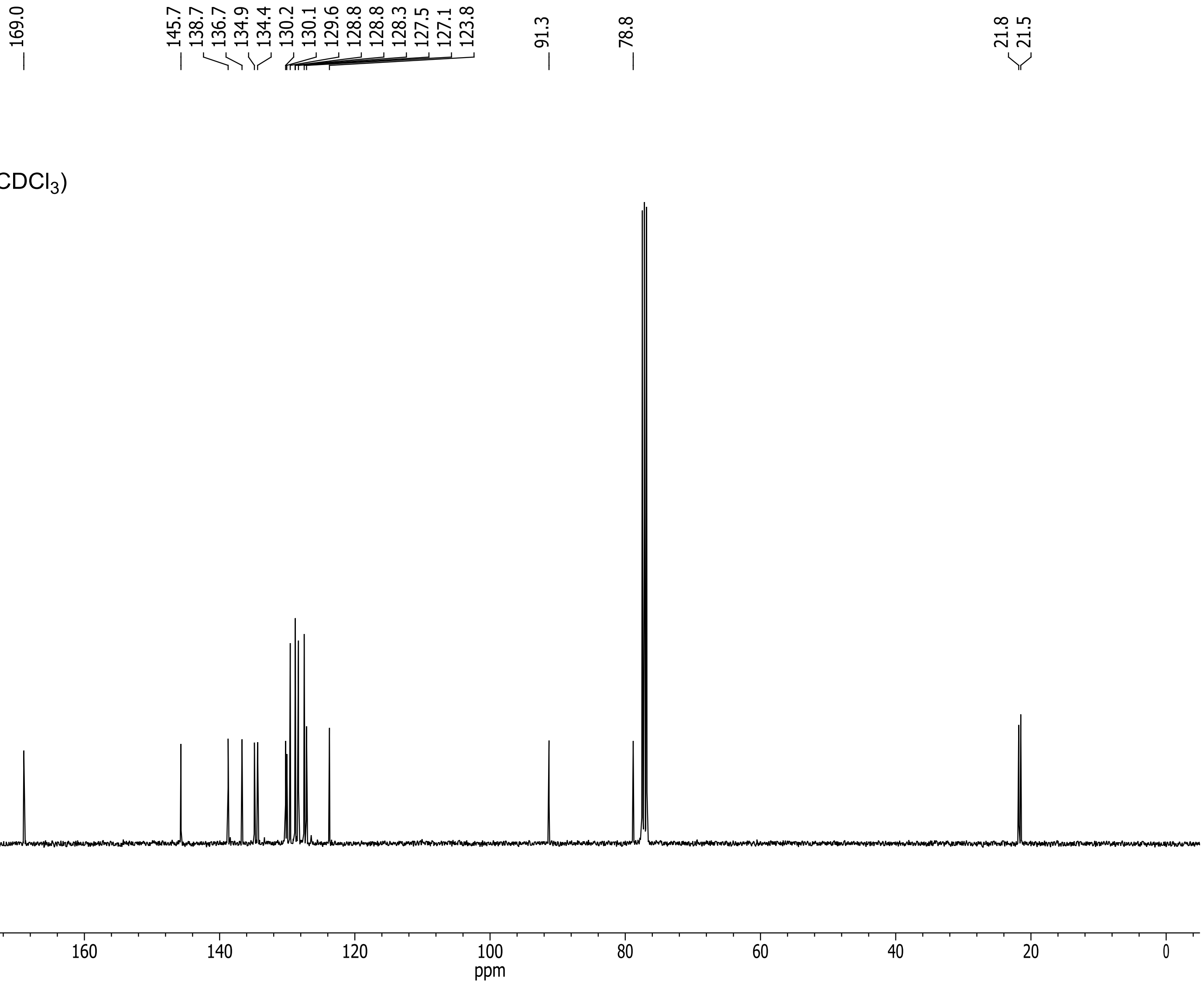

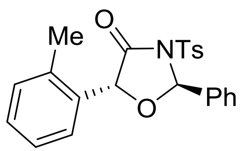

anti-**22a**

^{1}H NMR (400 MHz, $CDCl_3$)

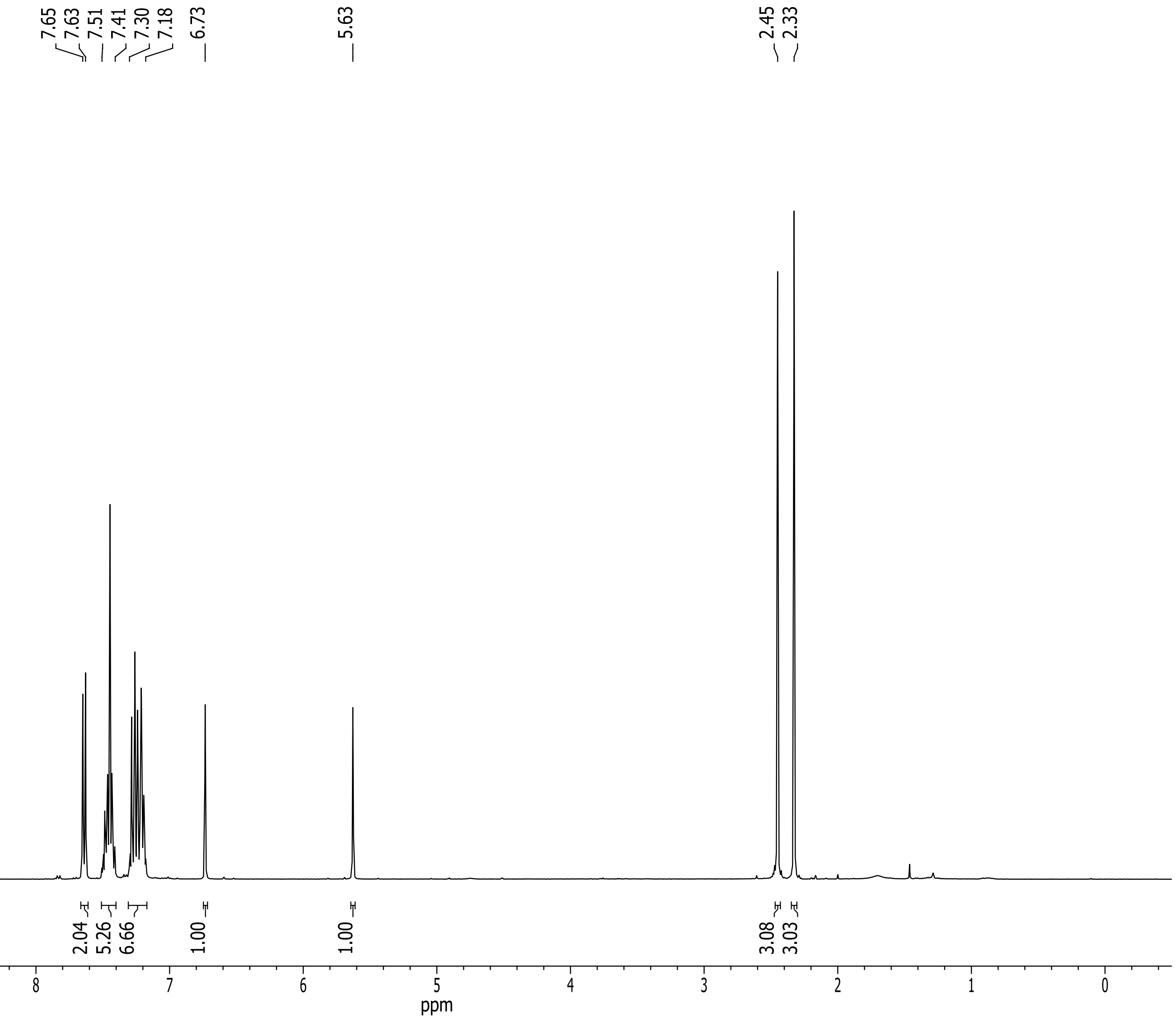

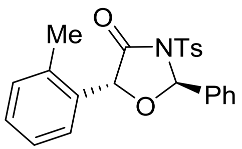

anti-**22a**

$^{13}C\{^{1}H\}$ NMR (100 MHz, $CDCl_3$)

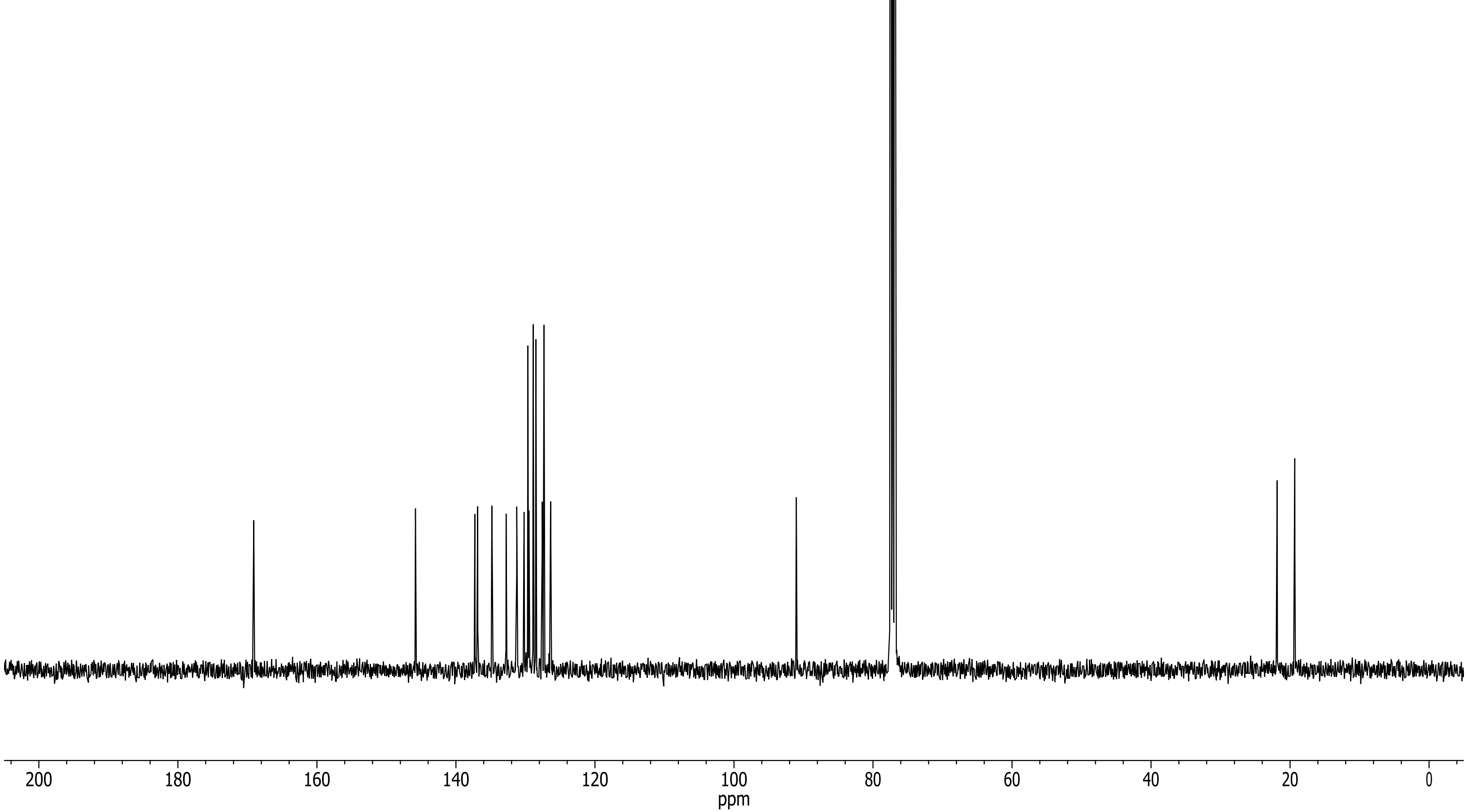

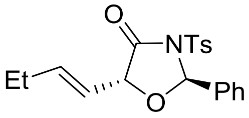

anti-**30a**

^{1}H NMR (400 MHz, $CDCl_3$)

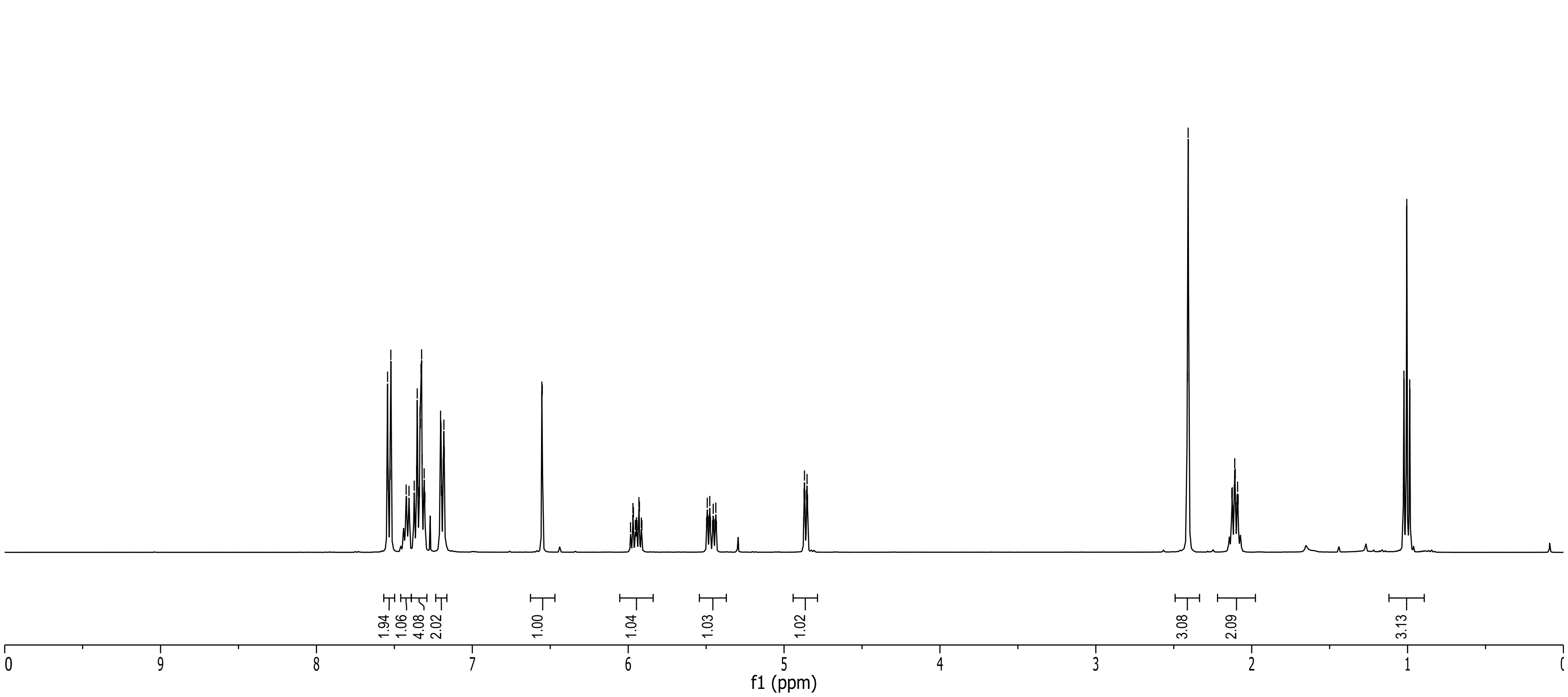

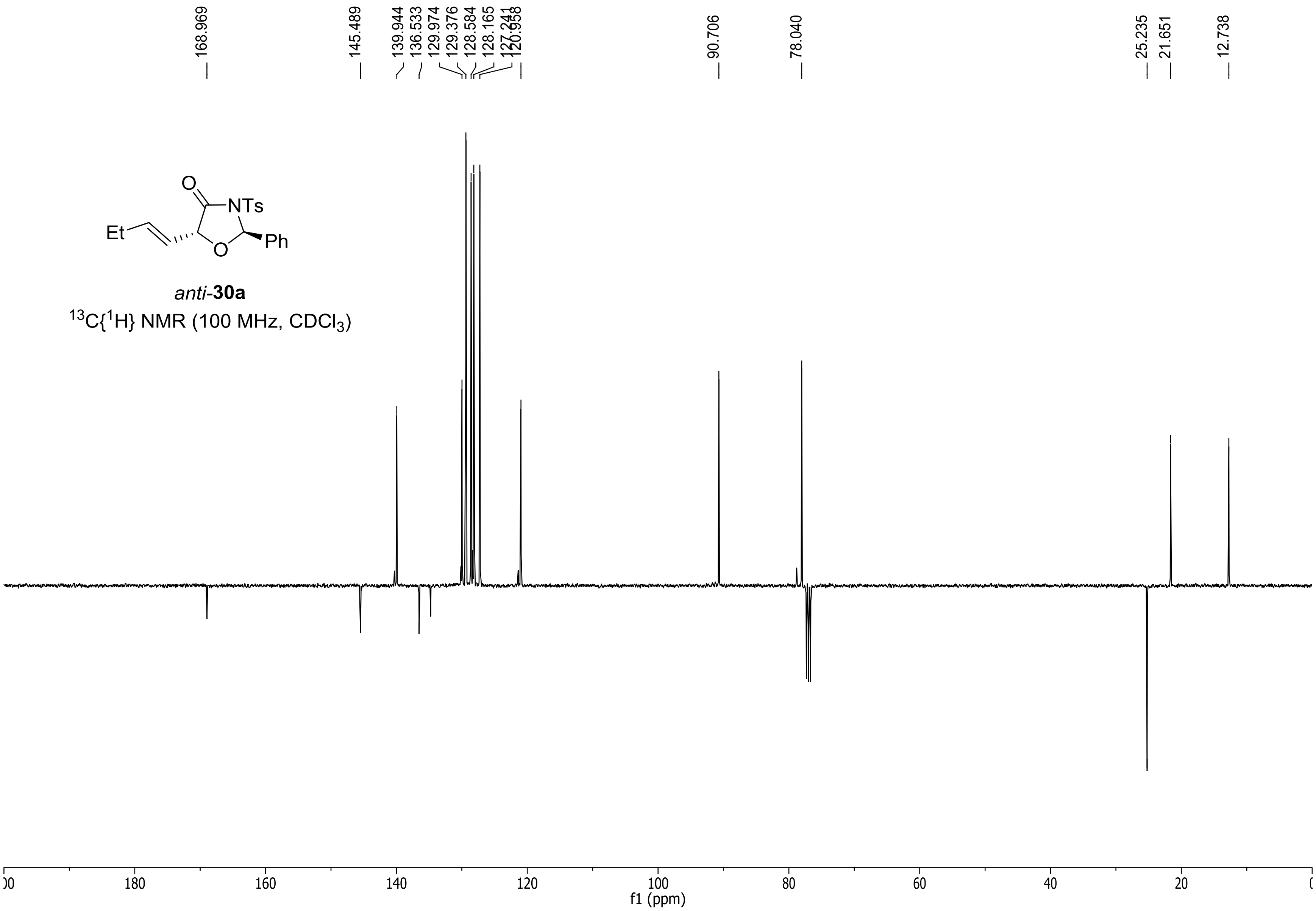
168.969
145.489
139.944
136.533
129.974
129.376
128.584
128.165
127.241
120.958
90.706
78.040
25.235
21.651
12.738
O
NTs
Et
O
Ph
anti-30a
$^{13}C\{^{1}H\}$ NMR (100 MHz, $CDCl_3$)
180
160
140
120
100
80
60
40
20
f1 (ppm)

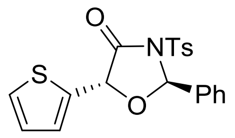

anti-**31a**

^{1}H NMR (500 MHz, $CDCl_3$)

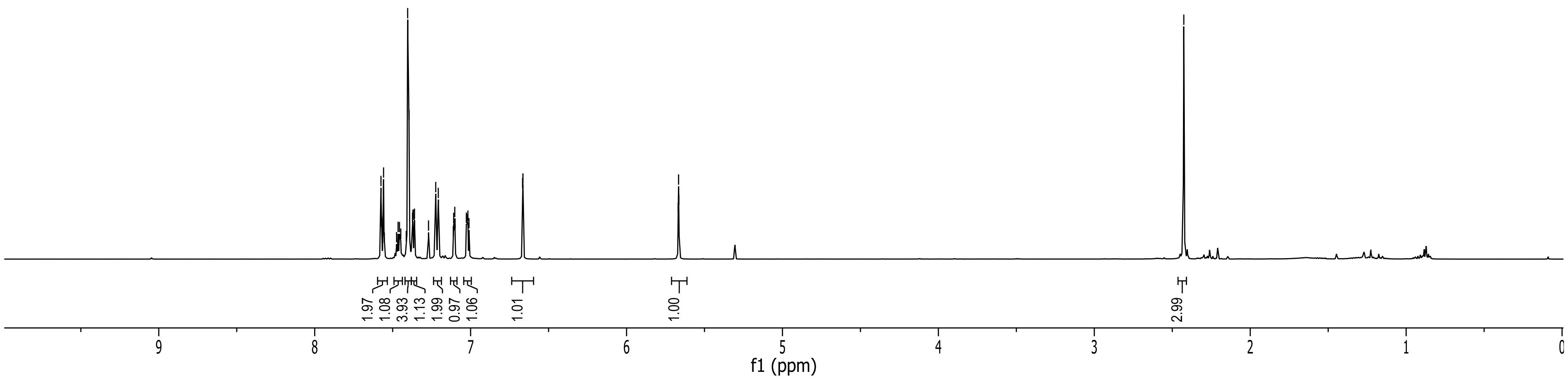

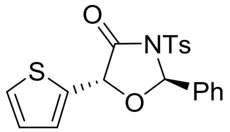

anti-**30a**
$^{13}C\{^{1}H\}$ NMR (100 MHz, $CDCl_3$)

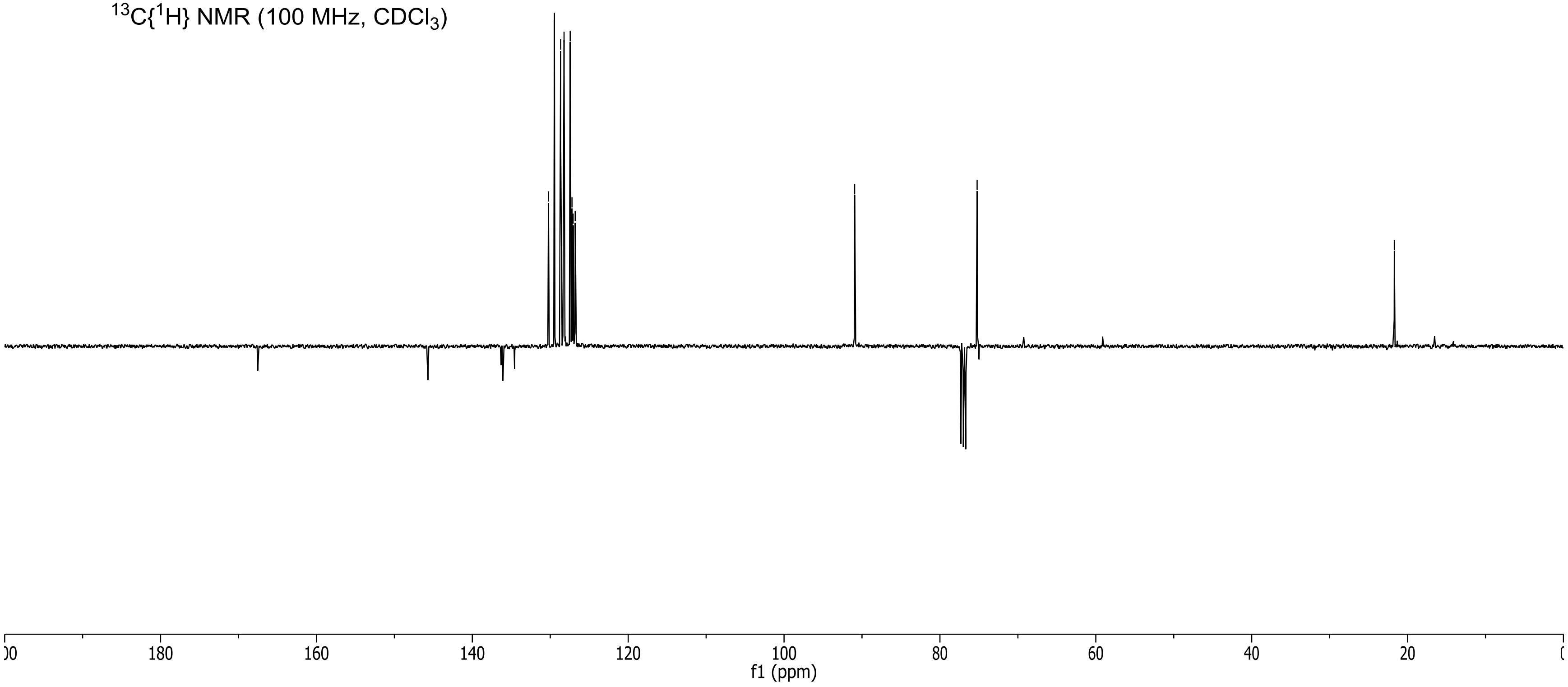

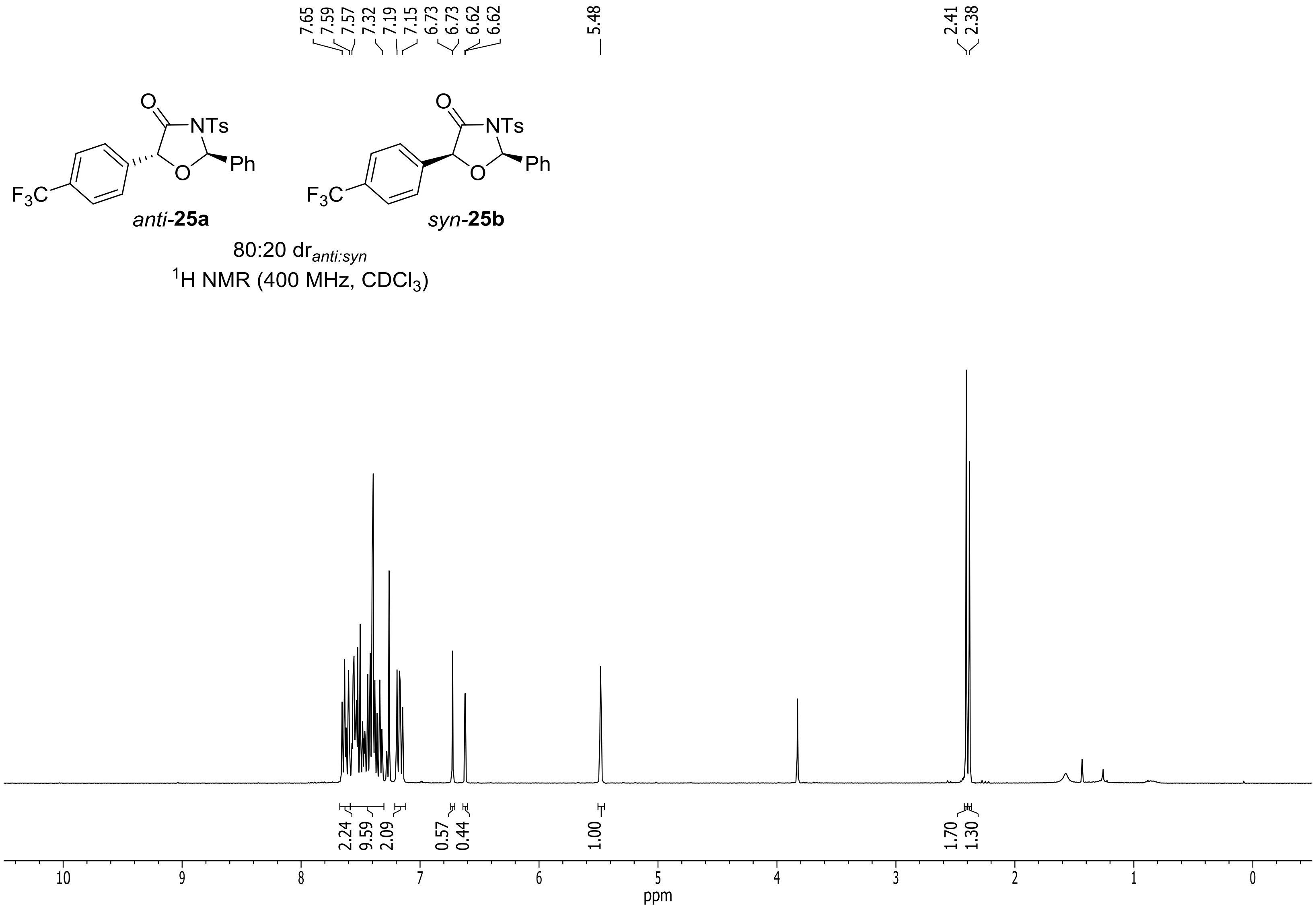

80:20 $dr_{anti:syn}$

^{1}H NMR (400 MHz, $CDCl_3$)

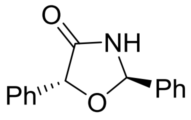

32

^1H NMR (500 MHz, $CDCl_3$)

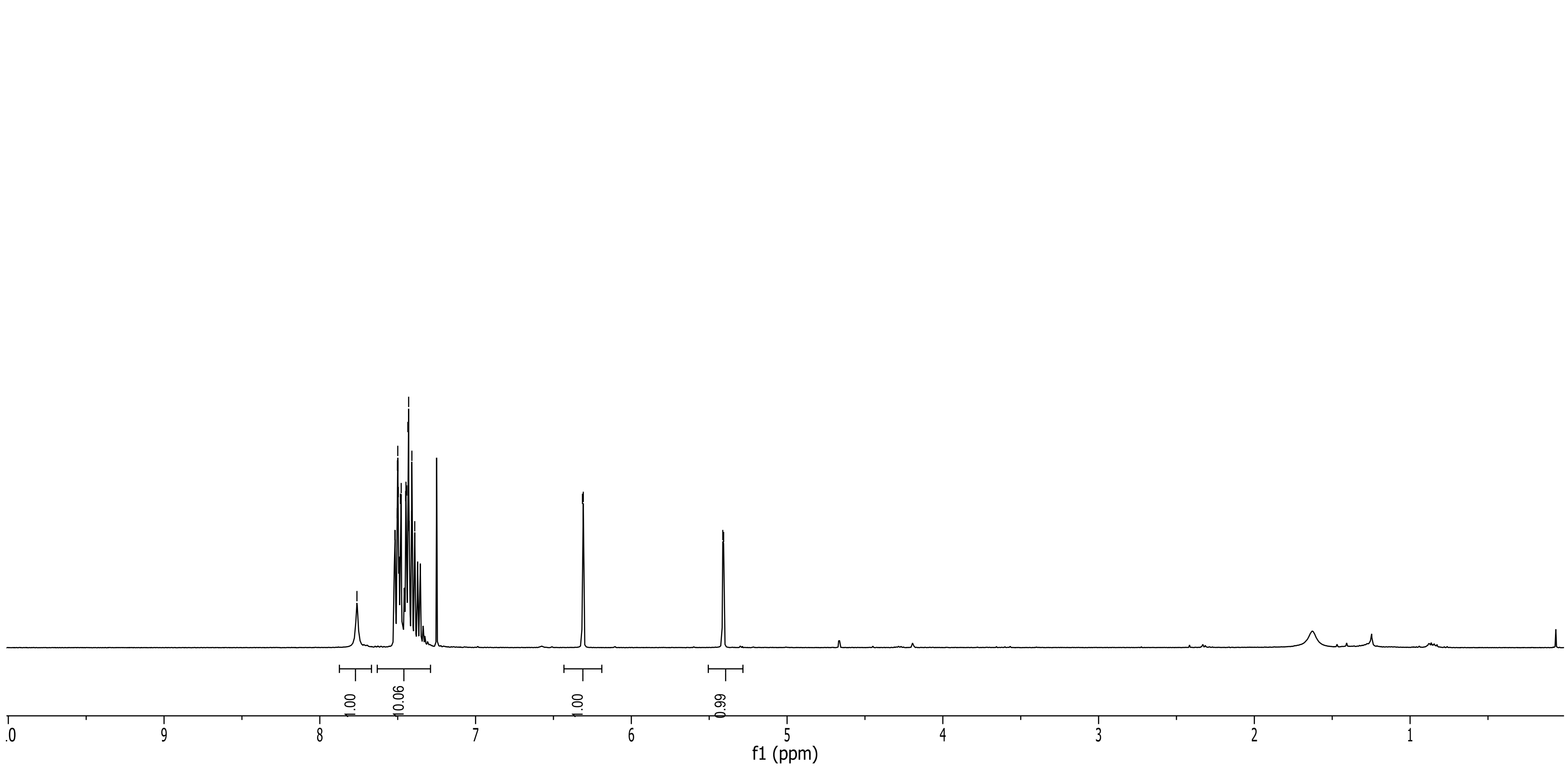

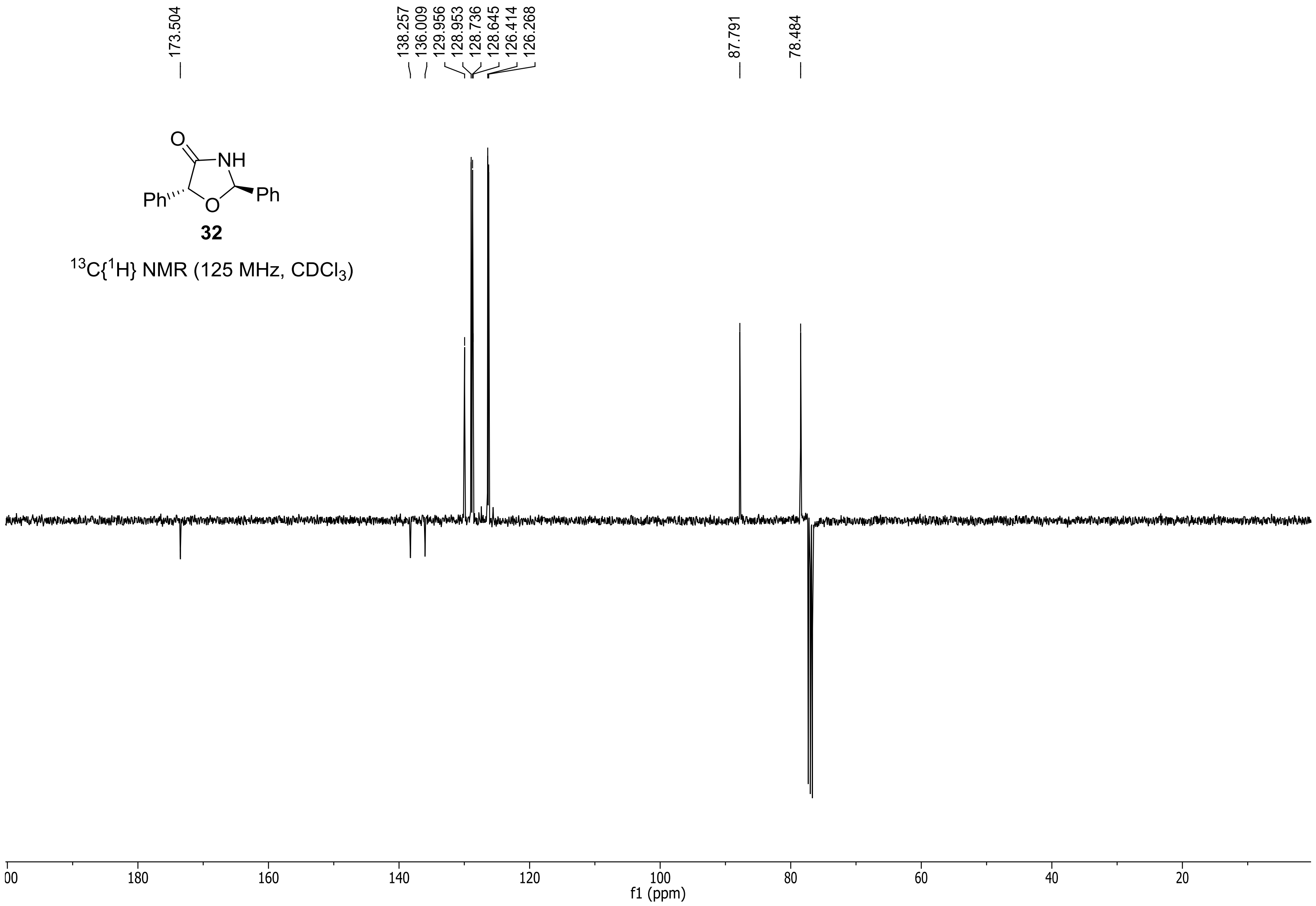
173.504
138.257
136.009
129.956
128.953
128.736
128.645
126.414
126.268
87.791
78.484
O
NH
Ph
Ph
O
32
$^{13}C\{^{1}H\}$ NMR (125 MHz, $CDCl_3$)
00
180
160
140
120
100
80
60
40
20
f1 (ppm)

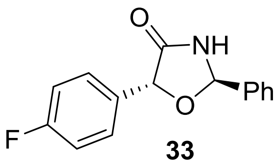

33

^{1}H NMR (300 MHz, $CDCl_3$)

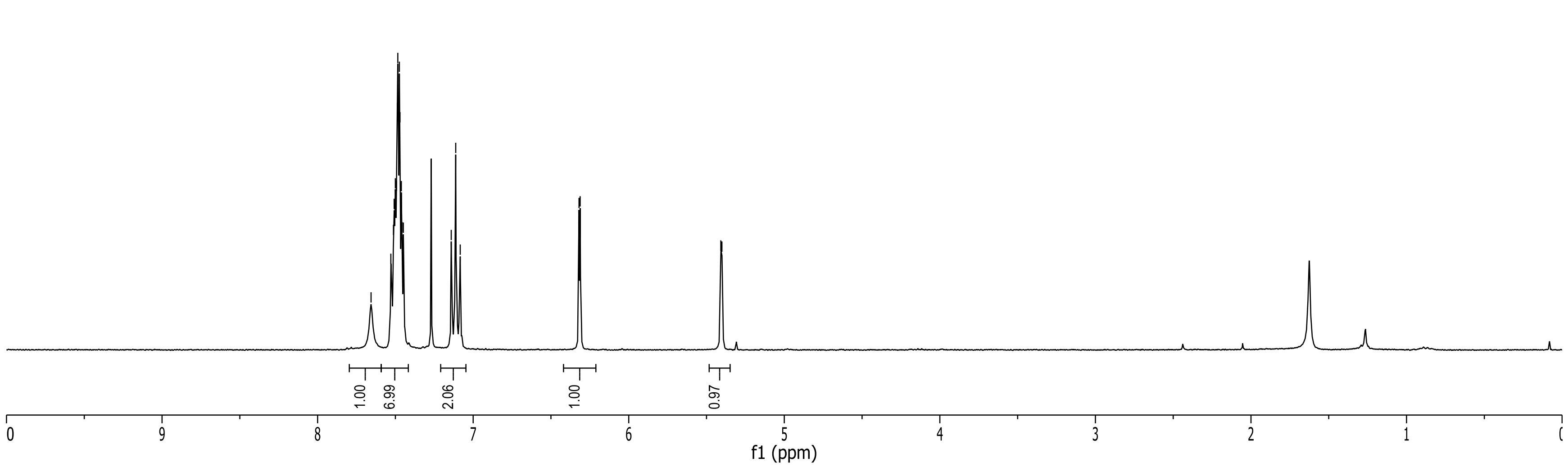

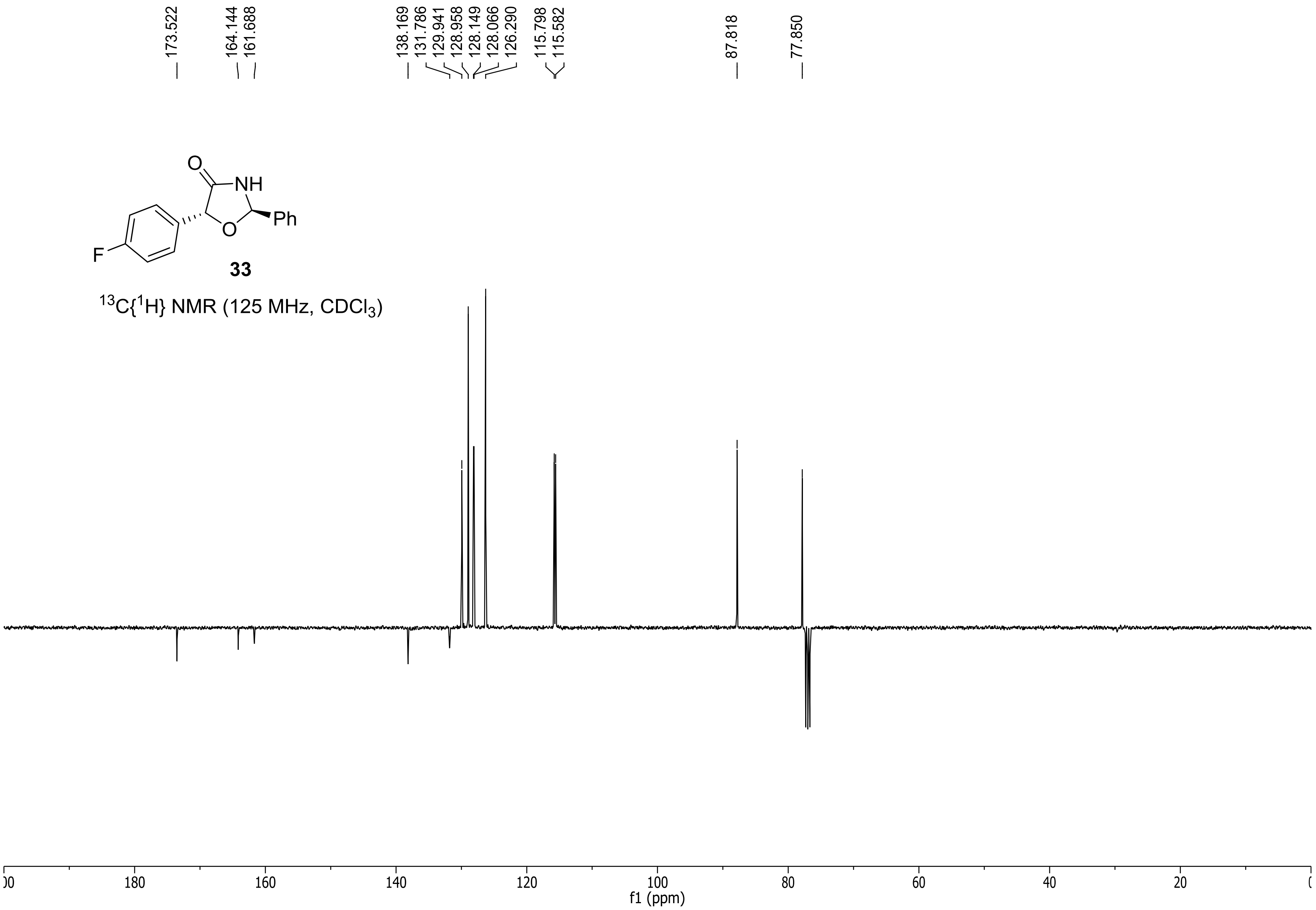
173.522
164.144
161.688
138.169
131.786
129.941
128.958
128.149
128.066
126.290
115.798
115.582
87.818
77.850
O
NH
Ph
O
F
33
$^{13}C\{^{1}H\}$ NMR (125 MHz, $CDCl_3$)
180
160
140
120
100
80
60
40
20
f1 (ppm)

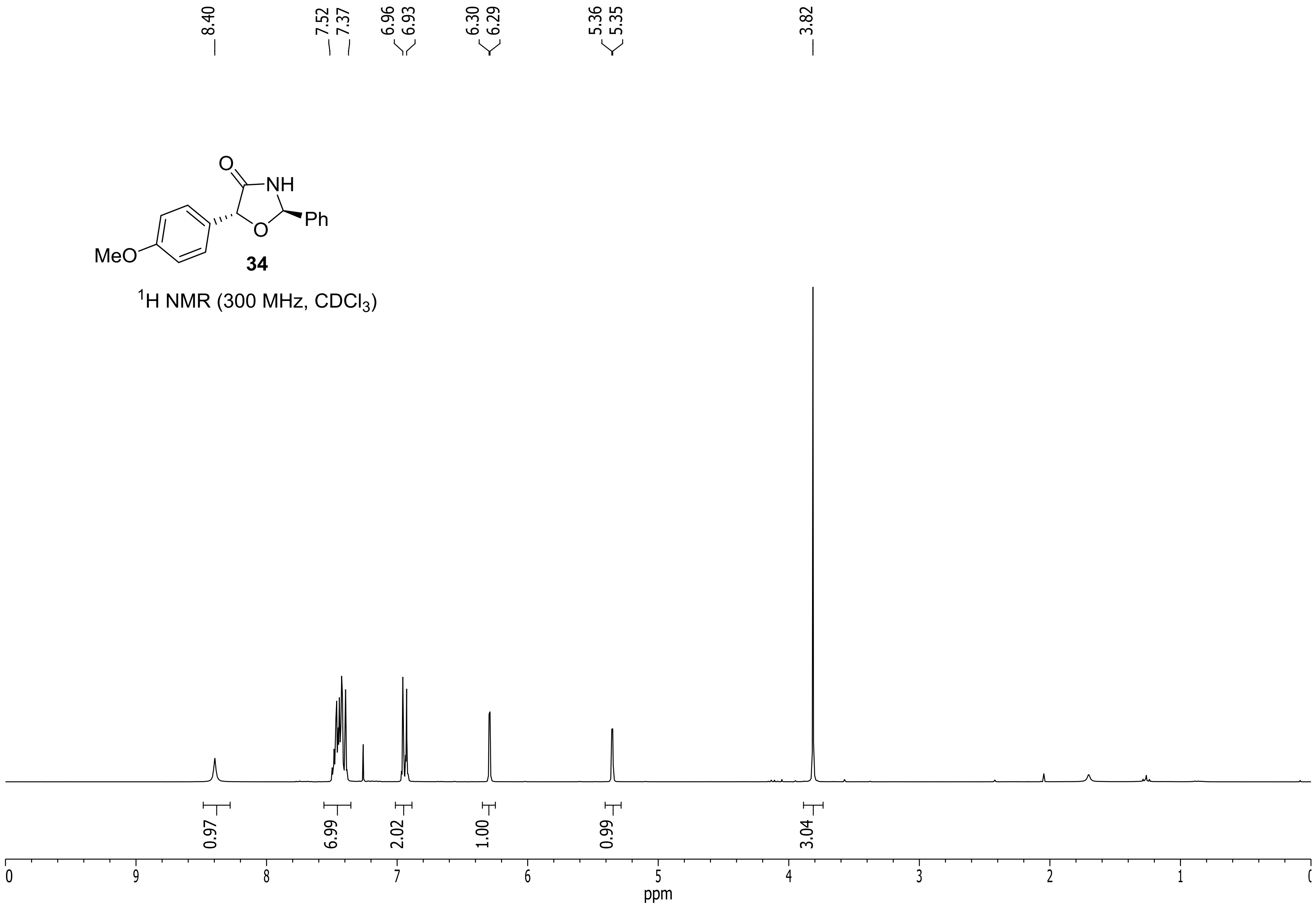
8.40
7.52
7.37
6.96
6.93
6.30
6.29
5.36
5.35
3.82
O
NH
Ph
O
MeO
34
¹H NMR (300 MHz, CDCl₃)
0.97
6.99
2.02
1.00
0.99
3.04
9
8
7
6
5
4
3
2
1
ppm

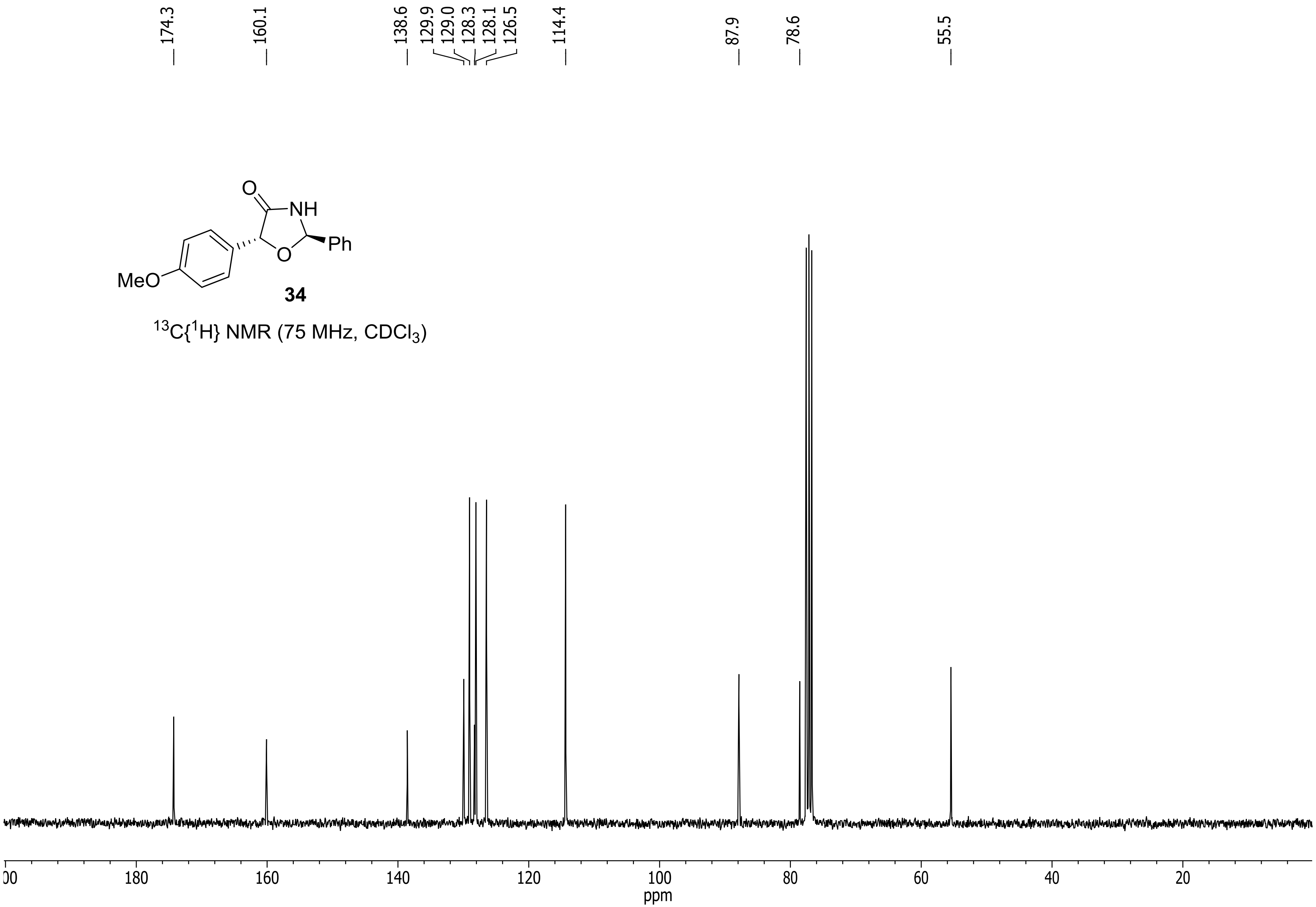

34

$^{13}C\{^{1}H\}$ NMR (75 MHz, $CDCl_3$)

HPLC Traces

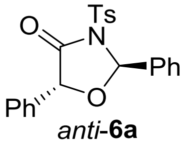

anti-**6a**

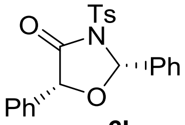

syn-**6b**

Data for the anti diastereoisomer: Chiral HPLC analysis, Chiralcel AD-H (95:5 hexane : IPA, flow rate 1.5 mL min^{-1}, 211 nm, 40 °C), t_R(2*R*,5*R*): 27.1 min, t_R(2*S*,5*S*): 53.9 min, 97% ee.

Data for the syn diastereoisomer: Chiral HPLC analysis, Chiralcel AD-H (95:5 hexane : IPA, flow rate 1.5 mL min^{-1}, 211 nm, 40 °C), t_R(2*S*,5*R*): 25.1 min, t_R(2*R*,5*S*): 30.4 min, 97% ee.

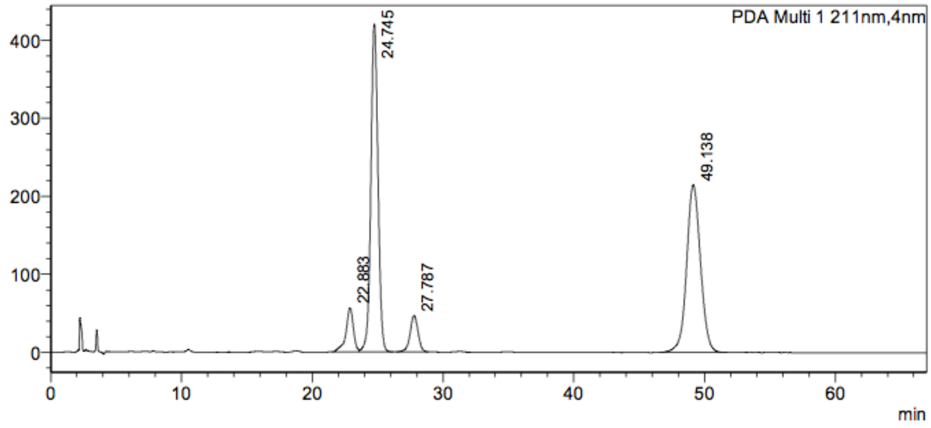

<Peak Table>

PDA Ch1 211nm

Peak#	Ret. Time	Area%
1	22.883	6.349
2	24.745	44.146
3	27.787	5.481
4	49.138	44.024
Total		100.000

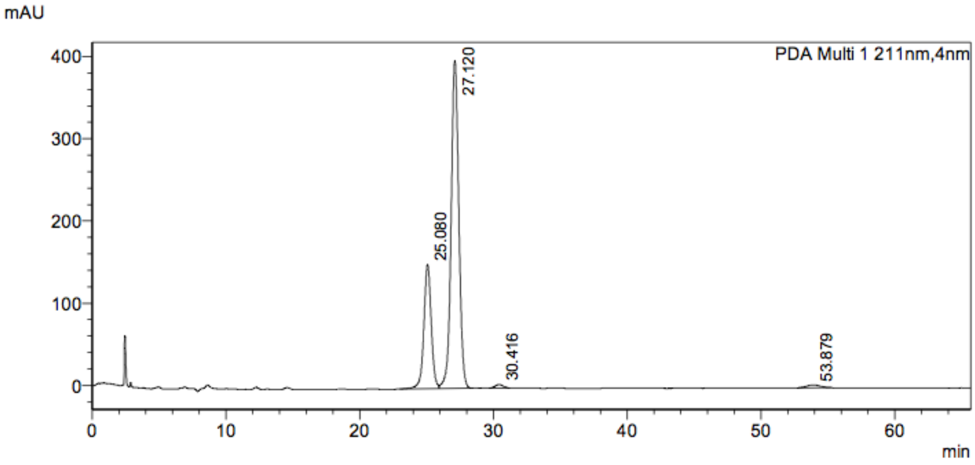

<Peak Table>

PDA Ch1 211nm

Peak#	Ret. Time	Area%
1	25.080	26.297
2	27.120	71.820
3	30.416	0.811
4	53.879	1.072
Total		100.000

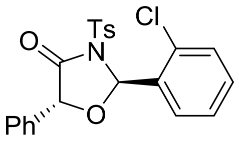

anti-**11a**

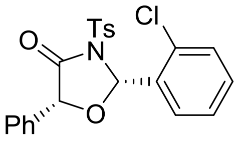

syn-**11b**

Data for the anti diastereoisomer: Chiral HPLC analysis, Chiralcel AD-H (95:5 hexane : IPA, flow rate 1.5 mL min^{-1}, 211 nm, 30 °C), t_R(2*R*,5*R*): 23.0 min, t_R(2*S*,5*S*): 53.2 min, 78% ee.

Data for the syn diastereoisomer: Chiral HPLC analysis, Chiralcel AD-H (95:5 hexane : IPA, flow rate 1.5 mL min^{-1}, 211 nm, 30 °C), t_R(2*S*,5*R*): 40.4 min, t_R(2*R*,5*S*): 46.0 min, 78% ee.

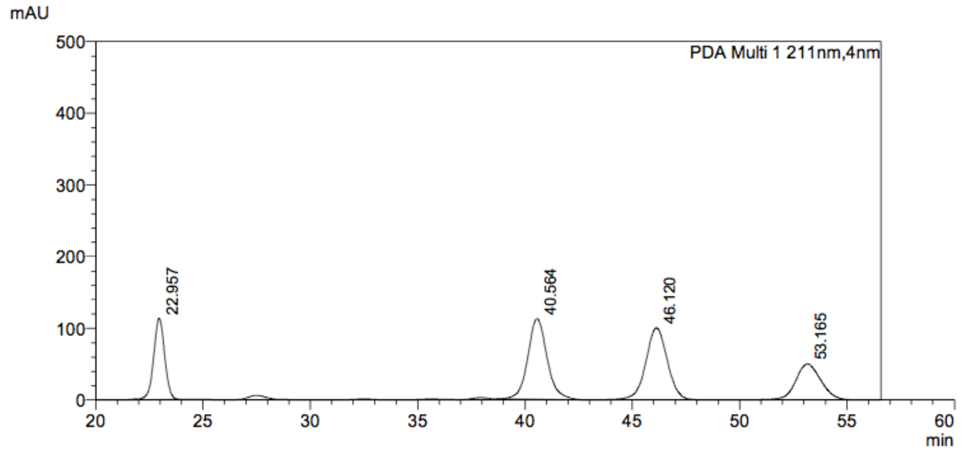

<Peak Table>

PDA Ch1 211nm

Peak#	Ret. Time	Area%
1	22.957	18.234
2	40.564	31.894
3	46.120	31.554
4	53.165	18.317
Total		100.000

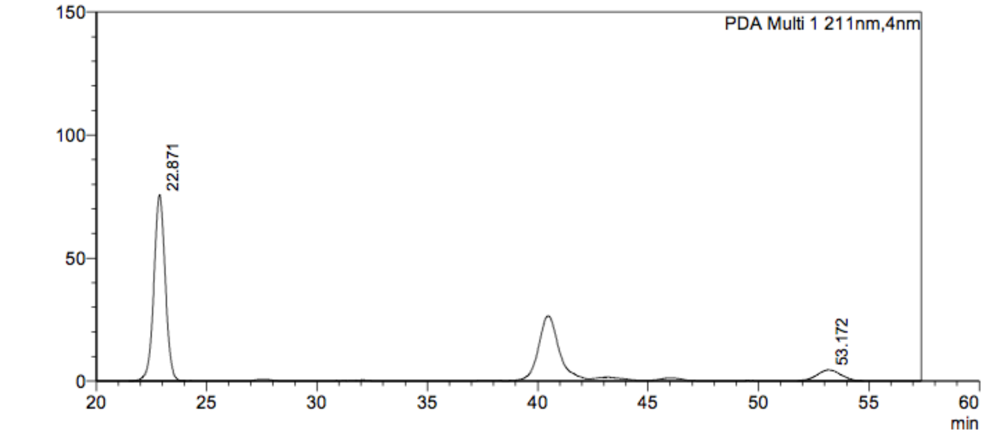

<Peak Table>

PDA Ch1 211nm

Peak#	Ret. Time	Area%
1	22.871	89.196
2	53.172	10.804
Total		100.000

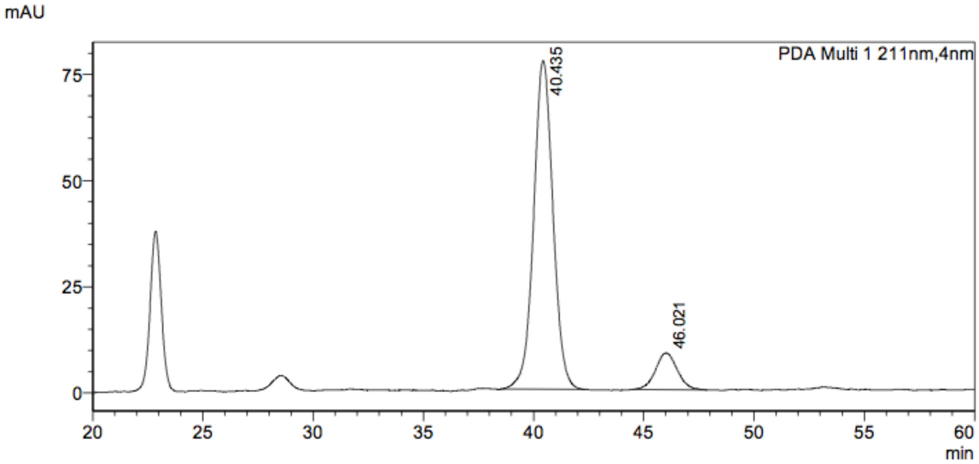

<Peak Table>

PDA Ch1 211nm

Peak#	Ret. Time	Area%
1	40.435	88.840
2	46.021	11.160
Total		100.000

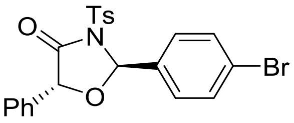

anti-**12a**

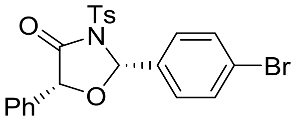

syn-**12b**

Data for the anti diastereoisomer: Chiral HPLC analysis, Chiralcel OD-H (90:10 hexane : IPA, flow rate 1.0 mL min^{-1}, 211 nm, 30 °C), t_R(2*R*,5*R*): 17.2 min, t_R(2*S*,5*S*): 20.5 min, 99% ee.

Data for the syn diastereoisomer: Chiral HPLC analysis, Chiralcel OD-H (90:10 hexane : IPA, flow rate 1.0 mL min^{-1}, 211 nm, 30 °C), t_R(2*R*,5*S*): 15.5 min, t_R(2*S*,5*R*): 24.6 min, 95% ee.

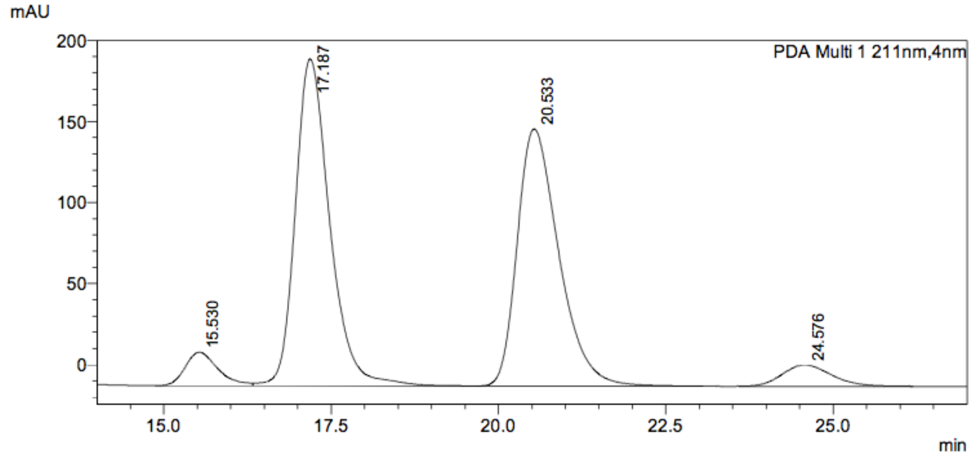

<Peak Table>

PDA Ch1 211nm

Peak#	Ret. Time	Area%
1	15.530	4.545
2	17.187	46.713
3	20.533	44.342
4	24.576	4.400
Total		100.000

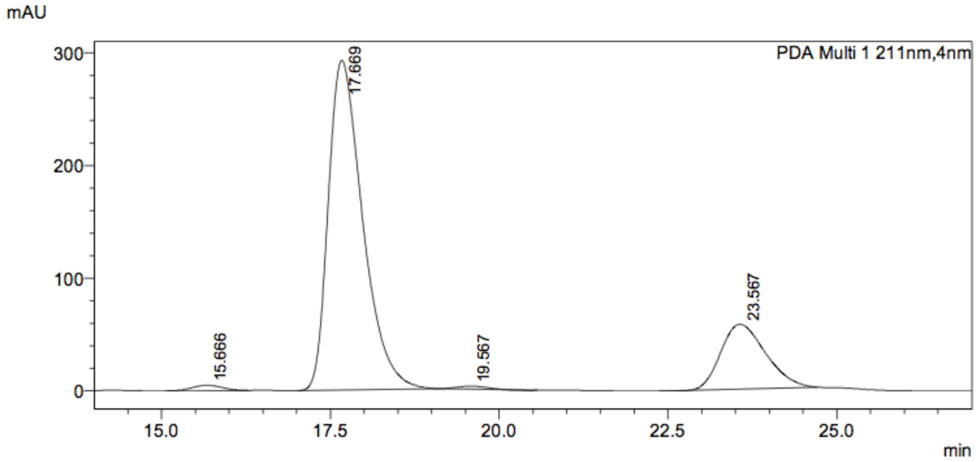

<Peak Table>

PDA Ch1 211nm

Peak#	Ret. Time	Area%
1	15.666	1.135
2	17.669	78.269
3	19.567	0.578
4	23.567	20.018
Total		100.000

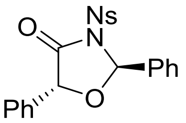

anti-**13a**

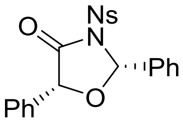

syn-**13b**

Data for the anti diastereoisomer: Chiral HPLC analysis, Chiralcel AD-H (80:20 hexane : IPA, flow rate 1.25 mL min^{-1}, 211 nm, 40 °C), t_R(2*R*,5*R*): 27.3 min, t_R(2*S*,5*S*): 29.9 min, 85% ee.

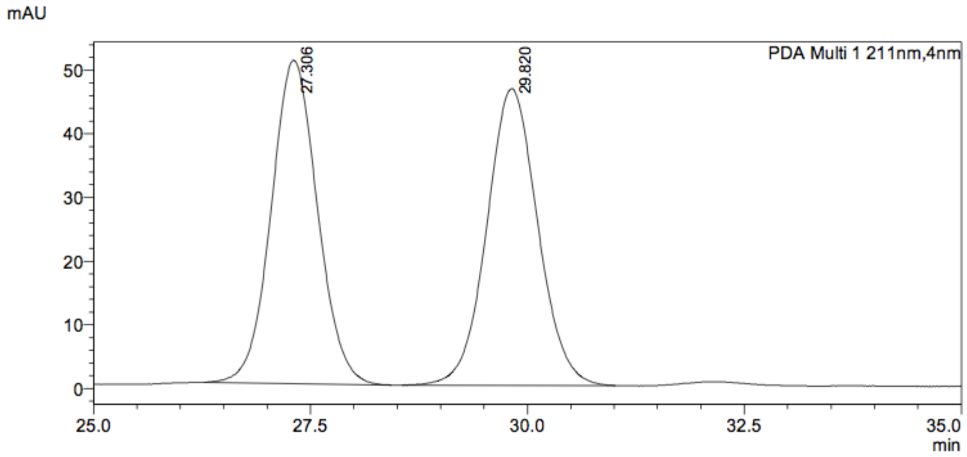

<Peak Table>

PDA Ch1 211nm

Peak#	Ret. Time	Area%
1	27.306	49.705
2	29.820	50.295
Total		100.000

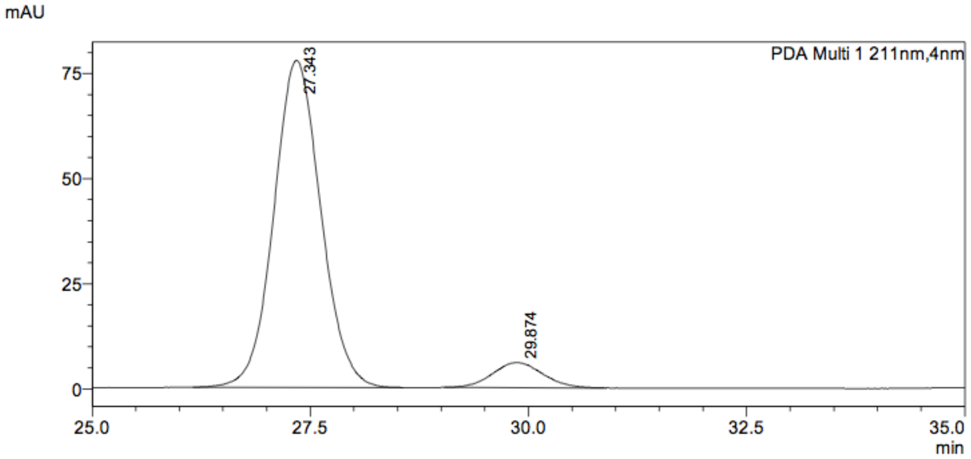

<Peak Table>

PDA Ch1 211nm

Peak#	Ret. Time	Area%
1	27.343	92.415
2	29.874	7.585
Total		100.000

Data for the syn diastereoisomer: Chiral HPLC analysis, Chiralcel AD-H (80:20 hexane : IPA, flow rate 1.5 mL min^{-1}, 211 nm, 30 °C), t_R(2*S*,5*R*): 18.3 min, t_R(2*R*,5*S*): 20.7 min, 80% ee.

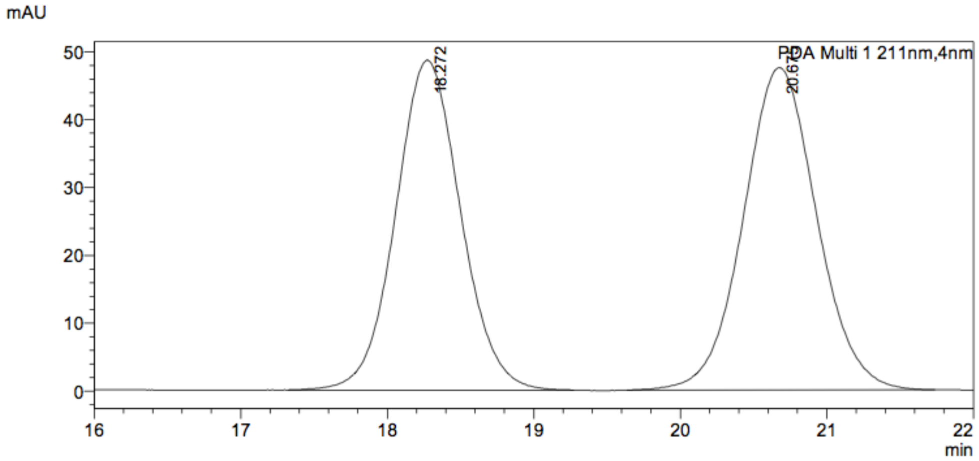

<Peak Table>

PDA Ch1 211nm

Peak#	Ret. Time	Area%
1	18.272	47.585
2	20.675	52.415
Total		100.000

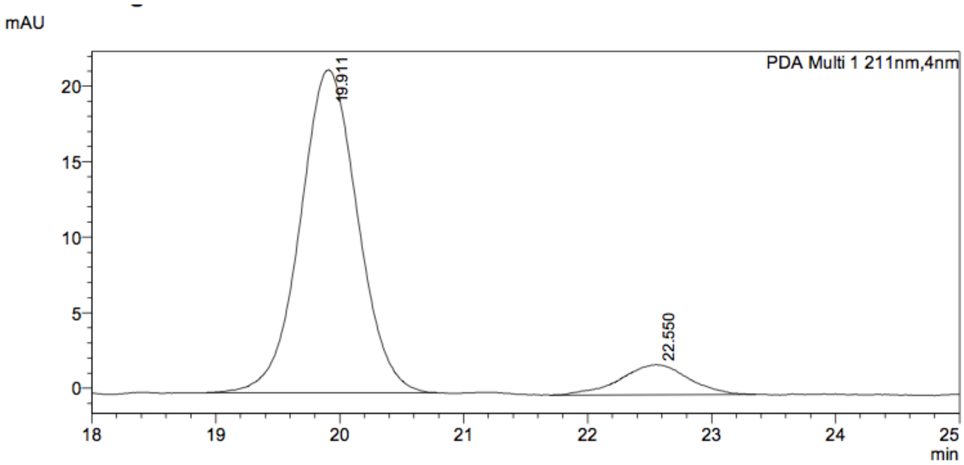

<Peak Table>

PDA Ch1 211nm

Peak#	Ret. Time	Area%
1	19.911	90.100
2	22.550	9.900
Total		100.000

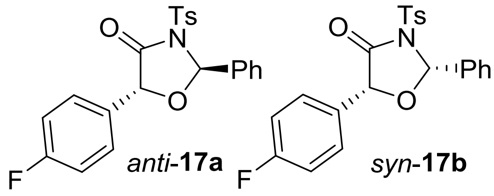

Data for the anti diastereoisomer: Chiral HPLC analysis, Chiralcel OD-H (90:10 hexane : IPA, flow rate 1.0 mL min^{-1}, 211 nm, 30 °C), t_R(2*S*,5*S*): 17.0 min, t_R(2*R*,5*R*): 18.9 min, 99% ee.

Data for the syn diastereoisomer: Chiral HPLC analysis, Chiralcel OD-H (90:10 hexane : IPA, flow rate 1.0 mL min^{-1}, 211 nm, 30 °C), t_R(2*S*,5*R*): 16.0 min, t_R(2*R*,5*S*): 27.6 min, 99% ee.

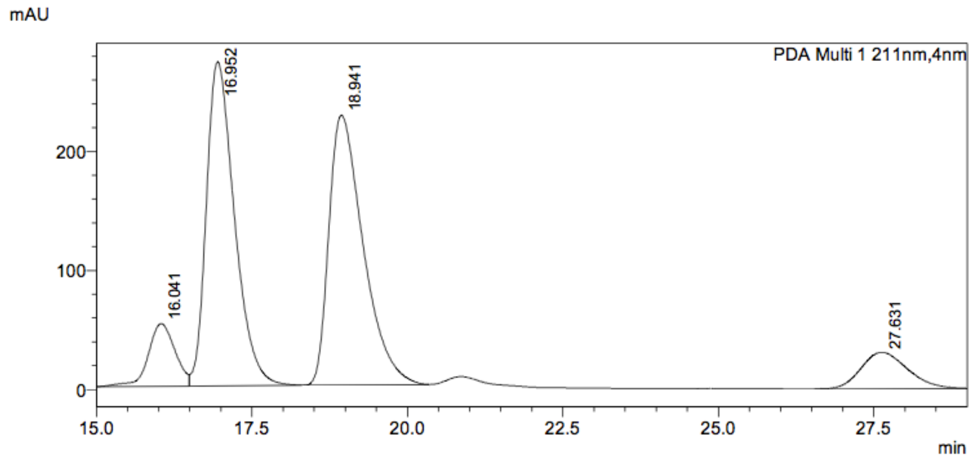

<Peak Table>

PDA Ch1 211nm

Peak#	Ret. Time	Area%
1	16.041	7.721
2	16.952	41.866
3	18.941	42.518
4	27.631	7.894
Total		100.000

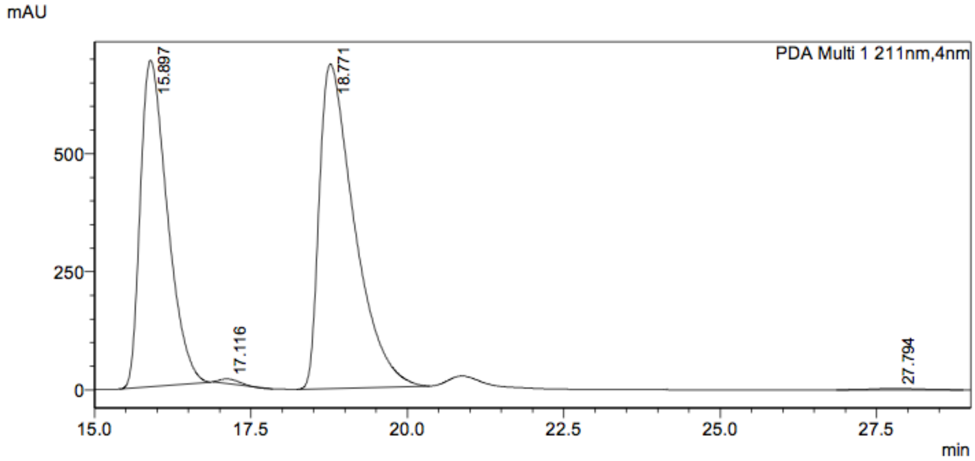

<Peak Table>

PDA Ch1 211nm

Peak#	Ret. Time	Area%
1	15.897	43.345
2	17.116	0.408
3	18.771	55.925
4	27.794	0.323
Total		100.000

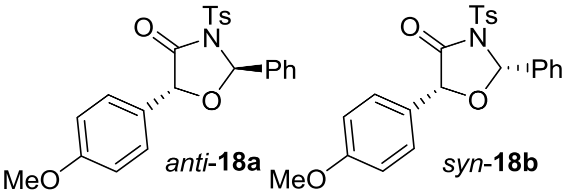

Data for the anti diastereoisomer: Chiral HPLC analysis, Chiralcel OD-H (90:10 hexane : IPA, flow rate 1.0 mL min^{-1}, 211 nm, 30 °C), t_R(2*S*,5*S*): 30.3 min, t_R(2*R*,5*R*): 33.4 min, 97% ee.

Data for the syn diastereoisomer: Chiral HPLC analysis, Chiralcel OD-H (90:10 hexane : IPA, flow rate 1.0 mL min^{-1}, 211 nm, 30 °C), t_R(2*S*,5*R*): 25.2 min, t_R(2*R*,5*S*): 27.6 min, 94% ee.

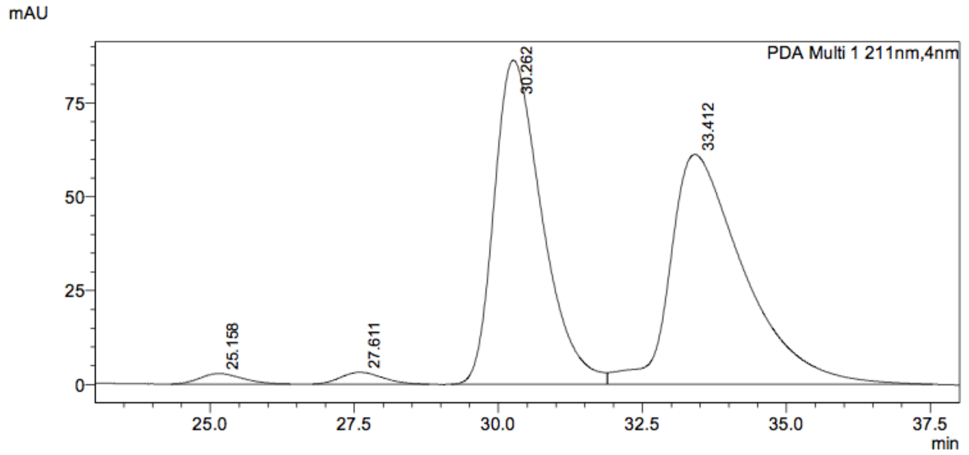

<Peak Table>

PDA Ch1 211nm

Peak#	Ret. Time	Area%
1	25.158	1.329
2	27.611	1.484
3	30.262	46.440
4	33.412	50.746
Total		100.000

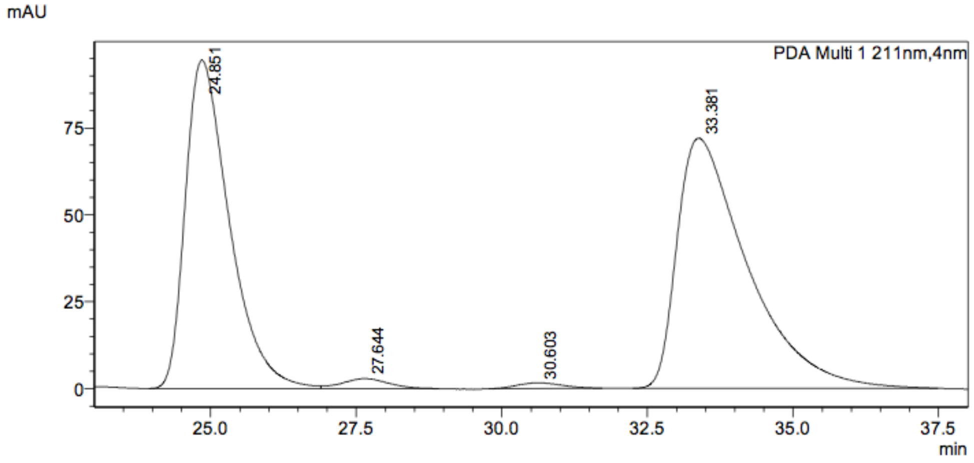

<Peak Table>

PDA Ch1 211nm

Peak#	Ret. Time	Area	Area%
1	24.851	4984985	44.131
2	27.644	159835	1.415
3	30.603	90169	0.798
4	33.381	6060800	53.655
Total		11295789	100.000

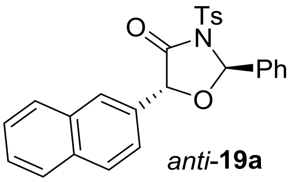

anti-**19a**

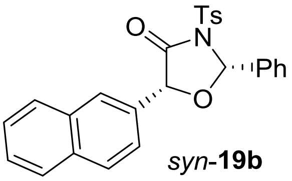

syn-**19b**

Data for the anti diastereoisomer: Chiral HPLC analysis, Chiralcel AD-H (90:10 hexane:IPA, flow rate 1.0 mL min^{-1}, 211 nm, 40 °C) t_R (2*R*,5*R*): 35.7 min, t_R (2*S*,5*S*): 84.8 min, >99% ee.

Data for the syn diastereoisomer: Chiral HPLC analysis, Chiralcel AD-H (90:10 hexane:IPA, flow rate 1.0 mL min^{-1}, 211 nm, 40 °C) t_R (2*S*,5*R*): 30.7 min, t_R (2*R*,5*S*): 47.1 min, >99% ee.

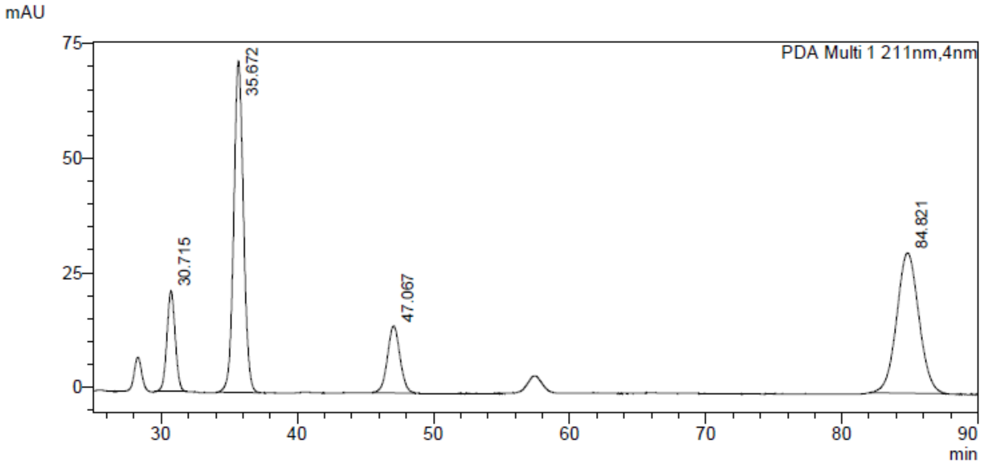

<Peak Table>

PDA Ch1 211nm

Peak#	Ret. Time	Area%
1	30.715	10.538
2	35.672	40.013
3	47.067	10.456
4	84.821	38.992
Total		100.000

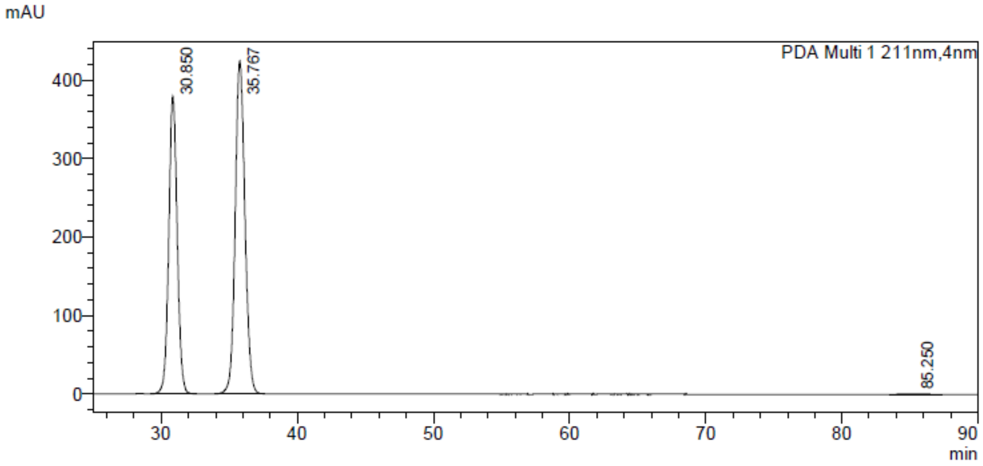

<Peak Table>

PDA Ch1 211nm

Peak#	Ret. Time	Area%
1	30.850	43.769
2	35.767	56.117
3	85.250	0.115
Total		100.000

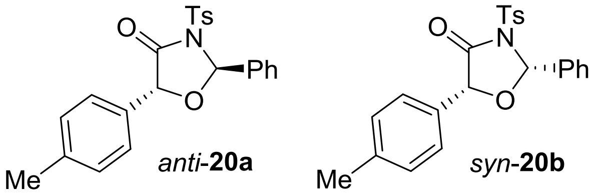

Data for the anti diastereoisomer: Chiral HPLC analysis, Chiralcel OD-H (90:10 hexane : IPA, flow rate 1.0 mL min^{-1}, 211 nm, 30 °C), t_R(2*R*,5*R*): 17.2 min, t_R(2*S*,5*S*): 22.5 min, 97% ee.

Data for the syn diastereoisomer: Chiral HPLC analysis, Chiralcel OD-H (90:10 hexane : IPA, flow rate 1.0 mL min^{-1}, 211 nm, 30 °C), t_R(2*S*,5*R*): 15.3 min, t_R(2*R*,5*S*): 26.7 min, 99% ee.

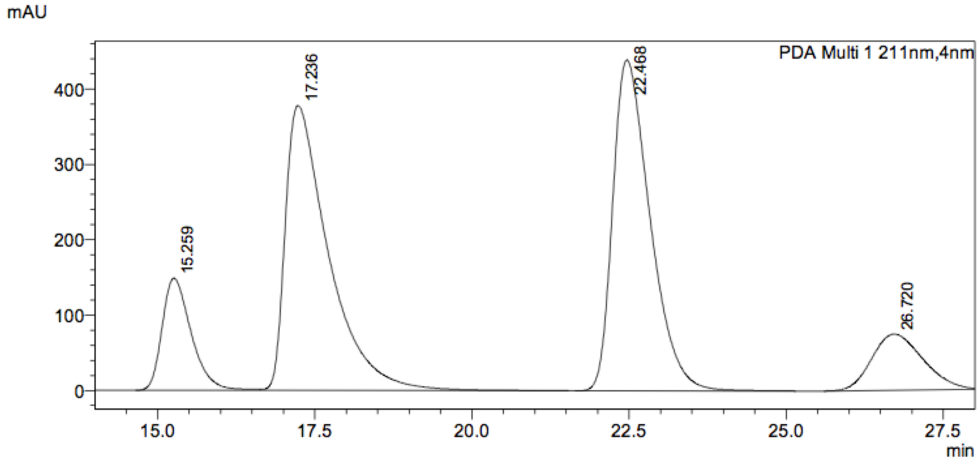

<Peak Table>

PDA Ch1 211nm

Peak#	Ret. Time	Area%
1	15.259	10.578
2	17.236	40.057
3	22.468	40.044
4	26.720	9.321
Total		100.000

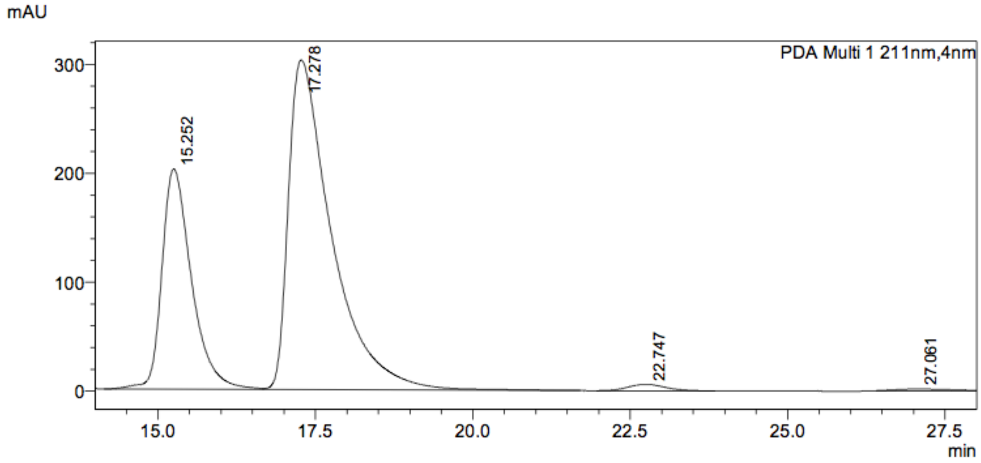

<Peak Table>

PDA Ch1 211nm

Peak#	Ret. Time	Area%
1	15.252	31.003
2	17.278	67.436
3	22.747	1.146
4	27.061	0.415
Total		100.000

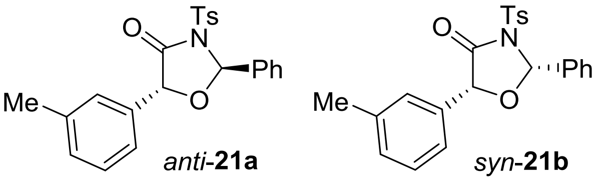

anti-**21a** *syn*-**21b**

Data for the anti diastereoisomer: Chiralpak AD-H (90:10 hexane : IPA, flow rate 1.0 mL min^{-1}, 211 nm, 40 °C), t_R(2*R*,5*R*): 20.9 min, t_R(2*S*,5*S*): 42.0 min, 92% ee.

Data for the syn diastereoisomer: Chiralpak AD-H (90:10 hexane : IPA, flow rate 1.0 mL min^{-1}, 211 nm, 40 °C), t_R(2*S*,5*R*): 19.1 min, t_R(2*R*,5*S*): 26.3 min, 94% ee.

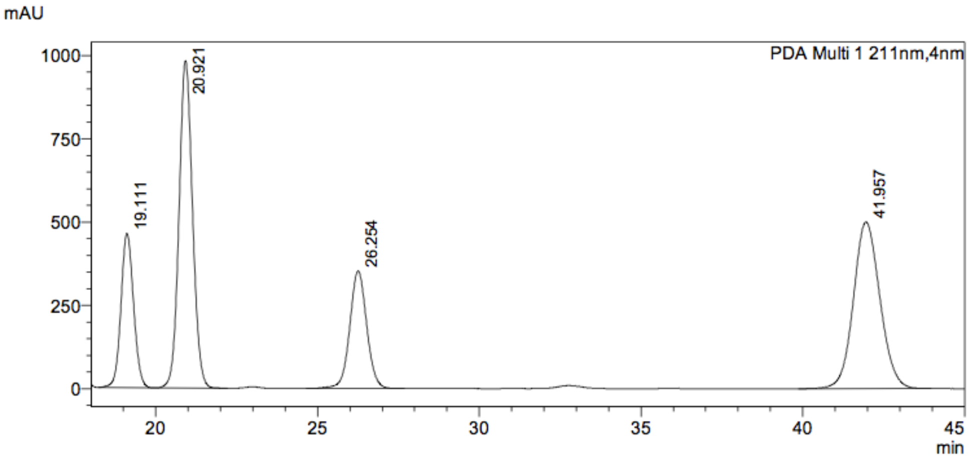

<Peak Table>

PDA Ch1 211nm

Peak#	Ret. Time	Area%
1	19.111	15.262
2	20.921	34.662
3	26.254	15.579
4	41.957	34.497
Total		100.000

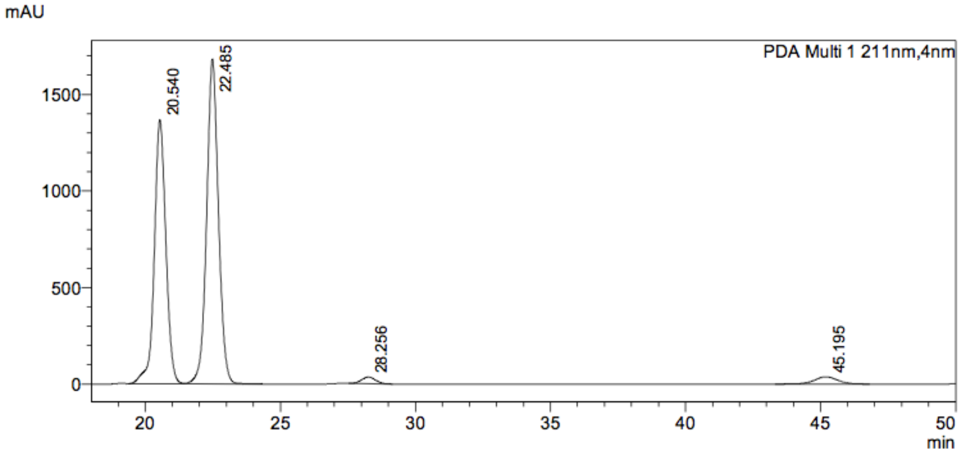

<Peak Table>

PDA Ch1 211nm

Peak#	Ret. Time	Area%
1	20.540	42.565
2	22.485	53.756
3	28.256	1.304
4	45.195	2.375
Total		100.000

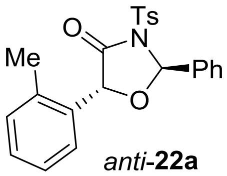
anti-**22a**

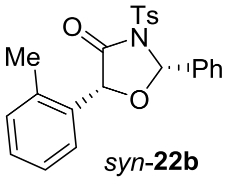
syn-**22b**

Data for the anti diastereoisomer: Chiral HPLC analysis, Chiralpak AD-H (90:10 hexane : IPA, flow rate 1.0 mL min^{-1}, 211 nm, 40 °C), t_R(2*S*,5*S*): 22.9 min, t_R(2*R*,5*R*): 26.5 min, >99% ee.

Data for the syn diastereoisomer: Chiral HPLC analysis, Chiralpak AD-H (90:10 hexane : IPA, flow rate 1.0 mL min^{-1}, 211 nm, 40 °C), t_R(2*R*,5*S*): 16.8 min, t_R(2*S*,5*R*): 18.8 min, >99% ee.

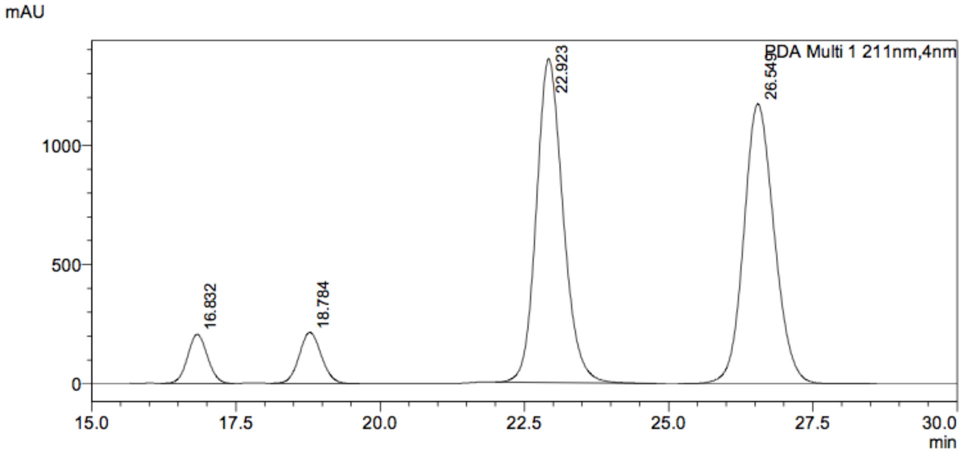

<Peak Table>

PDA Ch1 211nm

Peak#	Ret. Time	Area%
1	16.832	5.059
2	18.784	5.869
3	22.923	44.933
4	26.549	44.139
Total		100.000

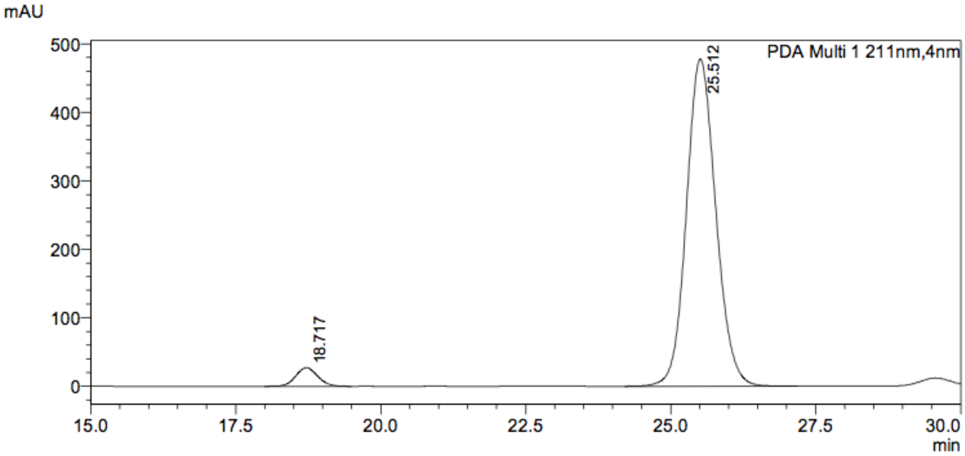

<Peak Table>

PDA Ch1 211nm

Peak#	Ret. Time	Area%
1	18.717	4.084
2	25.512	95.916
Total		100.000

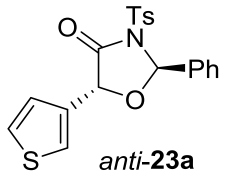

anti-**23a**

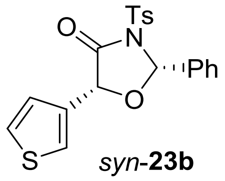

syn-**23b**

Data for the anti diastereoisomer: Chiral HPLC analysis, Chiralpak IA (80:20 hexane : IPA, flow rate 1.5 mL min^{-1}, 211 nm, 30 °C), t_R(2*R*,5*R*): 8.7 min, t_R(2*S*,5*S*): 13.2 min, 87% ee.

Data for the syn diastereoisomer: Chiral HPLC analysis, Chiralpak IA (80:20 hexane : IPA, flow rate 1.5 mL min^{-1}, 211 nm, 30 °C), t_R(2*S*,5*R*): 9.4 min, t_R(2*R*,5*S*): 9.8 min, 81% ee.

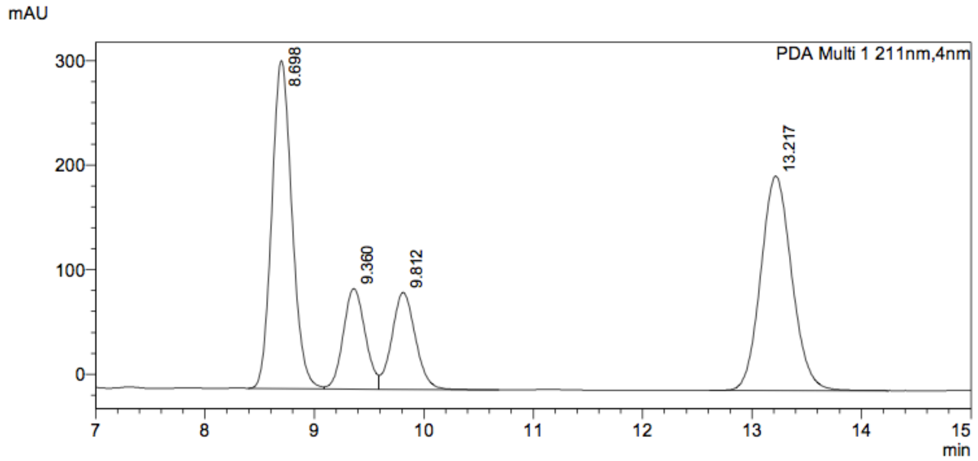

<Peak Table>

PDA Ch1 211nm

Peak#	Ret. Time	Area%
1	8.698	37.263
2	9.360	12.432
3	9.812	12.838
4	13.217	37.467
Total		100.000

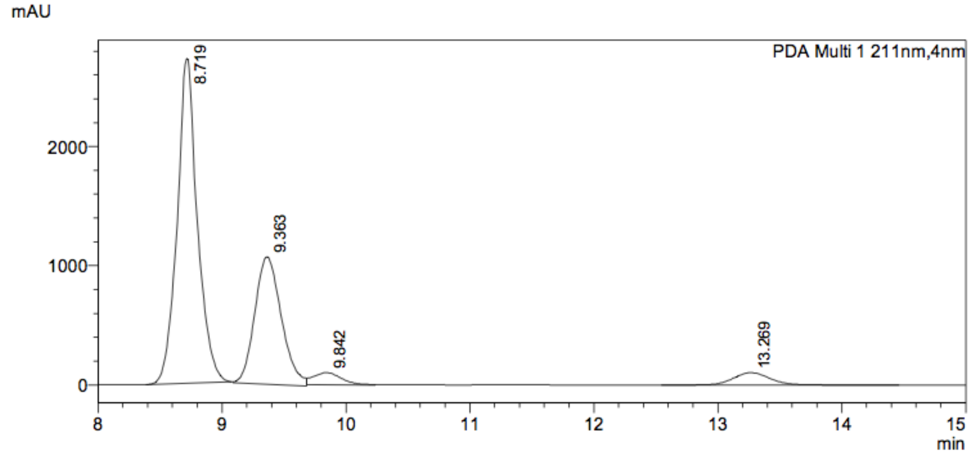

<Peak Table>

PDA Ch1 211nm

Peak#	Ret. Time	Area%
1	8.719	61.117
2	9.363	31.425
3	9.842	3.276
4	13.269	4.182
Total		100.000

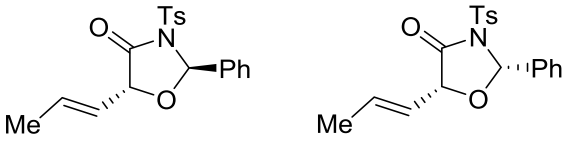

anti-**24a** *syn*-**24b**

Data for the anti diastereoisomer: Chiral HPLC analysis, Chiralcel OD-H (95:5 hexane : IPA, flow rate 1.0 mL min^{-1}, 211 nm, 30 °C), t_R(2*S*,5*S*): 19.0 min, t_R(2*R*,5*R*): 22.3 min, >99% ee.

Data for the syn diastereoisomer: Chiral HPLC analysis, Chiralcel OD-H (95:5 hexane : IPA, flow rate 1.0 mL min^{-1}, 211 nm, 30 °C), t_R(2*S*,5*R*): 21.0 min, t_R(2*R*,5*S*): 29.9 min, >99% ee.

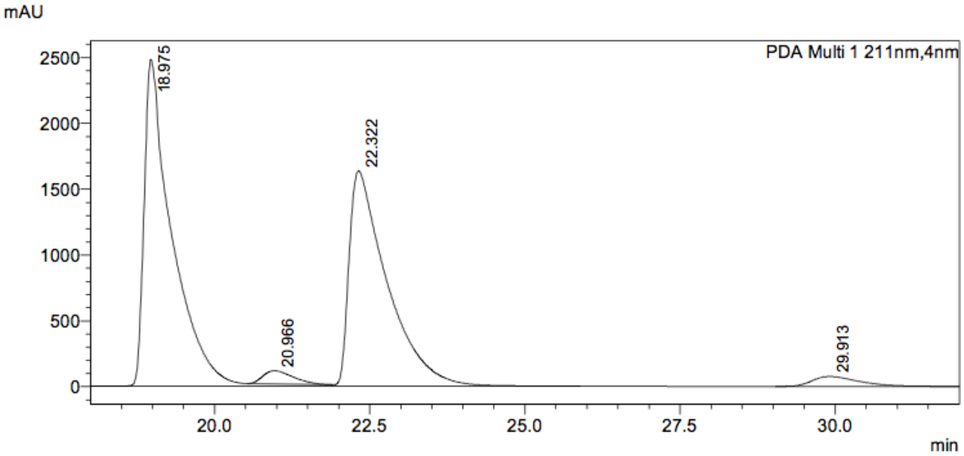

<Peak Table>

PDA Ch1 211nm

Peak#	Ret. Time	Area%
1	18.975	50.340
2	20.966	2.217
3	22.322	44.882
4	29.913	2.561
Total		100.000

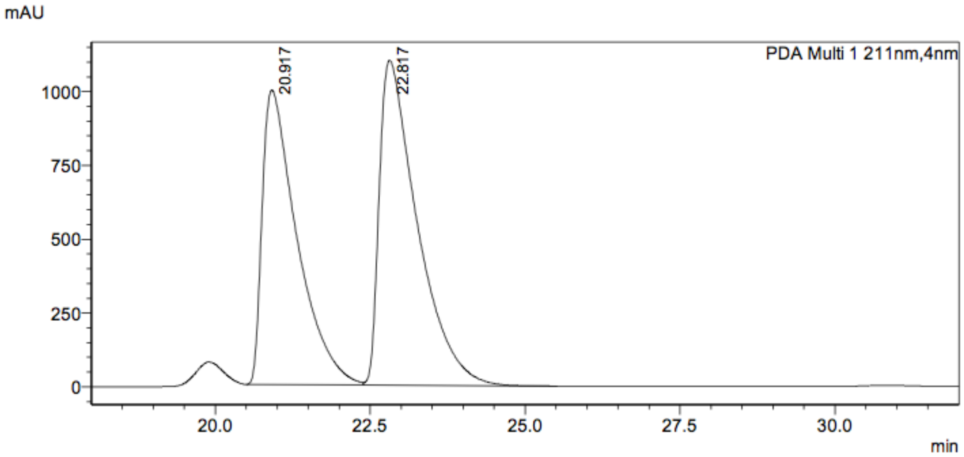

<Peak Table>

PDA Ch1 211nm

Peak#	Ret. Time	Area%
1	20.917	45.357
2	22.817	54.643
Total		100.000

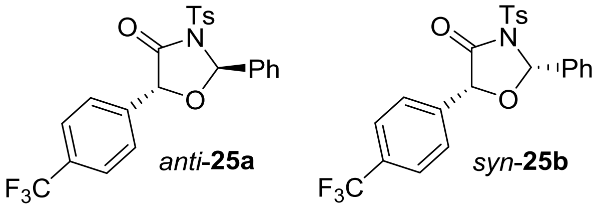

Data for the anti diastereoisomer: Chiral HPLC analysis, Chiralcel OD-H (90:10 hexane : IPA, flow rate 1.0 mL min^{-1}, 211 nm, 30 °C), t_R(2*S*,5*A*): 19.8 min, t_R(2*R*,5*R*): 22.6 min, 43% ee.

Data for the syn diastereoisomer: Chiral HPLC analysis, Chiralcel OD-H (90:10 hexane : IPA, flow rate 1.0 mL min^{-1}, 211 nm, 30 °C), t_R(2*S*,5*R*): 15.9 min, t_R(2*R*,5*S*): 35.9 min, 36% ee.

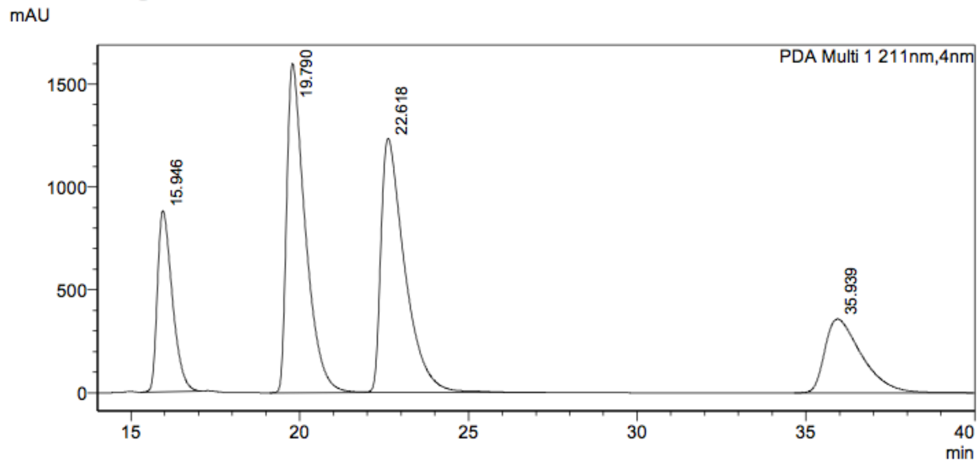

<Peak Table>

PDA Ch1 211nm

Peak#	Ret. Time	Area%
1	15.946	15.311
2	19.790	34.826
3	22.618	34.468
4	35.939	15.395
Total		100.000

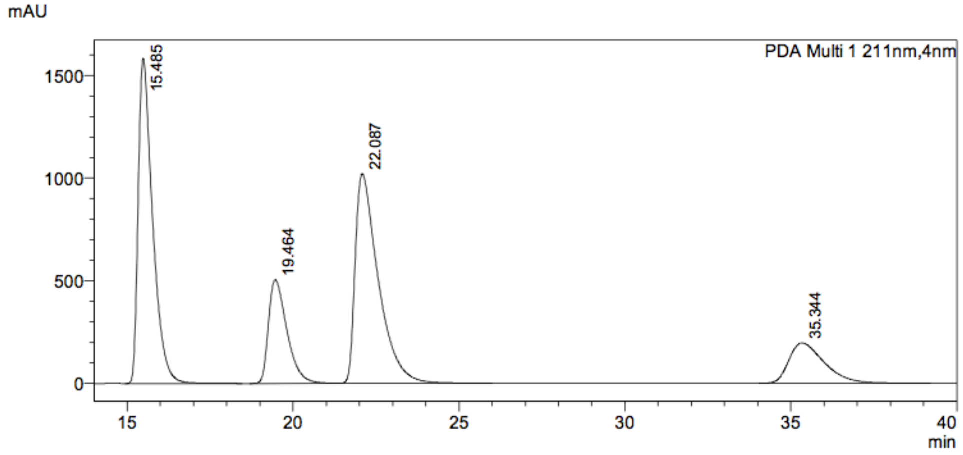

<Peak Table>

PDA Ch1 211nm

Peak#	Ret. Time	Area%
1	15.485	36.410
2	19.464	14.870
3	22.087	37.626
4	35.344	11.093
Total		100.000

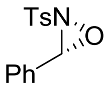

(*R*,*R*)-**5**

Chiral HPLC analysis, Chiralcel OD-H (95:5 hexane : IPA, flow rate 1.0 mL min^{-1}, 211 nm, 30 °C), t_R(*R*,*R*): 9.6 min, t_R(*S*,*S*): 13.7 min, 94% ee.

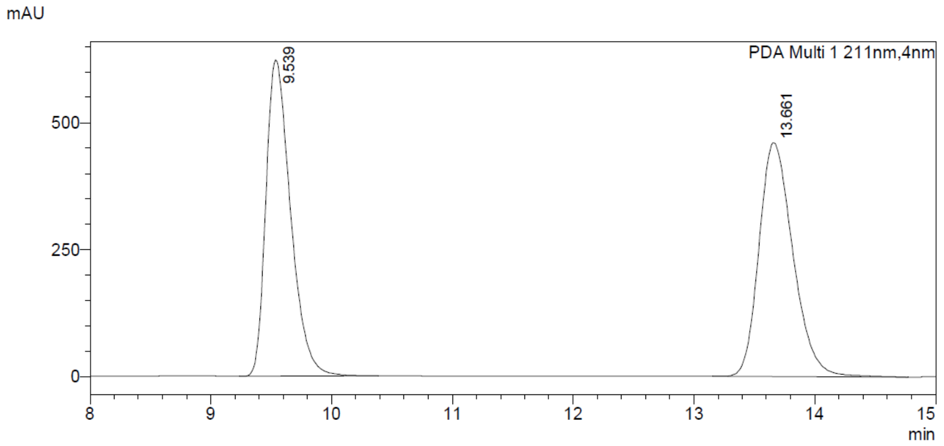

<Peak Table>

PDA Ch1 211nm

Peak#	Ret. Time	Area%
1	9.539	49.601
2	13.661	50.399
Total		100.000

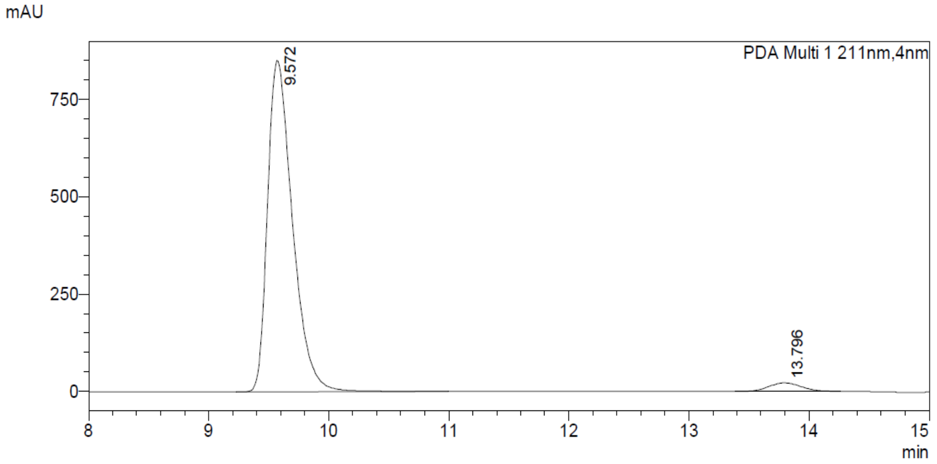

<Peak Table>

PDA Ch1 211nm

Peak#	Ret. Time	Area%
1	9.572	96.680
2	13.796	3.320
Total		100.000

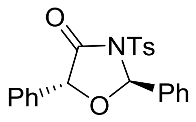

6a

Chiral HPLC analysis, AD-H (95:5 hexane : IPA, flow rate 1.5 mL min^{-1}, 211 nm, 40 °C), t_R (2*R*,5*R*): 27.1 min, t_R (2*S*,5*S*): 49.3 min, >99% ee.

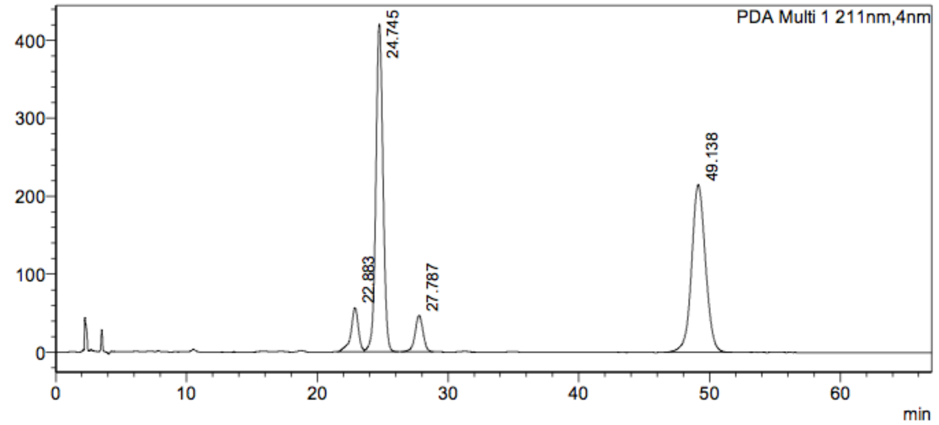

<Peak Table>

PDA Ch1 211nm

Peak#	Ret. Time	Area%
1	22.883	6.349
2	24.745	44.146
3	27.787	5.481
4	49.138	44.024
Total		100.000

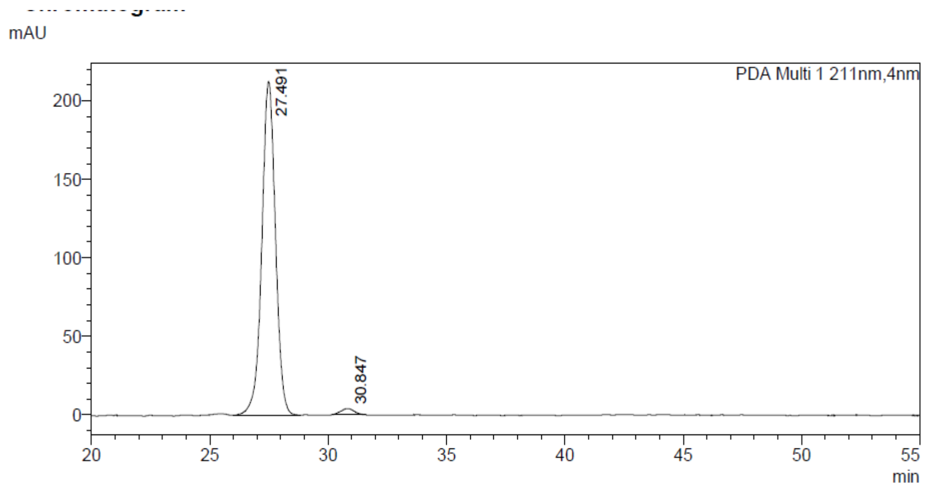

<Peak Table>

PDA Ch1 211nm

Peak#	Ret. Time	Area%
1	27.491	98.176
2	30.847	1.824
Total		100.000

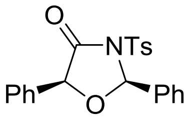

6b

Chiral HPLC analysis, AD-H (95:5 hexane : IPA, flow rate 1.5 mL min^{-1}, 211 nm, 40 °C), t_R (2*S*,5*R*): 25.1 min, t_R (2R,5*S*): 30.4 min, >98% ee.

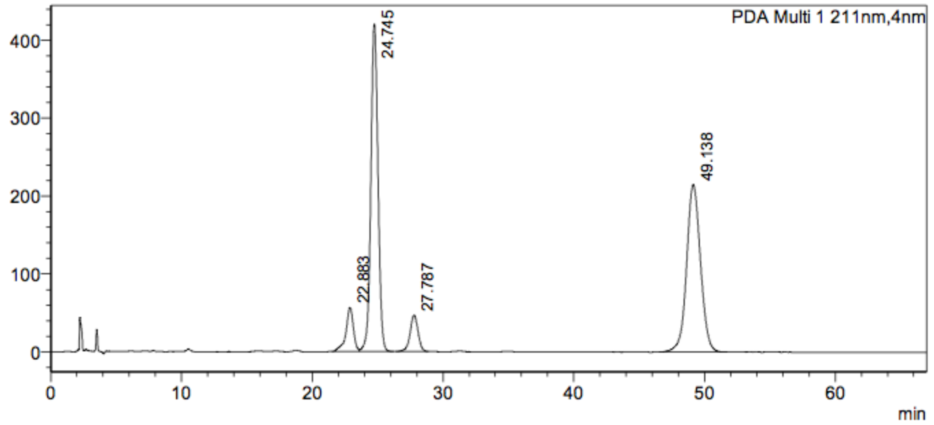

<Peak Table>

PDA Ch1 211nm

Peak#	Ret. Time	Area%
1	22.883	6.349
2	24.745	44.146
3	27.787	5.481
4	49.138	44.024
Total		100.000

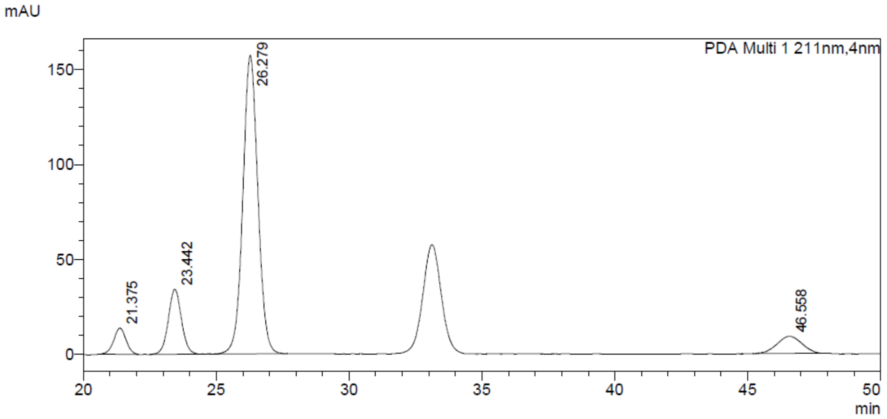

<Peak Table>

PDA Ch1 211nm

Peak#	Ret. Time	Area%
1	21.375	5.345
2	23.442	14.019
3	26.279	73.618
4	46.558	7.017
Total		100.000

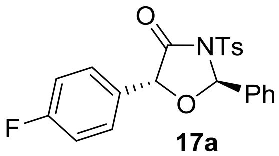

17a

Chiral HPLC analysis, OD-H (90:10 hexane : IPA, flow rate 1.0 mL min^{-1}, 211 nm, 30 °C), t_R (2*R*,5*R*): 13.5 min, t_R (2*S*,5*S*): 15.4 min, >99% ee.

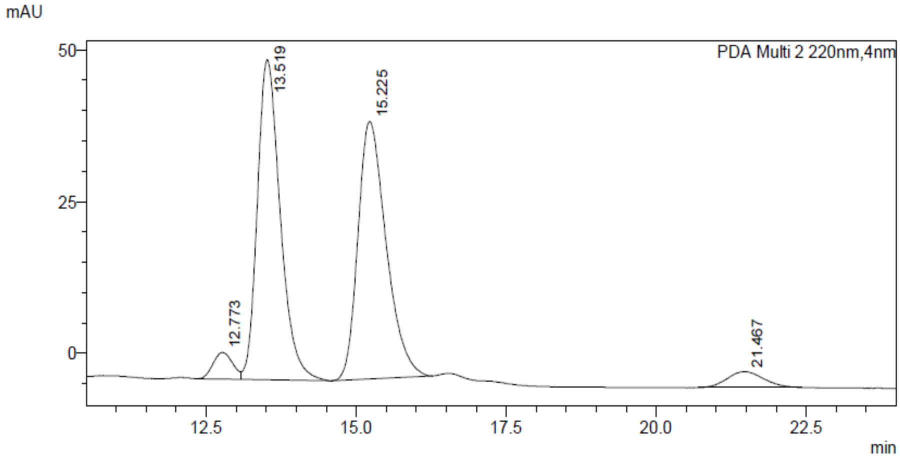

<Peak Table>

PDA Ch2 220nm

Peak#	Ret. Time	Area%
1	12.773	3.332
2	13.519	46.718
3	15.225	46.263
4	21.467	3.687
Total		100.000

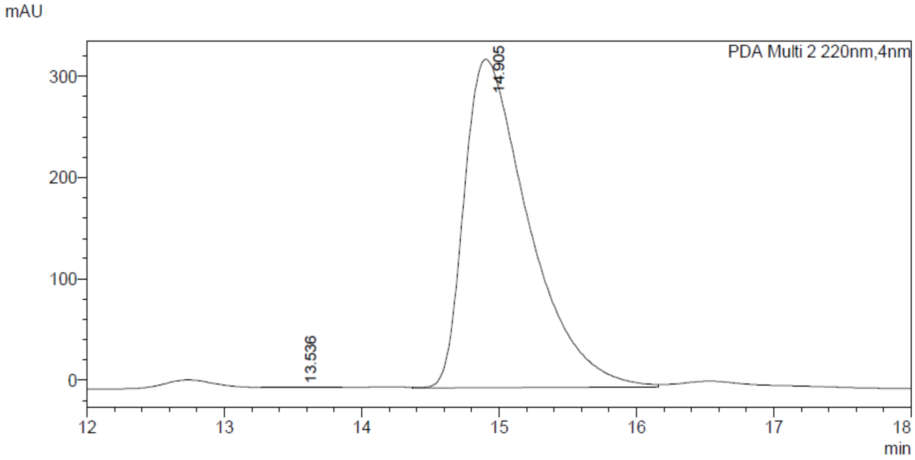

<Peak Table>

PDA Ch2 220nm

Peak#	Ret. Time	Area%
1	13.536	0.069
2	14.905	99.931
Total		100.000

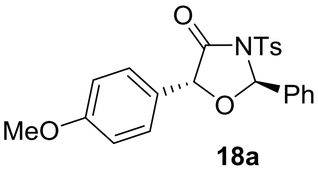

18a

Chiral HPLC analysis, OD-H (90:10 hexane : IPA, flow rate 1.0 mL min^{-1}, 211 nm, 30 °C), t_R (2*R*,5*R*): 24.2 min, t_R (2*S*,5*S*): 26.0 min, >99% ee.

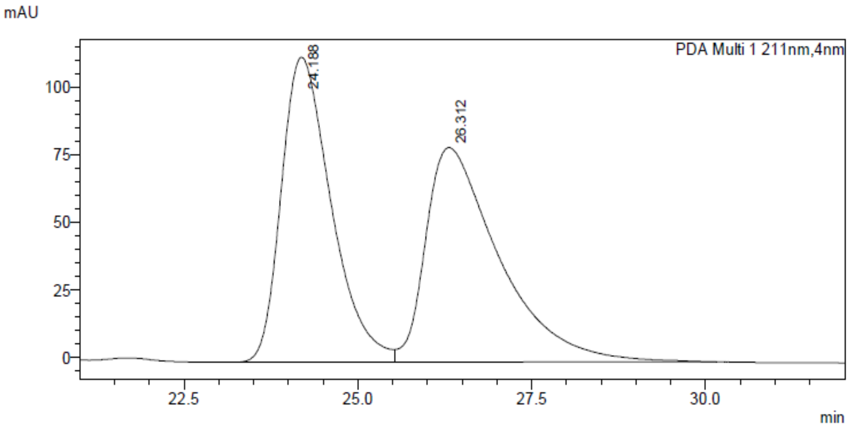

<Peak Table>

PDA Ch1 211nm

Peak#	Ret. Time	Area%
1	24.188	49.099
2	26.312	50.901
Total		100.000

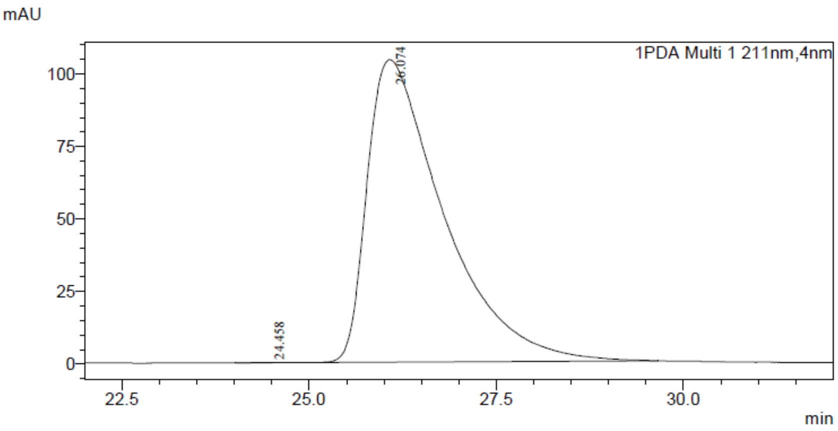

<Quantitative Results>

Peak Table

PDA Ch1 211nm

Peak#	Ret. Time	Area%
1	24.458	0.028
2	26.074	99.972
Total		100.000

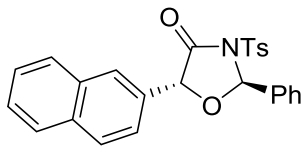

anti-**19a**

Chiral HPLC analysis, Chiralcel AD-H (90:10 hexane:IPA, flow rate 1.0 mL min^{-1}, 211 nm, 40 °C) t_R (2*R*,5*R*): 33.2 min, t_R (2*S*,5*S*): 84.8 min, >99% ee.

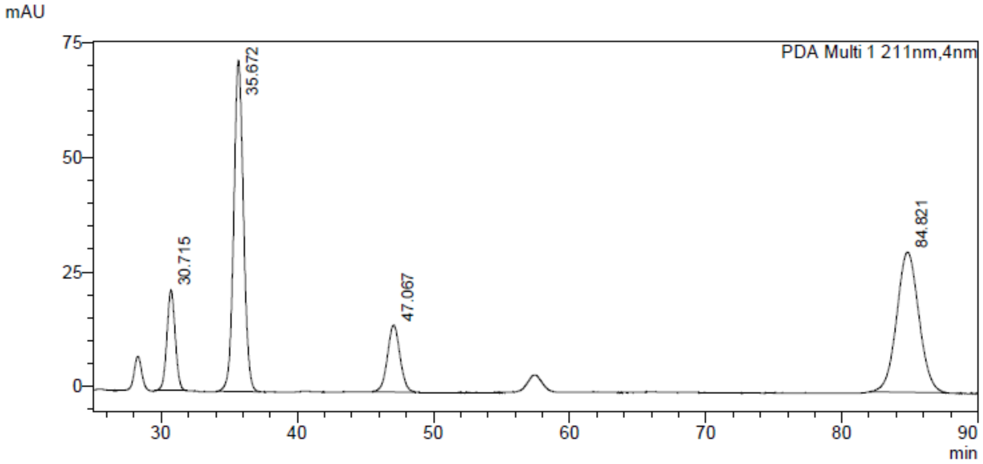

<Peak Table>

PDA Ch1 211nm

Peak#	Ret. Time	Area%
1	30.715	10.538
2	35.672	40.013
3	47.067	10.456
4	84.821	38.992
Total		100.000

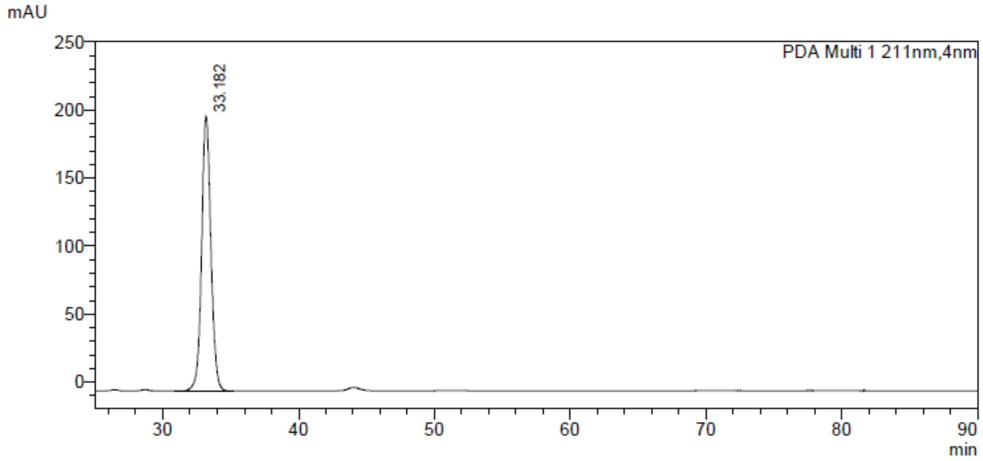

<Peak Table>

PDA Ch1 211nm

Peak#	Ret. Time	Area%
1	33.182	100.000
Total		100.000

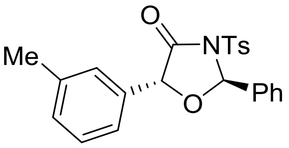

anti-**21a**

Chiral HPLC analysis, Chiralpak AD-H (90:10 hexane:IPA, flow rate 1.0 mL min^{-1}, 211 nm, 40 °C) t_R (2*R*,5*R*): 18.9 min, t_R (2*S*,5*S*): 42.0 min, >99% ee.

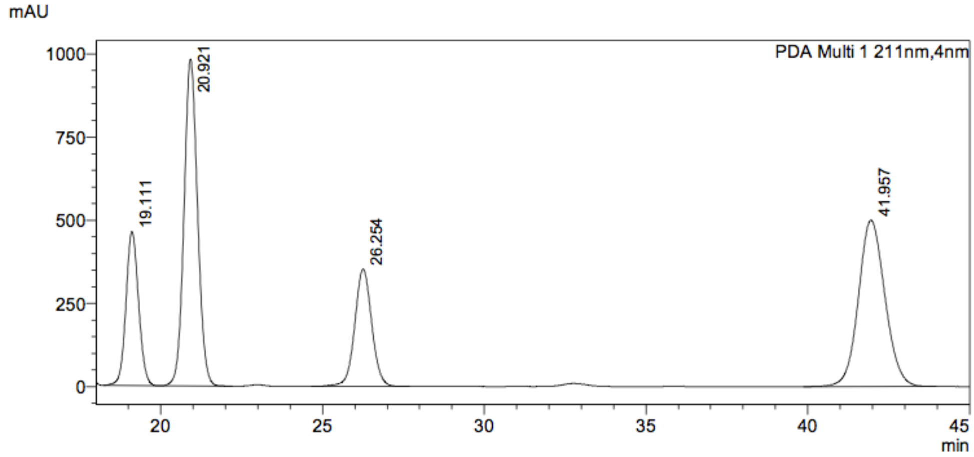

<Peak Table>

PDA Ch1 211nm

Peak#	Ret. Time	Area%
1	19.111	15.262
2	20.921	34.662
3	26.254	15.579
4	41.957	34.497
Total		100.000

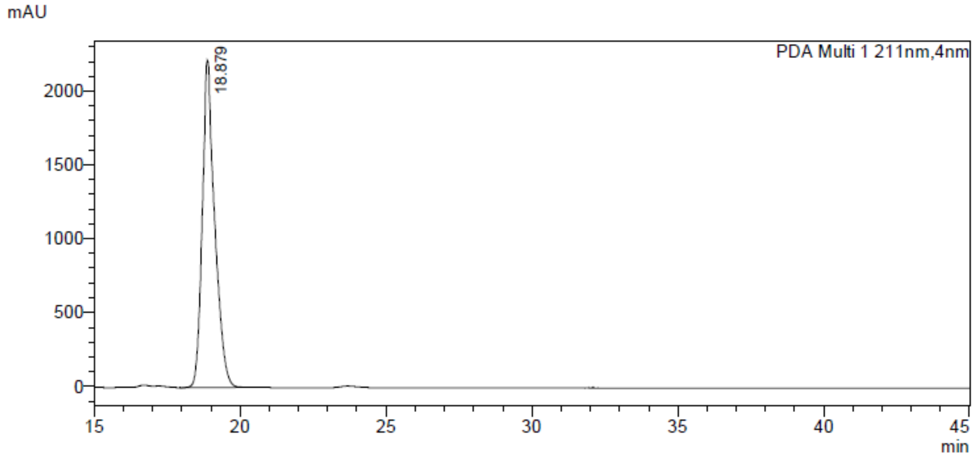

<Peak Table>

PDA Ch1 211nm

Peak#	Ret. Time	Area%
1	18.879	100.000
Total		100.000

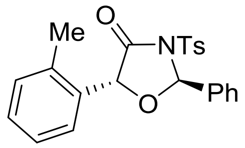

anti-**22a**

Chiral HPLC analysis, Chiralpak AD-H (90:10 hexane:IPA, flow rate 1.0 mL min^{-1}, 211 nm, 40 °C) t_R (2*R*,5*R*): 21.1 min, t_R (2*S*,5*S*): 24.4 min, >99% ee.

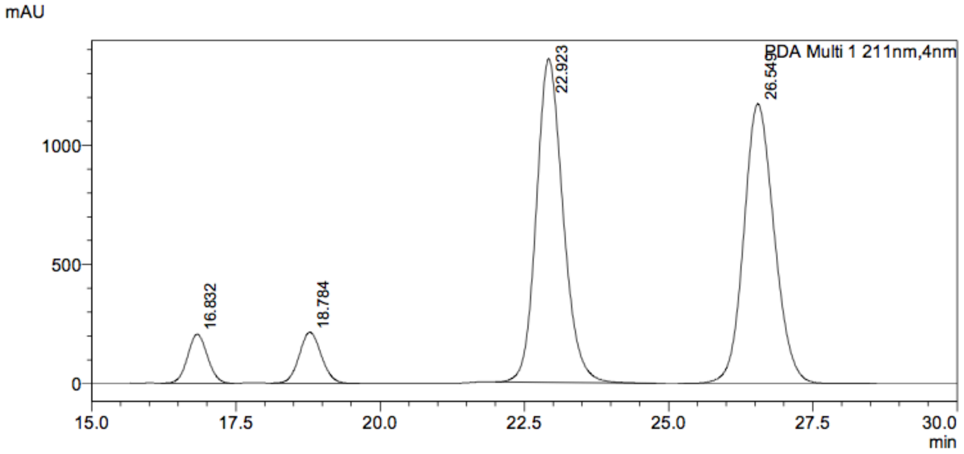

<Peak Table>

PDA Ch1 211nm

Peak#	Ret. Time	Area%
1	16.832	5.059
2	18.784	5.869
3	22.923	44.933
4	26.549	44.139
Total		100.000

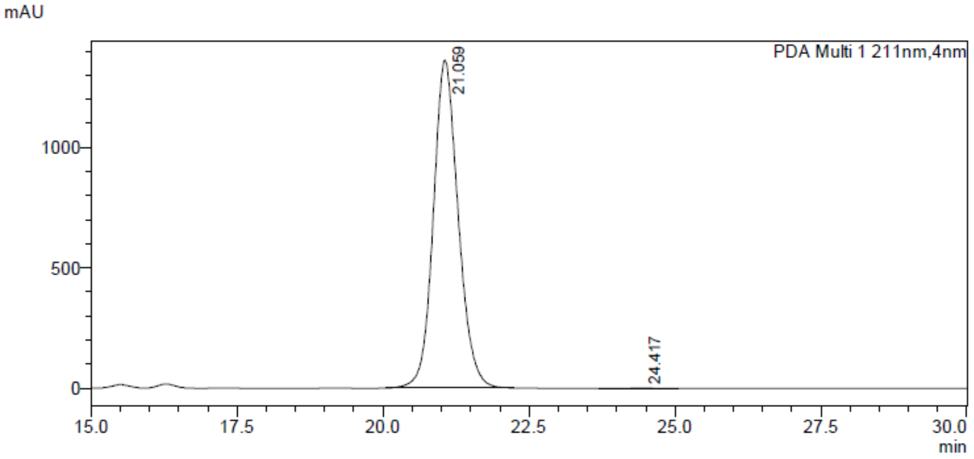

<Peak Table>

PDA Ch1 211nm

Peak#	Ret. Time	Area%
1	21.059	99.973
2	24.417	0.027
Total		100.000

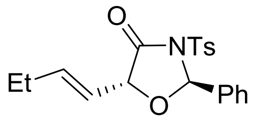

anti-**30a**

Chiral HPLC analysis, AD-H (90:10 hexane : IPA, flow rate 1.0 mL min^{-1}, 211 nm, 30 °C), t_R (2*R*,5*R*): 12.1 min, t_R (2*S*,5*S*): 27.6 min, >99% ee.

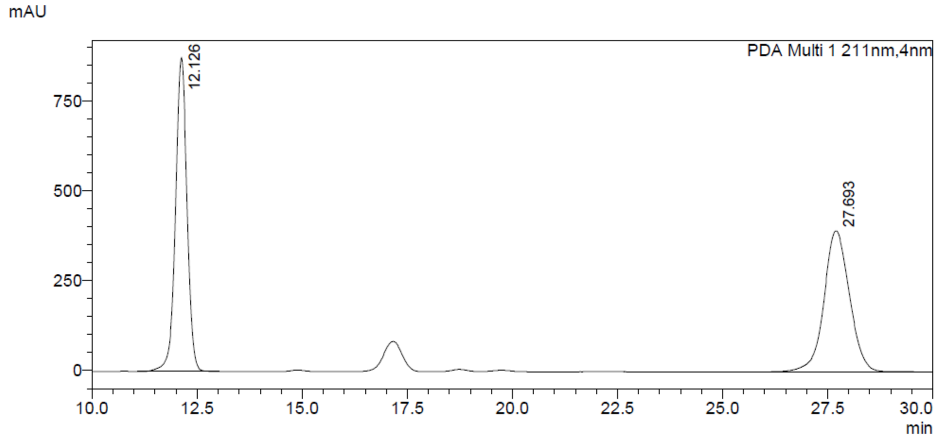

<Peak Table>

PDA Ch1 211nm

Peak#	Ret. Time	Area%
1	12.126	50.174
2	27.693	49.826
Total		100.000

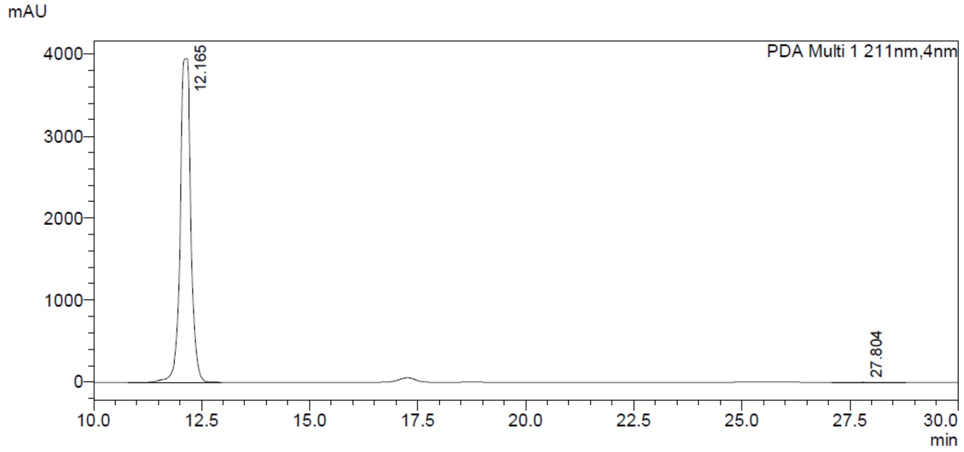

<Peak Table>

PDA Ch1 211nm

Peak#	Ret. Time	Area%
1	12.165	99.934
2	27.804	0.066
Total		100.000

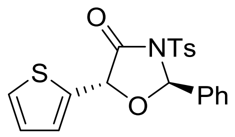

31a

Chiral HPLC analysis, AD-H (80:20 hexane : IPA, flow rate 1.0 mL min^{-1}, 211 nm, 30 °C), t_R (2*R*,5*R*): 17.1 min, t_R (2*S*,5*S*): 33.5 min, >99% ee.

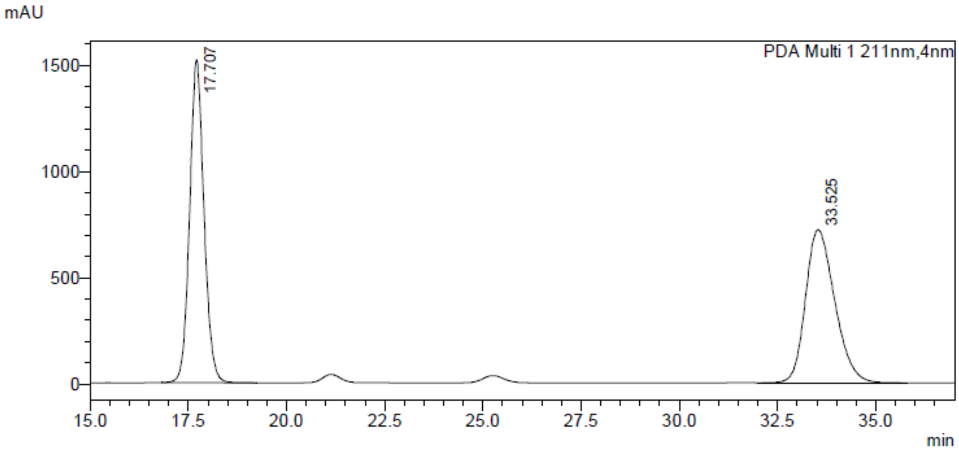

<Peak Table>

PDA Ch1 211nm

Peak#	Ret. Time	Area%
1	17.707	50.852
2	33.525	49.148
Total		100.000

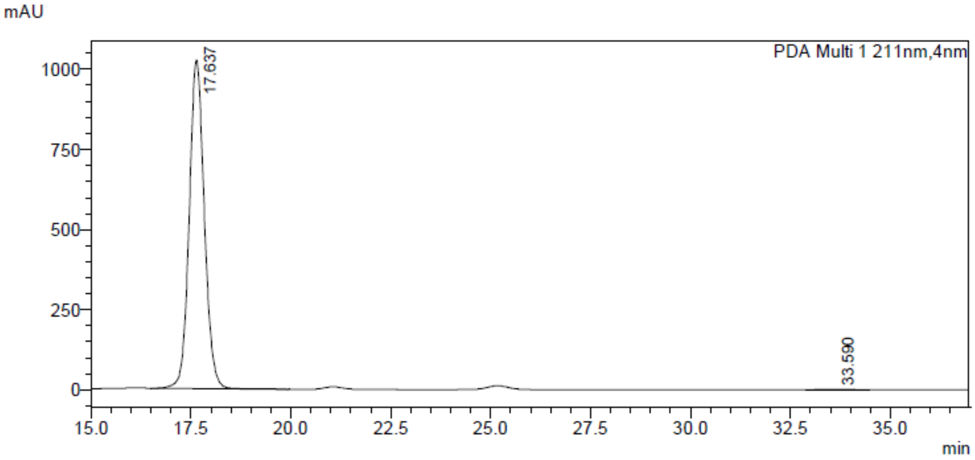

<Peak Table>

PDA Ch1 211nm

Peak#	Ret. Time	Area%
1	17.637	99.818
2	33.590	0.182
Total		100.000

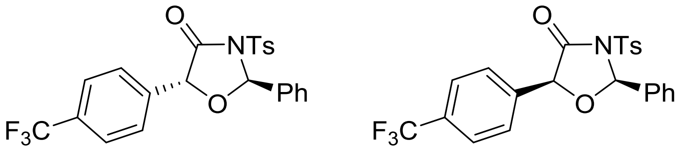

anti-**25a** *syn*-**25b**

Data for the anti diastereoisomer: Chiral HPLC analysis, Chiralcel OD-H (90:10 hexane:IPA, flow rate 1.0 mL min^{-1}, 211 nm, 30 °C) t_R (2*S*,5*S*): 19.5 min, t_R (2*R*,5*R*): 25.2 min, >99% ee.

Data for the syn diastereoisomer: Chiral HPLC analysis, Chiralcel OD-H (90:10 hexane:IPA, flow rate 1.0 mL min^{-1}, 211 nm, 30 °C) t_R (2*S*,5*R*): 15.8 min, t_R (2*R*,5*S*): 36.0 min, >99% ee.

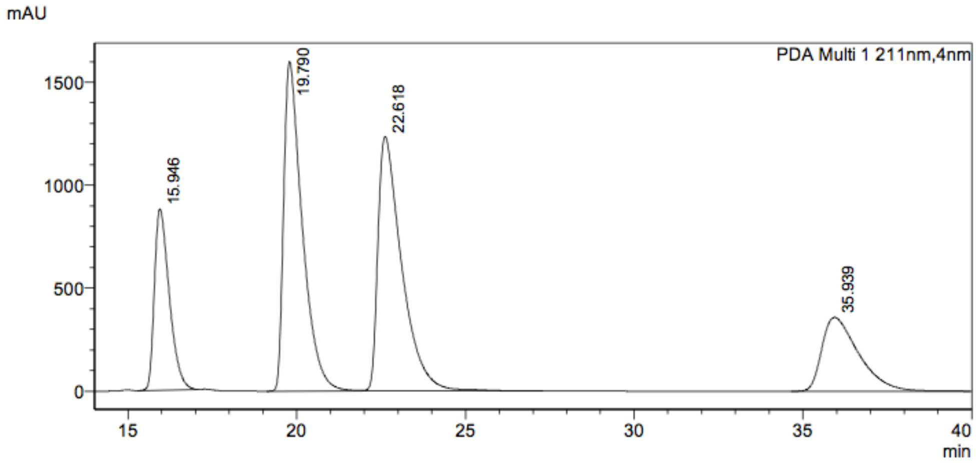

<Peak Table>

PDA Ch1 211nm

Peak#	Ret. Time	Area%
1	15.946	15.311
2	19.790	34.826
3	22.618	34.468
4	35.939	15.395
Total		100.000

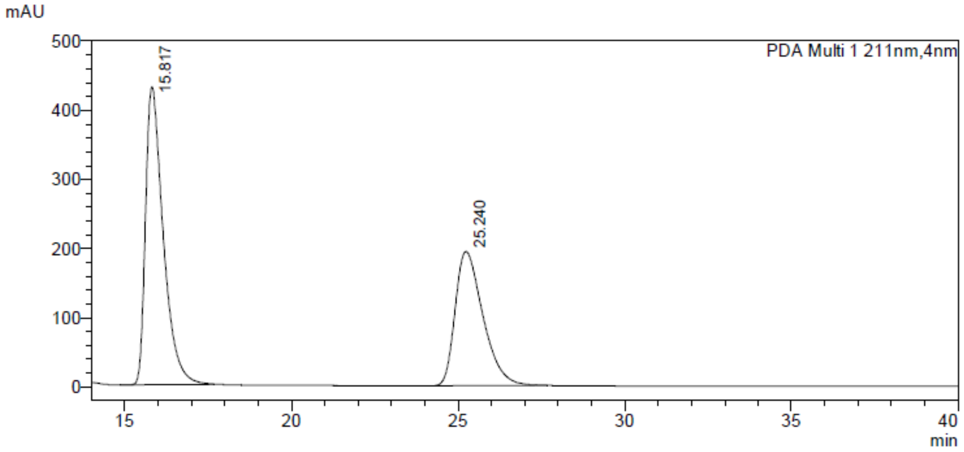

<Peak Table>

PDA Ch1 211nm

Peak#	Ret. Time	Area%
1	15.817	58.468
2	25.240	41.532
Total		100.000

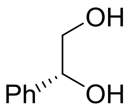

16

Chiral HPLC analysis, Chiralcel OD-H (95:5% hexane : IPA, flow rate 1.0 mL min^{-1}, 211 nm, 30 °C), t_R(*R*): 23.4 min, t_R(*S*): 26.8 min, 90% ee.

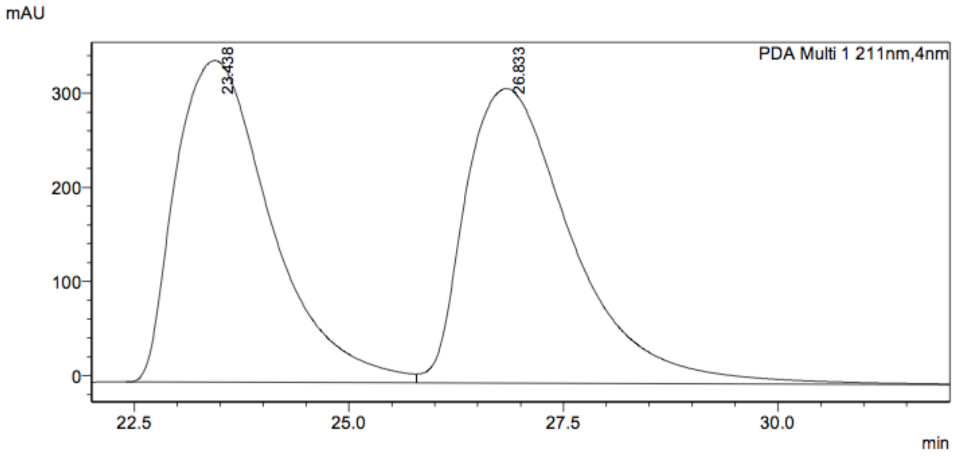

<Peak Table>

PDA Ch1 211nm

Peak#	Ret. Time	Area%
1	23.438	49.315
2	26.833	50.685
Total		100.000

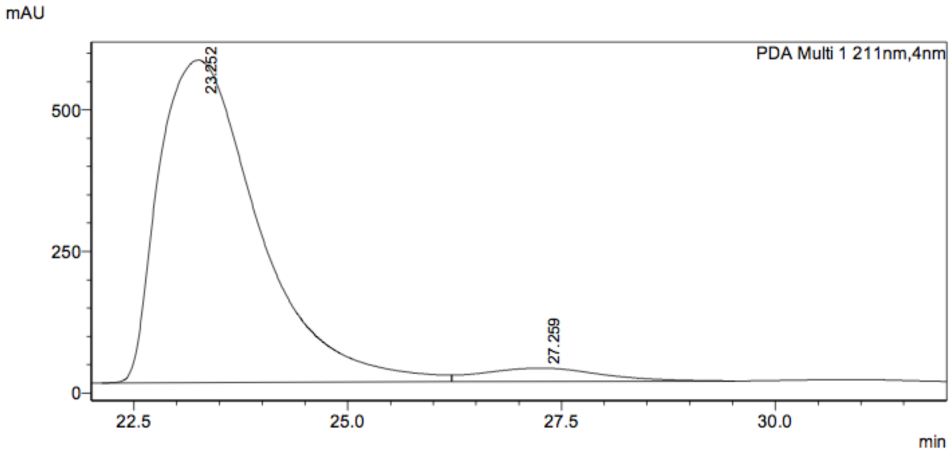

<Peak Table>

PDA Ch1 211nm

Peak#	Ret. Time	Area%
1	23.252	95.276
2	27.259	4.724
Total		100.000

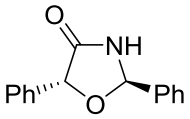

32

Chiral HPLC analysis, Chiralpak AD-H (90:10 hexane : IPA, flow rate 1.0 mLmin^{-1}, 211 nm, 30 °C) t_R (2*R*,5*R*): 10.6 min, t_R (2*S*,5*S*): 14.3 min, 99% ee.

PDA Ch1 211nm

Peak#	Ret. Time	Area%
1	10.501	49.597
2	14.211	50.403
Total		100.000

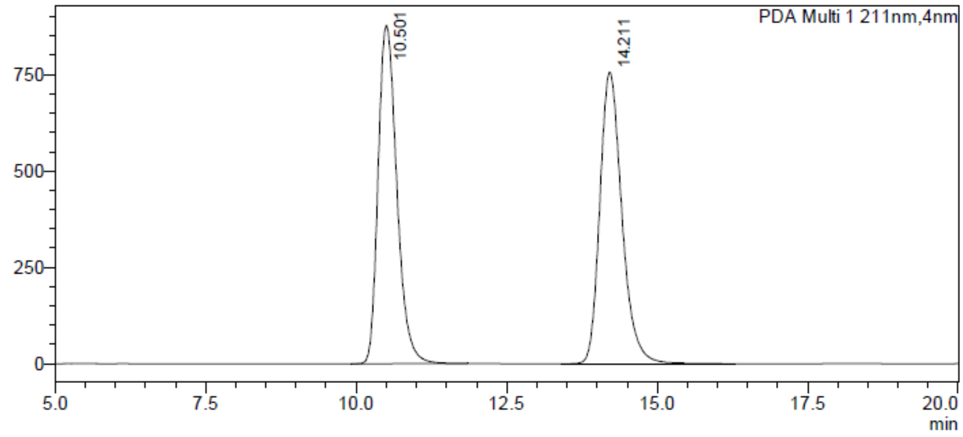

PDA Ch1 211nm

Peak#	Ret. Time	Area%
1	10.588	99.866
2	14.334	0.134
Total		100.000

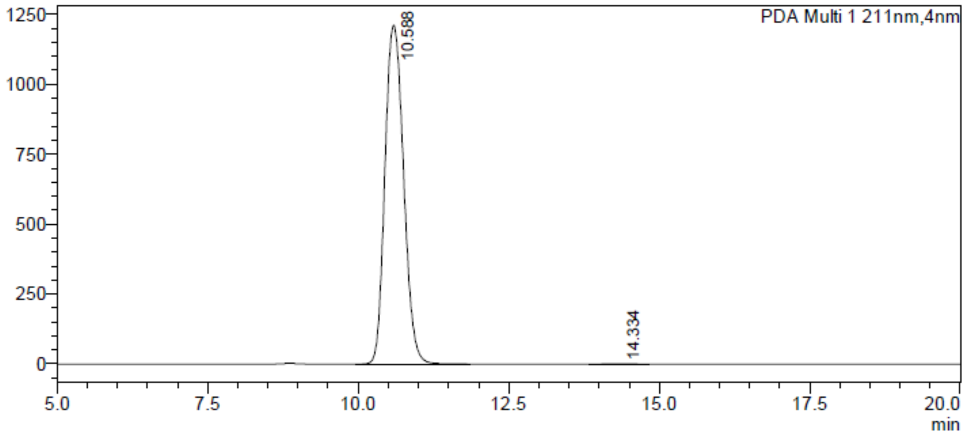

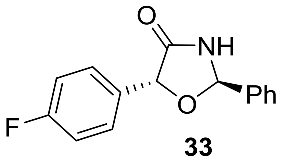

33

Chiral HPLC analysis, Chiralcel AD-H (90:10 hexane:IPA, flow rate 1.0 mL min^{-1}, 211 nm, 30 °C) t_R (2*R*,5*R*): 10.6 min, t_R (2*S*,3*S*): 14.4 min, >99% ee.

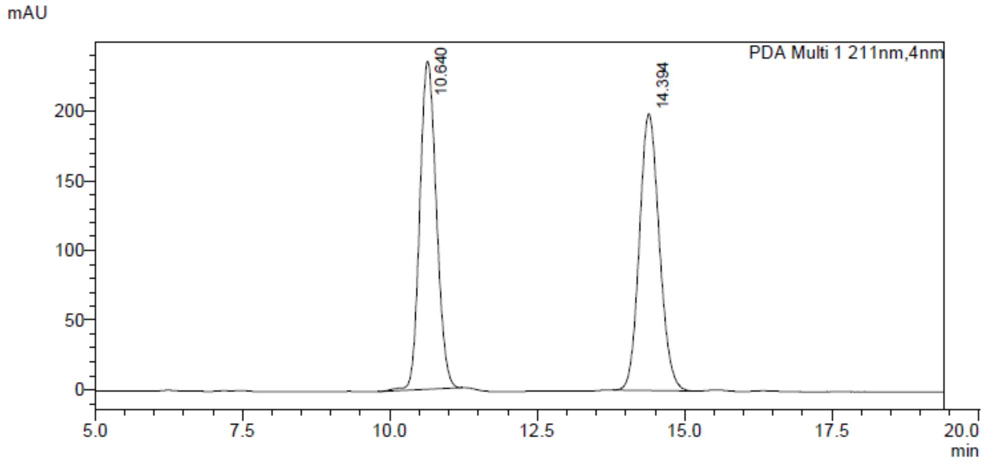

<Peak Table>

PDA Ch1 211nm

Peak#	Ret. Time	Area%
1	10.640	49.782
2	14.394	50.218
Total		100.000

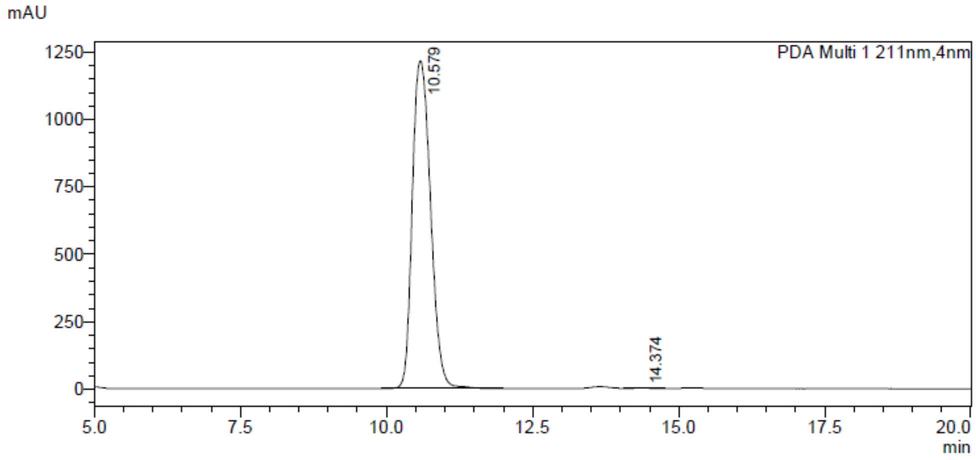

<Peak Table>

PDA Ch1 211nm

Peak#	Ret. Time	Area%
1	10.579	99.888
2	14.374	0.112
Total		100.000

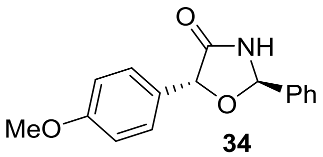

34

Chiral HPLC analysis, Chiralpak AD-H (90:10 hexane : IPA, flow rate 1.0 mLmin^{-1}, 211 nm, 30 °C) t_R (2*R*,5*R*): 17.8 min, t_R (2*S*,5*S*): 24.0 min, 99% ee.

PDA Ch1 211nm

Peak#	Ret. Time	Area%
1	17.879	49.960
2	24.007	50.040
Total		100.000

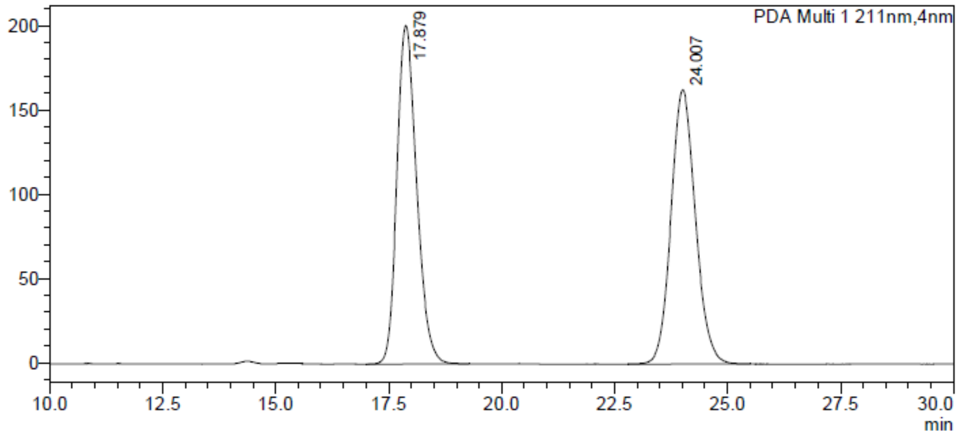

PDA Ch1 211nm

Peak#	Ret. Time	Area%
1	17.801	99.937
2	24.003	0.063
Total		100.000

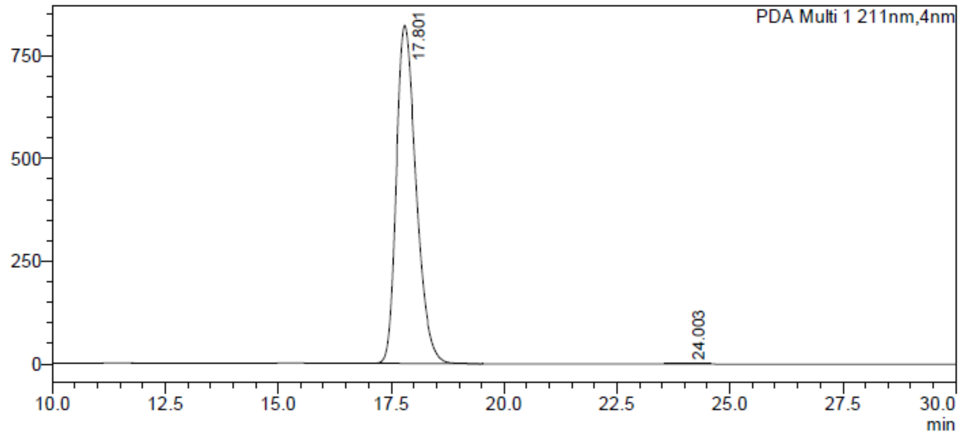

Supplement: Supplementary file 1 — miscellaneous_information [file chem0021-10530-sd1.pdf]
